# Assessment of forest restoration with multitemporal remote sensing imagery

**DOI:** 10.1038/s41598-019-43544-5

**Published:** 2019-05-13

**Authors:** Cheng-Chien Liu, Yi-Hsin Chen, Mei-Heng Margaret Wu, Chiang Wei, Ming-Hsun Ko

**Affiliations:** 1 Department of Earth Sciences, National Cheng Kung University, Tainan, 701 Taiwan; 2 Global Earth Observation and Data Analysis Center, National Cheng Kung University, Tainan, 701 Taiwan; 3 Department of Geography, University of California, Los Angeles, USA; 4 Experimental Forest, College of Bio-Resources and Agriculture, National Taiwan University, Nantou, Taiwan

**Keywords:** Climate-change mitigation, Forestry, Natural hazards

## Abstract

Climate variability and man-made impacts have severely damaged forests around the world in recent years, which calls for an urgent need of restoration aiming toward long-term sustainability for the forest environment. This paper proposes a new three-level decision tree (TLDT) approach to map forest, shadowy, bare and low-vegetated lands sequentially by integrating three spectral indices. TLDT requires neither image normalization nor atmospheric correction, and improves on the other methods by introducing more levels of decision tree classification with inputs from the same multispectral imagery. This approach is validated by comparing the results obtained from aerial orthophotos (25 cm) that were acquired at approximately the same time in which the Formosa-2 images (8 m) were being taken. The overall accuracy is as high as 96.8% after excluding the deviations near the boundary of each class caused by the different resolutions. With TLDT, the effectiveness of forest restoration at 30 sites are assessed using all available multispectral Formosat-2 images acquired between 2005 and 2016. The distinction between natural regeneration and regrowth enhanced by restoration efforts were also made by using the existing dataset and TLDT developed in this research. This work supports the use of multitemporal remote sensing imagery as a reliable source of data for assessing the effectiveness of forest restoration on a regular basis. This work also serves as the basis for studying the global trend of forest restoration in the future.

## Introduction

In recent years, recurring natural disasters such as wildfire and landslides have severely damaged forest ecosystems, resulting in a loss of habitat for numerous species, significant soil erosion and changes in land cover^1^. This condition is aggravated by the current climate variability and man-made impacts including timber harvest and fire succession that are detrimental to the forest environment on an even more massive scale^2^. The current scale of deforestation all around the world calls for an urgent need to restore biodiversity and the ecological structure and functioning, aiming toward long-term sustainability for the forest environment^3^. Although many countries’ governments and various environmental management sectors have launched initiatives on restoring forest ecosystems, they lack a systematic and synoptic view for monitoring the effects of forest restoration^3^. Thus, it had become increasingly important to assess the effectiveness of forest restoration and monitor the posttreatment abiotic and biotic characteristics of the landscape^1,3^. Without an effective monitoring system, there is neither sufficient information provided on the impact on the restoration efforts, nor is there any basis for further improvements^3^.

Taiwan is located in the center of the East-Asian island arc formed by the slow collision of the Asian continental plate and the Philippine plate. The high mountains, broken terrain and frequent earthquakes, together with the heavy rainfall during the rainy and typhoon seasons, results in a very high erosion rates in the world^4^. Consequently, more than 90% of the country’s population lives in areas that are at a relatively high risk of typhoons, earthquakes and landslides^5^. For example, Typhoon Morakot brought an extreme precipitation of 2,777 mm in less than a week in August 2009^6^, and triggered enormous landslides that caused massive destructions to the landscapes. To stabilize those areas affected by landslides found in the mountainous region, the Forestry Bureau of Taiwan (FBT) launched initiatives and utilized various techniques on restoring forest. The traditional method for evaluating forest restoration is *in situ* site surveying twice per year, once in the summer and once in the winter to monitor vegetation recovery. However, for these inaccessible sites with large-scale restoration, this method is neither cost- nor labour-effective, in terms of providing a comprehensive and up-to-date review of the results of forest restoration.

By contrast, remote sensing imagery is more advantageous in assessing forest restoration due to its ability to detect changes in large areas over long periods of time that are difficult to observe from the ground^3,7^. Although the updated optical instrumentation like hyperspectral imagers^8^ and light detection and ranging (LIDAR) scanners^9^ provide more information than the multispectral sensor does, it would be too costly and impractical to employ those updated sensors to detect changes in large areas over long periods of time, in our case, the mountain area of Taiwan between 2005 to 2016. Taking these factors into consideration, analyzing the multispectral and multitemporal imagery is still the most feasible and practical approach to assess the forest restoration, particularly the Landsat imagery collected by the Landsat program tracing back to 1972^10,11^. To facilitate the production of image-ready-to-use quality Landsat time series stacks, Huang *et al*.^12^ developed a streamlined approach that includes an image selection protocol, updated radiometric calibration and atmospheric correction for calculating surface reflectance, as well as precision registration and orthorectification routines for improving geolocation accuracy. Together with a highly automated algorithm called vegetation change tracker, Huang *et al*.^13^ applied their streamlined approach to reconstruct the forest disturbance history based on the spectral-temporal properties of land cover and forest change processes. Note that such an approach relies on the updated radiometric calibration and atmospheric correction for calculating surface reflectance, yet the associated atmospheric properties at the time of image acquisition are usually unavailable or difficult to obtain for the high-spatial-resolution sensors with only four or five spectral bands, such as IKONOS, Quickbird, Formosat-2 and SPOT-6/7. There is no consideration of topographic shadows nor relief shadows in this kind of approach either. Giles^14^ pointed out that shadows are inevitably found as main features in an optical imagery of mountainous areas, and they can occupy as much as 30% of an entire image acquired in winter in Taiwan^15^. They would be even more detailed and clear in the high-spatial-resolution imagery. Therefore, special care of shadows should be taken when dealing with high-spatial-resolution imagery.

The classification and regression trees (CART) approach, on the other hand, requires neither image normalization nor atmospheric correction to determine thresholds for disturbance or regrowth. Helmer *et al*.^9^ developed an automated procedure threshold age mapping algorithm to isolate the lowland forests by separately mapping land cover and old growth forest types with two decision tree classifications. Olsson^16^ found the deviation in reflectance development between different types of forest plantations could be characterized by fitting a linear regression model through the bandwise spectral mean values for each stand, starting 5 years after the final felling. Li and Fox^17^ mapped the distribution of rubber tree growth across this mainland Southeast Asia landscape using the standard MODIS product. These works demonstrate that CART approach is advantageous in analyzing the multispectral and multitemporal imagery. More levels of decision tree classifications enable us to separate more types of land cover, but this would require more information by either using data collected from other sensors^12^ or introducing other spectral indices derived from the same multispectral imagery.

The process of forest restoration that is revealed from the time series of remote sensing imagery is a gradual transition from bare land (*BL*) to low-vegetated land (*LVL*), and eventually, forest land (*FL*) that is similar to the trees of background. As time progresses, the area ratios of forest land (*A*_*FL*_), and low-vegetated land (*A*_*LVL*_), gradually expand while the area ratio of bare land (*A*_*BL*_), slowly decreases. Since most of the remote sensing imagery are acquired while the sun is not in the nadir direction, shadowy land (*SL*) is one of the main features that are inevitably found in an optical imagery over mountainous areas^14^, and it should be excluded from the calculation of *A*_*FL*_ and *A*_*BL*_. Thus, to assess the effectiveness of forest restoration, a sound approach to map *FL*, *SL*, *BL* and *LVL* and an accurate calculation of area ratios *A*_*FL*_, *A*_*SL*_, *A*_*BL*_ and *A*_*FL*_ from the multitemporal remote sensing imagery are required. This paper proposes a three-level decision tree (TLDT) approach to map *FL*, *SL*, *BL* and *LVL* sequentially and calculate the area ratios *A*_*FL*_, *A*_*SL*_, *A*_*BL*_ and *A*_*FL*_ from the remote sensing imagery with multispectral bands, by integrating three spectral indices: the normalized difference vegetation index (*NDVI*), shadow index (*SI*), and normalized green red difference indices (*NGRDI*). This new TLDT approach belongs to the category of CART approach and improves on the other methods by introducing more levels of decision tree classification with inputs from the same multispectral imagery. Like the other CART approaches, TLDT requires neither image normalization nor atmospheric correction. However, we found that the process of radiance normalization provides an appropriate way of examining the quality of every image. With this new TLDT approach, the effectiveness of forest restoration at 30 sites are assessed, using all available multispectral Formosat-2 images acquired between 2005 and 2016 pre-processed by the Formosat-2 automatic image processing system (F-2 AIPS)^18^.

The assessments of 15 sites are compared with the results obtained from the high spatial resolution (25 cm) aerial orthophotos that were acquired at approximately the same time in which the Formosa-2 images (8 m) were being taken. These comparisons are not intended to verify the assessment accuracy but to clarify the reasons for discrepancy, since the spatial resolutions are rather different between the aerial orthophoto (25 cm) and Formosat-2 image (8 m). After excluding the deviations near the boundary of each class caused by the different resolutions between Formosat-2 imagery and Aerial orthophoto, the overall accuracy is as high as 96.8%. Among the 30 study sites, 10 have been restored successfully, 4 are recovering slowly, and 4 have hardly re-vegetated. For the rest of the 12 sites, the shaded areas are too large to derive a detailed trend of restoration. But the effectiveness can still be assured by comparing the pre-restoration and the most up-to-date Formosat-2 images. The distinction between natural regeneration and regrowth enhanced by restoration efforts were also made by using the existing dataset and the TLDT approach developed in this research. The result highlights the importance of restoration for it accelerates the natural regeneration to at least four times. This work supports the use of multitemporal remote sensing imagery as a reliable source of data for assessing the effectiveness of forest restoration on a regular basis. This work also serves as the basis for studying the global trend of forest restoration in the future.

## Material

### Study sites

**Figure 1:**
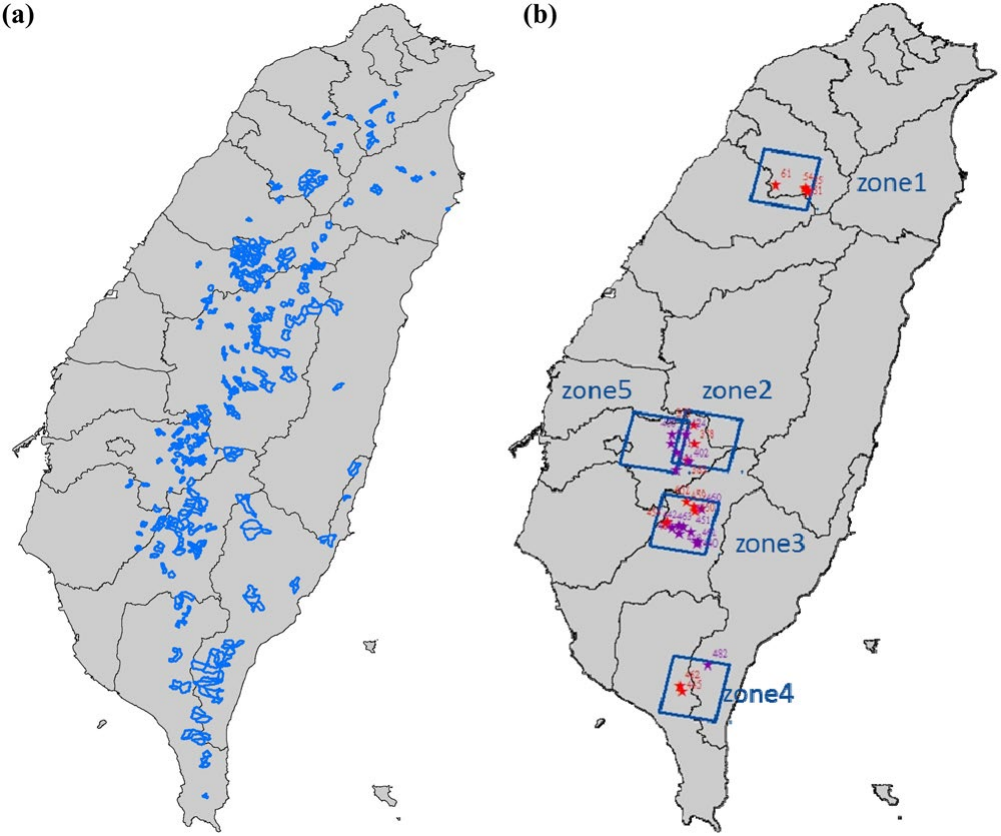
(**a**) Geographical locations of 511 sites (blue polygons) of forest restoration accomplished by FBT. (**b**) 30 study sites are denoted as star symbols and grouped into five zones (blue boxes). 15 of 30 study sites are marked as purple star symbols and used as test sites for validation.

A total of 511 sites of forest restoration in Taiwan have been accomplished so far by FBT. Their geographical locations are labeled as blue polygons in Fig. 1(a). Considering the cost and availability, the major source of satellite imagery for this work is from Formosat-2, which was operated by National Space Organization of Taiwan from 2004 to 2016. Therefore, the timing of restoration in the study site has to be later than 2004 so the full process of restoration can be covered by the span of Formosat-2 mission. In addition, the area of restoration should be large enough to ensure that the gradual changes can be captured by the 2-m resolution of Formosat-2 imagery. Taking these conditions into consideration, a total of 30 study sites are selected and grouped into five zones, as marked as star symbols and blue boxes in Fig. 1(b). The detailed descriptions of each site including the restoration year and engineering method are listed in Table 1. Note that the results of restoration effectiveness assessed by this work are also listed in Table 1 for comparison. Together with the overall accuracy of 15 test sites, these results will be explained in detail later.

**Table 1:** Descriptions of 30 study sites, of which 15 sites with orthorectified aerial orthophotos are selected as test sites and marked as purple star symbols in Fig. 1(b).

No.	District Office	Working station	Zone	Area (ha)	Restoration Year	Restoration Engineering method*	Restoration Effectiveness**	Overall Accuracy
51	Hsinchu	Daxi	1	30.19	2006	A, B, C	IV	N/A
54	Hsinchu	Daxi	1	13.17	2007	A, B, C	IV	N/A
55	Hsinchu	Daxi	1	34.41	2008	A, B, C	IV	N/A
61	Hsinchu	Zhudong	1	10.92	2012	B, E	I	N/A
355	Chiayi	Alishan	2	47.53	2000	B, D	IV	N/A
378	Chiayi	Dapu	2	26.28	2010	B	I	N/A
385	Chiayi	Dapu	2	14.04	2011	B	I	N/A
430	Pingtung	Chishan	4	14.76	2010	A	I	N/A
431	Pingtung	Chishan	4	9.34	2010	A	IV	N/A
445	Pingtung	Chaozhou	3	49.30	2011	B	III	N/A
450	Pingtung	Chishan	4	300.77	2011	B	III	N/A
452	Pingtung	Chishan	4	40.02	2011	B	I	N/A
454	Pingtung	Luokuei	4	28.27	2011	A, D	IV	N/A
459	Pingtung	Chishan	4	120.81	2012	B	II	N/A
462	Pingtung	Chaozhou	3	74.70	2012	B	III	N/A
391	Chiayi	Dapu	5	7.13	2011	B	IV	78.16%
400	Chiayi	Alishan	5	6.51	2011	B	IV	75.96%
402	Chiayi	Dapu	2	10.59	2011	B	I	72.23%
411	Chiayi	Dapu	5	13.29	2012	B	I	81.11%
415	Chiayi	Alishan	5	6.00	2012	B	IV	82.48%
424	Chiayi	Alishan	5	26.01	2012	B	IV	69.46%
435	Pingtung	Chishan	4	21.63	2010	B, C, E	I	69.14%
440	Pingtung	Laonong River	4	11.23	2010	B	II	82.31%
442	Pingtung	Chishan	4	49.38	2011	B	I	87.51%
443	Pingtung	Chishan	4	14.94	2011	B	I	80.18%
451	Pingtung	Chishan	4	25.07	2011	B, C, E	IV	74.26%
460	Pingtung	Chishan	4	67.77	2012	B	II	81.72%
461	Pingtung	Laonong River	4	122.05	2012	B	III	83.07%
463	Pingtung	Chishan	4	33.62	2012	B	IV	77.67%
482	Taitung	Dawu	3	11.07	2010	B	II	79.30%

^*^A: staking and wattling; B: seeds spreading; C: drainage of longitudinal and transverse; D: tree planting; E: Cover grass net. ^**^I: restored well apparently; II: restored in a slow pace; III: restored inefectively; IV: no assessment.

### Remote sensing imagery

Formosat-2 is the first satellite with a high-spatial-resolution (2 m) sensor placed in a daily revisit orbit, as well as the second satellite that is owned and operated by the National Space Organization (NSPO), Taiwan^18,19^. The remote sensing instrument (RSI) onboard Formosat-2 acquires 2-m resolution panchromatic images and 8-m resolution multispectral images in four multispectral bands (blue, green, red and near-infrared) over 24 km swath width in the nadir direction. The spectral bands definition and the spectral radiances at the entrance aperture, including the mean radiance and the saturation radiance can be referred to Table 2 of Liu *et al*.^19^. RSI has a field of regard of ±45 deg for along-track and cross-track viewing, and our study areas are covered by Strip Nos. 3, 4 and 5 of Orbit 1. During twelve years of operations and services from 2005 to 2016, Formosat-2 has acquired a total of 1,453 strips covered our study areas. All quick-look images (Strip Nos. 3, 4 and 5 of Orbit 1) are annotated with the dates of acquisition and given in the supplement A. After excluding those images with mostly clouds and haze, there are 42, 37, 31, 28 and 26 available images for zone 1 to 5 respectively, as listed in Table 2. Note that four seasons are indeed covered in the entire time series of images for every zone. But it is not the case for every zone to have images for every season in every year.

**Table 2:** List of all available Formosat-2 imagery with detailed dates for Zone 1 to 5, after excluding those images with mostly clouds and haze.

Zone 1	Zone 2	Zone 3	Zone 4	Zone 5
20110210:4	20050801:4	20090721:5	20090203:4	20090412:3
20110917:4	20051106:4	20090902:5	20090817:4	20091031:3
20111204:4	20060201:4	20091215:5	20090824:4	20100131:3
20120103:4	20060729:4	20100111:5	20091105:4	20100521:3
20120327:4	20061126:4	20100410:5	20100223:4	20100921:3
20121019:4	20070219:4	20101118:5	20100306:4	20110205:3
20130305:4	20070508:4	20110227:5	20100409:4	20110609:3
20130705:4	20070703:4	20110613:5	20100923:4	20111130:3
20131123:4	20080622:4	20110926:5	20101121:4	20120201:3
20140117:4	20081115:4	20111027:5	20110416:4	20120514:3
20140410:4	20090203:4	20111218:5	20110817:4	20121025:3
20140825:4	20090824:4	20120326:5	20120103:4	20130116:3
20141123:4	20091023:4	20120702:5	20121017:4	20130317:3
20150115:4	20091212:4	20121022:5	20121221:4	20130919:3
20150406:4	20100306:4	20130119:5	20130217:4	20131231:3
20150609:4	20100810:4	20130308:5	20130705:4	20140223:3
20151219:4	20100911:4	20130810:5	20131030:4	20140815:3
20160301:4	20101121:4	20131202:5	20140101:4	20141122:3
20050816:5	20110416:4	20140225:5	20140222:4	20150111:3
20060131:5	20110817:4	20140712:5	20141123:4	20150514:3
20060801:5	20111217:4	20140713:5	20150125:4	20151028:3
20061209:5	20120103:4	20150124:5	20150609:4	20160126:3
20070310:5	20120707:4	20150318:5	20151219:4	20160420:3
20070507:5	20121021:4	20150619:5	20160407:4	20091023:4
20070721:5	20130115:4	20150913:5	20120201:3	20100129:4
20080610:5	20130316:4	20151122:5	20120514:3	20100911:4
20080826:5	20130827:4	20160328:5	20121023:3	
20081221:5	20131123:4	20160507:5	20121217:3	
20090204:5	20140117:4	20111217:4		
20090721:5	20140317:4	20120328:4		
20090902:5	20141123:4	20121108:4		
20091213:5	20150115:4			
20100301:5	20150320:4			
20100704:5	20150609:4			
20101201:5	20151107:4			
20110301:5	20160107:4			
20110708:5	20160407:4			
20130711:5				
20140225:5				
20140712:5				
20141021:5				
20160402:5				

Convention: yyyymmdd:s. yyyy: year; mm: month; dd: date; s: Strip No.

**Figure 2:**
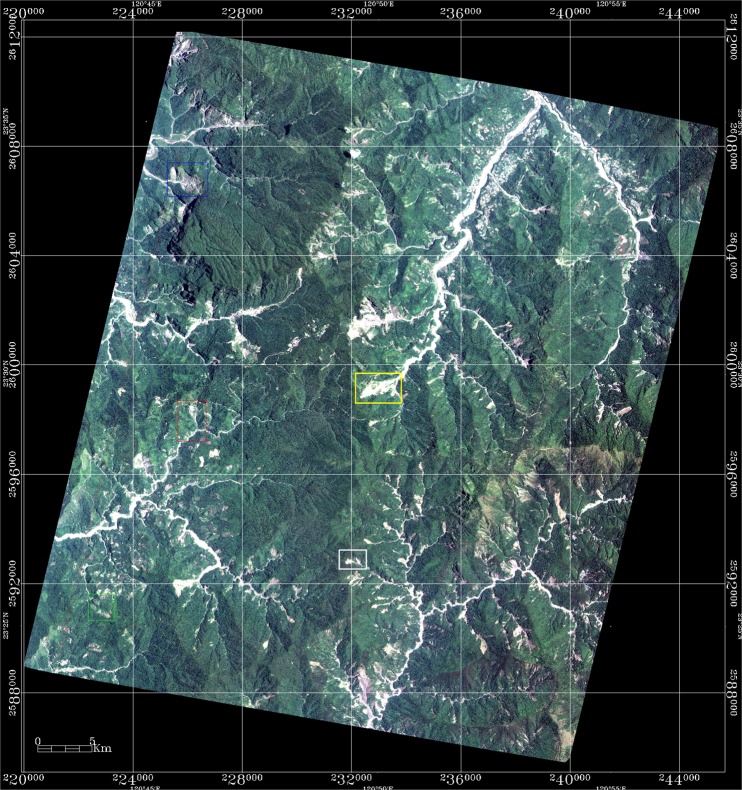
The true color images of zone 2 taken by Formosat-2 on 9 June 2015, where the regions of site No. 355, 378 and 385 are annotated as blue, red and green boxes; the regions of natural restoration sites NRS 1 and NRS 2 are annotated as white and yellow boxes, respectively. (Formosat-2 image ©National Space Organization of Taiwan).

All available Formosat-2 imagery of each zone are pre-processed by F-2 AIPS^18^. F-2 AIPS is able to digest the Gerald format of the raw data, apply the basic radiometric and geometric correction, output the level-1A product, conduct the rigorous band-to-band co-registration^20^, automatic orthorectification^21^, multitemporal image geometrical registration^22^, multitemporal image radiometric normalization^23^, spectral summation intensity modulation pan-sharpening^20^, and the absolute radiometric calibration^19^. One true color image of zone 2 taken by Formosat-2 on 9 June 2015 is shown in Fig. 2 as an example, of which the regions of site No. 355, 378 and 385 are annotated as blue, red and green boxes respectively. The time series of co-registered and radiance-normalized imagery are further divided into smaller rectangles so that each study site is fully enclosed by a small rectangle. Note that two landslides near site No. 378 are selected and labeled as the natural restoration site (NRS) 1 (white box) and 2 (yellow box) in Fig. 2. These landslides were also triggered by Typhoon Morakot but they were not selected as restoration sites by FBT. Therefore, the changes found at NRS 1 and 2 are purely the effect of natural regeneration, which can help us to gain insight into the difference between natural regeneration and restoration.

The general source of ground truth is usually collected on the ground by conducting a considerable amount of *in situ* survey. Interpreting aerial colored images to obtain the ground truth information offers an alternative to this general approach^24^. For 15 of 30 study sites, the aerial orthophotos with 25 cm resolution and four spectral bands (blue, green, red, and near-infrared) were provided by FBT. Note that the position, attitude values, as well as digital image of the photograph at the moment of being taken were decoded by using Global Positioning System (GPS) and Inertial Measurement Unit (IMU). Together with the Digital Terrain Model (DTM), the center projected aerial photographs were rectified pixel by pixel into orthophotos. These orthophotos are resampled to 2 m resolution and co-registered with their corresponding Formosat-2 imagery, to serve as an alternative source of ground truth. These 15 sites are marked as purple star symbols in Fig. 1(b) and used as test sites to validate our approach.

## Method

For detecting the changes of land cover, and in our case, assessing the effectiveness of forest restoration, the radiometric normalization of multitemporal satellite optical images of the same terrain is often necessary^25^. Absolute radiometric correction requires an atmospheric correction algorithm and the associated atmospheric properties at the time of image acquisition, but this information is either unavailable or difficult to obtain^26^, particularly for Formosat-2 imagery that has only four spectral bands with broad bandwidth. Therefore, we attempt to employ the CART approach that requires neither image normalization nor atmospheric correction to determine thresholds for disturbance or regrowth. However, we also found that the process of radiance normalization provides an appropriate way of examining the quality of every image.

### Radiance normalization

**Figure 3:**
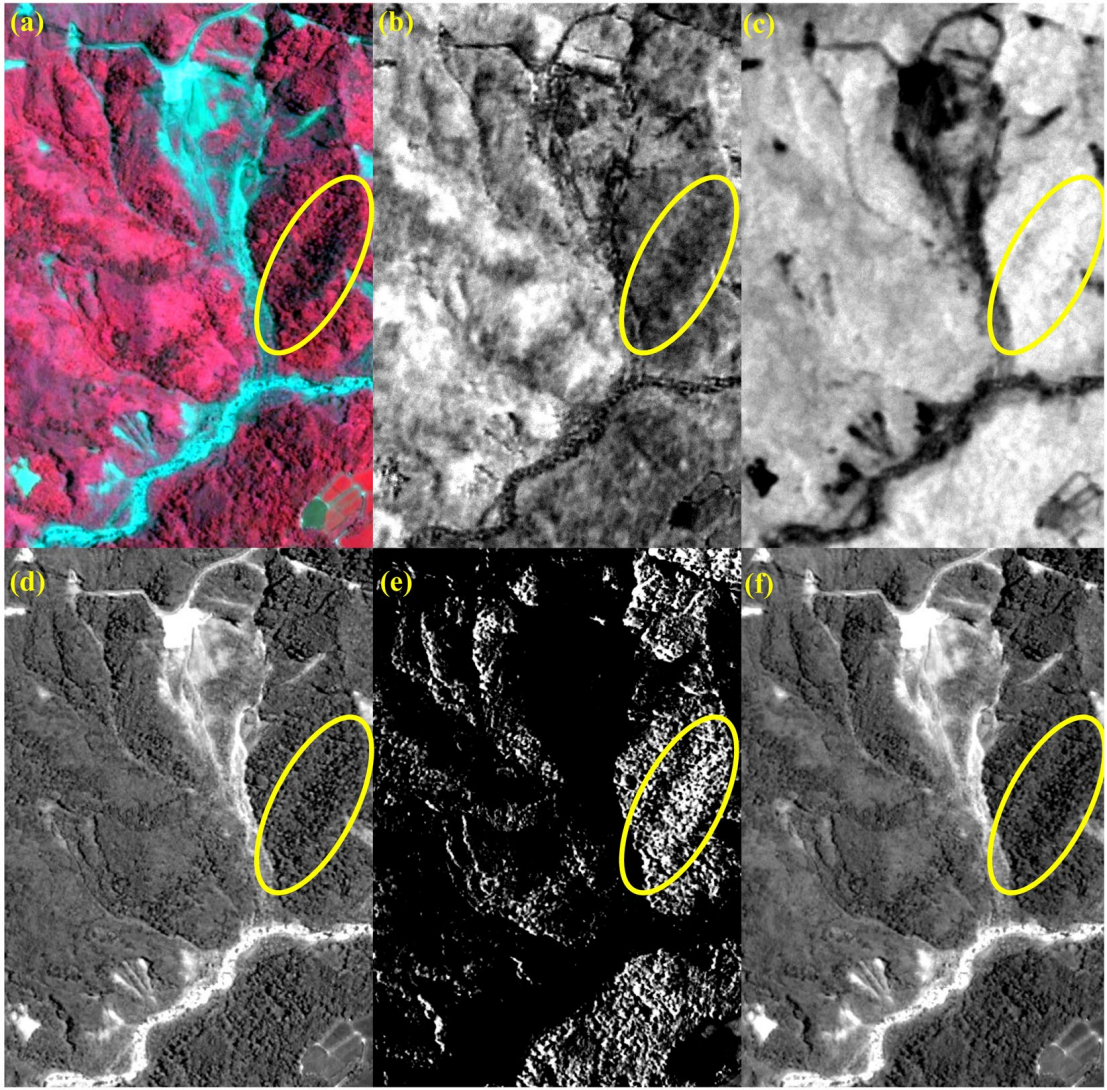
Illustration of how SI enhances the contrast between shadow and non-shadow regions. (**a**) Standard false colour image, (**b**) hue, (**c**) intensity, (**d**) saturation, (**e**) SI, and (**f**) PC1. The case of study site No. 378 (annotated as the red box in Fig. 2) (Formosat-2 image ©National Space Organization of Taiwan).

A total of 36 images of site No. 378 (located in Zone 2) acquired by Formosat-2 between 2005 and 2016 are collected and pre-processed by F-2 AIPS^18^. The one taken on 9 June 2015 (Fig. 2) is selected as the base image of radiance normalization^27^, based on the technique of searching the pseudo invariant features (PIFs)^25^. Using the image taken on 7 July 2012 as an example, the DN values of four spectral bands collected at all searched PIFs before (cross marks) and after (circle marks) the process of radiance normalization are shown in Fig. 3(a). This scatter plot demonstrates that a consistent and robust correlation can be established from these PIFs to meet the requirement of radiance normalization for change detection^28^. By contrast, the image taken on 15 January 2013 gives an example that fails to give a one-to-one relationship because the image was taken with a narrow dynamic range, as shown in Fig. 3(b). Therefore, the process of radiance normalization provides an appropriate way to examine the quality of every image. All scatter plots of the other 34 images are given in the supplement B, of which a total of 19 images with poor quality are excluded from the time series analysis, as listed in Table 3.

**Table 3:** List of 19 images of Zone 2 that are excluded from the time series analysis due to poor quality.

No.	Date
1	20070219
2	20070508
3	20081115
4	20090203
5	20091023
6	20091212
7	20100911
8	20101121
9	20111217
10	20120103
11	20130115
12	20131123
13	20140117
14	20140317
15	20141123
16	20150115
17	20150320
18	20150609
19	20160107

### Three-level decision tree approach

Automatic classification of landslides from multispectral imagery has been progressing rapidly and a few practical approaches have been proposed in the past few years. There are some similarities and differences among these approaches. Both Yang *et al*.^29^ and Liu^30^ classified the bare/non-vegetated land by using the normalized difference vegetation index (*NDVI*).

1 $$NDVI\equiv \frac{NIR-Red}{NIR+Red},$$

for its superiority to differentiate vegetation. Ma *et al*.^31^ took the same idea but used the inverse normalized difference vegetation index (NDVI^*^)

2 $$NDV{I}^{\ast }\equiv 1-\frac{NIR-Red}{NIR+Red}\mathrm{.}$$

Since the scattering of visible and near-infrared light are different, the partially-shaded topography regions are often misinterpreted as landslides for *NDVI* or NDVI^*^ are distorted in these regions. Therefore, the normalized difference soil index (*NDSI*)^29^

3 $$NDSI\equiv \frac{Red-Green}{Red+Green},$$

the normalized green red difference indices (*NGRDI*)^30^

4 $$NGRDI\equiv \frac{Green-Red}{Green+Red},$$

and the inverse normalized difference soil index (NDSI^*^)^31^

5 $$NDS{I}^{\ast }\equiv \frac{Green-Blue}{Green+Blue},$$

are employed to assist the interpretation. *NDSI*, *NGRDI* and NDSI^*^ are essentially the spectral ratios of visible bands that have similar levels of scattering either inside or outside the partially-shaded topography regions. They are not as sensitive as *NDVI* or NDVI^*^, in terms of discriminating vegetation. However, they are ideal in identifying the partially-shaded topography regions from those misinterpreted bare/non-vegetated lands.

Regarding to classifying shadows, Yang *et al*.^29^ employed the first principal component (*PC1*), while Liu^30^ used the intensity of panchromatic band that is proportional to *PC1*^32^. Ma *et al*.^31^ took a further step to calculate the shadow index (*SI*):

6 $$SI\equiv \frac{(PC{{1}}_{nor}-I)\times \mathrm{(1}+S)}{PC{{1}}_{nor}+I+S},$$

where S and I are the saturation and intensity components respectively, after converting the Red-Green-Blue color model to the Hue-Saturation-Intensity color model^33^. *PC1*_*nor*_ is the normalized value of *PC1* according to

7 $$PC{{1}}_{nor}\equiv \{\begin{array}{ll}\frac{PC{1}}{max(PC{1})} & {\rm{PC}}{1}\, > \,0\\ \frac{PC{1}}{min(PC{1})} & {\rm{PC}}{1}\,\le \,0.\end{array}$$

**Figure 4:**
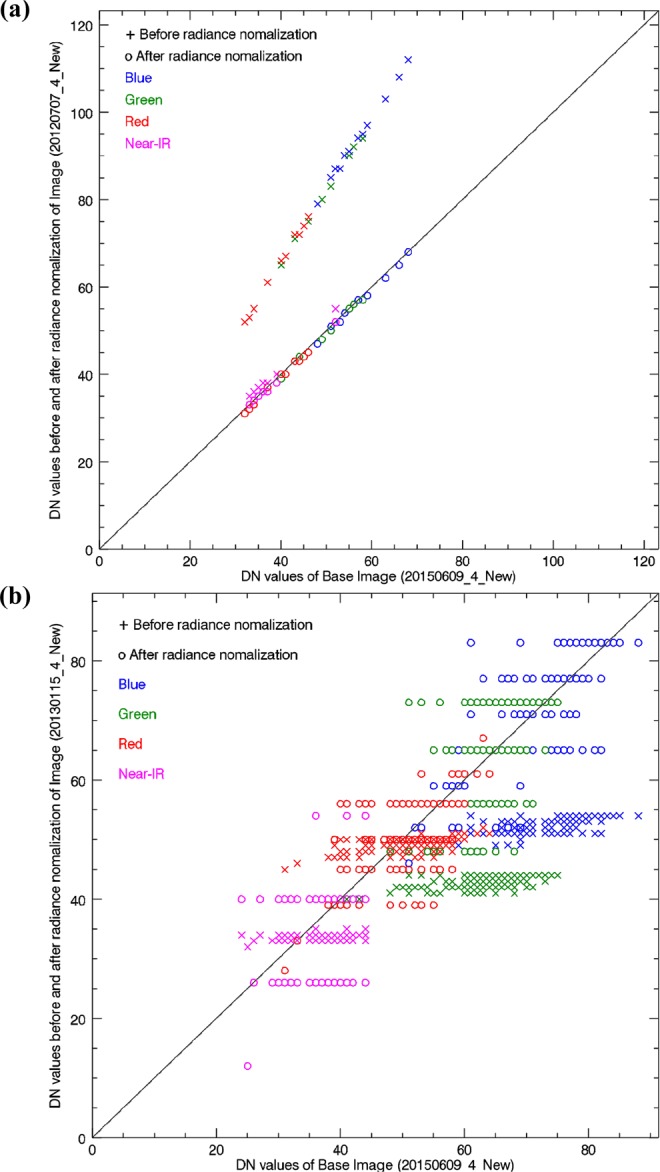
Illustration of the effect of radiance normalization for the images taken on (**a**) 7 July 2012 and (**b**) 15 January 2013, based on the image taken on 9 June 2015 (Fig. 2). The DN values of four spectral bands collected at all searched PIFs before and after the process of radiance normalization are denoted as cross marks and circle marks, respectively.

To illustrate how Eqs (6) and (7) are designed to enhance the contrast between shadow and non-shadow regions, the case of study site No. 385 (annotated as the green box in Fig. 2) is given in Fig. 4 as an example. The standard false color composite (Fig. 4a) clearly shows two large shadows on the upper-left and lower-right, as well as a considerable amount of shadows scattered throughout the entire area. By utilizing three properties of shadows: lower hue (closer to black, Fig. 4b), larger saturation (more scattering light in blue band, Fig. 4c), and lower intensity (blocked of light, Fig. 4d)^33^, the map of *SI* is calculated using Eqs (6) and (7) and presented in Fig. 4(e). Comparing to the map of *PC1* (Fig. 3f), *SI* indeed enhances the difference between shadow and non-shadow regions, which facilitates the determination of threshold to detect shadow.

**Figure 5:**
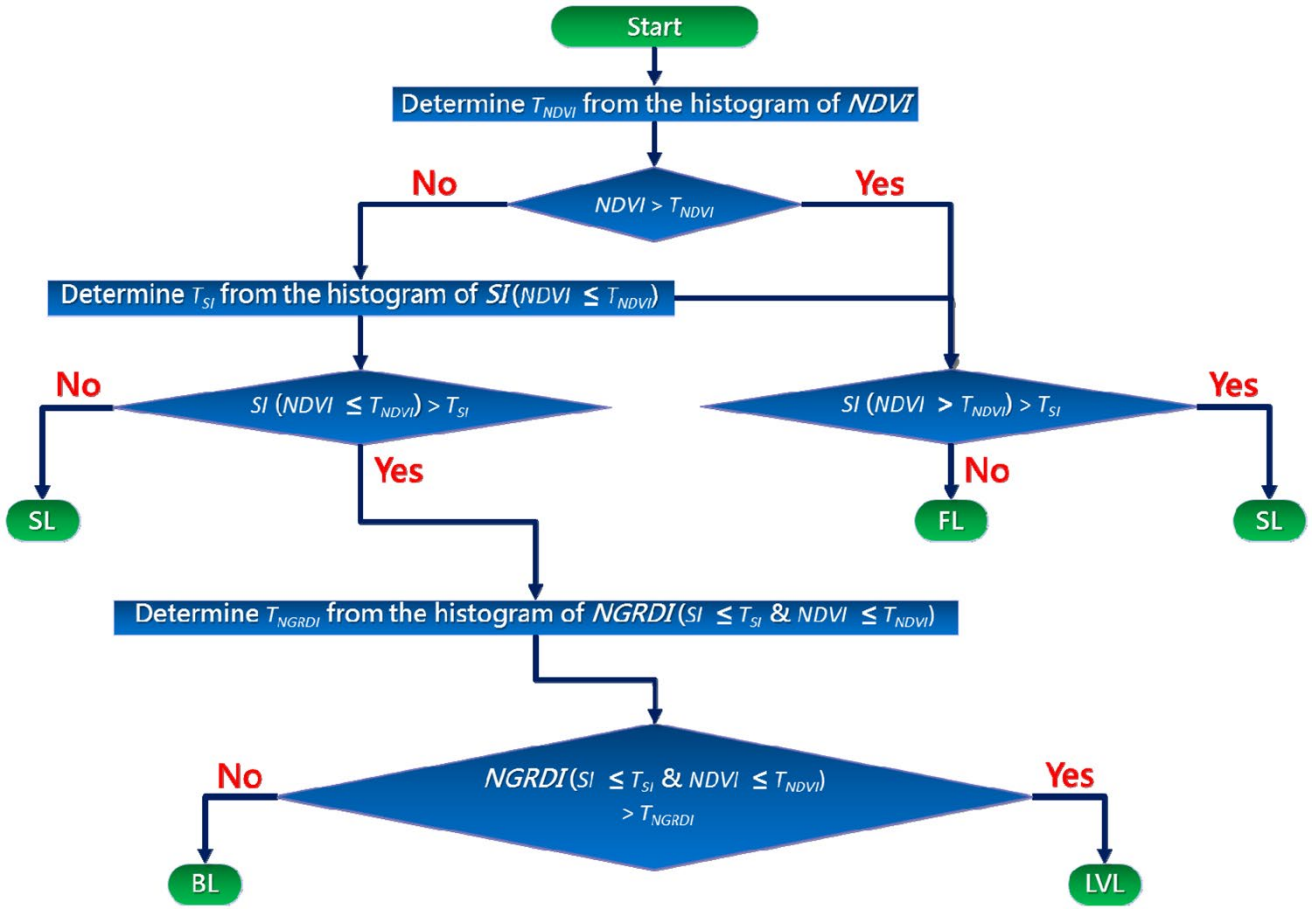
Flow chart of TLDT approach to map *FL*, *SL*, *BL* and *LVL* sequentially from one optical image by integrating *NDVI*, *SI* and *NGRDI*.

Considering the characteristic of each spectral index: *NDVI* is sensitive to vegetation, *SI* is sensitive to shadow, and *NGRDI* is sensitive to bare land, we adopt the concept of decision tree: building a classification model by breaking down a dataset into smaller and smaller subsets, and map *FL*, *SL* and *BL* sequentially from one optical image. The rest of the region can be regarded as *LVL*, under the assumption that there is no other land cover/land use in the study area. The flow chart of TLDT approach is shown in Fig. 5.

**Figure 6:**
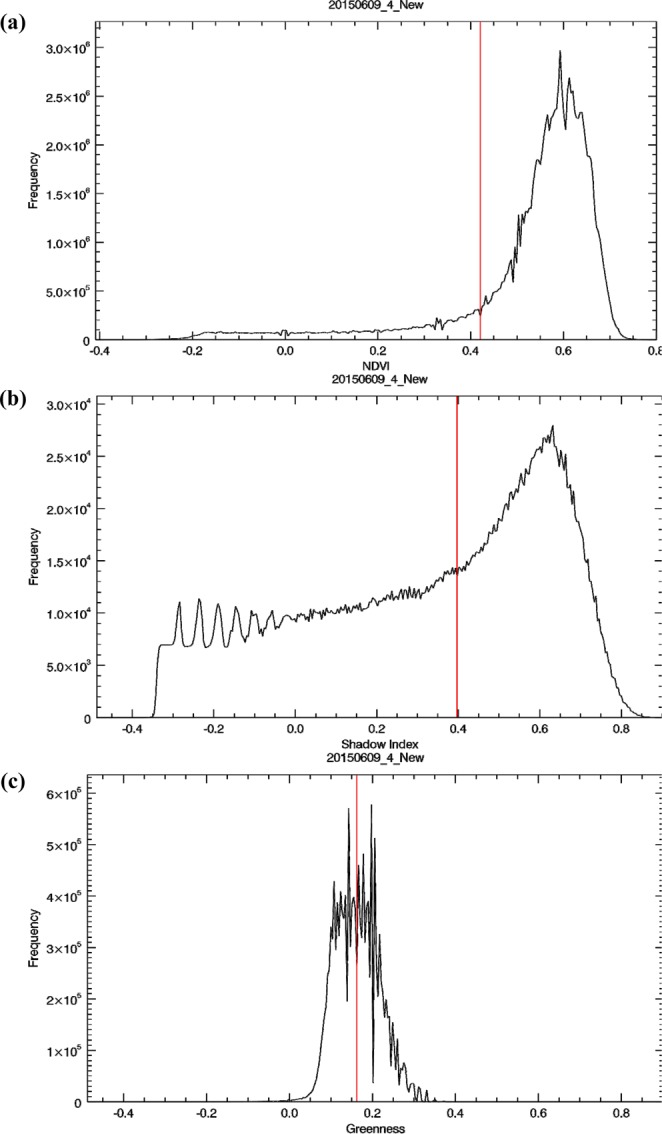
Histogram of (**a**) $NDVI$, (**b**) $SI$ and (**c**) *NGRDI*, calculated from Fig. 2. The thresholds *T*_*NDVI*_, *T*_*SI*_ and *T*_*NGRDI*_ are annotated as red lines in each plot.

**Figure 7:**
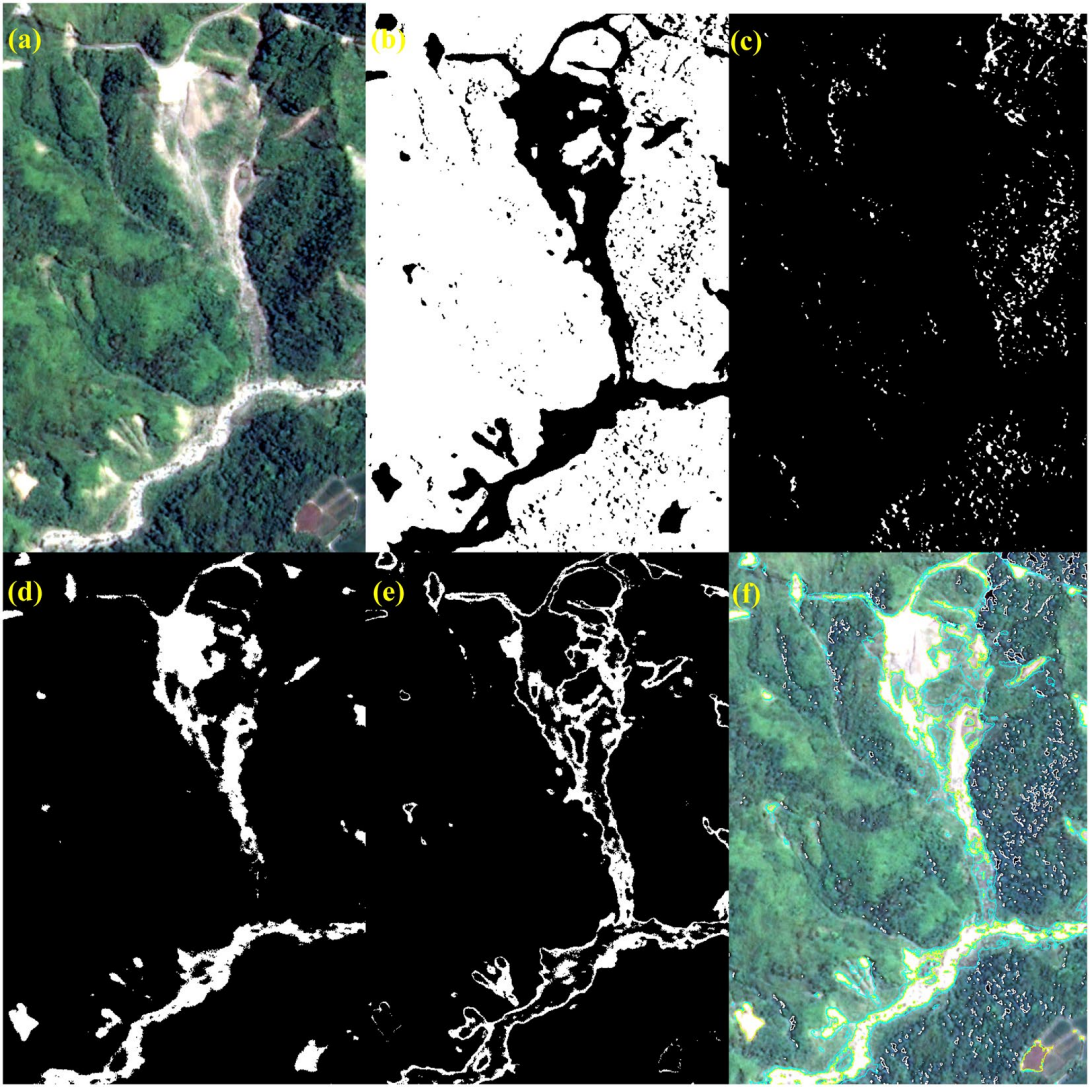
Results of classification for the case of site No. 378 (annotated as the red box in Fig. 2). (**a**) The true color composite, (**b**) *FL*, (**c**) *SL*, (**d**) *BL*, (e) *LVL* and (**f**) The true color composite overlaid with the boundaries of *SL* (white lines), *BL* (yellow lines) and *LVL* (cyan lines) (Formosat-2 image ©National Space Organization of Taiwan).

The case of site No. 378 (annotated as the red box in Fig. 2) is used as an example to illustrate the procedure of processing step-by-step, and the results are shown in Figs. 6 and 7, respectively. The histogram of *NDVI* exhibits a typical pattern of Gaussian distribution with single peak (Fig. 6a) since most of the study area is covered by forest (Fig. 7a). The threshold *T*_*NDVI*_ can be determined by the inflection point without ambiguity to separate the first subset: *FL* (Fig. 7b) from the non-vegetated area. The second subset: *SL* is another feature that is frequently found in an optical imagery of mountainous areas, because the sun is not usually in the nadir direction while the image is acquired^14^. Note that *SI* is designed to enhance the difference between the shaded and non-shaded areas. The threshold *T*_*SI*_ can also be determined by the inflection point in the histogram of *SI*, as long as there is a certain ratio of shadow in the non-vegetated area. This is exactly the case of Fig. 6(b), where a considerable part of topographic shadows can be seen in the study area (Fig. 7c). Under the assumption that there is no other land cover/land use in the study area, the rest of the region can be regarded as *BL* and *LVL*. Because the difference between the amount of *BL* and *LVL* is not as large as what we have seen for the cases of *FL* and *SL*, there is no such a typical pattern of Gaussian distribution with single peak in the histogram of *NGRDI* (Fig. 6c). Instead of selecting the inflection point, the Otsu’s method can be employed to determine the threshold *T*_*NGRDI*_^31^ and separate *BL* (Fig. 7d) from *LVL* (Fig. 7e). The Otsu’s method assumes that the image contains two classes and calculates the optimum threshold separating the two classes so that their inter-class variance is at the maximum. All boundaries of *FL* (green lines), *SL* (white lines), *BL* (yellow lines) and *LVL* (cyan lines) are overlaid on the true color composite and shown in Fig. 7(f) that gives a reasonable result by visual examination. To assess the effectiveness of forest restoration, this TLDT approach is applied to the time series of Formosat-2 images acquired at the same site. Then, the results are compared with the ones obtained independently from the high spatial resolution (25 cm) aerial orthophotos that were acquired approximately the same time in which the Formosat-2 images (8 m) were taken.

### Validation

**Figure 8:**
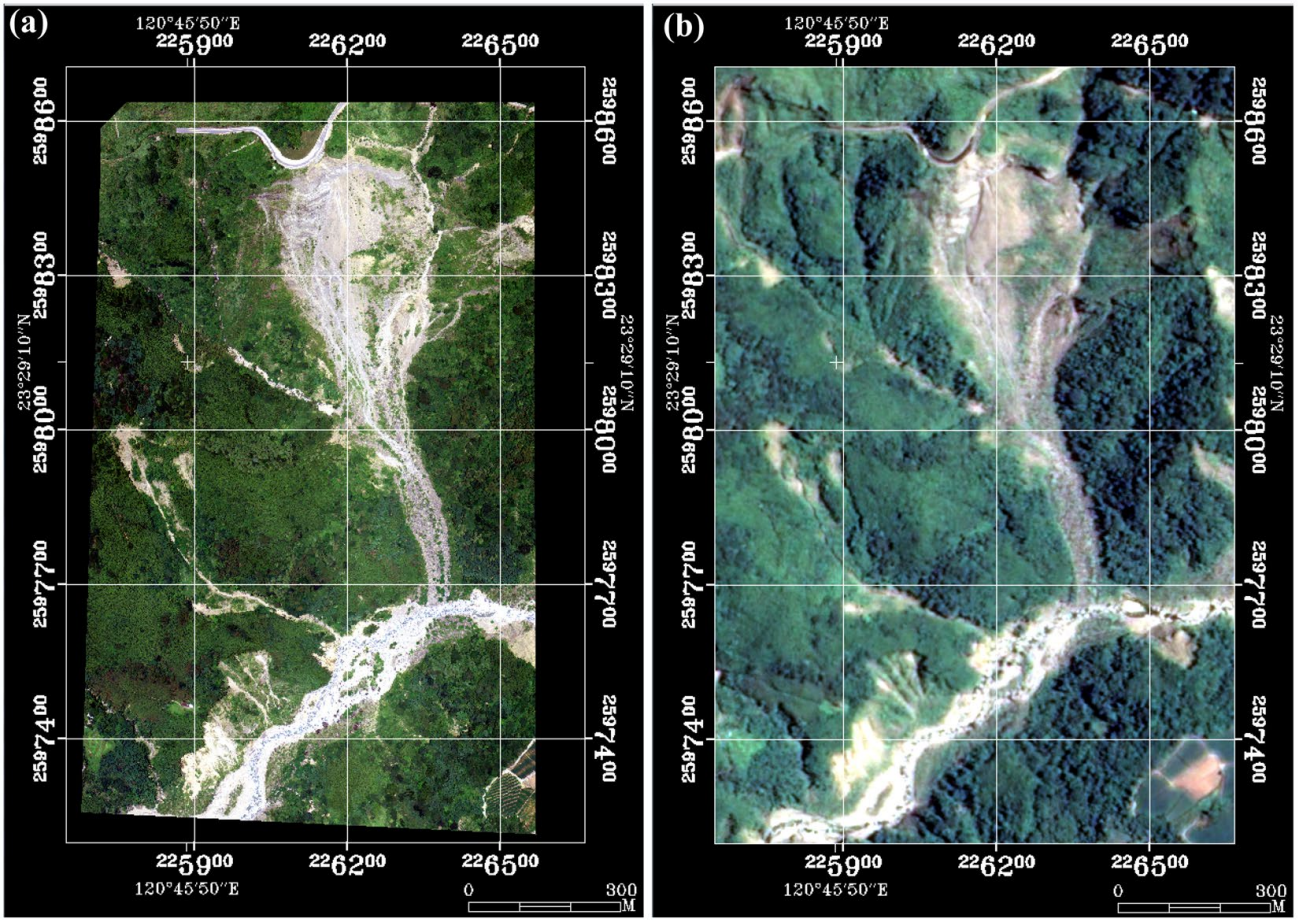
True color images of site No. 378 taken by (**a**) Airplane on 3 July 2012 (©Aerial Survey Office, Forestry Bureau of Taiwan), and (**b**) Formosat-2 on 7 July 2012 (©National Space Organization of Taiwan).

**Figure 9:**
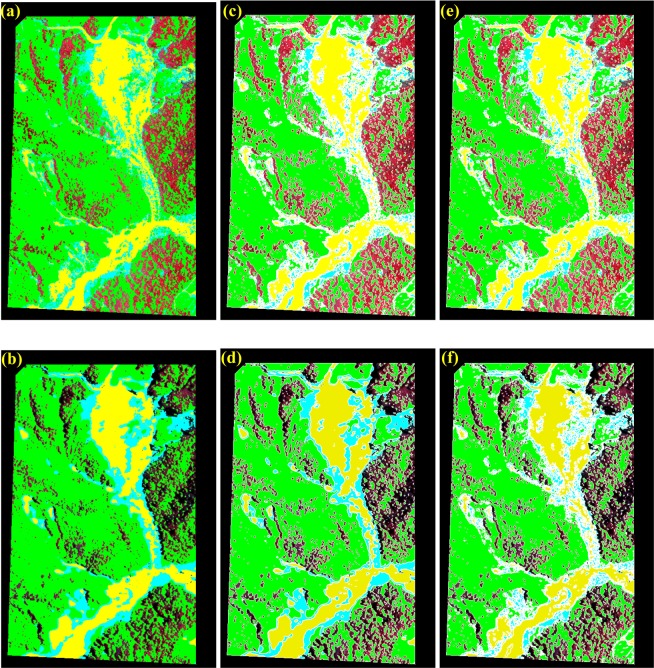
The regions of *FL* (green), *SL* (black), *BL* (yellow) and *LVL* (cyan) mapped from the images of site No. 378 taken by (**a**) Airplane on 3 July 2012 (the same as Fig. 8a, ©Aerial Survey Office, Forestry Bureau of Taiwan), and (**b**) Formosat-2 on 7 July 2012 (the same as Fig. 8b, ©National Space Organization of Taiwan). (**c**) the same as (**a**) but overlaid with the buffer zone (white regions). (**d**) the same as (**b**) but overlaid with the buffer zone (white regions). (**e**) the same as (**a**) but overlaid with the union of two buffer zones (white regions). (**f**) the same as (**b**) but overlaid with the union of two buffer zones (white regions).

To validate the results of *FL*, *SL*, *BL* and *LVL* mapped from the TLDT approach, the aerial orthophoto of site No. 378 taken on 3 July 2012 is used as an alternative source of ground truth, shown in Fig. 8(a). The same region is cut from the Formosat-2 image taken on 7 July 2012, shown in Fig. 8(b) for comparison. The results of classification of *FL*, *SL*, *BL* and *LVL* are shown in Fig. 9(a,b) and the confusion matrix is given in Table 4. The same assumption is made that the region of *LVL* is regarded as the rest from those of *FL*, *SL* and *BL*. Although the overall accuracy is as high as 86.4%, the commission error and omission error for *LVL* are both low (42.8% and 50.6%). After carefully examining Fig. 9(a,b), we confirm that the deviations are mainly found near the boundary of each class. Because the spatial resolutions of two images are intrinsically different (aerial orthophoto: 25 cm; multispectral bands of Formosat-2 image: 8 m), it is expectable that they won’t give the same details near the boundary of each class. To exclude the deviations caused by difference resolution, the buffer zone with a size of one pixel is taken at the boundary of each class, as shown as the white regions in Fig. 9(c,d). The union of both buffer zones is then generated and applied to both images, in order to mask out those suspicious pixels near the boundary of each class, as shown as the white regions in Fig. 9(e,f). The confusion matrix is calculated again and the result is shown in Table 5. Not only the overall accuracy increases to 96.8%, but the commission error and omission error for *LVL* are both improved to 23.0% and 35.8%. This provides an alternative way to validate the TLDT approach.

**Table 4:** Confusion matrix of validating the results of *FL*, *BL* and *LVL* mapped from the TLDT approach.

	*FL*(pixel)^†^	*LVL*(pixel)^†^	*BL*(pixel)^†^	Commission error (%)
*FL*(pixel)^‡^	155,191	6,990	1,043	4.9%
*LVL*(pixel)^‡^	8,810	17,367	8,991	50.6%
*BL*(pixel)^‡^	1,189	5,988	37,782	16.0%
Omission error (%)	6.1%	42.8%	21.0%	Overall accuracy = 86.4%

^†^The aerial orthophoto of site No. 378 was taken on 3 July 2012, as shown in Fig. 8(a). ^‡^Formosat-2 image was taken on 7 July 2012, as shown in Fig. 8(b).

**Table 5:** Confusion matrix of validating the results of *FL*, *BL* and *LVL* mapped from the TLDT approach, after excluding the deviations near the boundary of each class caused by the different resolutions between Formosat-2 imagery and Aerial orthophoto.

	*FL*(pixel)^†^	*LVL*(pixel)^†^	*BL*(pixel)^†^	Commission error (%)
*FL*(pixel)^‡^	120,267	971	50	0.8%
*LVL*(pixel)^‡^	1,479	5,872	1,798	35.8%
*BL*(pixel)^‡^	80	786	30,032	2.8%
Omission error (%)	1.3%	23.0%	5.8%	Overall accuracy = 96.8%

^†^The aerial orthophoto of site No. 378 was taken on 3 July 2012, as shown in Fig. 8(a). ^‡^Formosat-2 image was taken on 7 July 2012, as shown in Fig. 8(b).

### Quantitative assessment of forest restoration

**Figure 10:**
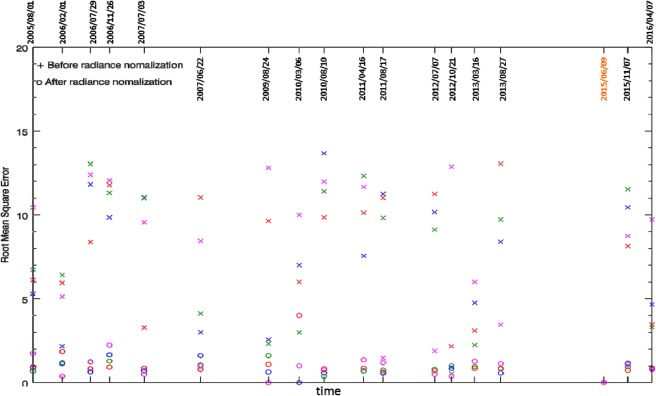
Root mean square errors of the time series of Formosat-2 image before (cross marks) and after (circle marks) the radiance normalization.

For a successful case of forest restoration, we expect to see a gradual decrease of *BL* and a gradual increase of *FL*. Before performing the time series analysis, the same procedure of radiance normalization as described in section 3.2 is applied to the entire time series of Formosat-2 imagery to ensure that the classification of *FL*, *SL*, *BL* and *LVL* are comparable on various dates. For site No. 378 (located in Zone 2), the root mean square errors (RMSD) of each spectral band before (cross marks) and after (circle marks) the radiance normalization are shown in Fig. 10, using the one taken on 9 June 2015 as the base image of radiance normalization. Results show that RMSD is reduced significantly by the process of radiance normalization. For example, the RMSD of blue band of the image taken on 10 August 2010 is reduced from the highest value of 13.6 to less than 1.0. The radiance-normalized images, are employed for classification of *FL*, *SL*, *BL* and *LVL*. Based on the classified *FL*, *SL*, *BL* and *LVL*, the area ratio *A*_*X*_ is defined as

8 $${A}_{X}\equiv \frac{X}{FL+SL+BL+LVL},$$

where *X* represents the class of *FL*, *SL*, *BL* and *LVL*. Considering the fact that *A*_*SL*_ is different in different images acquired in different seasons, we can use the shadow area ratio

9 $$SAR\equiv \frac{{A}_{SL}}{{A}_{FL}+{A}_{BL}+{A}_{LVL}},$$

to correct the influence of shadow on class *X* and calculate its corrected area ratio by

10 $${A}_{X}^{\ast }\equiv {A}_{X}\times \mathrm{(1}+SAR),$$

and $${A}_{X}^{\ast }$$ satisfies

11 $${A}_{FL}^{\ast }+{A}_{BL}^{\ast }+{A}_{LVL}^{\ast }={A}_{FL}+{A}_{SL}+{A}_{BL}+{A}_{LVL}.$$

**Figure 11:**
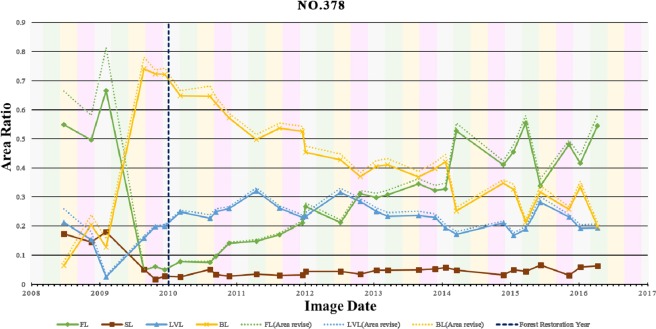
Time series of *A*_*FL*_, *A*_*SL*_, *A*_*BL*_, *A*_*LVL*_, ${A}_{FL}^{\ast }$, ${A}_{BL}^{\ast }$ and ${A}_{LVL}^{\ast }$ at site No. 378.

Tracing the variation of ${A}_{FL}^{\ast }$, ${A}_{BL}^{\ast }$ and ${A}_{LVL}^{\ast }$ with time provides a good indicator of restoration. Figure 11 shows the time series of *A*_*FL*_, *A*_*SL*_, *A*_*BL*_, *A*_*LVL*_, ${A}_{FL}^{\ast }$, ${A}_{BL}^{\ast }$ and ${A}_{LVL}^{\ast }$ at site No. 378. Though *A*_*SL*_ indeed varies with time, it occupies about merely 5% of the study area and results in a small value of *SAR* (Eq. 9). The influence of shadow is not that significant, and thus *A*_*X*_ (solid lines) are very close to ${A}_{X}^{\ast }$ (dotted lines) using Eq. (10), where *X* represents the class of *FL*, *BL* and *LVL*. Both the time series of ${A}_{FL}^{\ast }$ and ${A}_{BL}^{\ast }$ indicate that the landslide was triggered by Typhoon Morakot in early August of 2009 when the largest *A*_*BL*_ is found. Ever since the restoration was implemented in early 2010, ${A}_{BL}^{\ast }$ (orange dotted line) has been decreased and ${A}_{FL}^{\ast }$ (green dotted line) has been increased gradually. Meanwhile, ${A}_{LVL}^{\ast }$ (blue dotted line) has maintained at an approximately constant level that was slightly higher in the early stage and slightly lower in the later stage of restoration. This sheds some light on the process of forest restoration: ${A}_{FL}^{\ast }$ is only one-third to a half of ${A}_{LVL}^{\ast }$ in the beginning of restoration; ${A}_{FL}^{\ast }$ keeps increasing while ${A}_{LVL}^{\ast }$ stays at the same level; and eventually, ${A}_{FL}^{\ast }$ could reach double or even triple of ${A}_{LVL}^{\ast }$ in the later stage of restoration. Figure 11 also reveals the seasonal variations of ${A}_{FL}^{\ast }$, ${A}_{BL}^{\ast }$ and ${A}_{LVL}^{\ast }$ by annotating four seasons with four difference background colors. Generally speaking, the seasonal variations of ${A}_{FL}^{\ast }$ are more apparent before Typhoon Morakot than in the later stage of restoration. Because the seasonal variations of ${A}_{FL}^{\ast }$ is one of the methods of representing the function of plant metabolism, ${A}_{FL}^{\ast }$ can be regarded as another indicator of forest restoration. In any case, the long-term trend of successful forest restoration at site No. 378 is clear, shown by the comparison of the annual data of the same season and the regression lines in Fig. 11.

## Results and Discussion

**Figure 12:**
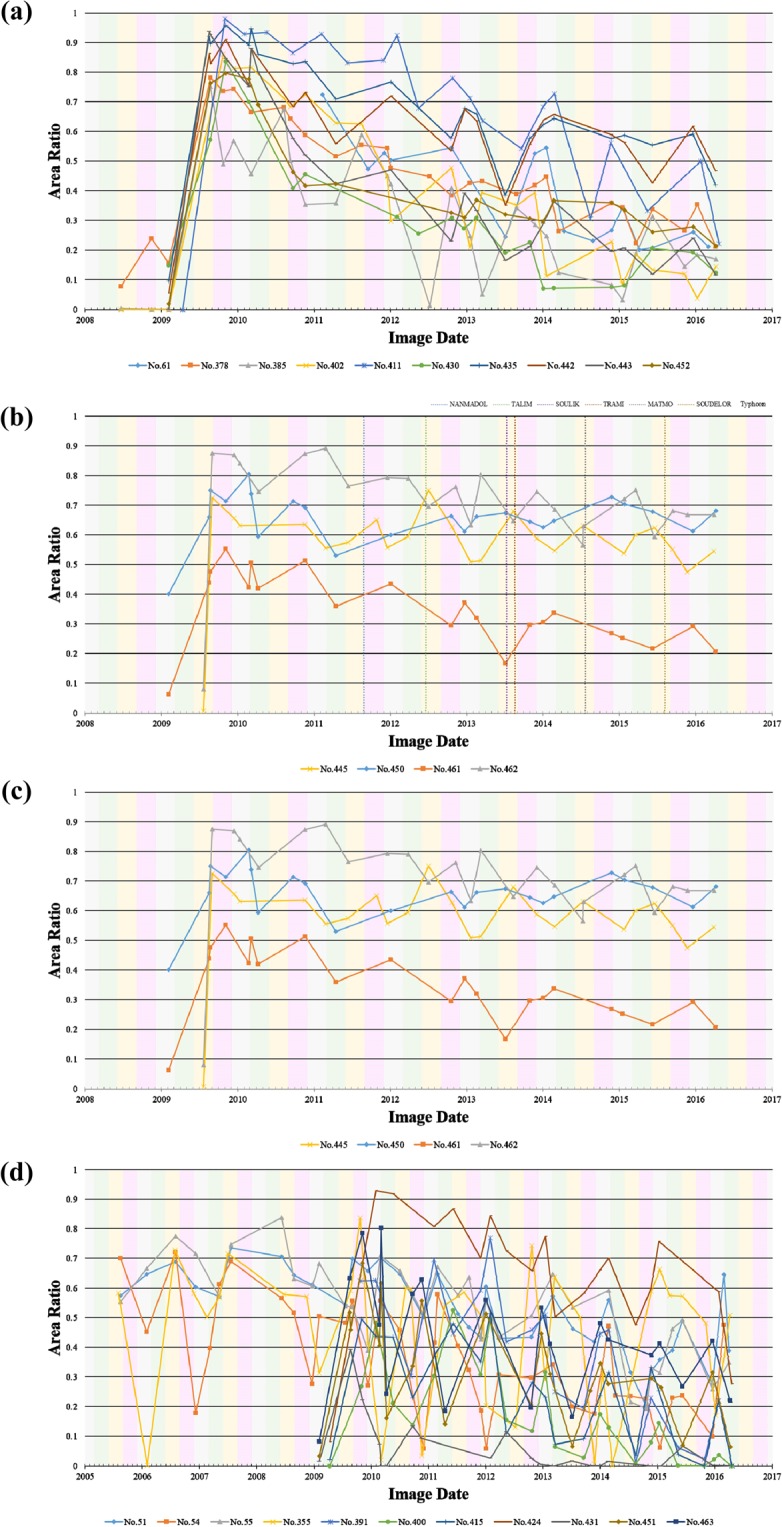
Quantitative assessment of forest restoration at all 30 study sites based on the time series of ${A}_{BL}^{\ast }$. (**a**) restored well apparently; (**b**) restored in a slow pace; (**c**) restored ineffectively; (**d**) no assessment.

Following the same procedure described in section 3.3, the results of *FL*, *SL*, *BL* and *LVL* mapped from the TLDT approach at the other 14 test sites are compared to the results derived from the aerial orthophotos. The overall accuracy for each test site is listed in Table 1. As explained and emphasized in section 3.3, these values do not serve as an accuracy report, because the spatial resolutions of source images are intrinsically different. Despite those deviations found near the boundary of each class, the overall consistency of classification for all 14 test sites demonstrates that the TLDT approach is valid. The same approach is employed to assess the effectiveness of forest restoration for all 30 study sites, and the time series of ${A}_{BL}^{\ast }$ are grouped, shown in Fig. 12 for comparison. The results of effectiveness assessment are also listed in Table 1, which indicate that the forests are restored efficiently at ten sites (No. 61, 378, 385, 402, 411, 430, 435, 442, 443 and 452) (Fig. 12a), but restored in a slow pace at four sites No. 440, 459, 460 and 482 (Fig. 12b). The other four sites, No. 445, 450, 461 and 462 show that the landslide areas have been stabilized or even expanded and there is no significant change of forests. Therefore, the forest restoration is ineffective (Fig. 12c). The rest of the 12 sites (No. 51, 54, 55, 355, 391, 400, 415, 424, 431, 451, 454 and 463) are all located in the shaded sides of mountain areas, where a considerable fraction of Formosat-2 image are severely covered by shadows (Fig. 12d). Consequently, no assessment of the effectiveness of forest restoration can be drawn from the time series of Formosat-2 images.

The most popular and widely-used engineering method of forest restoration in Taiwan is seeds spreading. In this research, a total of 27 sites adopted this method, though a few of them also employed other methods such as staking and wattling, drainage of longitudinal and transverse, tree planting, and covering the grass net. After excluding those 10 sites with no assessment due to shadows, 9 sites (52.9%) are restored well apparently, 4 sites (23.5%) are restored in a slow pace, and 4 sites (23.5%) are ineffective. In other words, the successful rate of forest restoration in Taiwan is 76.5%. The reason of unsuccessful restoration is also clarified by denoting the dates of serious earthquake, typhoon, and storm events in Fig. 12(c). The secondary or even tertiary disasters destroyed the vulnerable newly-restored land. As a result, all earlier efforts of restoration dissipated and *BL* was not converted to *FL*. Such a failure has nothing to do with the engineering method of seeds spreading. Nevertheless, a timely response to the secondary disaster can mitigate the damage. This can be achieved by acquiring the aftermath remote sensing imagery, analyzing with the approach proposed in this research, and comparing with the time series of observations at the same site. Because almost all study sites adopted the similar method of seeds spreading, there is no intention to specify how or whether the restoration technique affected recovery in this work. It is better not to include many types of restoration technique at the stage of developing and evaluating a new approach. Future works have been planned to employ this new approach to investigate how different restoration techniques affect recovery.

**Figure 13:**
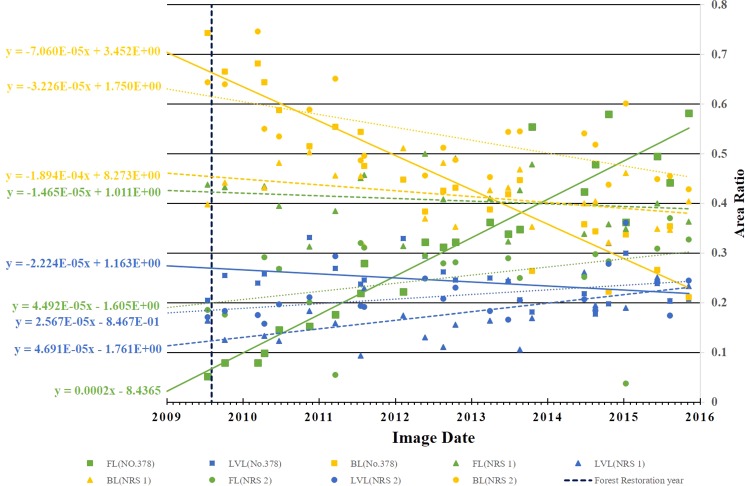
Comparison of the time series of ${A}_{FL}^{\ast }$ (green), ${A}_{LVL}^{\ast }$ (blue) and ${A}_{BL}^{\ast }$ (yellow) and their corresponding trends at site No. 378 (square marks, solid lines), NRS 1 (triangle marks, dashed lines), and NRS 2 (circle marks, dotted lines). The corresponding linear regression equations are listed in Table 6.

The distinction between natural regeneration and regrowth enhanced by restoration efforts can be made by using the existing dataset and method developed in this research. As shown in Fig. 2, the landslides at NRS 1 and 2 were also triggered by Typhoon Morakot but they were not selected as restoration sites by FBT. Therefore, the changes found at NRS 1 and 2 are purely the effect of natural regeneration, which can help us to gain insight into the difference between natural regeneration and restoration. The time series of ${A}_{FL}^{\ast }$, ${A}_{LVL}^{\ast }$ and ${A}_{BL}^{\ast }$ at sites No. 378, NRS 1 and NRS 2 are plotted in Fig. 13 for comparison, and their corresponding linear regression equations are listed in Table 6. Generally speaking, both ${A}_{FL}^{\ast }$ and ${A}_{BL}^{\ast }$ keep the same values and ${A}_{LVL}^{\ast }$ increases slightly at NRS 1 (triangle marks) throughout time, except for some seasonal variations. A slight trend of natural regeneration is shown at NRS 2 (circle marks): ${A}_{FL}^{\ast }$ (0.19–0.33), ${A}_{LVL}^{\ast }$ (0.17–0.24) and ${A}_{BL}^{\ast }$ (0.64–0.42); while a significant amount of restoration is attained at No. 378: ${A}_{FL}^{\ast }$ (0.05–0.58), ${A}_{LVL}^{\ast }$ (0.21–0.21) and ${A}_{BL}^{\ast }$ (0.74–0.21). This comparison also highlights the importance of restoration for it accelerates the natural regeneration to at least four times, in terms of the slopes of the linear regression equation of ${A}_{FL}^{\ast }$ listed in Table 6 (2.0 × 10^−4^ vs. 4.492 × 10^−5^).

**Table 6:** The corresponding linear regression equations of the time series of ${A}_{FL}^{\ast }$, ${A}_{LVL}^{\ast }$ and ${A}_{BL}^{\ast }$ at sites No. 378, NRS 1 and NRS 2, as shown in Fig. 13.

	No. 378	NRS 1	NRS 2
${A}_{FL}^{\ast }$	$y=2.0\times {10}^{-4}x-8.4365$	$y=-1.465\times {10}^{-5}x+1.011$	$y=4.492\times {10}^{-5}x-1.605$
${A}_{LVL}^{\ast }$	$y=-2.224\times {10}^{-5}x+1.163$	$y=4.691\times {10}^{-5}x-1.761$	$y=2.567\times {10}^{-5}x-0.8467$
${A}_{BL}^{\ast }$	$y=-7.06\times {10}^{-5}x+3.452$	$y=-1.894\times {10}^{-4}x+8.273$	$y=-3.326\times {10}^{-5}x+1.75$

Considering the scale of landslides in Taiwan’s mountain areas, the high spatial resolution satellite imagery or aerial orthophotos are preferable sources of data in the management of forest watershed. For example, the preparation of a long-term national landslide inventory is based on 2 m resolution Formosat-2 imagery^30^, while the events responding to landslides and debris flow are based on tens of centimeter resolution aerial orthophotos acquired from a low-cost unmanned aerial vehicle^34^. Though these optical imagery with multispectral bands (e.g. red, green, blue and near-infrared) have high spatial resolution, they lack the critical spectral bands to retrieve the amount of water vapor and aerosol. Consequently, it is very unlikely to conduct a rigorous atmospheric correction interference before conducting the time series analysis. This work demonstrates an alternative method, confirming that the relative calibration based on the technique of radiance normalization enables us to map *FL*, *SL*, *BL* and *LVL* using the TLDT approach and meets the requirement of time series analysis. The process of radiance normalization also provides an appropriate way of examining the quality of every image.

Geographical locations of forest restoration sites in Taiwan are all located in mountainous areas, where *SL* is one of the main features that are inevitably found in an optical imagery^14^. In this research, 12 of 30 sites (40%) are severely covered by shadows and no assessment of the effectiveness of forest restoration can be drawn from the time series of Formosat-2 images. Some shadows are casted by topographic relief and may not always be completely dark. They might be illuminated by scattering light and can be recovered by considering the topographic relief after rigorous atmospheric correction. Thanks to the success of Sentinel-2 mission^35^, high-temporal (5 days), -spatial (10 m), -spectral (13 bands), and -radiometric (12 bits), data are now available systematically and freely to all registered users. European Space Agency also developed and released an official tool, Sen2Cor, which is able to perform a rigorous atmospheric correction and produce the Bottom-Of-Atmosphere reflectance images^36^. Together with the usage of 90 m SRTM Digital Elevation Database, Sen2Cor is able to conduct terrain correction for rugged terrains. In light of the successful launch/operation of Sentinel-2A/B and the following new identical missions continuing to take the data record to the 2030 time frame^37^, future research is being proposed to investigate the feasibility of employing Sentinel-2 multitemporal imagery to assess the restoration of forest based on the experience acquired and technique developed in this work.

## Conclusion

Climate variability and man-made impacts have severely damaged the forest around the world in recent years, which calls for an urgent need of restoration aiming toward long-term sustainability for the forest environment. To assess the effectiveness of forest restoration requires a systematic and synoptic view for monitoring the forest. However, this cannot be achieved by the traditional method of *in situ* site surveying, particularly for those inaccessible sites with large-scale restoration. Remote sensing imagery by far is more advantageous in assessing forest restoration, mainly due to its ability to detect changes in large areas over long periods of time that are difficult to observe from the ground. Considering the cost, restoration scale and data availability in Taiwan, the multitemporal, multispectral and high-spatial-resolution Formosat-2 imagery acquired between 2005 and 2016 are employed to assess the effectiveness of forest restoration at 30 sites. However, Formosat-2 imagery cannot go through a rigorous atmospheric correction due to the lack of critical spectral bands to retrieve the amount of water vapour and aerosol. This paper proposes a new TLDT approach to map *FL*, *SL*, *BL* and *LVL* sequentially from the remote sensing imagery by integrating three spectral indices: *NDVI*, *SI* and *NGRDI* TLDT requires neither image normalization nor atmospheric correction, and improves on the other methods by introducing more levels of decision tree classification with inputs from the same multispectral imagery. This paper also demonstrates that the relative calibration based on the technique of radiance normalization meets the requirement of time series analysis of Formosat-2 imagery. The process of radiance normalization provides an appropriate way to examine the quality of every image. With TLDT, the effectiveness of forest restoration at 30 sites are assessed, using all available multispectral Formosat-2 images acquired between 2005 and 2016. The assessments at 15 test sites are compared with the results obtained independently from the high-spatial-resolution (25 cm) aerial orthophotos that were acquired approximately the same time in which the Formosat-2 images (8 m) were taken. Results show that the overall consistency is between 69.14% and 87.51%, while the kappa values are between 0.46 and 0.76. These comparisons are not intended to conclude the assessment accuracy but to clarify why and where the discrepancy is, since the spatial resolutions are rather different between the aerial orthophoto (25 cm) and Formosat-2 image (8 m). Among the 30 study sites, 10 have restored successfully, 4 are recovering slowly, and 4 have hardly re-vegetated. For the rest of the 12 sites, the shaded areas are too large to derive a detailed trend of restoration. But the effectiveness can still be assured by examining the pre-restoration and the most up-to-date Formosat-2 images. The distinction between natural regeneration and regrowth enhanced by restoration efforts were also made by using the existing dataset and TLDT developed in this research. This work supports the use of multitemporal remote sensing imagery as a reliable source of data for assessing the effectiveness of forest restoration on a regular basis. The global trend of forest restoration can be obtained by applying the same approach to Landsat dataset, the longest archive of space-based moderate-resolution land remote sensing data. Therefore, this work also serves as the basis for studying the global trend of forest restoration in the future.

## Supplementary information

LaTeX Supplementary File

Supplementary Information
