# Supplementary figures and images for: Assessment of forest restoration with multitemporal remote sensing imagery
Source: Sci Rep. 2019 May 13;9:7279. doi: 10.1038/s41598-019-43544-5 (PMC6513895; doi:10.1038/s41598-019-43544-5)

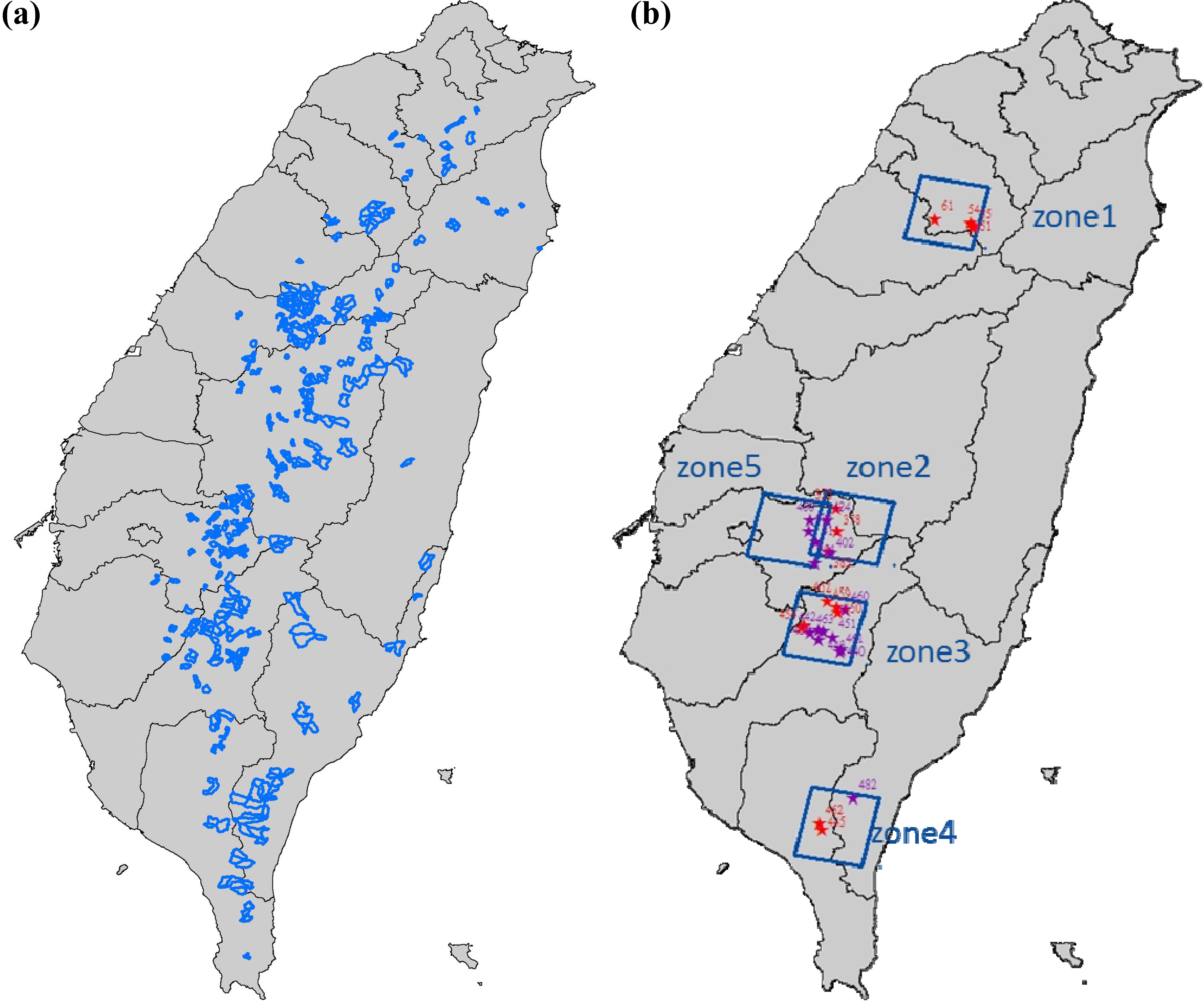

Supplement: Supplementary file 1 — LaTeX Supplementary File [file 41598_2019_43544_MOESM1_ESM.zip › Fig1.jpg]

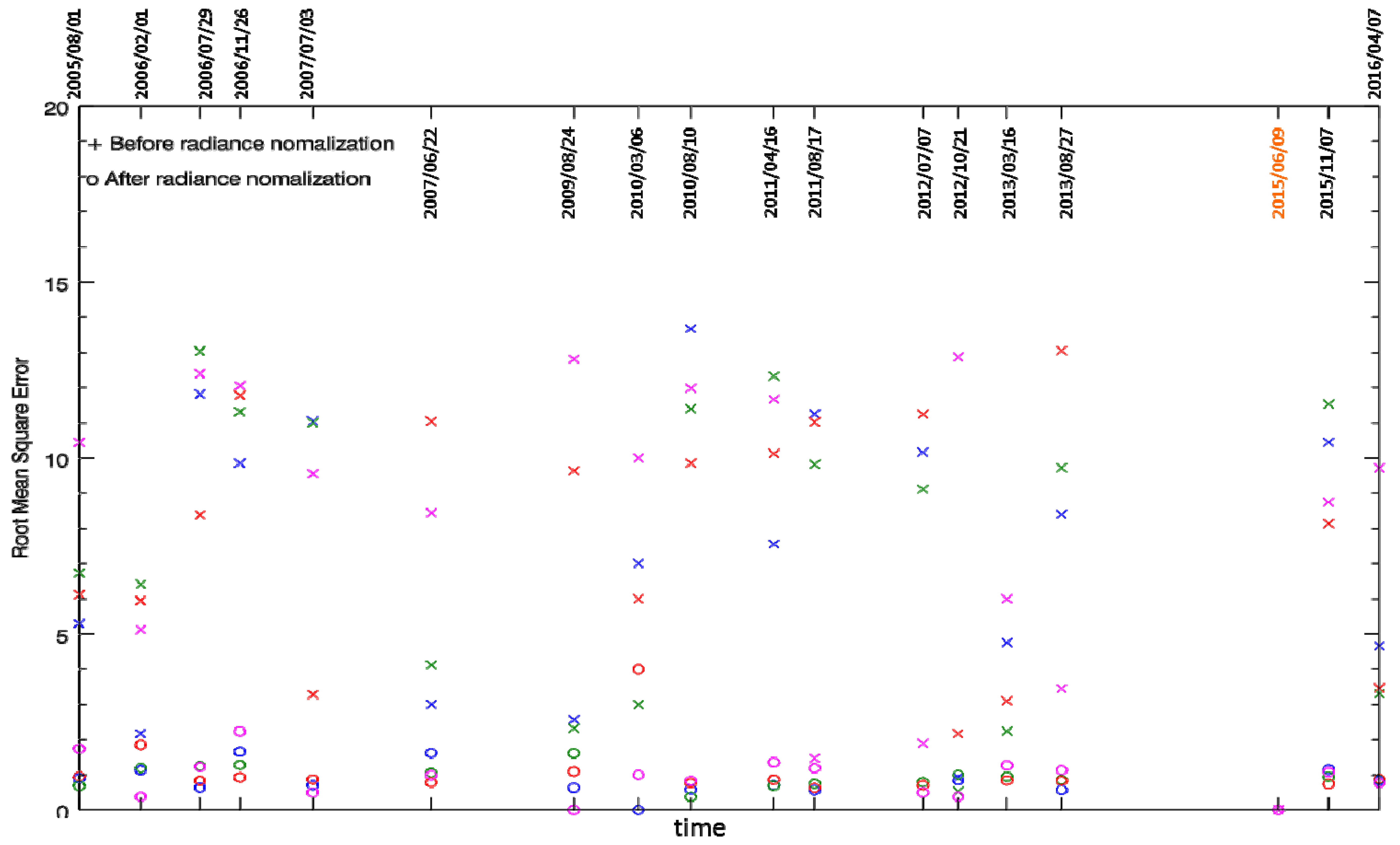

Supplement: Supplementary file 1 — LaTeX Supplementary File [file 41598_2019_43544_MOESM1_ESM.zip › Fig10.jpg]

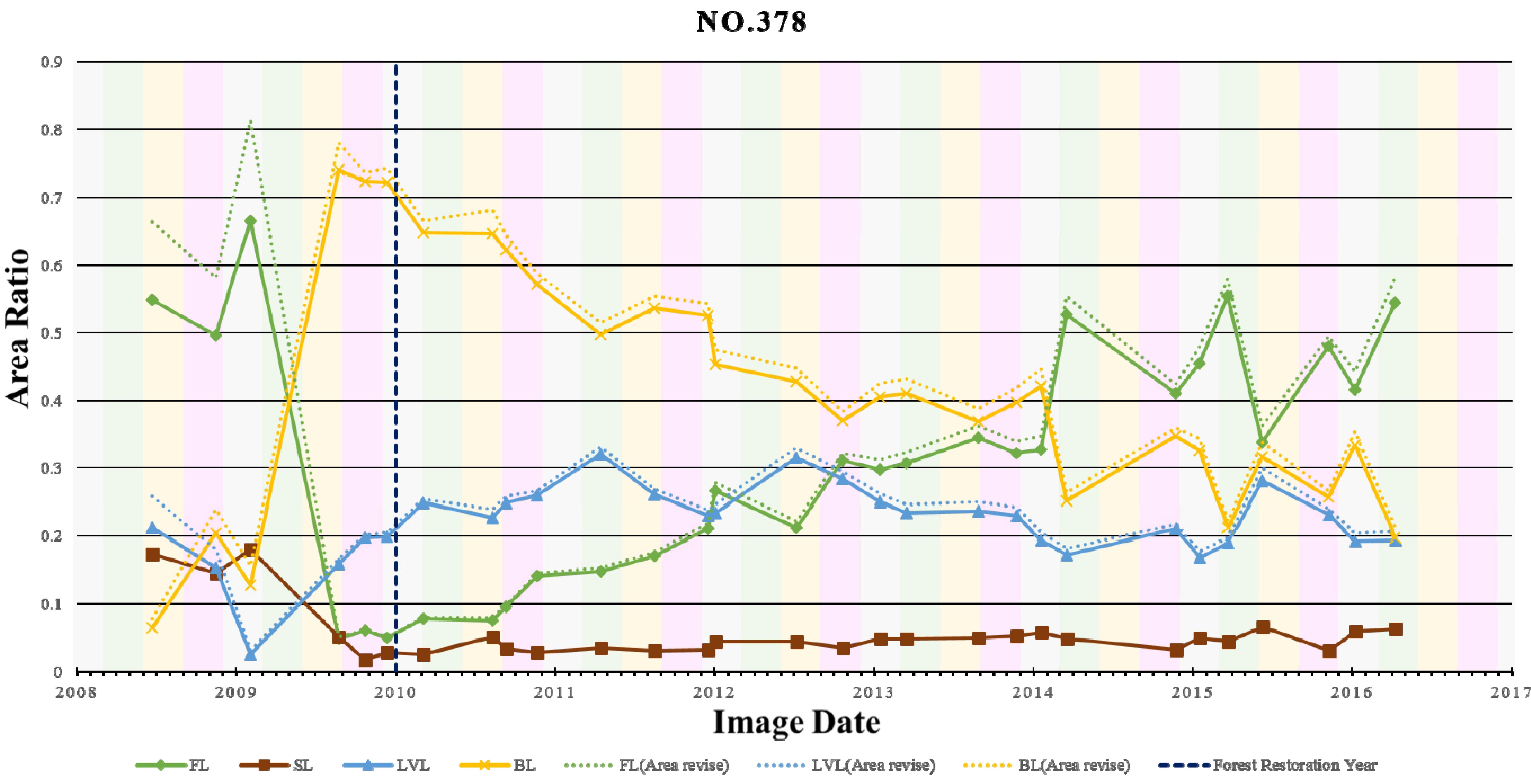

Supplement: Supplementary file 1 — LaTeX Supplementary File [file 41598_2019_43544_MOESM1_ESM.zip › Fig11.jpg]

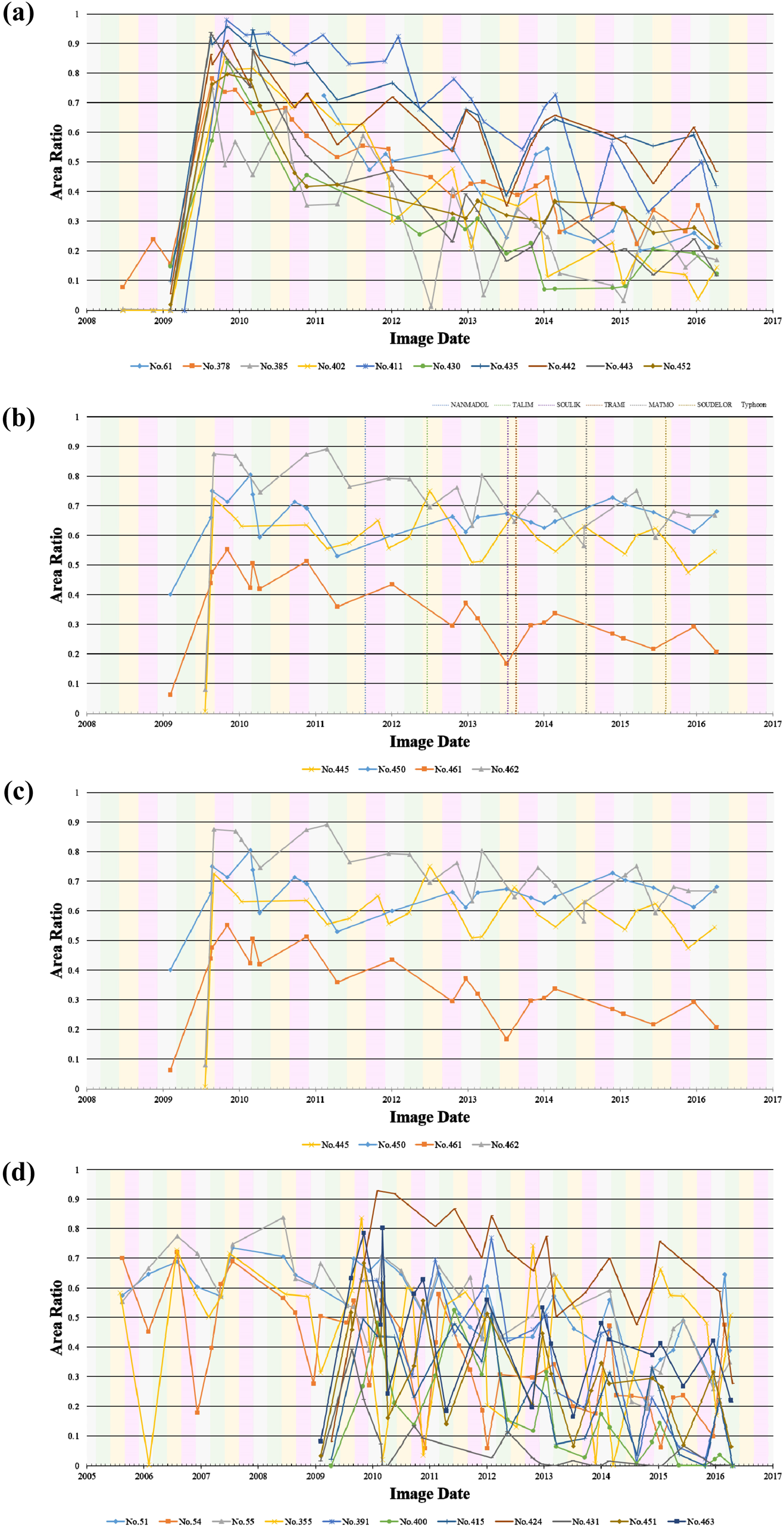

Supplement: Supplementary file 1 — LaTeX Supplementary File [file 41598_2019_43544_MOESM1_ESM.zip › Fig12.jpg]

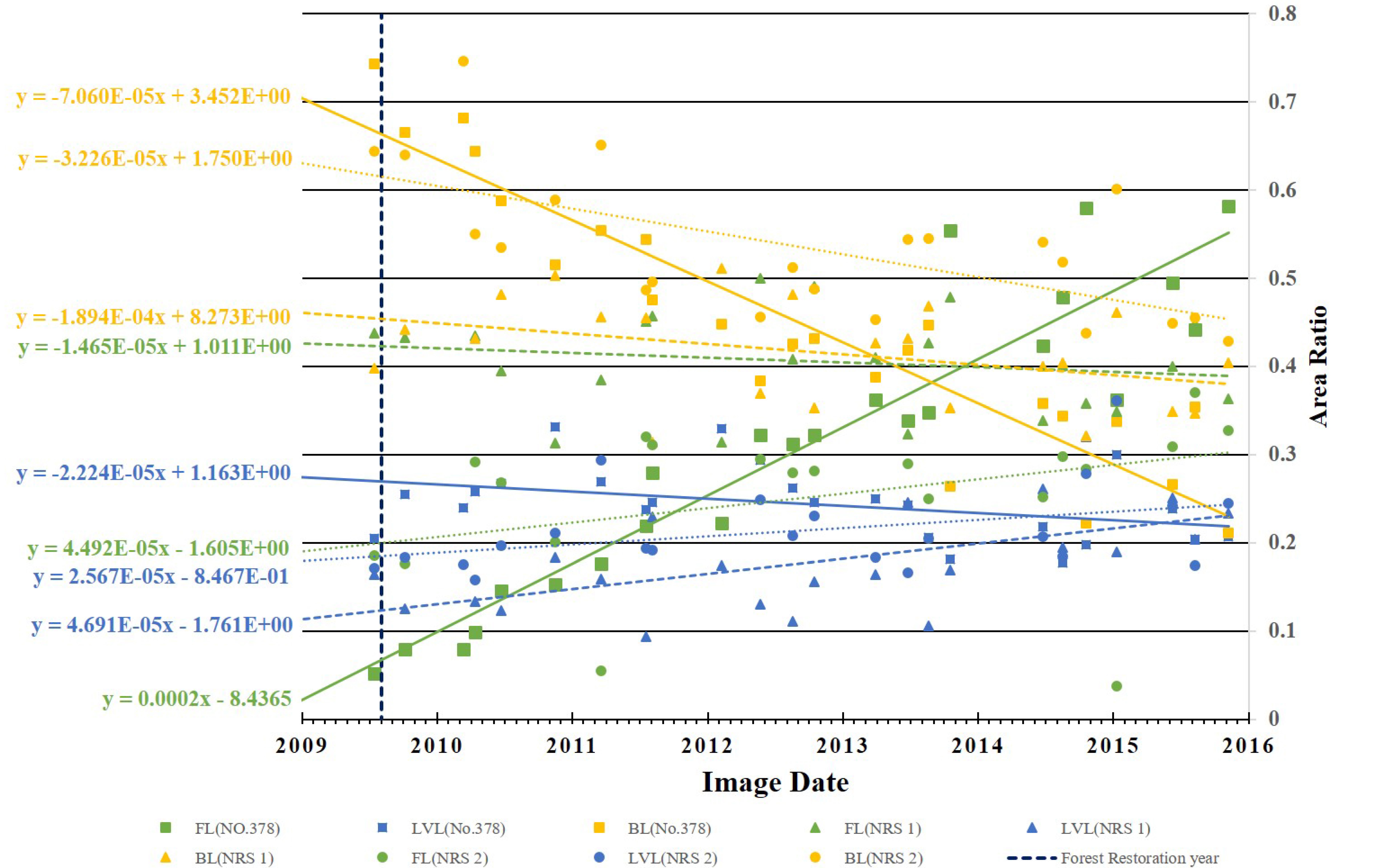

Supplement: Supplementary file 1 — LaTeX Supplementary File [file 41598_2019_43544_MOESM1_ESM.zip › Fig13.jpg]

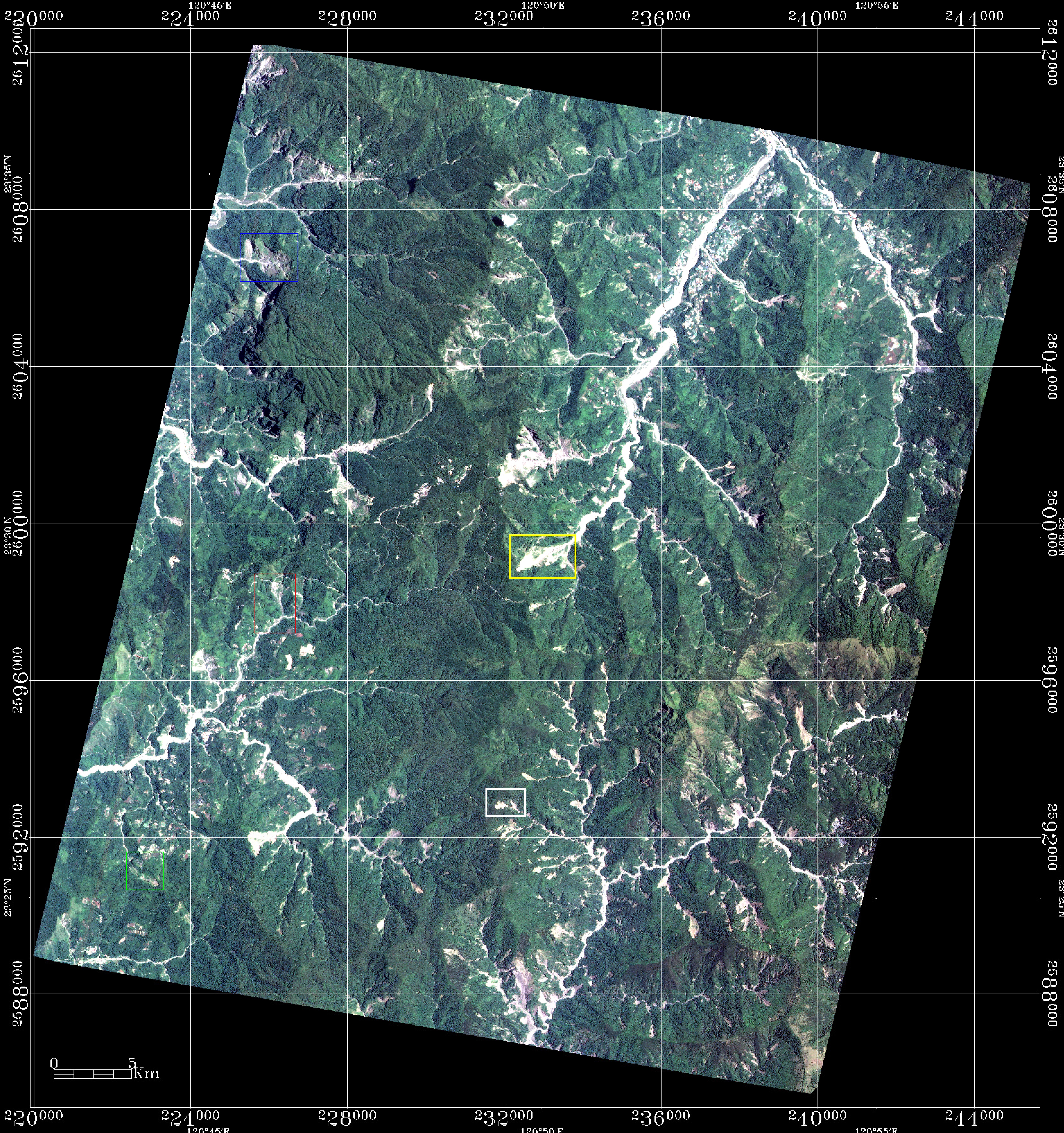

Supplement: Supplementary file 1 — LaTeX Supplementary File [file 41598_2019_43544_MOESM1_ESM.zip › Fig2.jpg]

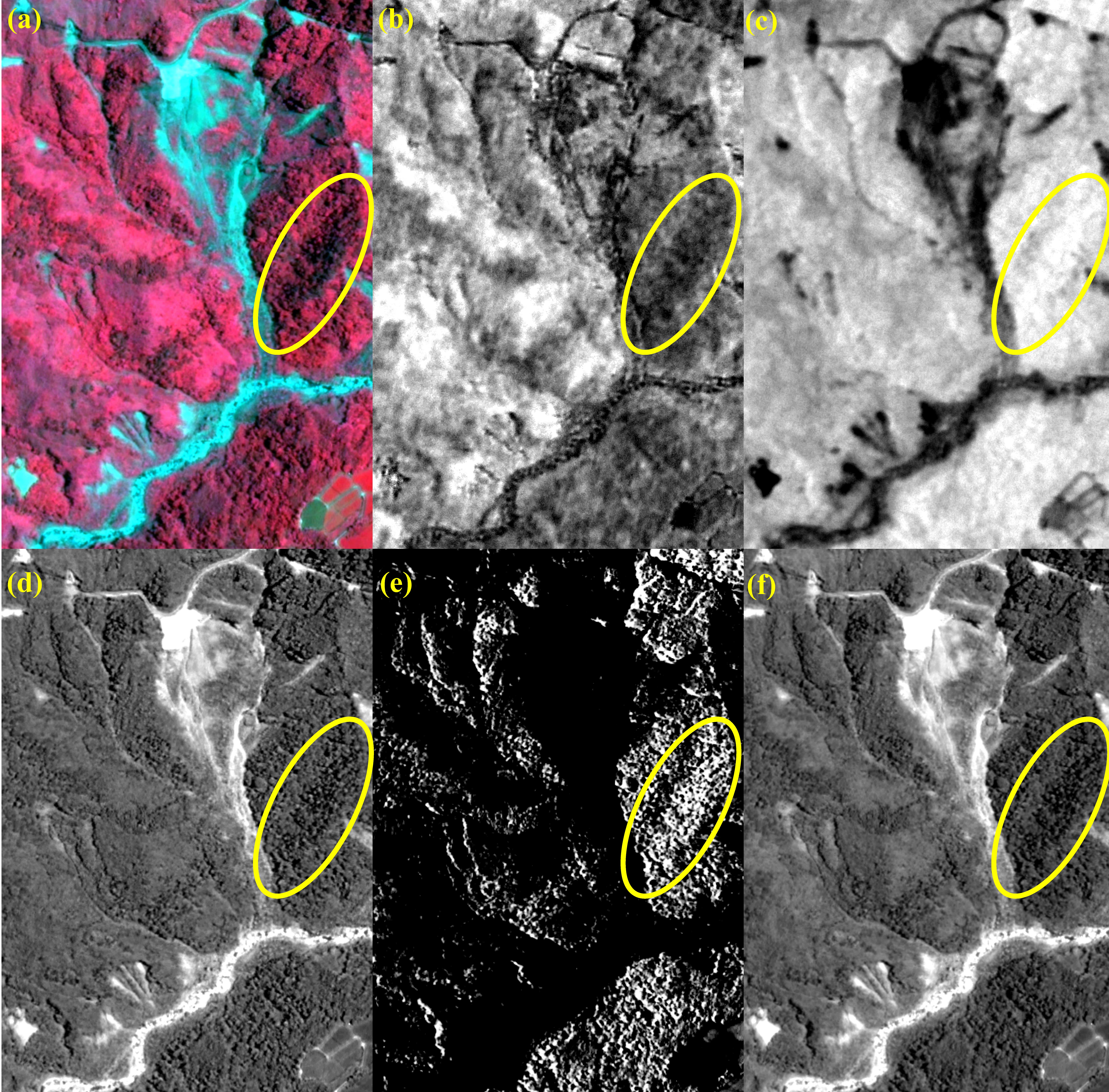

Supplement: Supplementary file 1 — LaTeX Supplementary File [file 41598_2019_43544_MOESM1_ESM.zip › Fig3.jpg]

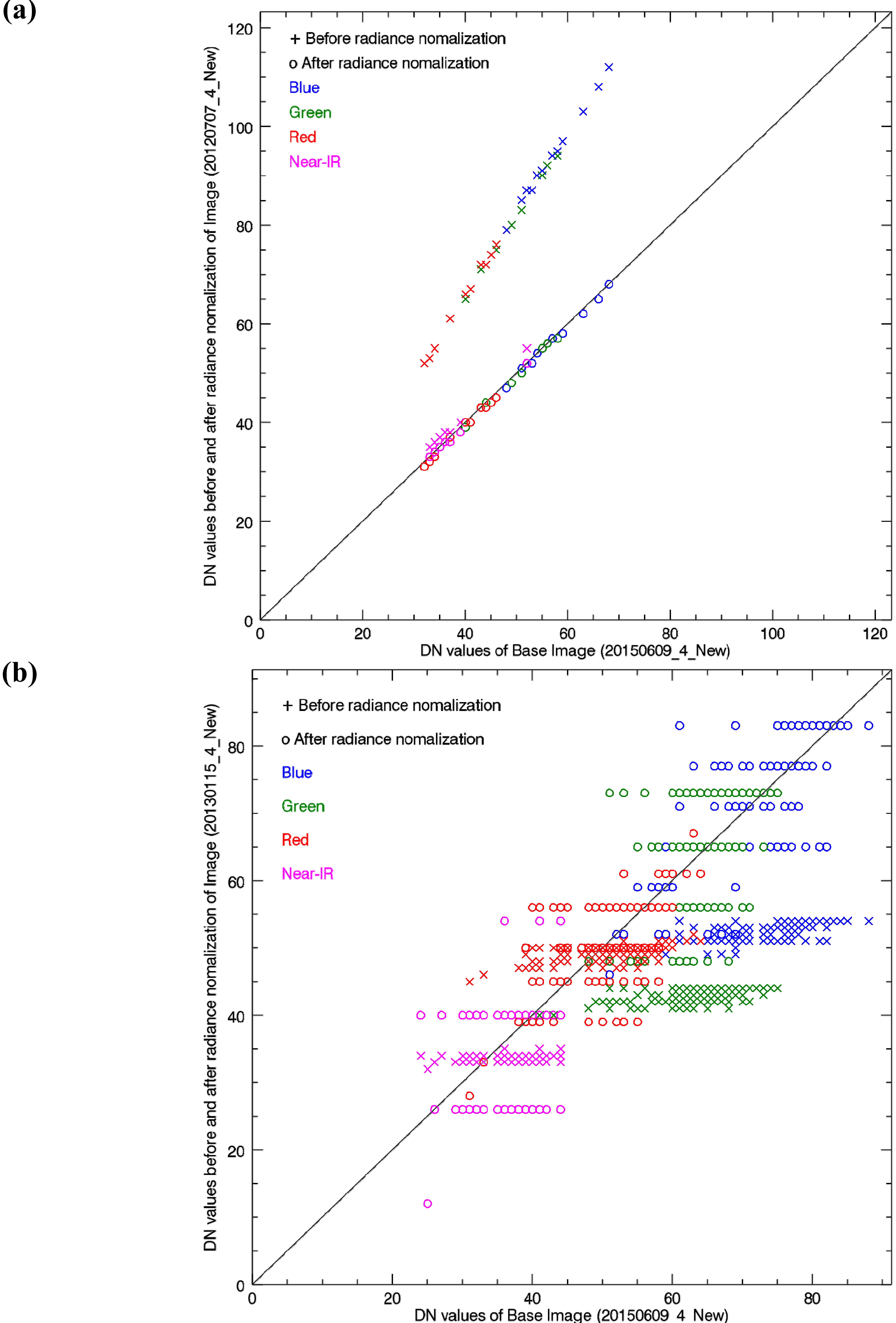

Supplement: Supplementary file 1 — LaTeX Supplementary File [file 41598_2019_43544_MOESM1_ESM.zip › Fig4.jpg]

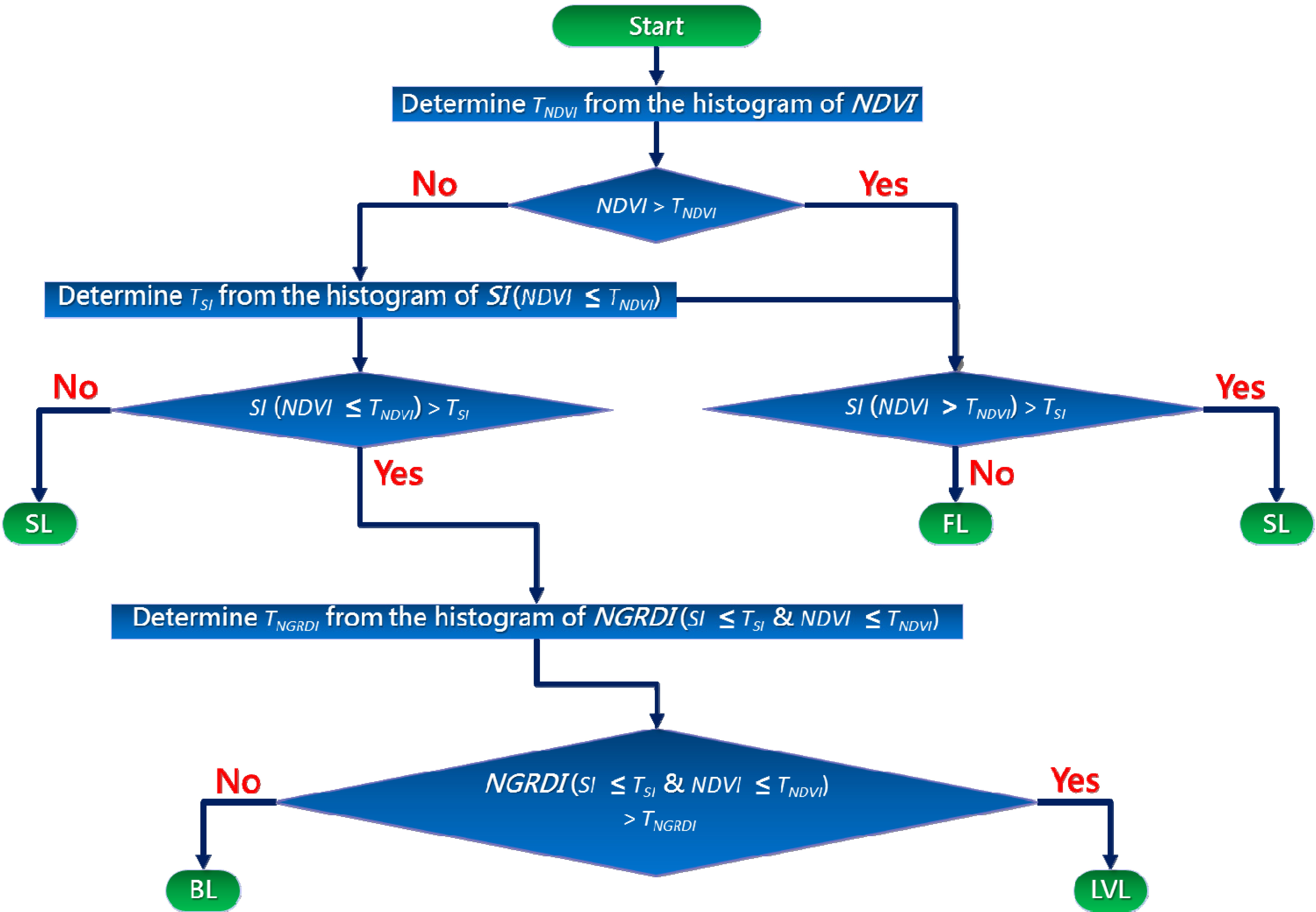

Supplement: Supplementary file 1 — LaTeX Supplementary File [file 41598_2019_43544_MOESM1_ESM.zip › Fig5.jpg]

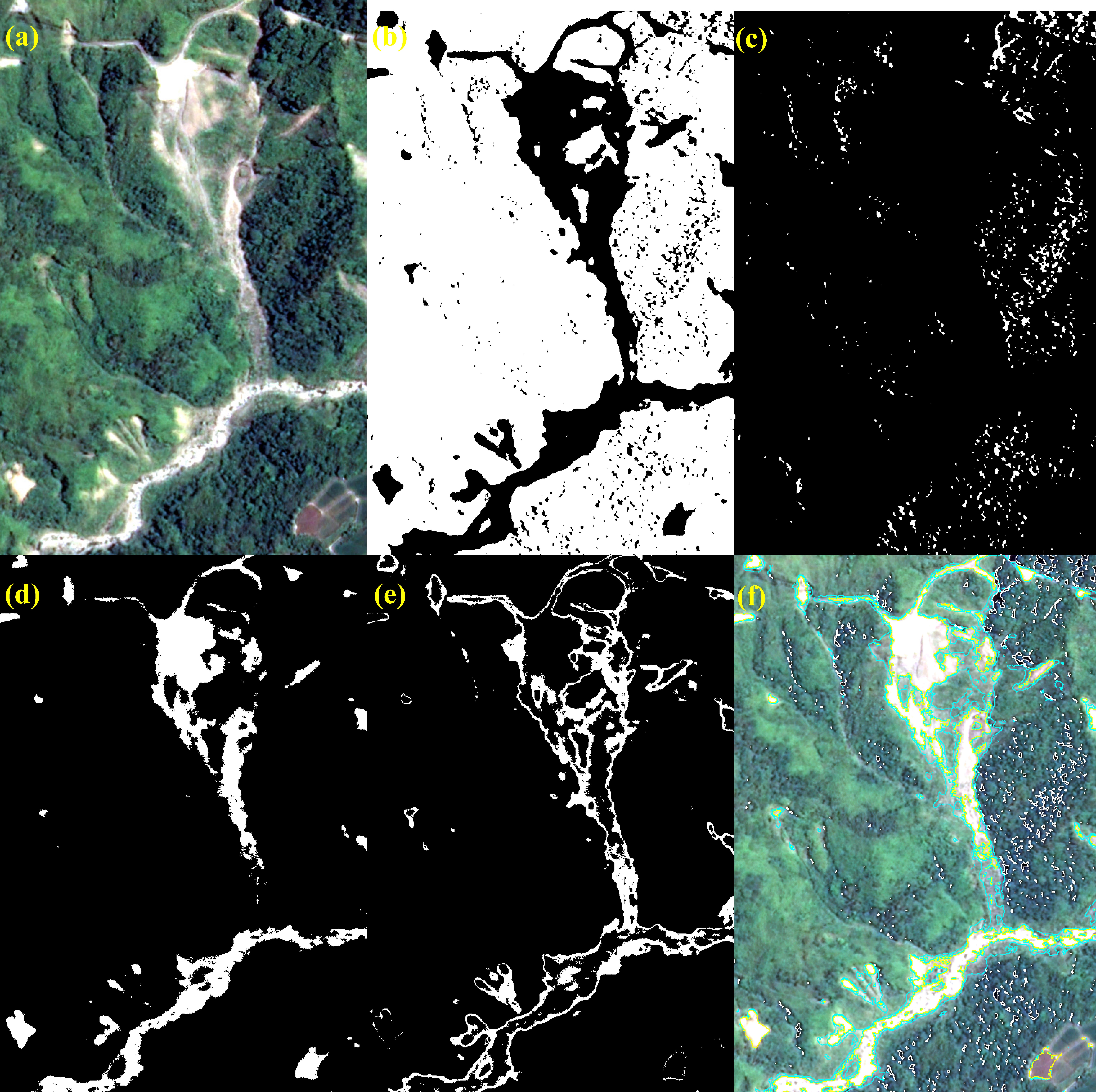

Supplement: Supplementary file 1 — LaTeX Supplementary File [file 41598_2019_43544_MOESM1_ESM.zip › Fig7.jpg]

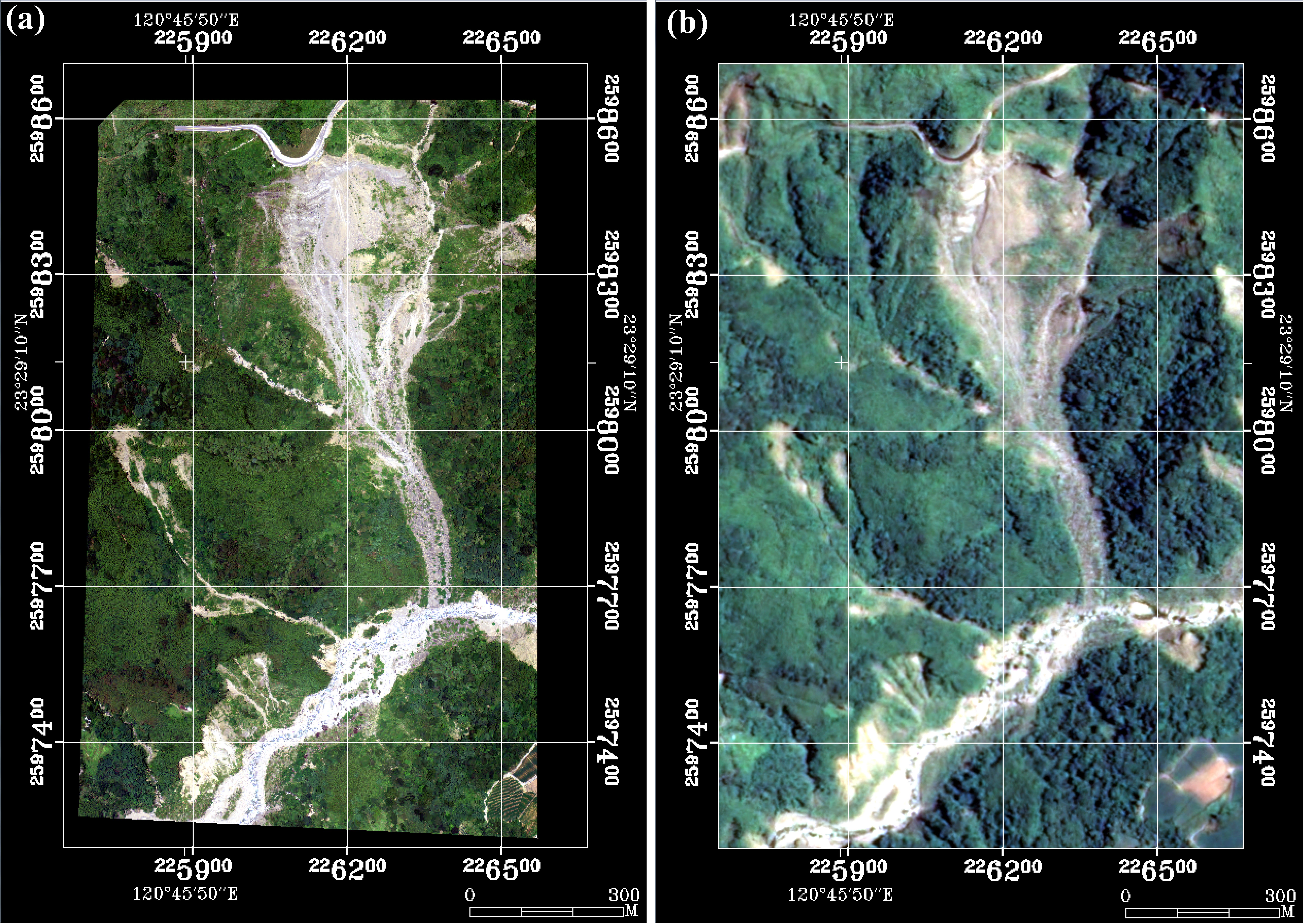

Supplement: Supplementary file 1 — LaTeX Supplementary File [file 41598_2019_43544_MOESM1_ESM.zip › Fig8.jpg]

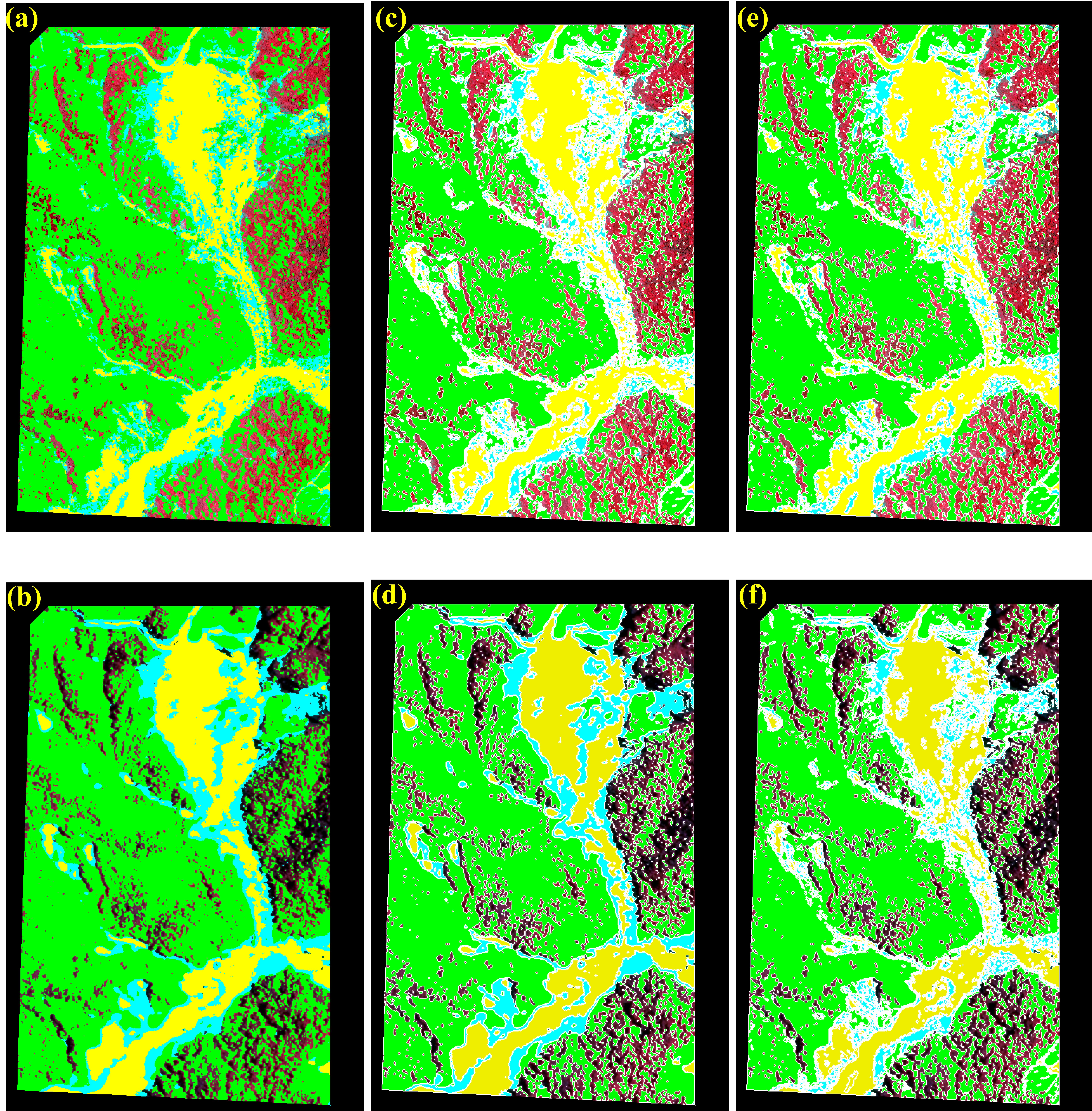

Supplement: Supplementary file 1 — LaTeX Supplementary File [file 41598_2019_43544_MOESM1_ESM.zip › Fig9.jpg]
