# Supplementary material for: Assessment of forest restoration with multitemporal remote sensing imagery
Source: Sci Rep. 2019 May 13;9:7279. doi: 10.1038/s41598-019-43544-5 (PMC6513895; doi:10.1038/s41598-019-43544-5)
Supplement: Supplementary file 2 — Supplementary Information [file 41598_2019_43544_MOESM2_ESM.pdf]

Assessment of forest restoration with multitemporal remote sensing imagery

Cheng-Chien Liu[1,2,*], **Yi-Hsin Chen**[2], **Mei-Heng Margaret Wu**[3], **Chiang Wei**[4], **and Ming-Hsun Ko**[2]

[1]Department of Earth Sciences, National Cheng Kung University, Tainan, 701, Taiwan

[2]Global Earth Observation and Data Analysis Center, National Cheng Kung University, Tainan, 701, Taiwan

[3]Department of Geography, University of California, Los Angeles, USA

[4]Experimental Forest, College of Bio-Resources and Agriculture, National Taiwan University, Nantou, Taiwan

[*]ccliu88@mail.ncku.edu.tw

Supplement A. All quick-look images of 1,453 strips covered our study areas (Strip Nos. 3, 4 and 5 of Orbit 1) acquired by Formosat-2 during twelve years of operations and services from 2005 to 2016. The date of acquisition is annotated in each image.

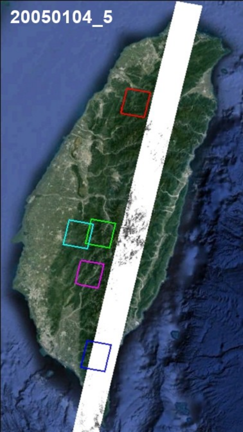
20050104_5

20050306_5

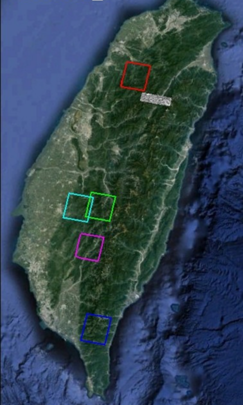

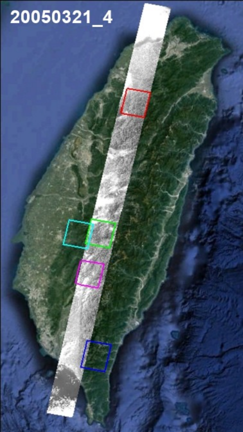
20050321_4

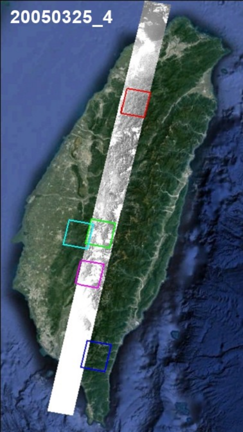
20050325_4

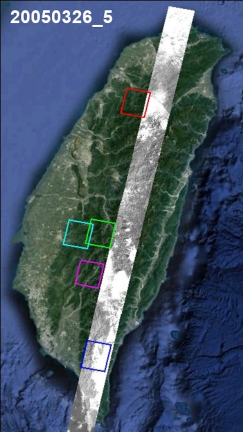
20050326_5

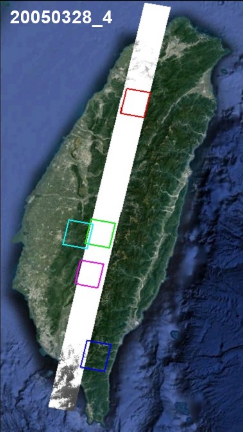
20050328_4

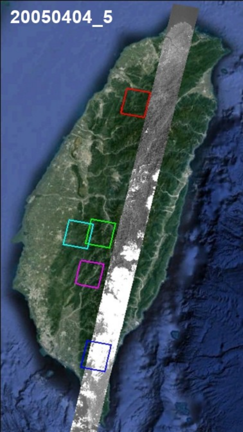
20050404_5

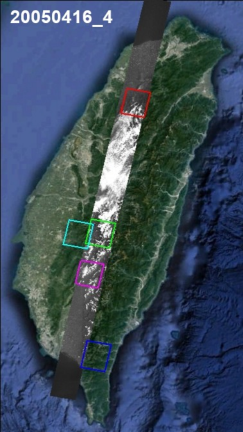
20050416_4

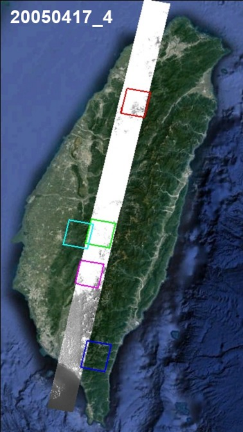
20050417_4

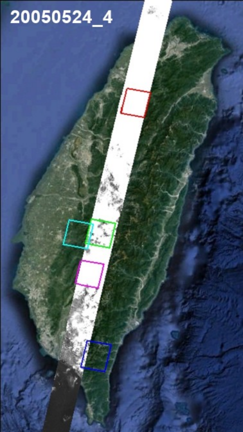
20050524_4

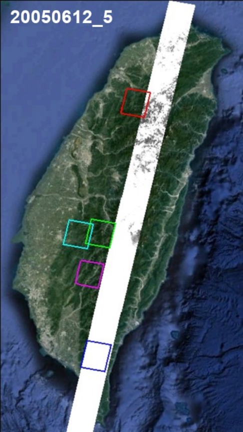
20050612_5

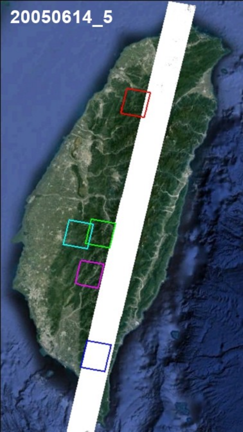
20050614_5

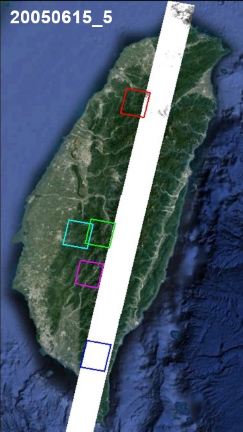
20050615_5

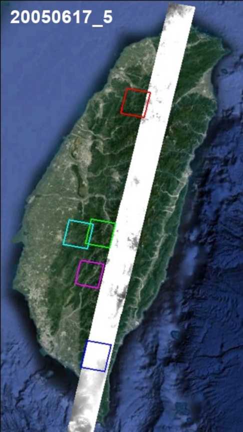
20050617_5

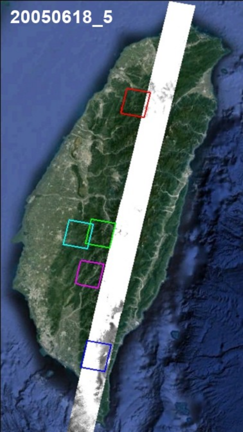
20050618_5

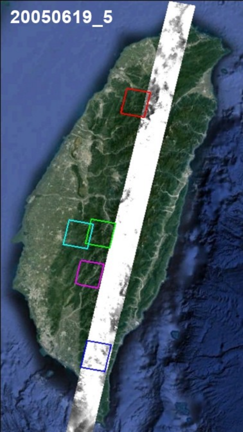
20050619_5

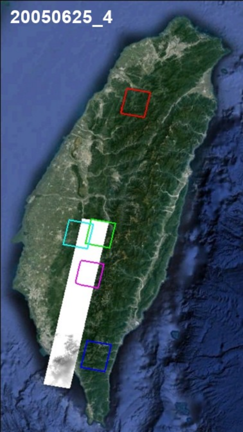
20050625_4

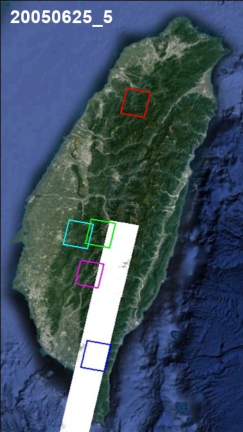
20050625_5

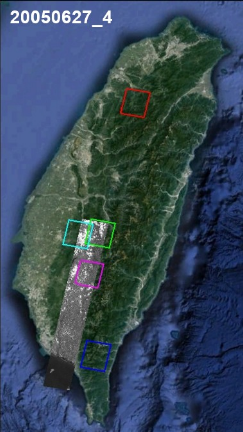
20050627_4

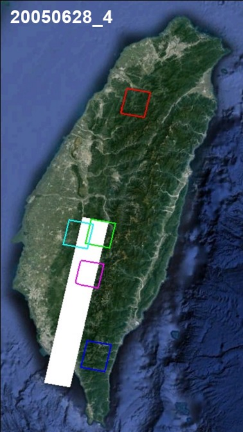
20050628_4

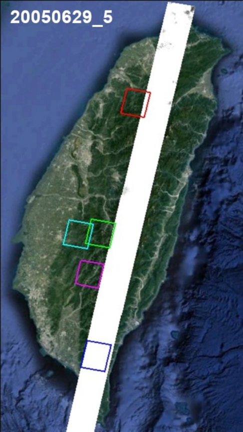
20050629_5

20050701_5

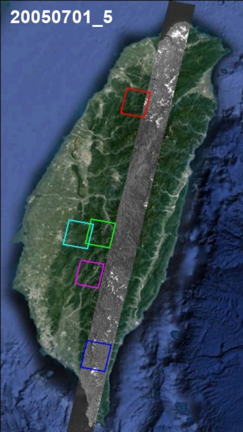

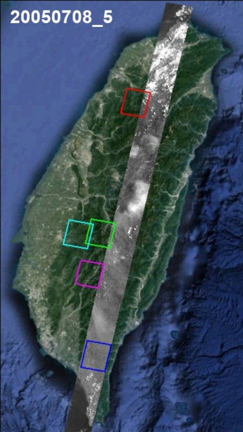
20050708_5

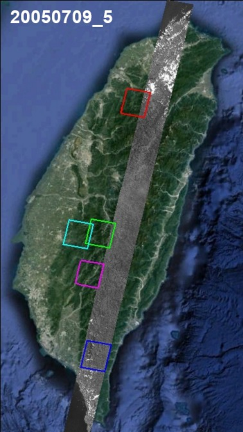
20050709_5

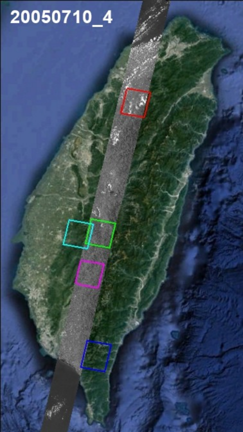
20050710_4

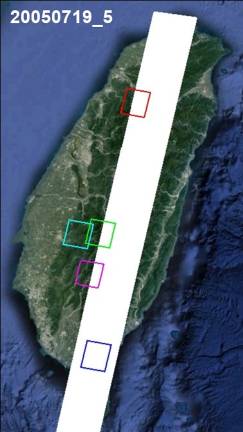
20050719_5

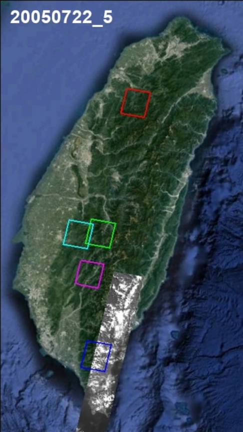
20050722_5

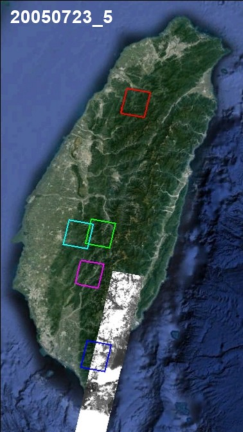
20050723_5

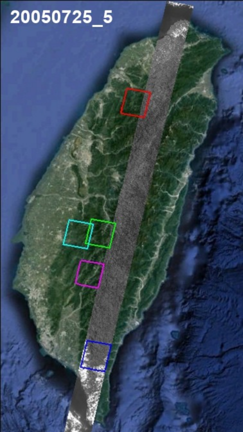
20050725_5

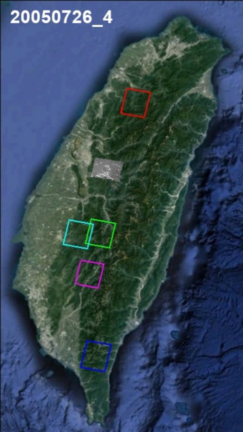
20050726_4

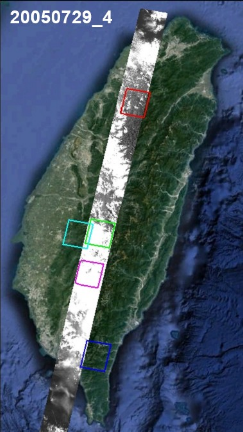
20050729_4

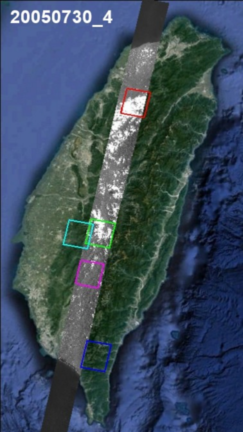
20050730_4

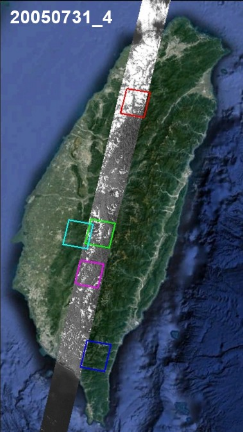
20050731_4

20050801_4

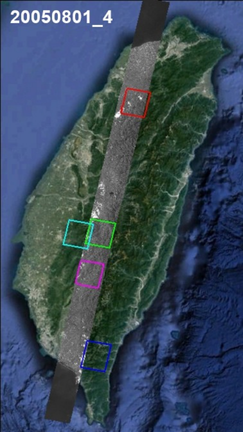

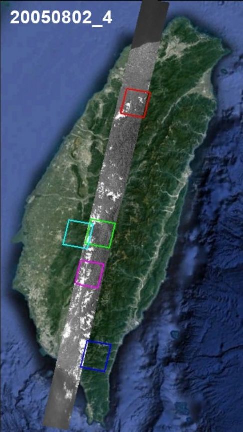
20050802_4

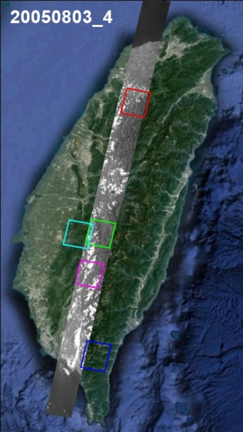
20050803_4

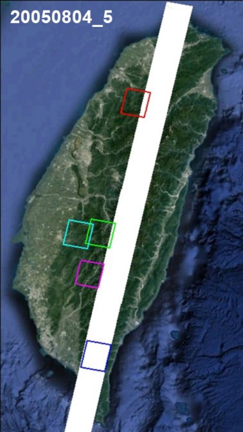
20050804_5

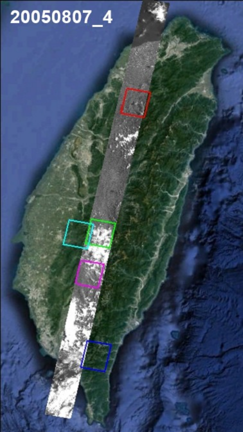
20050807_4

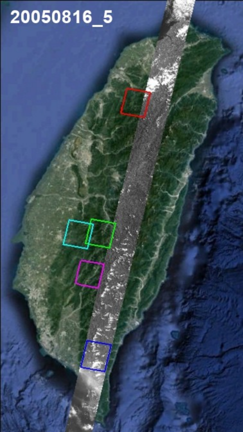
20050816_5

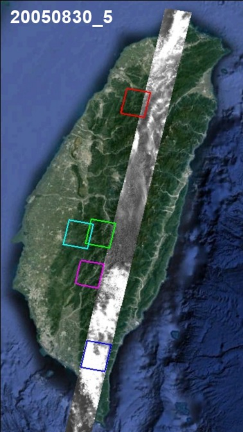

20050830_5

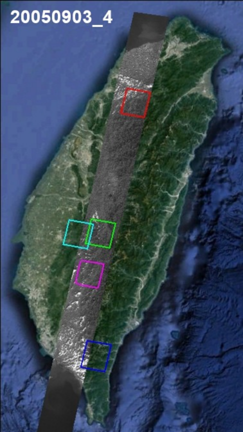
20050903_4

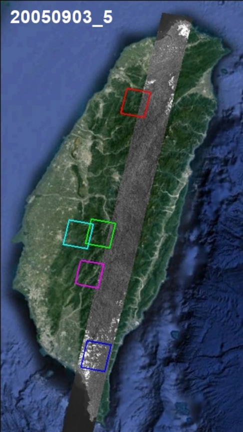
20050903_5

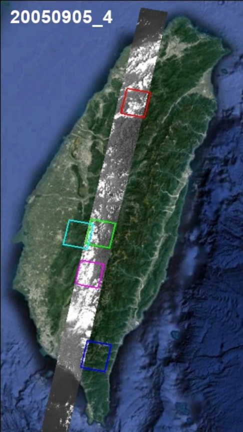
20050905_4

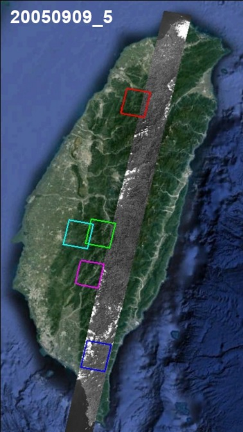
20050909_5

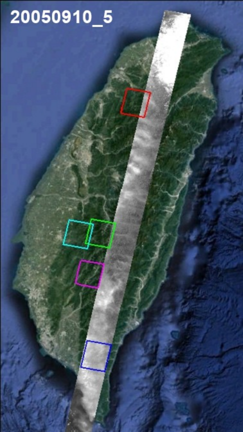
20050910_5

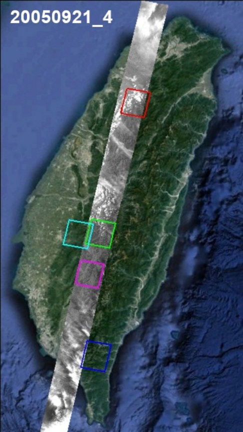
20050921_4

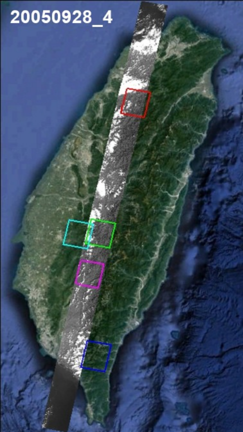
20050928_4

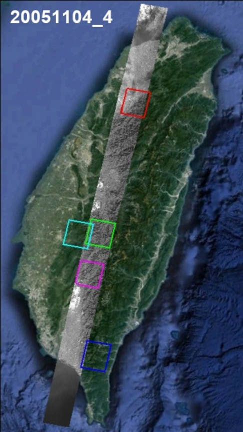
20051104_4

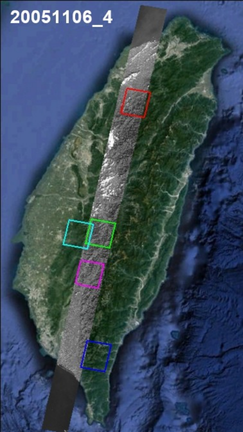
20051106_4

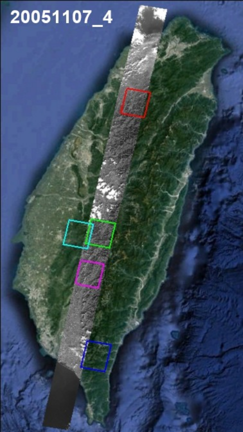
20051107_4

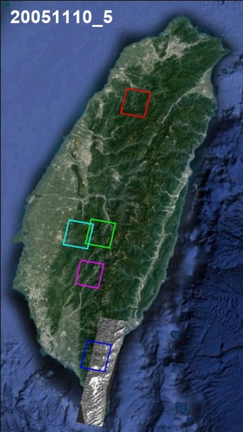
20051110_5

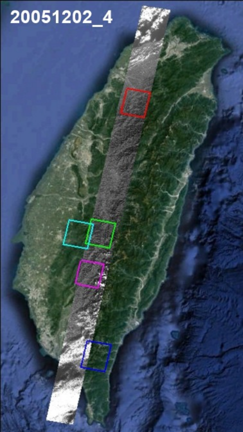
20051202_4

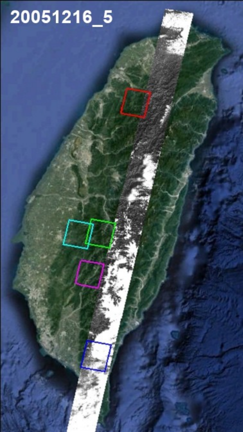
20051216_5

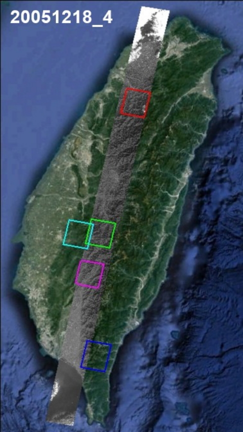
20051218_4

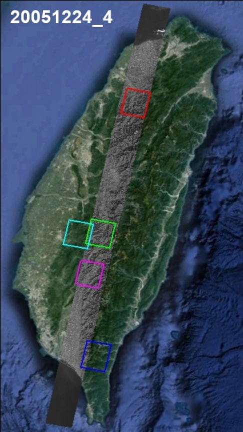
20051224_4

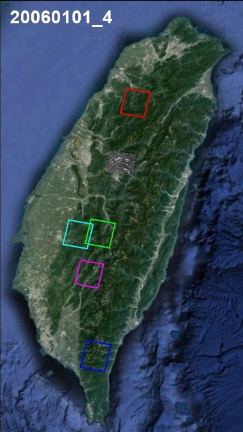
20060101_4

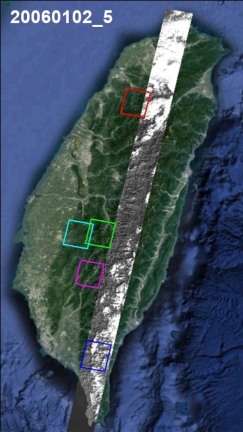
20060102_5

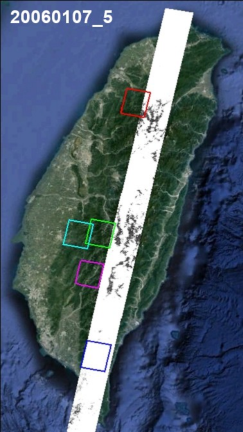
20060107_5

20060126_5

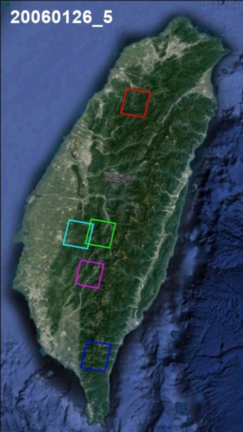

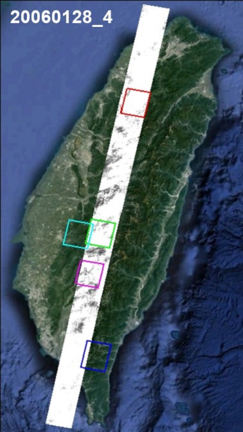
20060128_4

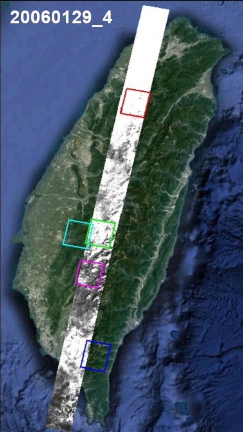
20060129_4

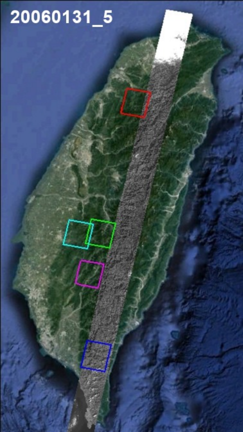
20060131_5

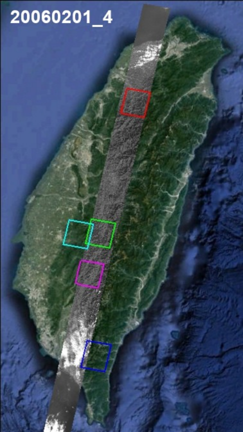
20060201_4

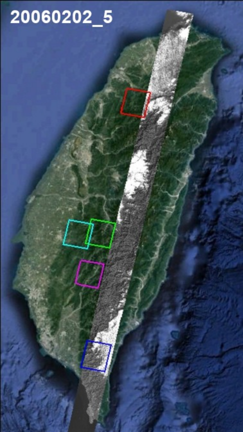
20060202_5

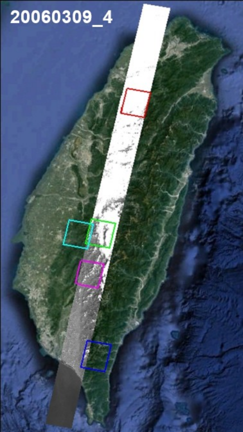
20060309_4

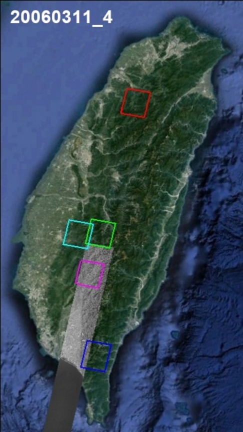
20060311_4

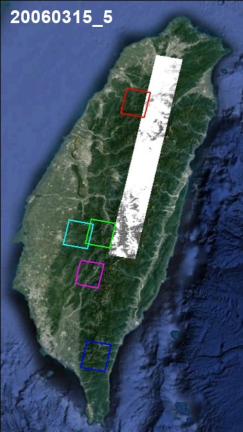
20060315_5

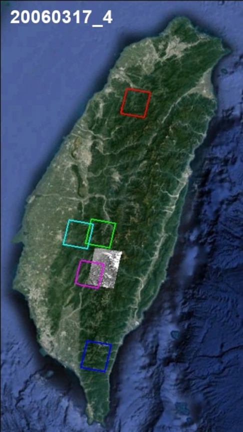
20060317_4

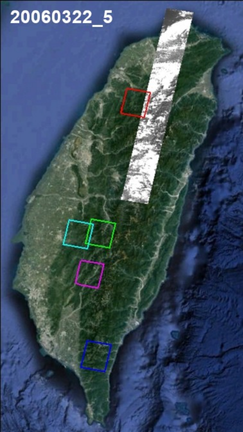
20060322_5

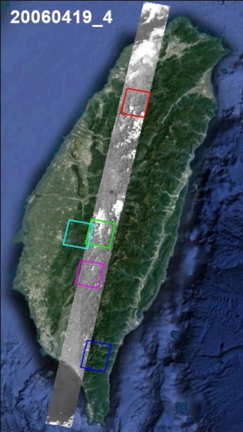
20060419_4

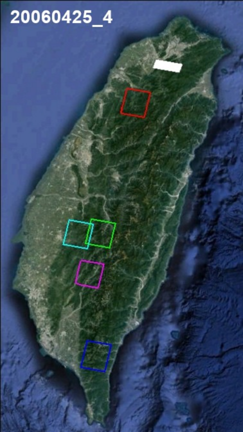
20060425_4

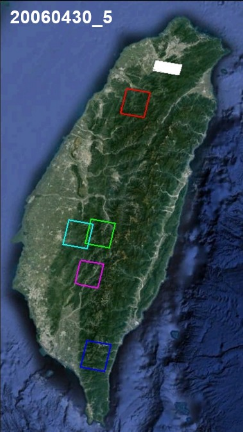
20060430_5

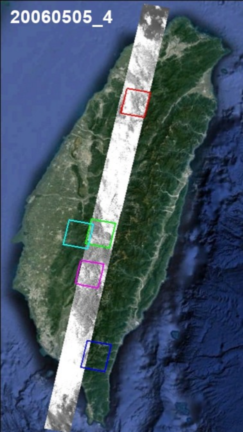
20060505_4

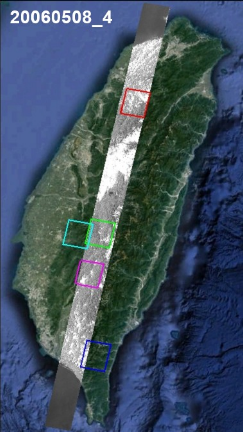
20060508_4

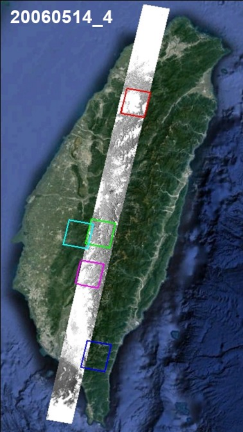
20060514_4

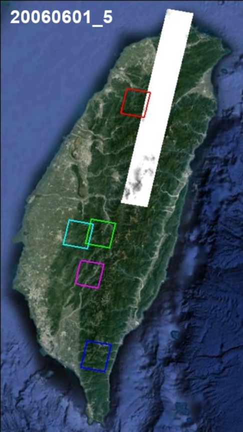
20060601_5

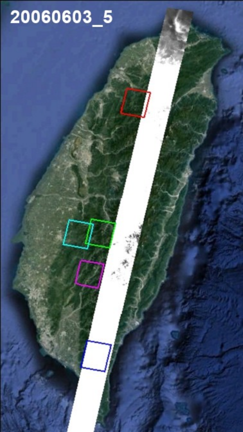
20060603_5

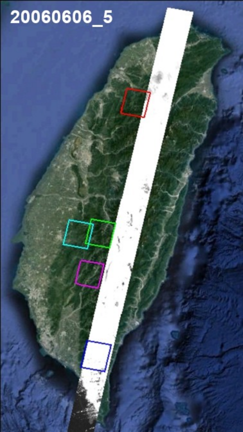
20060606_5

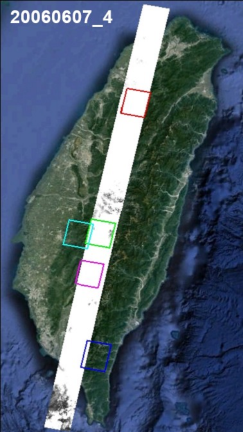
20060607_4

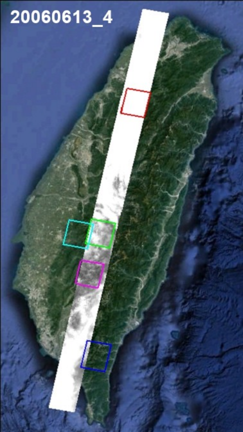
20060613_4

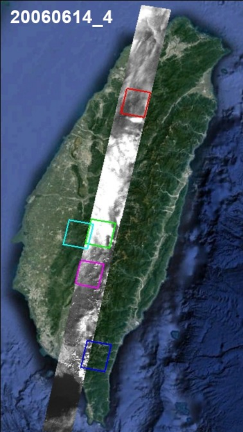
20060614_4

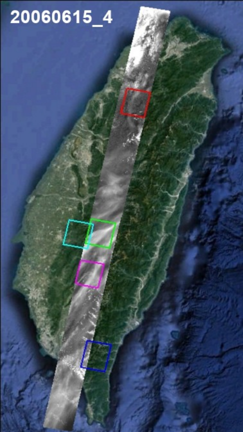
20060615_4

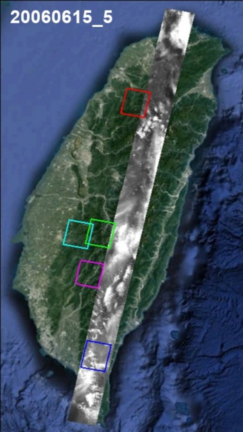
20060615_5

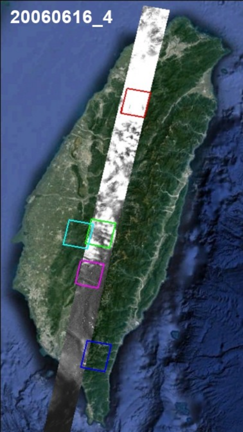

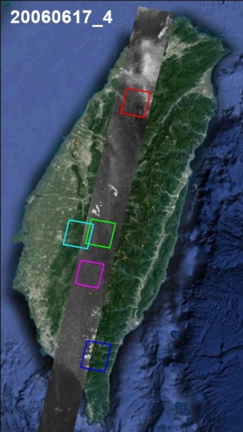
20060617_4

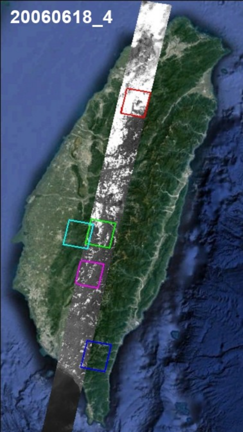
20060618_4

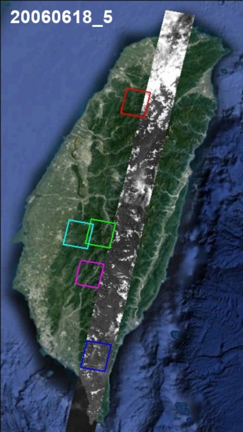
20060618_5

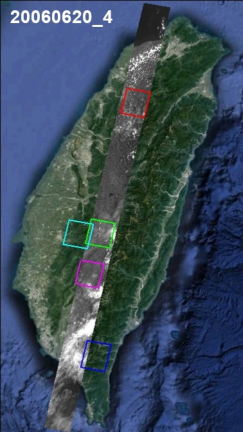
20060620_4

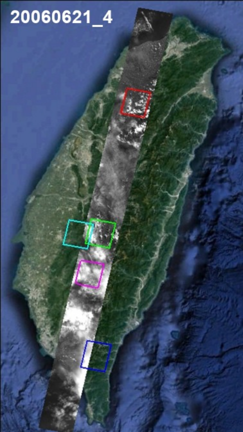
20060621_4

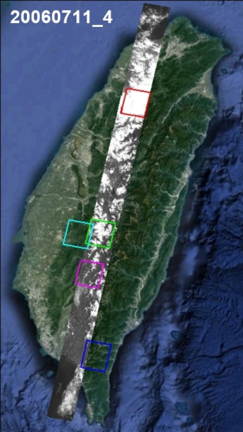
20060711_4

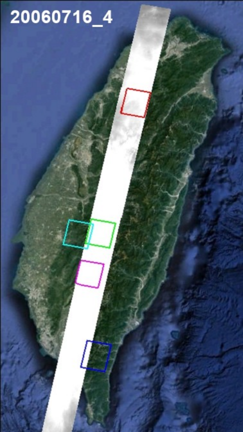
20060716_4

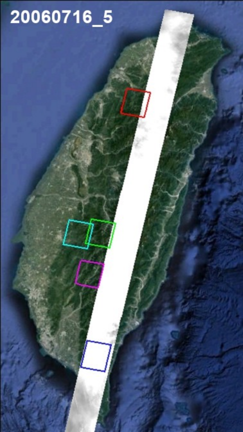
20060716_5

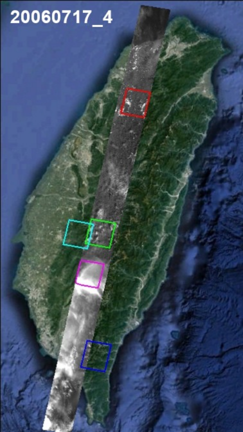
20060717_4

20060717_5

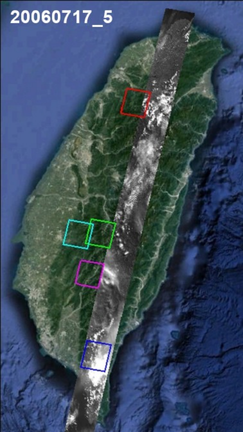

20060719_5

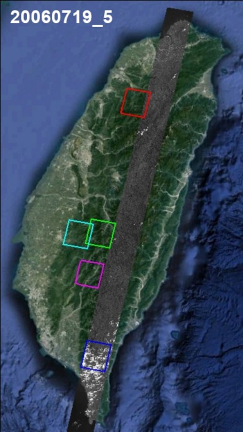

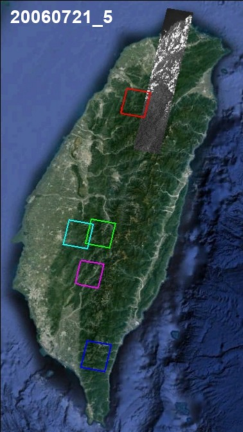
20060721_5

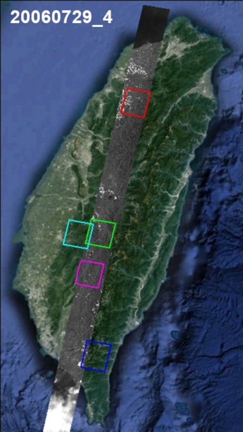
20060729_4

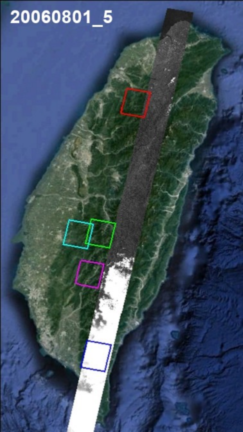
20060801_5

20060804_5

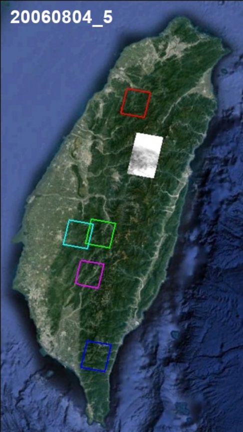

20060804_5

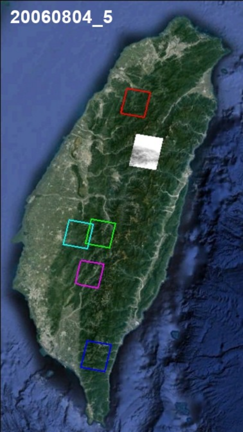

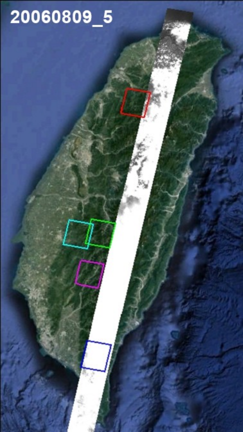
20060809_5

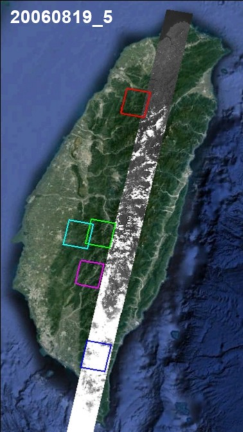
20060819_5

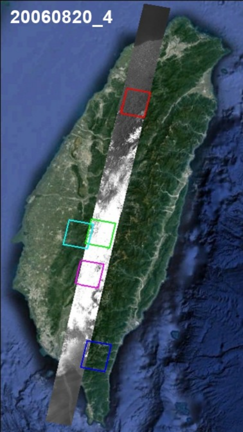
20060820_4

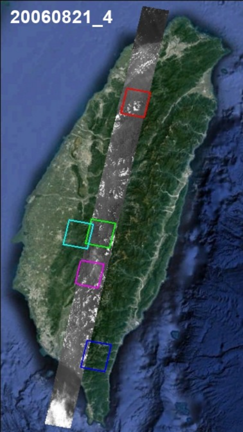
20060821_4

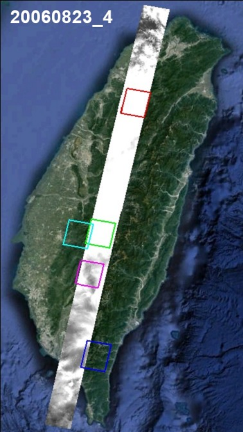
20060823_4

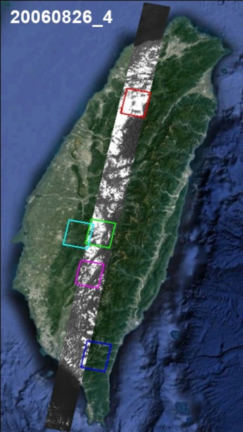
20060826_4

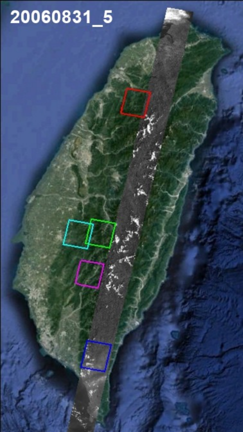
20060831_5

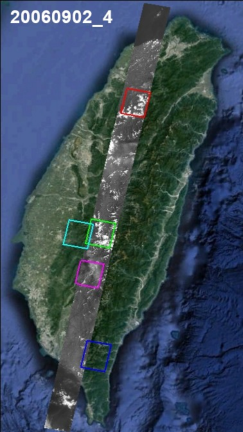
20060902_4

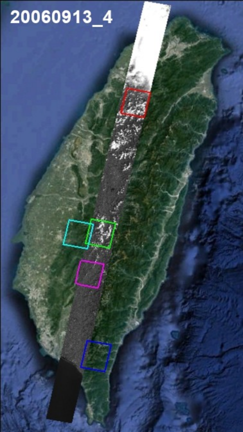
20060913_4

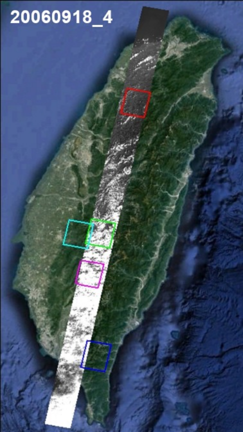
20060918_4

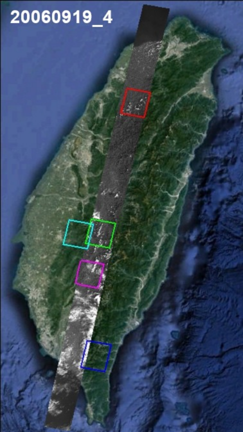
20060919_4

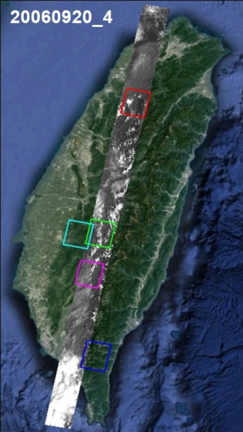
20060920_4

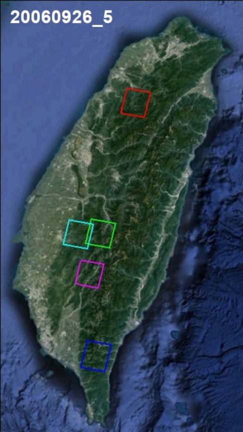
20060926_5

20060926_5

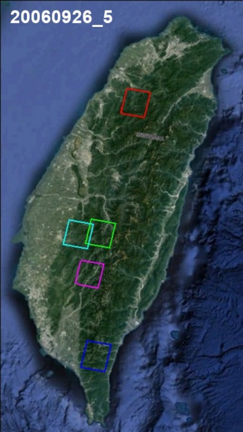

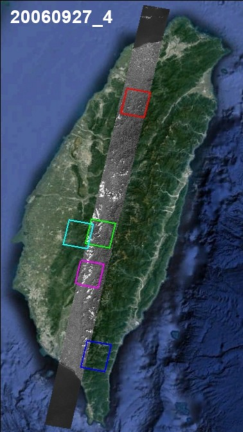
20060927_4

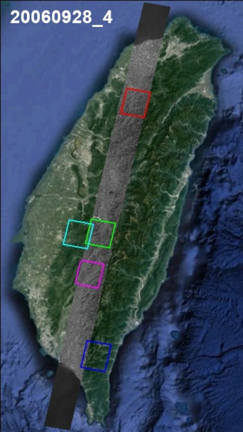
20060928_4

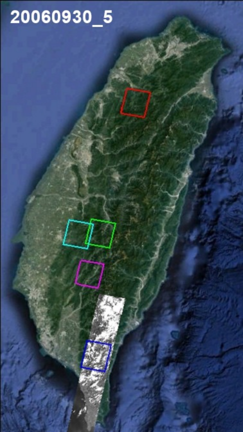
20060930_5

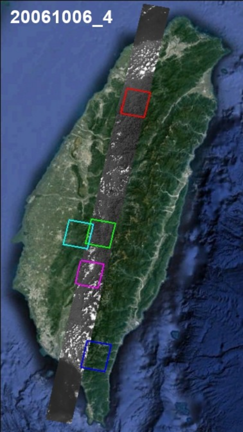
20061006_4

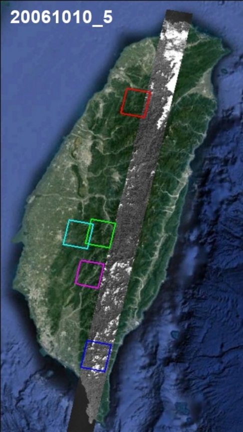
20061010_5

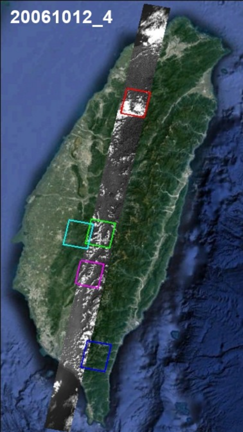
20061012_4

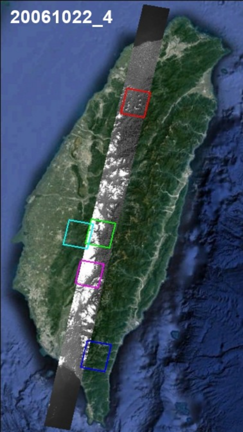
20061022_4

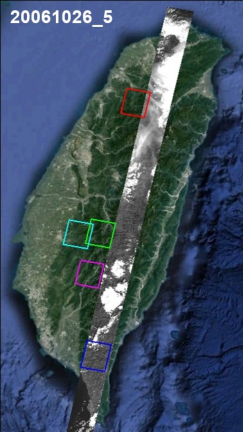
20061026_5

20061030_5

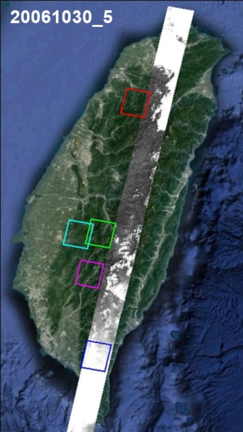

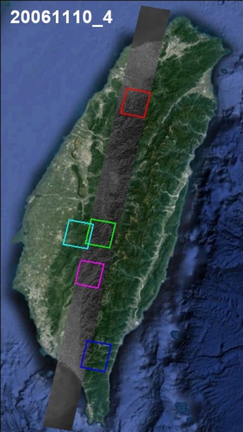
20061110_4

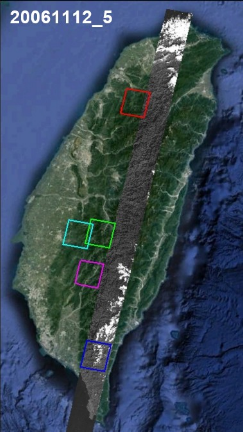
20061112_5

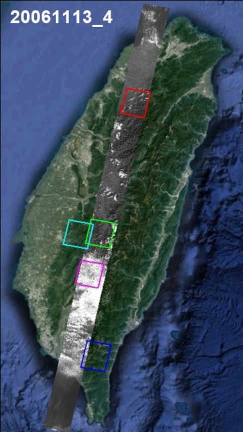
20061113_4

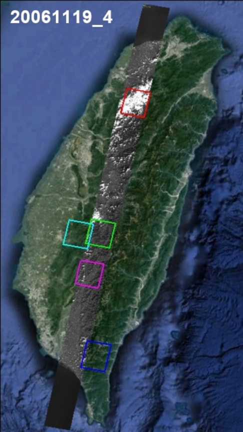
20061119_4

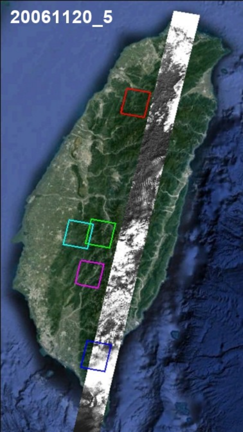
20061120_5

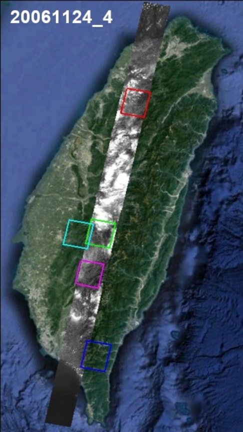
20061124_4

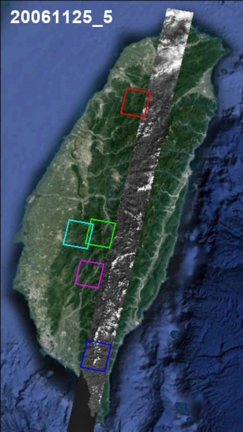
20061125_5

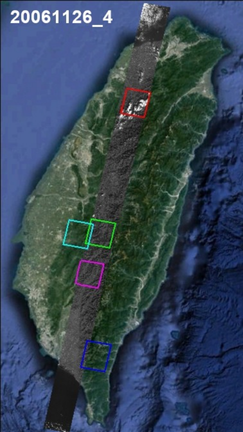
20061126_4

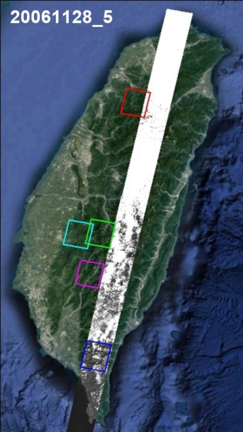
20061128_5

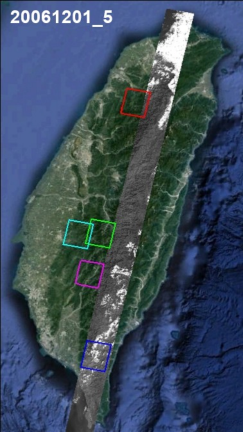
20061201_5

20061205_4

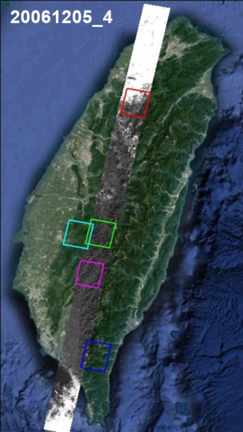

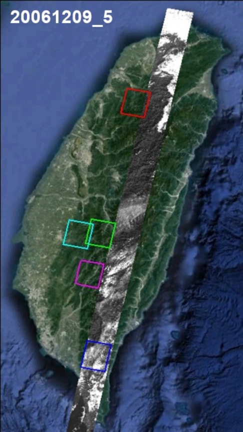

20061209_5

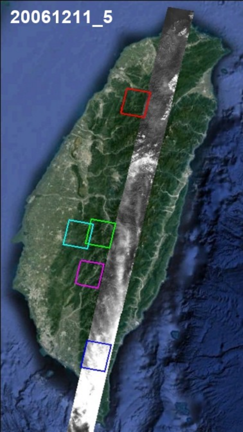
20061211_5

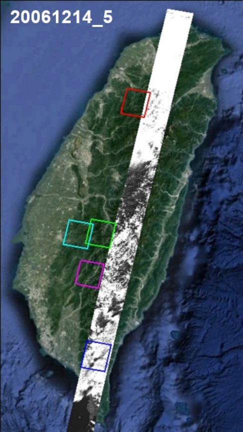
20061214_5

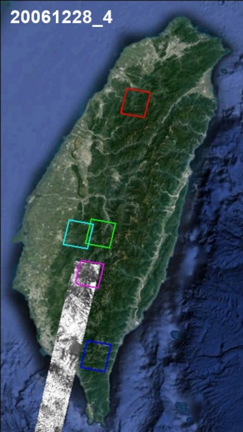
20061228_4

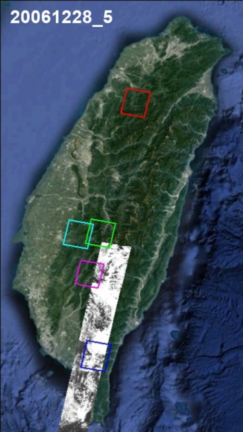
20061228_5

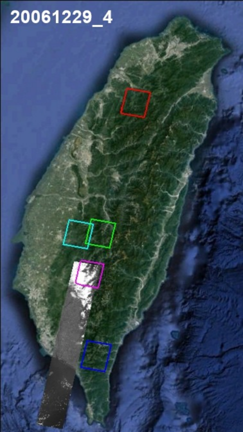
20061229_4

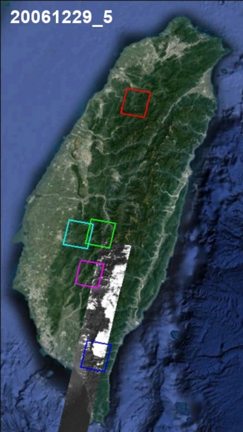
20061229_5

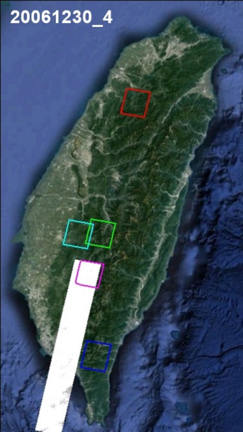
20061230_4

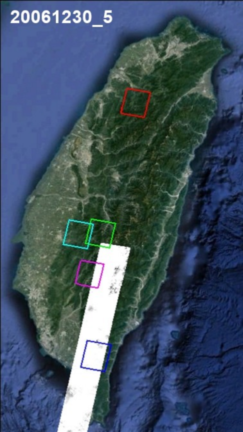
20061230_5

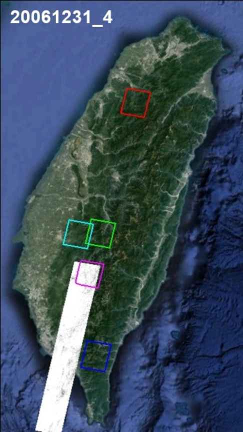
20061231_4

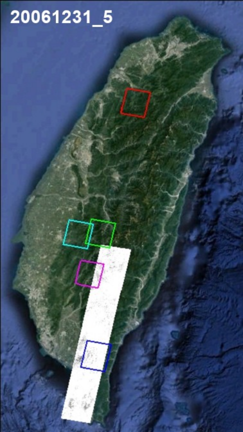
20061231_5

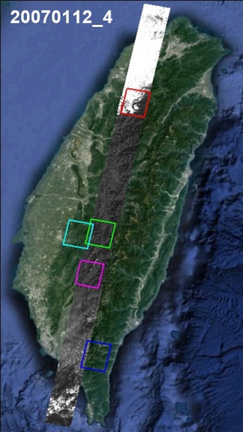
20070112_4

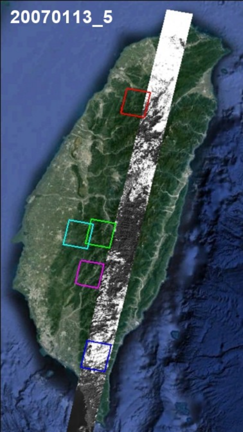
20070113_5

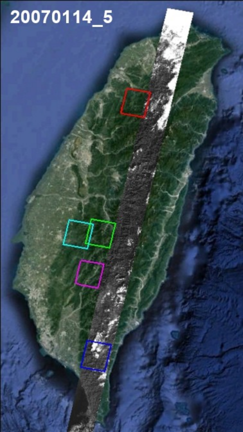
20070114_5

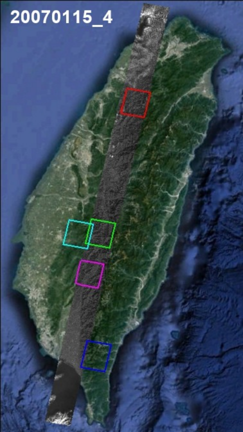
20070115_4

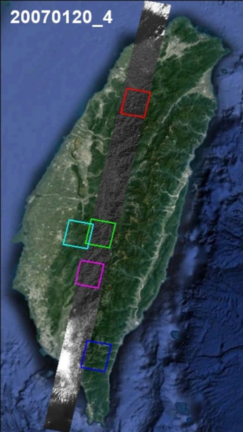
20070120_4

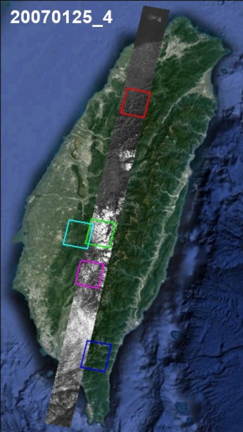
20070125_4

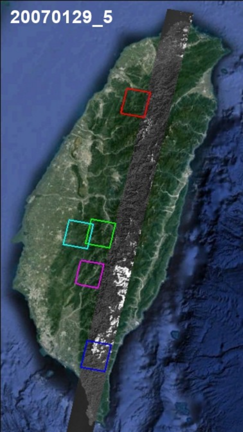
20070129_5

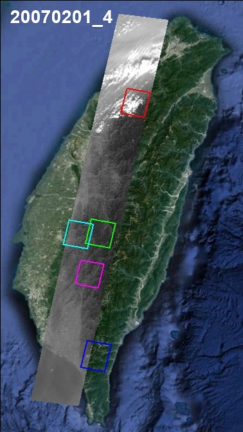
20070201_4

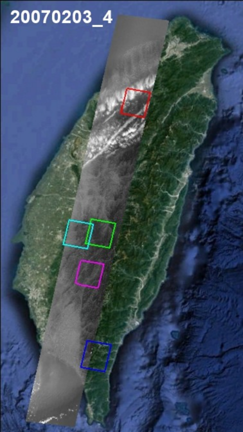
20070203_4

20070205_5

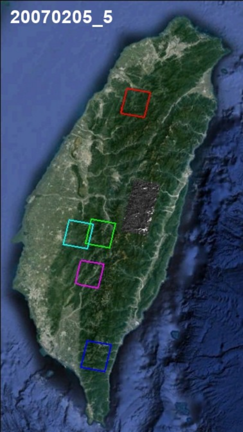

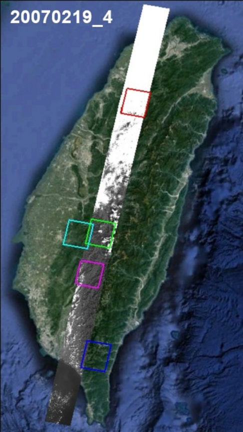
20070219_4

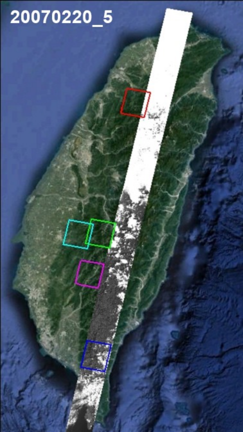
20070220_5

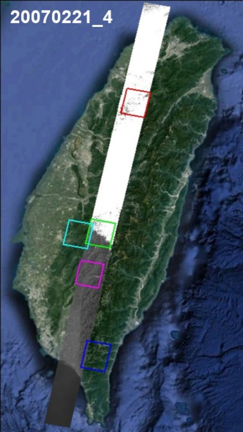
20070221_4

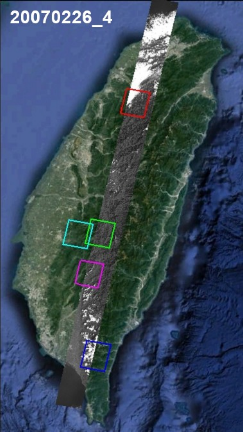
20070226_4

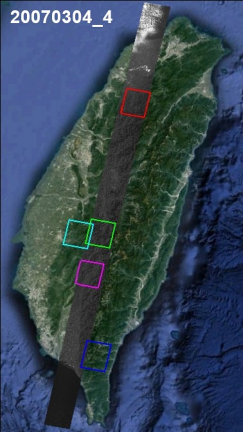
20070304_4

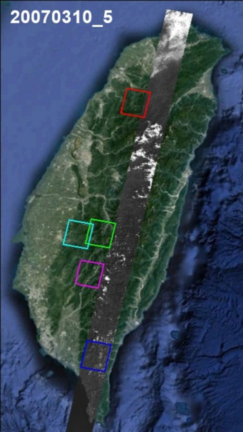
20070310_5

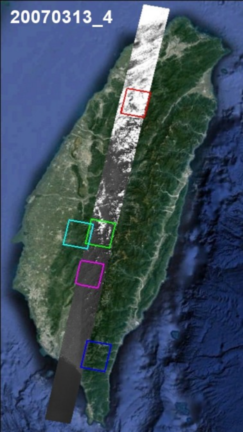
20070313_4

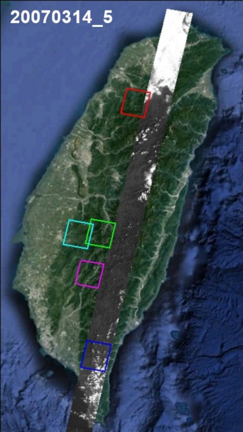
20070314_5

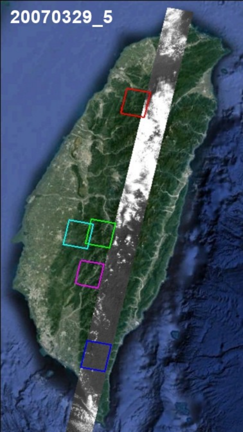
20070329_5

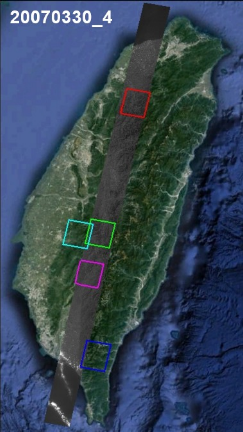
20070330_4

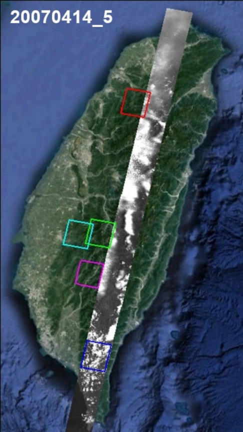
20070414_5

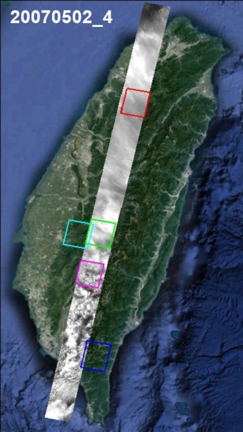
20070502_4

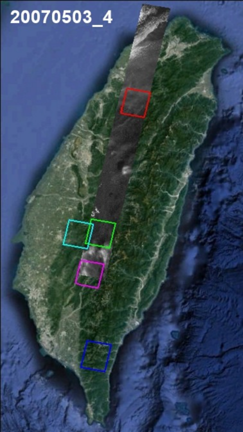
20070503_4

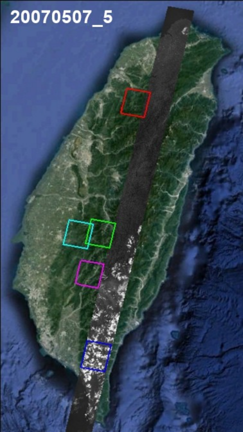
20070507_5

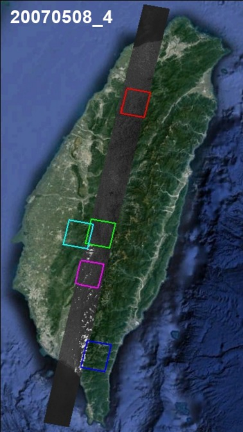
20070508_4

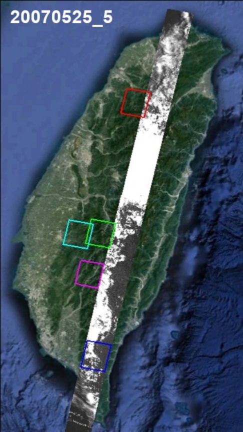
20070525_5

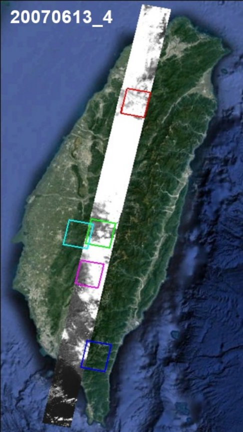
20070613_4

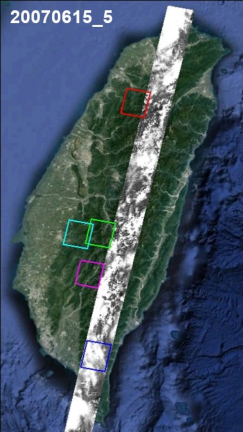
20070615_5

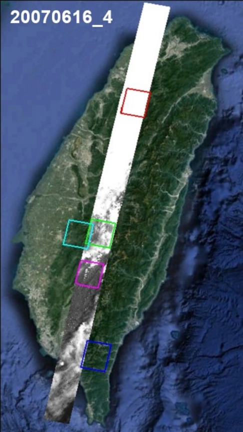
20070616_4

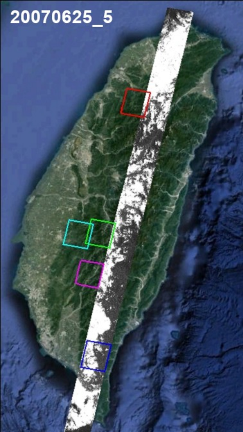
20070625_5

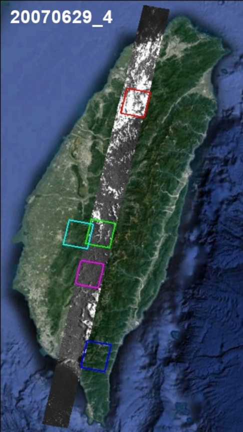
20070629_4

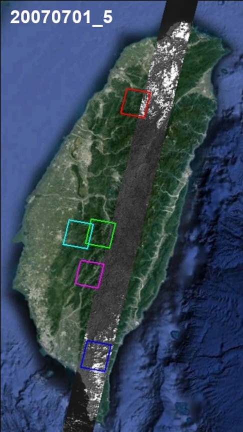
20070701_5

20070703_4

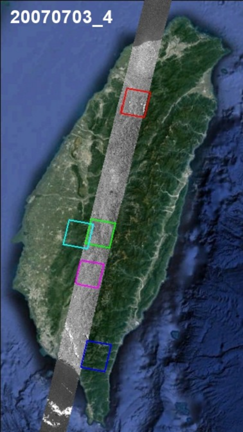

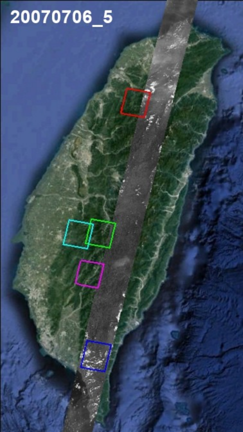
20070706_5

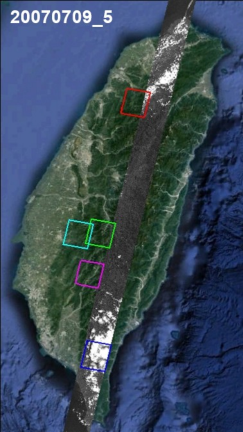
20070709_5

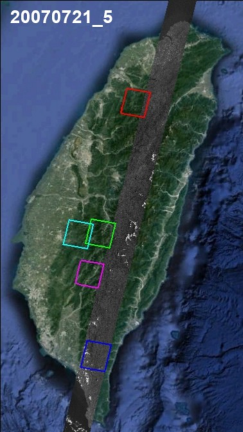
20070721_5

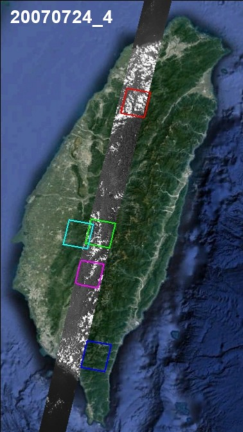
20070724_4

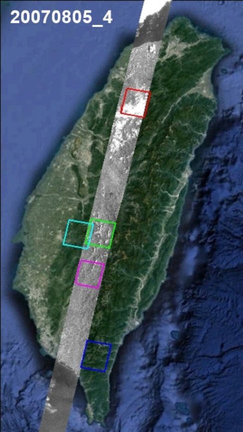
20070805_4

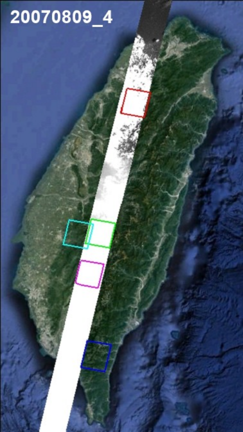

20070809_4

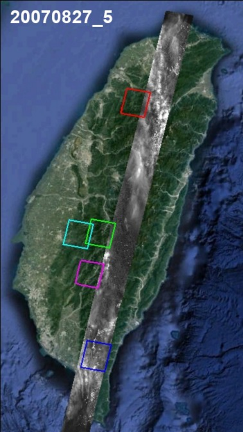
20070827_5

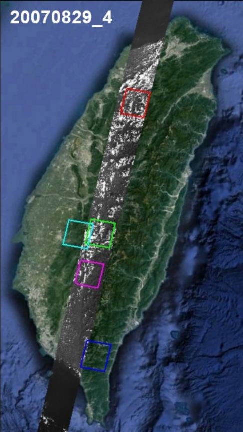
20070829_4

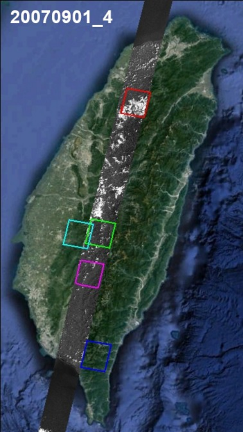
20070901_4

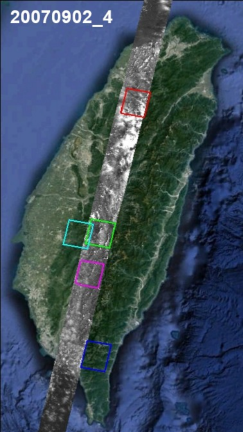
20070902_4

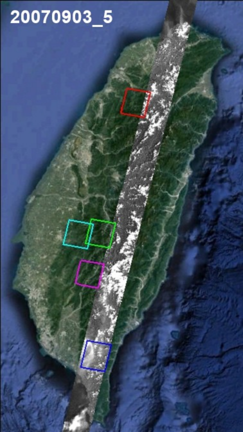
20070903_5

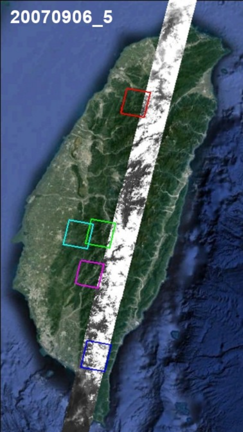
20070906_5

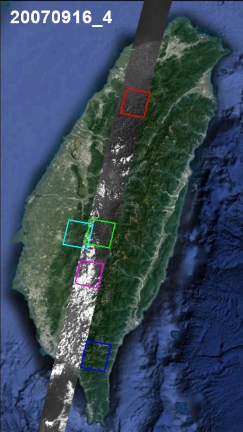
20070916_4

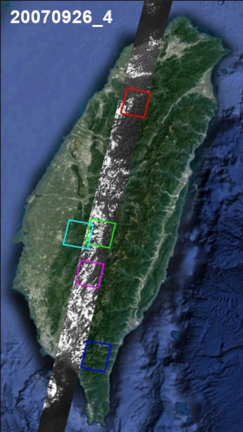
20070926_4

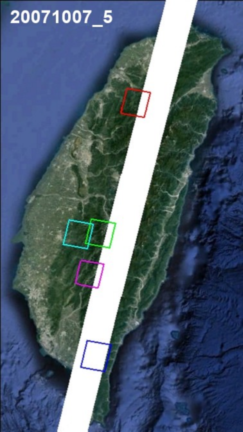
20071007_5

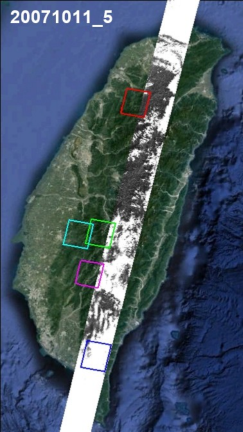
20071011_5

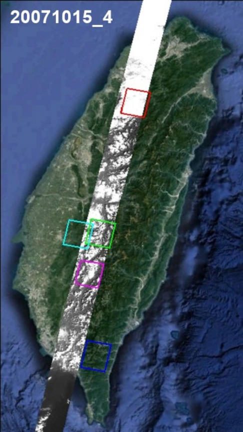
20071015_4

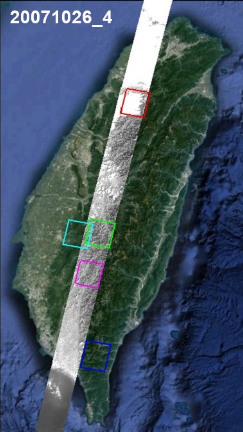
20071026_4

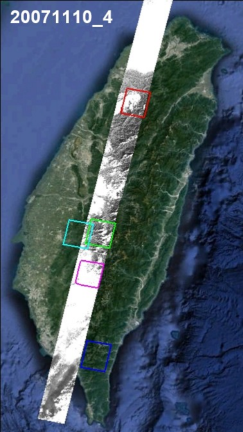
20071110_4

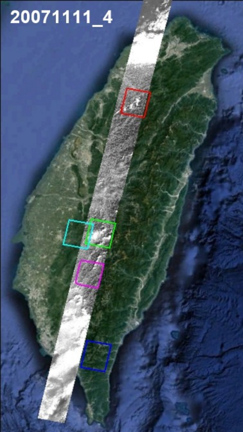
20071111_4

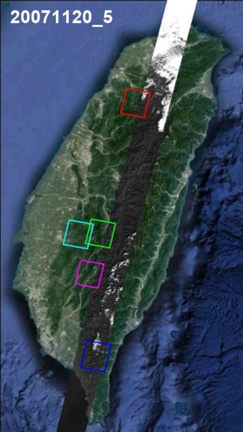
20071120_5

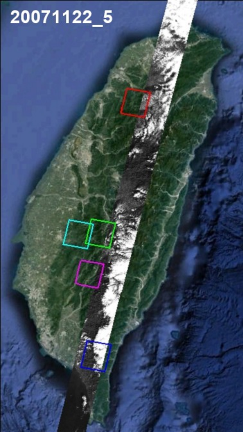
20071122_5

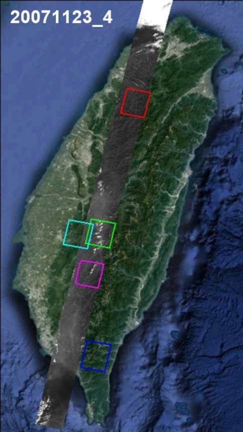
20071123_4

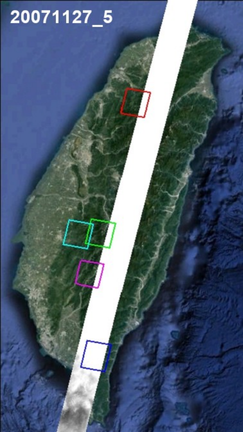
20071127_5

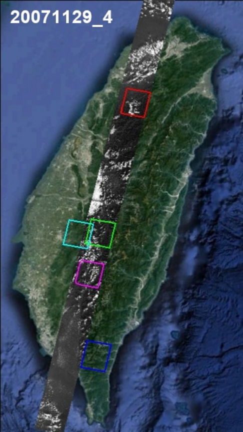
20071129_4

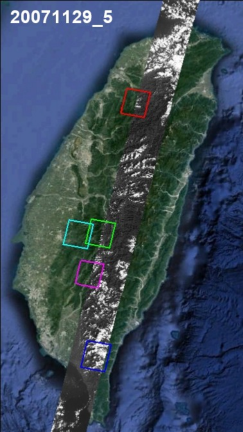
20071129_5

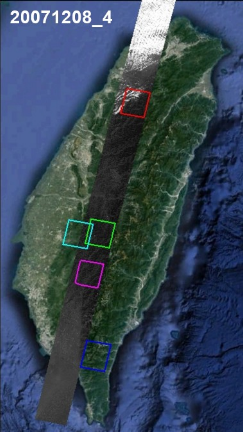
20071208_4

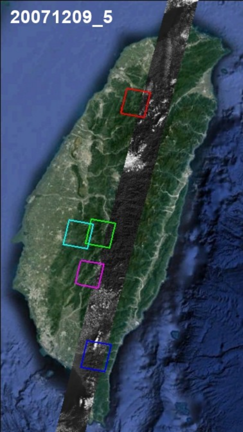
20071209_5

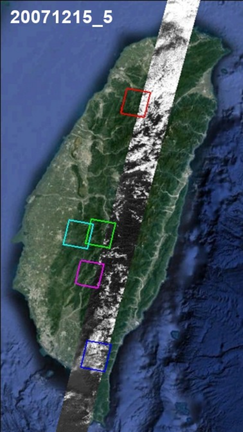
20071215_5

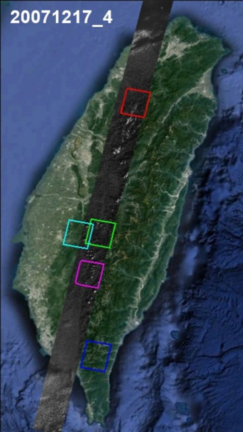
20071217_4

20071221_5

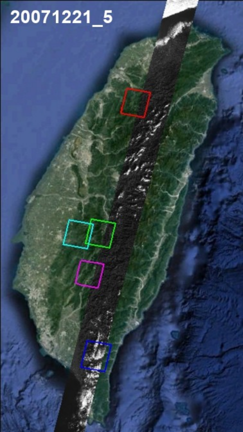

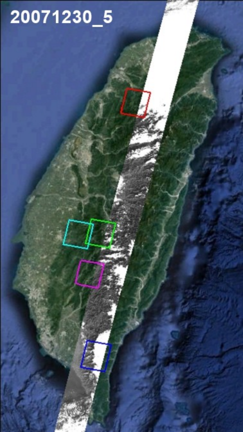
20071230_5

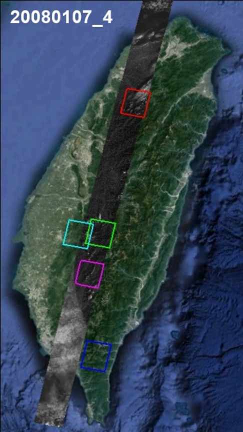
20080107_4

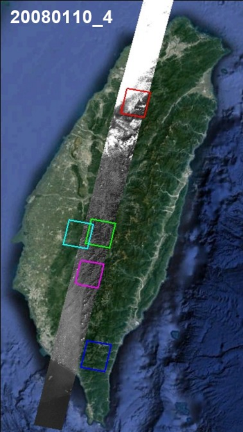
20080110_4

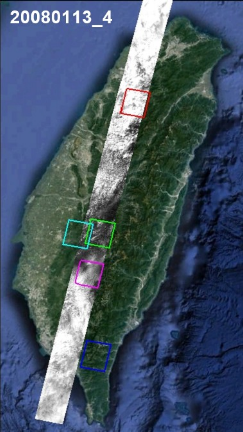
20080113_4

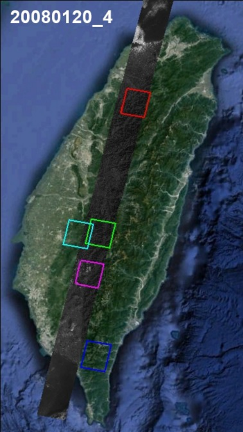
20080120_4

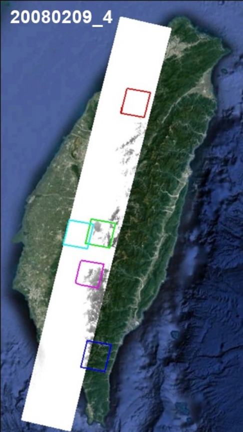
20080209_4

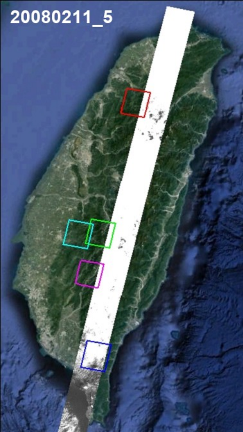
20080211_5

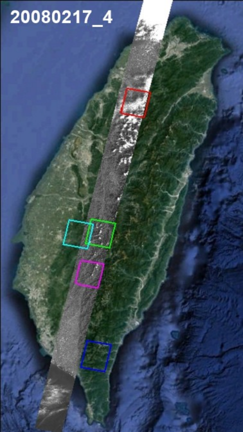
20080217_4

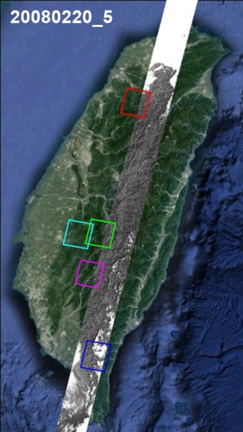
20080220_5

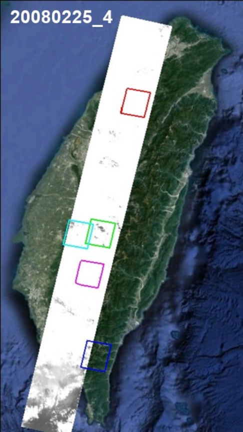
20080225_4

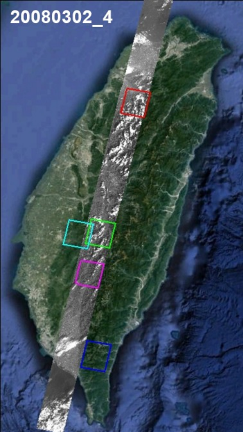
20080302_4

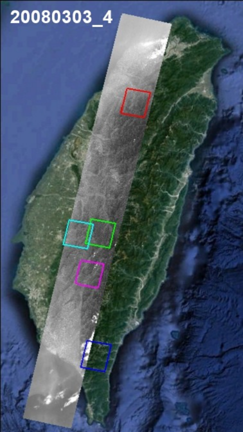
20080303_4

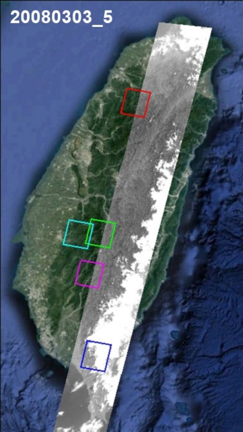
20080303_5

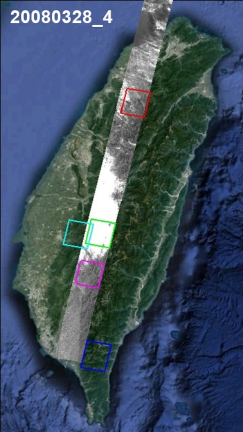
20080328_4

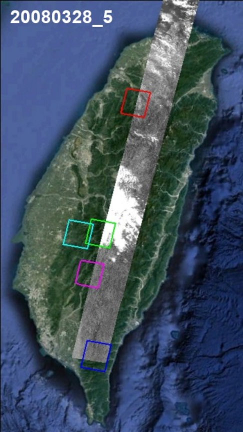
20080328_5

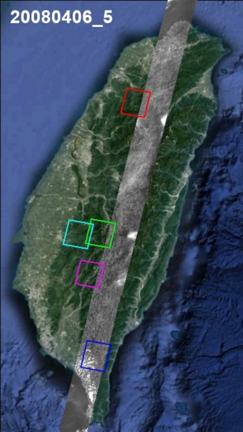
20080406_5

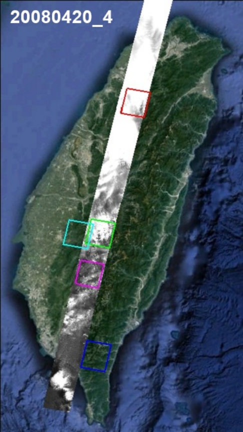
20080420_4

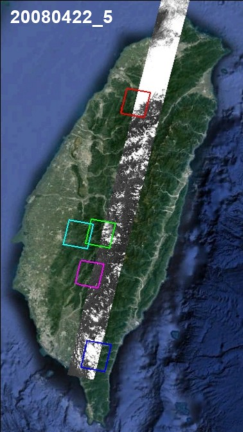
20080422_5

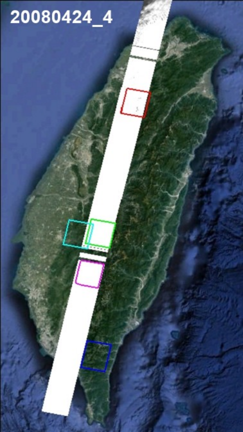
20080424_4

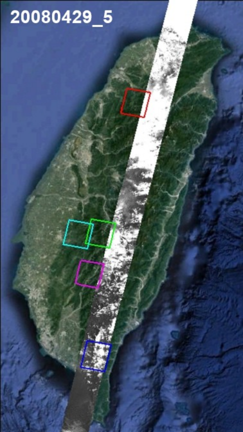
20080429_5

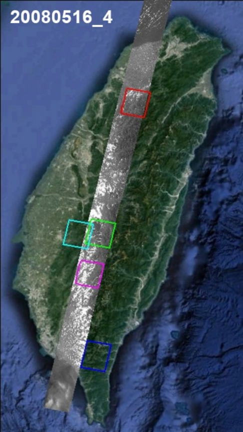
20080516_4

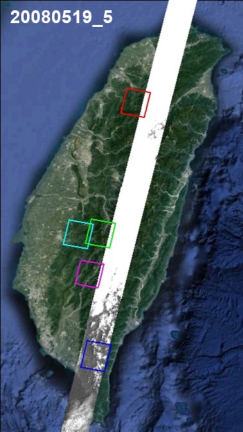
20080519_5

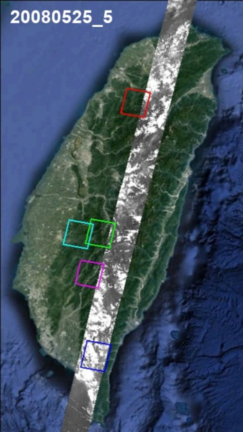
20080525_5

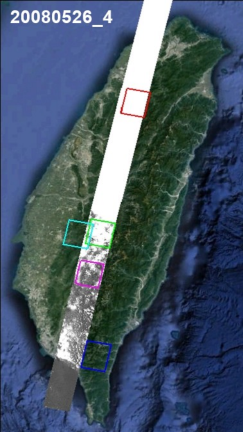
20080526_4

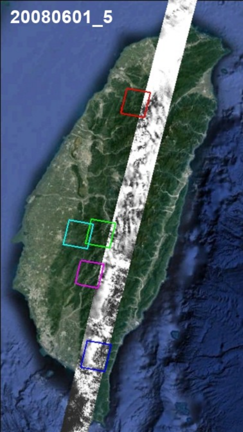
20080601_5

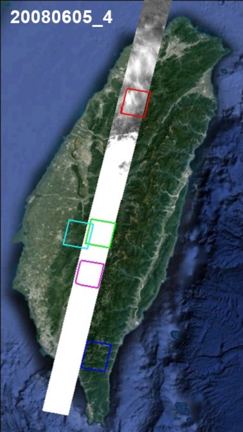
20080605_4

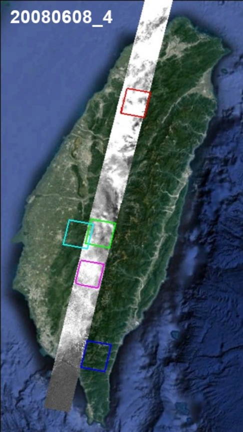
20080608_4

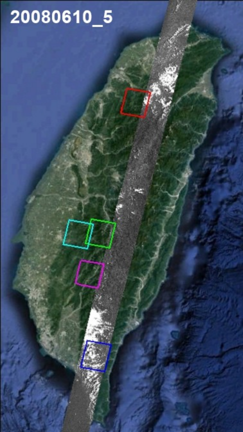
20080610_5

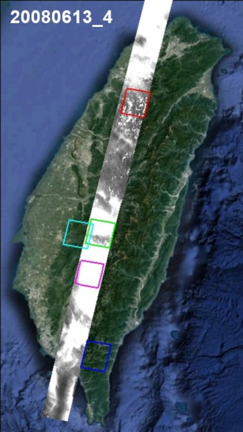
20080613_4

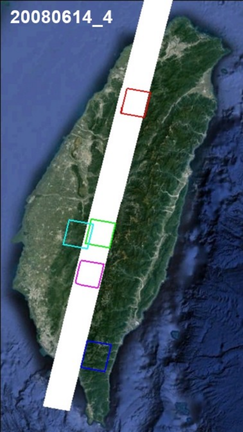
20080614_4

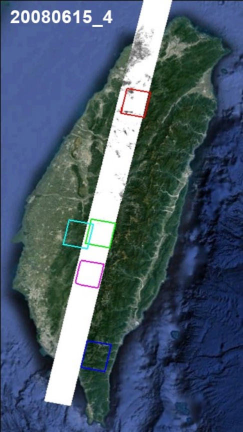
20080615_4

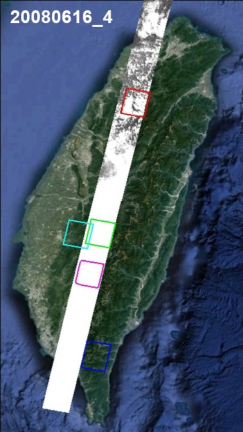
20080616_4

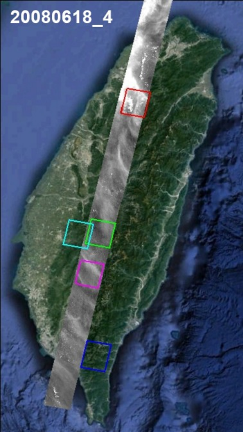
20080618_4

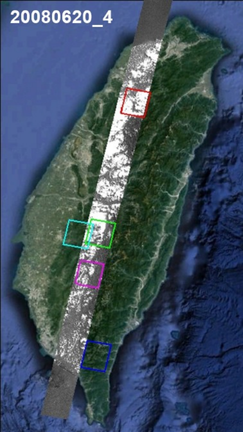
20080620_4

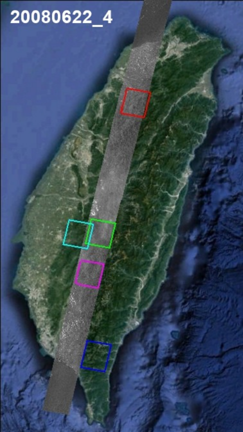
20080622_4

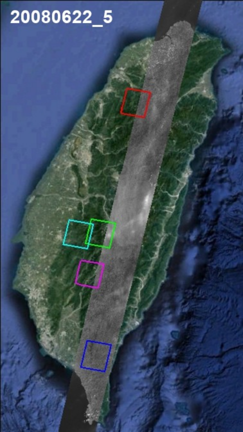
20080622_5

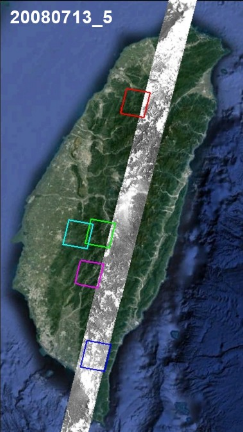
20080713_5

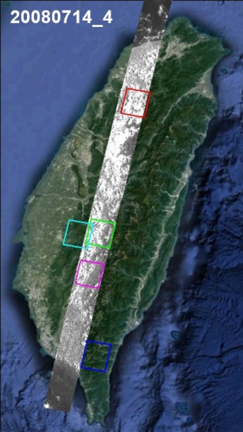
20080714_4

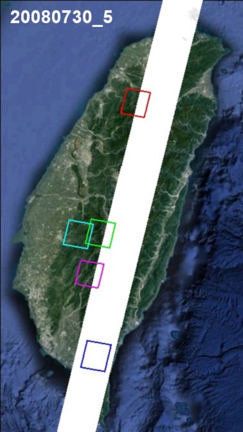
20080730_5

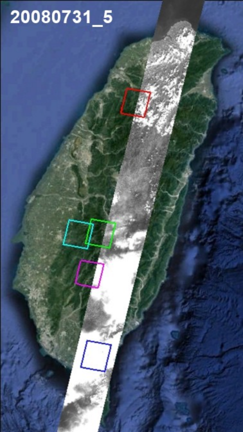
20080731_5

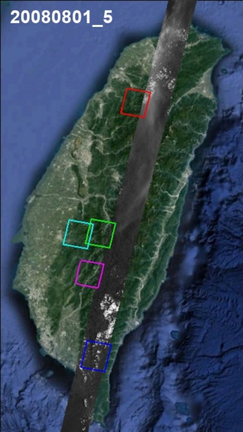
20080801_5

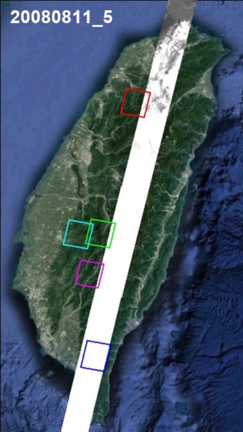
20080811_5

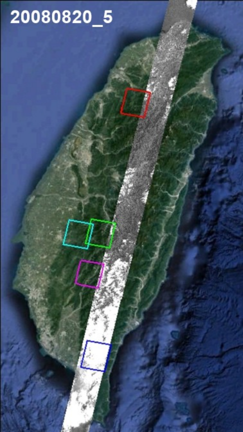
20080820_5

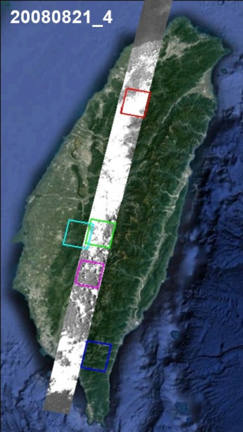
20080821_4

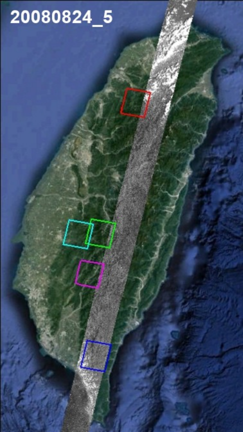
20080824_5

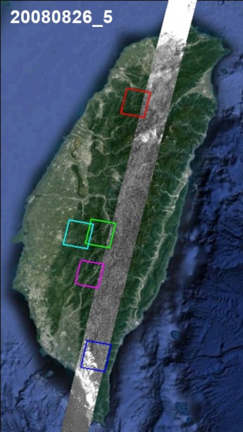
20080826_5

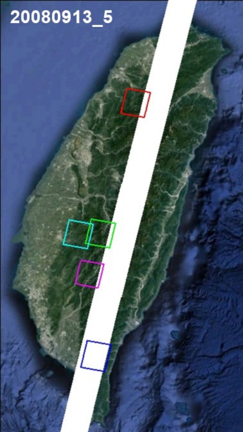
20080913_5

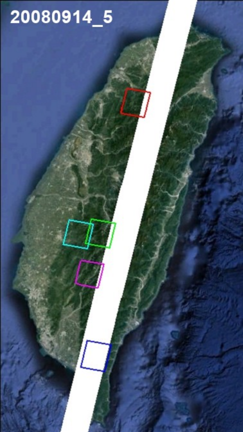
20080914_5

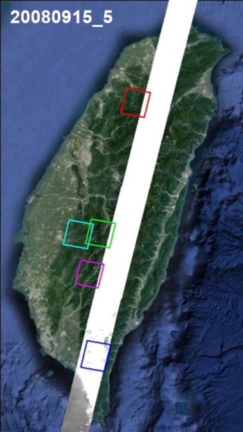
20080915_5

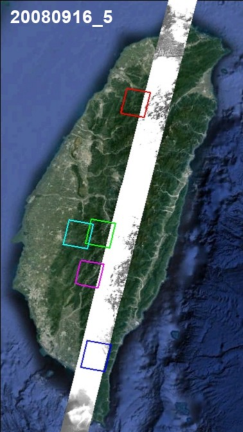

20080916_5

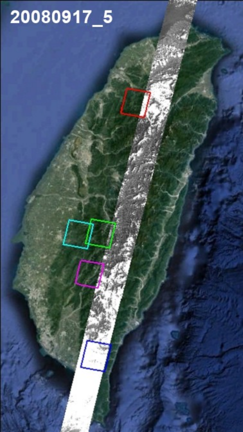
20080917_5

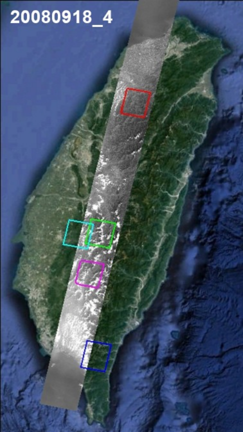
20080918_4

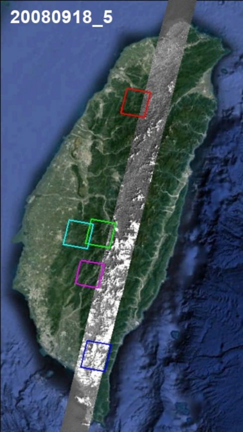
20080918_5

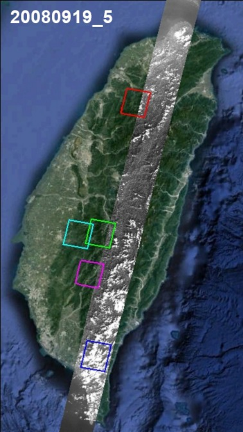
20080919_5

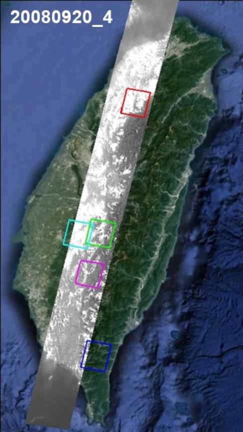
20080920_4

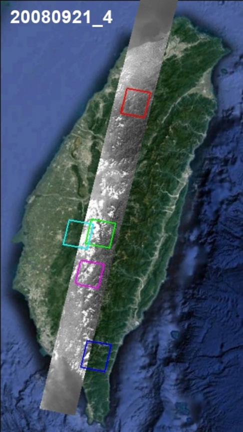
20080921_4

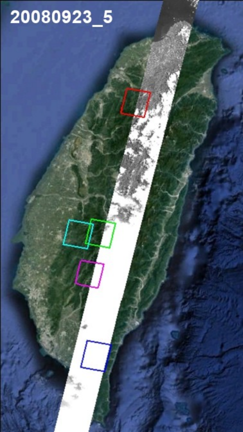
20080923_5

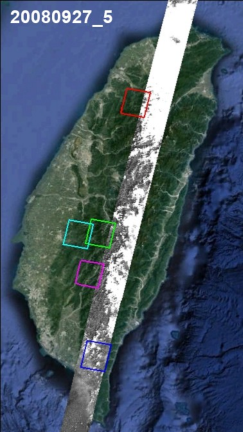
20080927_5

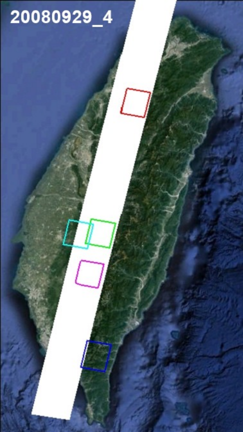
20080929_4

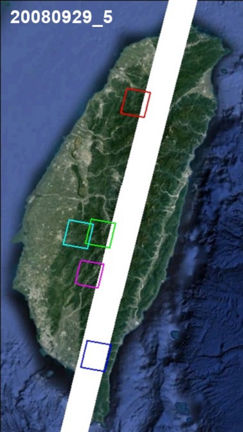
20080929_5

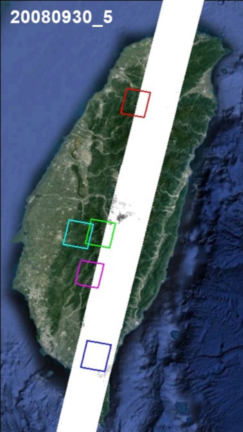
20080930_5

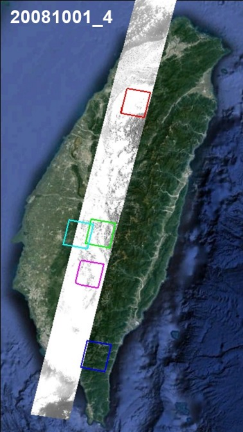
20081001_4

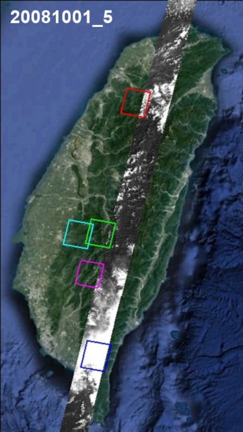
20081001_5

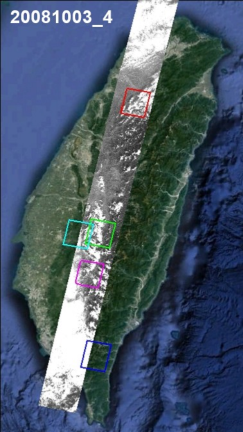
20081003_4

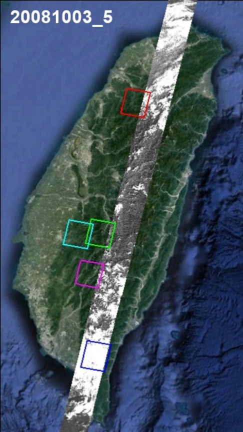
20081003_5

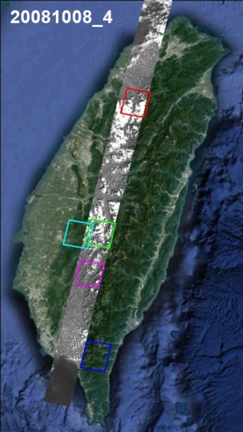
20081008_4

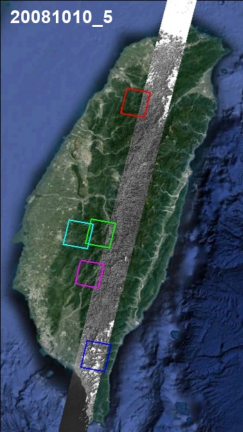
20081010_5

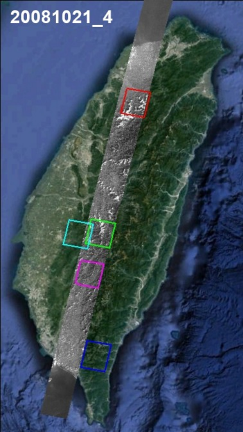
20081021_4

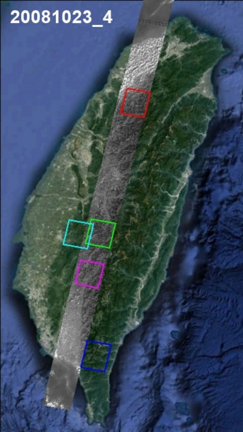
20081023_4

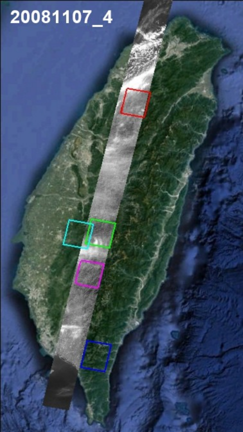
20081107_4

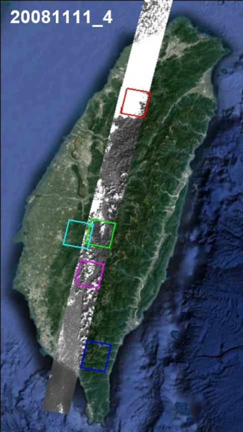
20081111_4

20081113_5

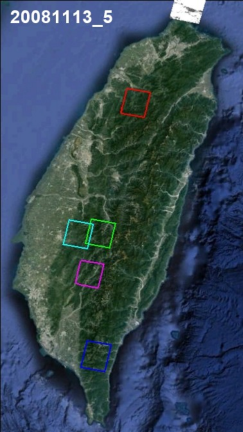

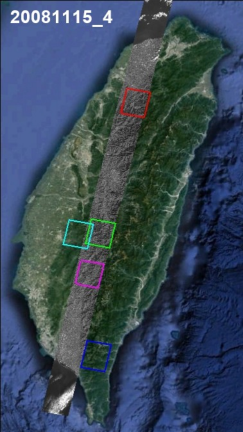
20081115_4

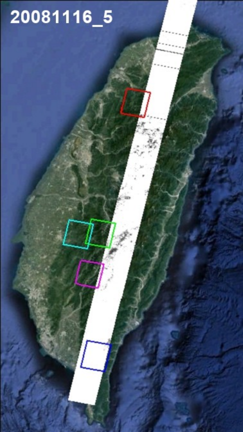
20081116_5

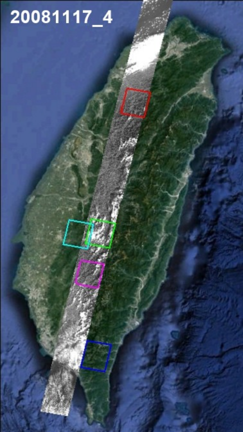
20081117_4

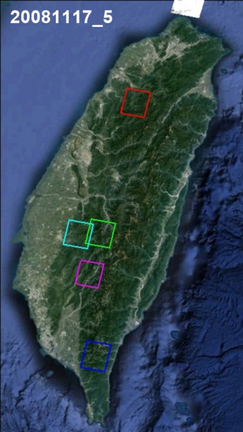
20081117_5

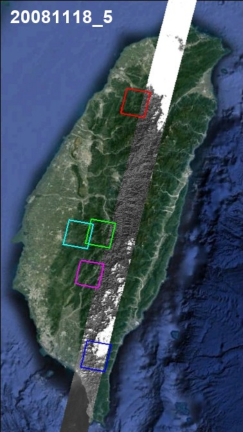
20081118_5

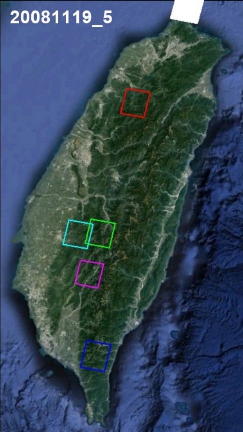
20081119_5

20081122_5

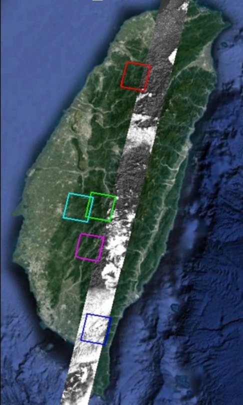

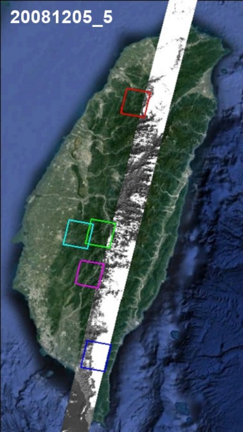
20081205_5

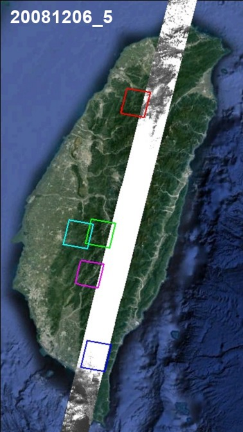
20081206_5

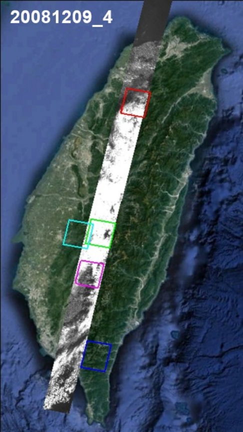
20081209_4

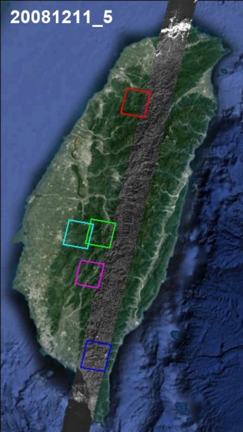
20081211_5

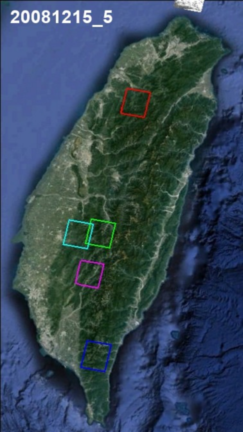
20081215_5

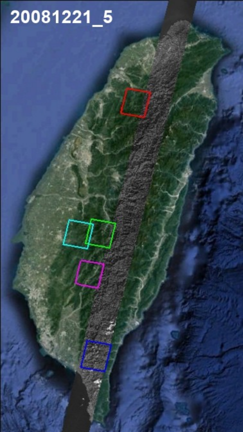
20081221_5

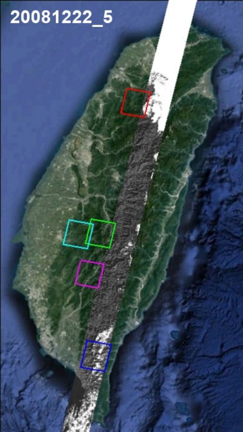
20081222_5

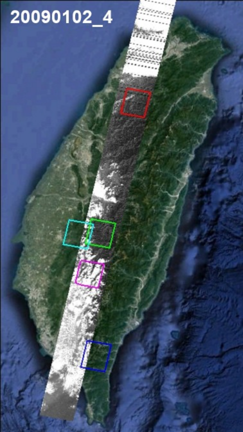
20090102_4

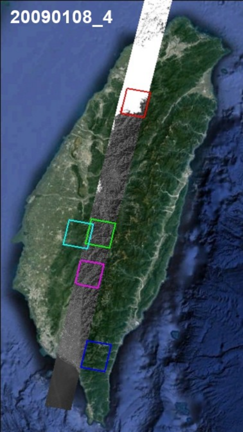
20090108_4

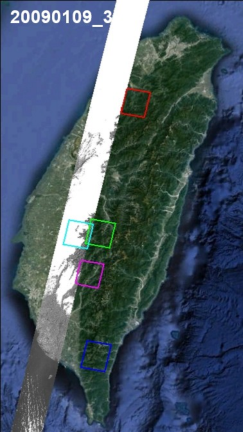
20090109_3

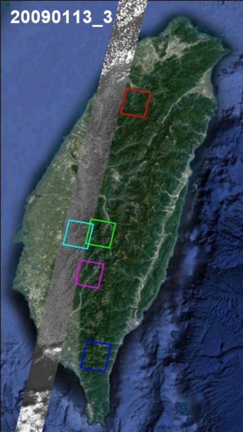
20090113_3

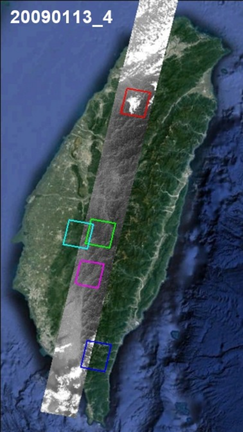
20090113_4

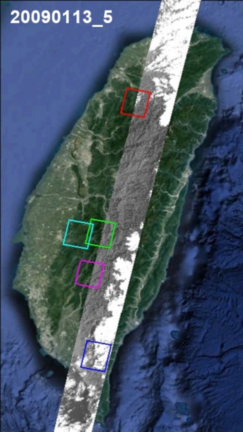
20090113_5

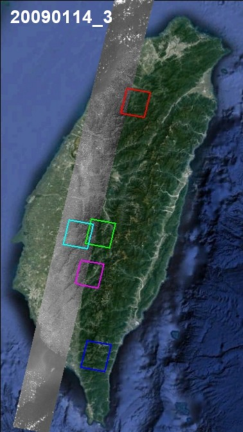
20090114_3

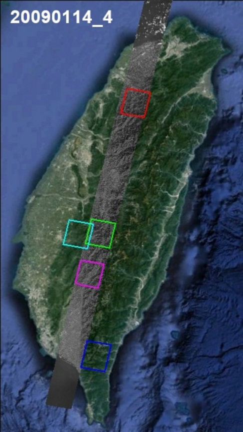
20090114_4

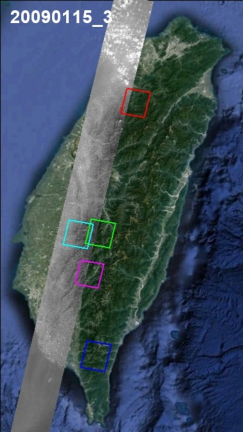
20090115_3

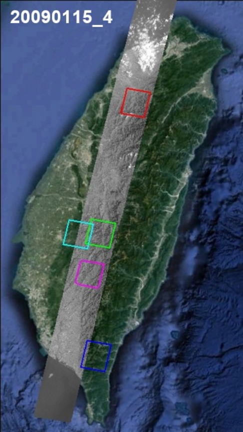
20090115_4

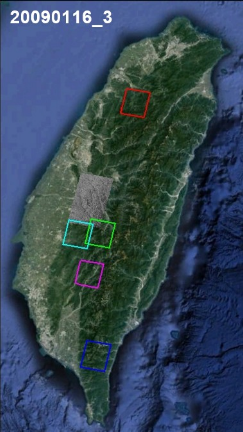
20090116_3

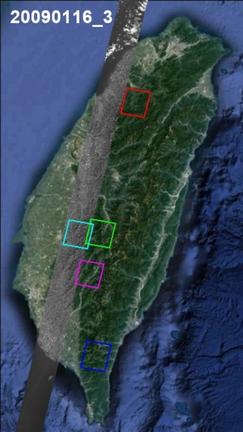
20090116_3

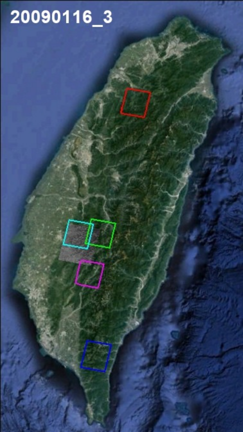
20090116_3

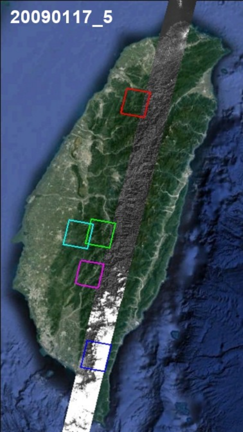
20090117_5

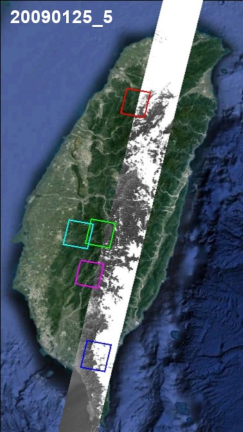
20090125_5

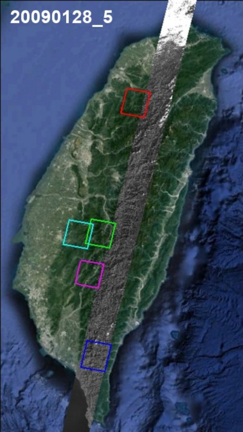
20090128_5

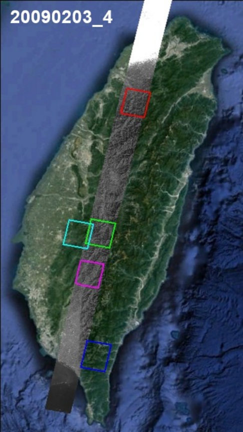
20090203_4

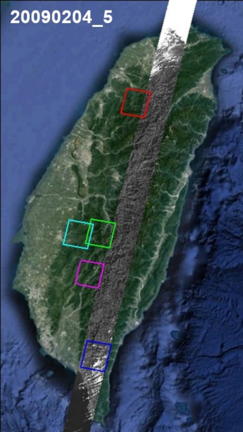
20090204_5

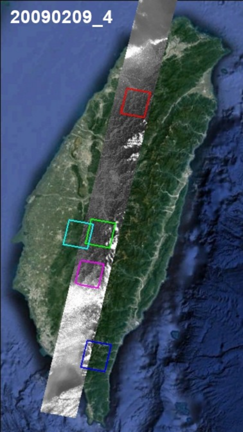
20090209_4

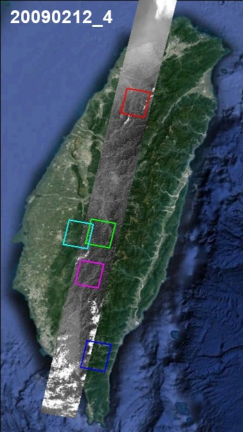
20090212_4

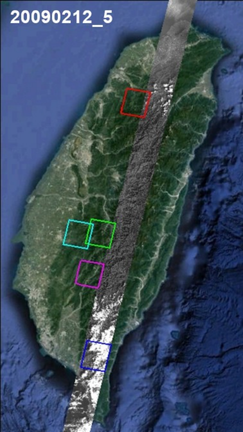
20090212_5

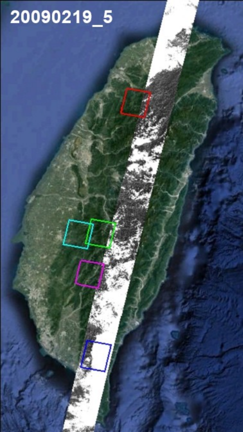
20090219_5

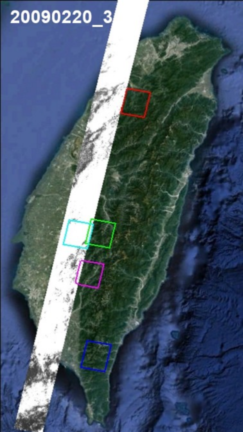
20090220_3

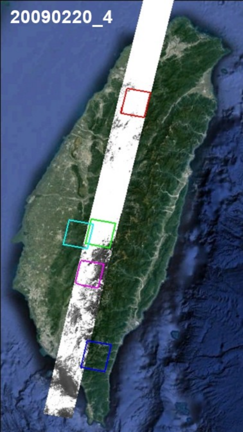
20090220_4

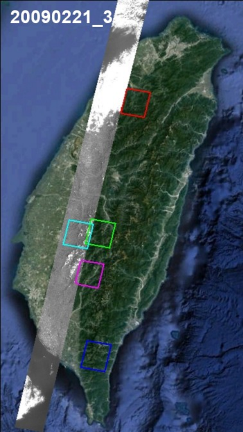
20090221_3

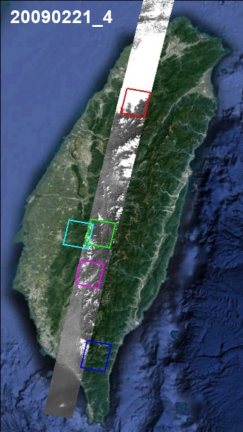
20090221_4

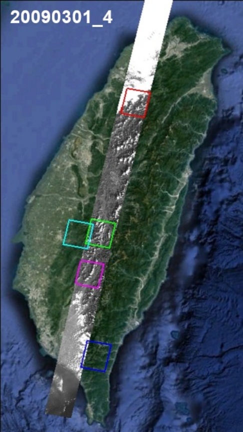
20090301_4

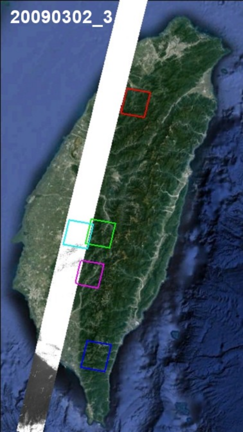
20090302_3

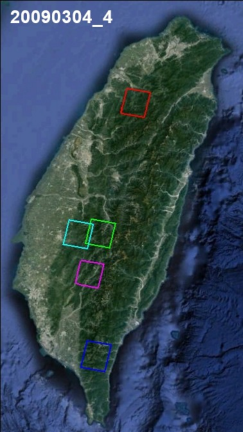
20090304_4

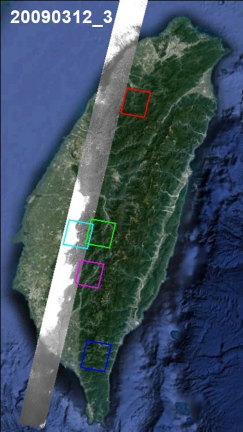
20090312_3

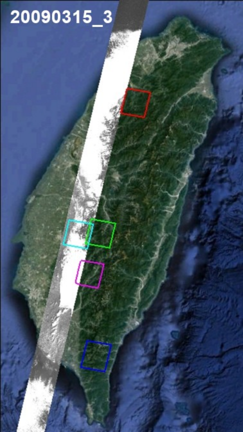
20090315_3

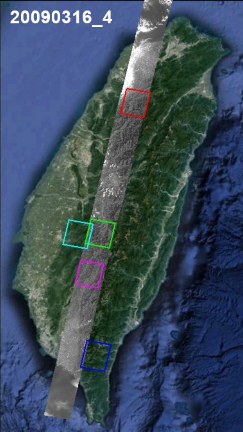
20090316_4

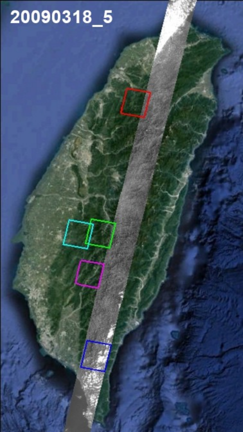
20090318_5

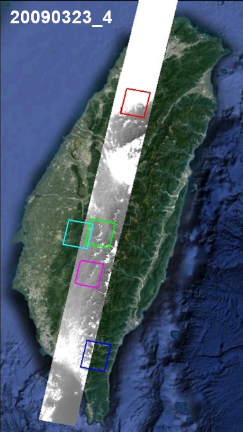
20090323_4

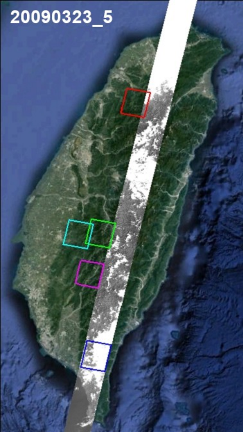
20090323_5

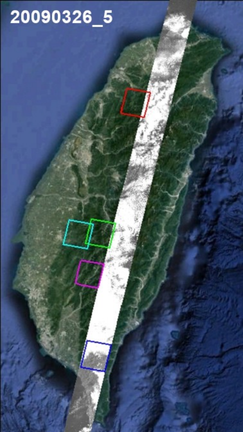
20090326_5

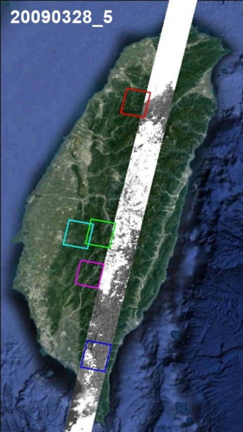
20090328_5

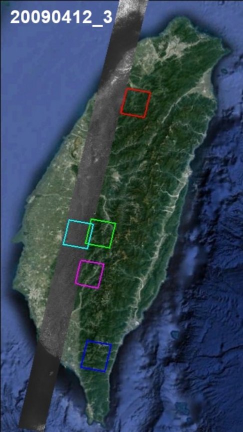
20090412_3

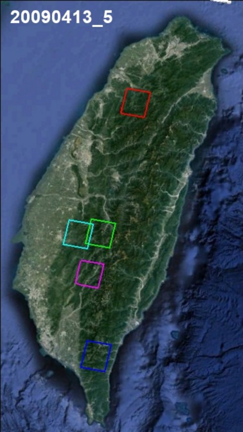
20090413_5

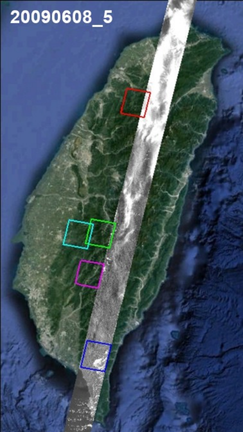
20090608_5

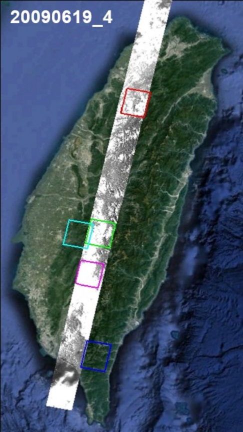
20090619_4

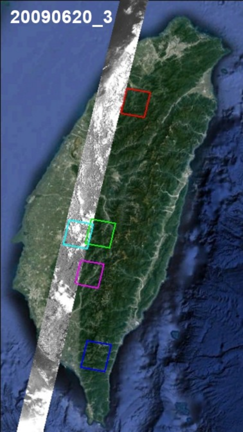
20090620_3

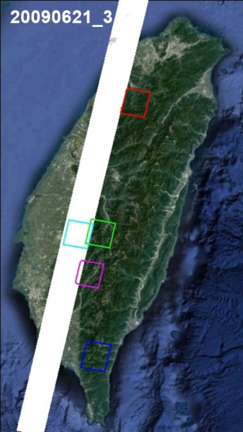
20090621_3

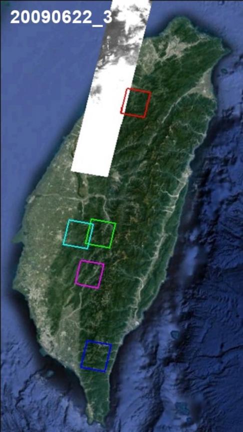
20090622_3

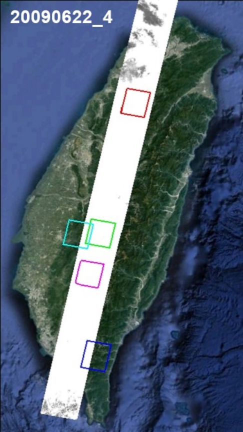
20090622_4

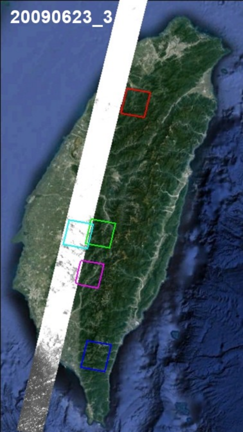
20090623_3

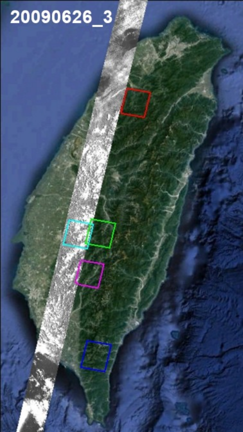
20090626_3

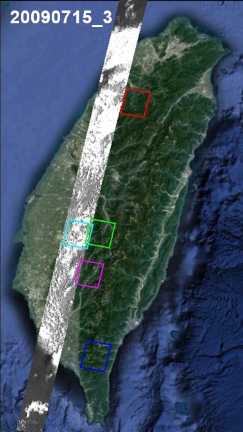
20090715_3

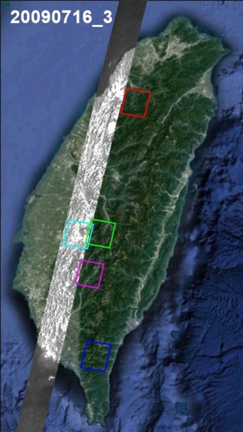
20090716_3

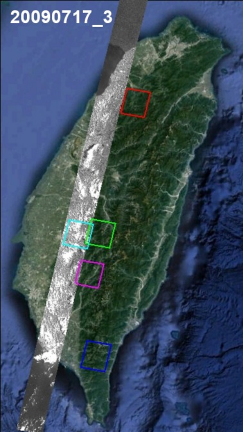
20090717_3

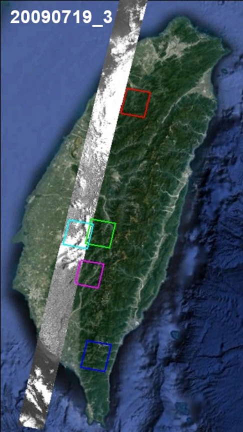
20090719_3

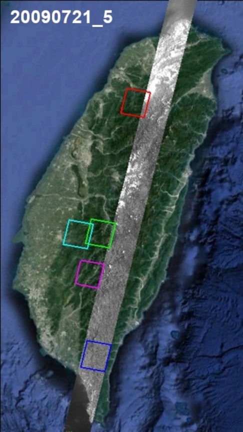
20090721_5

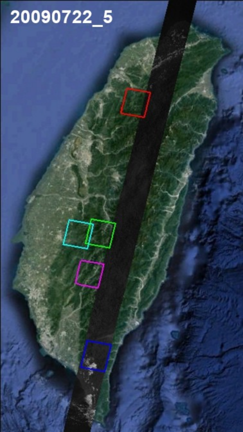
20090722_5

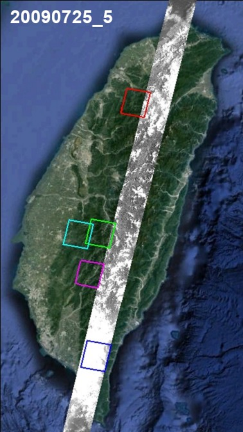
20090725_5

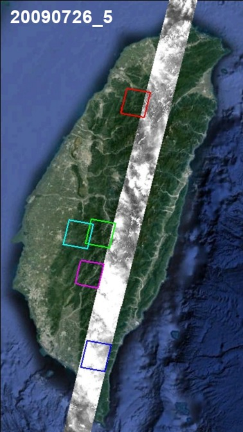
20090726_5

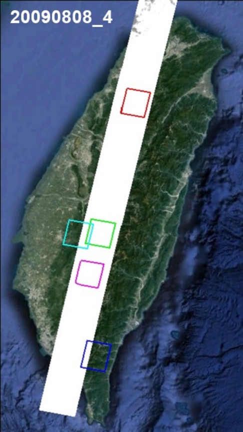
20090808_4

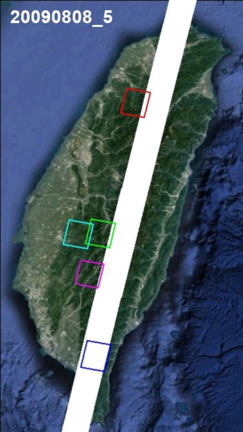
20090808_5

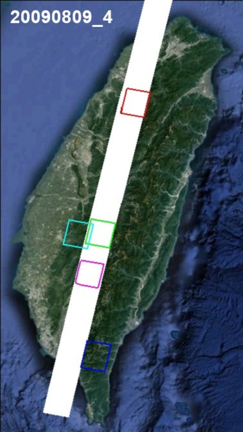
20090809_4

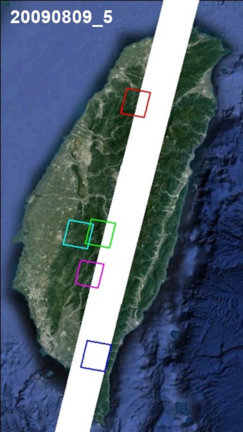
20090809_5

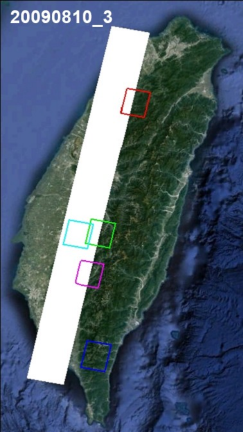
20090810_3

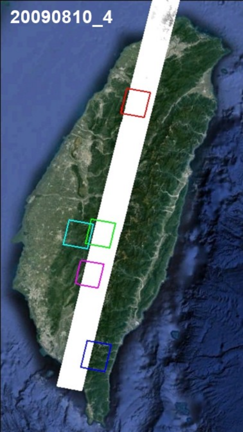
20090810_4

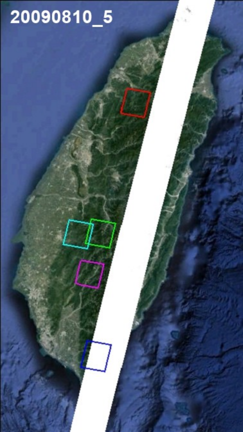
20090810_5

20090811_3

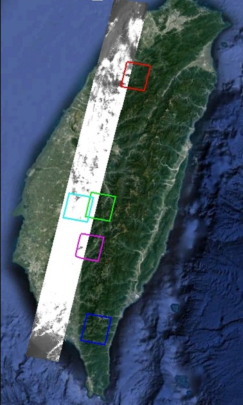

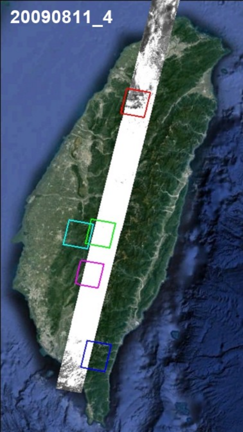
20090811_4

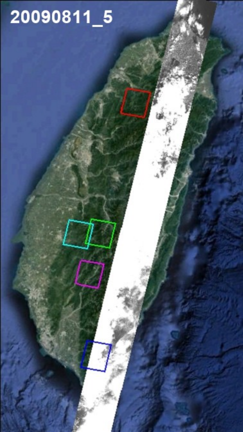
20090811_5

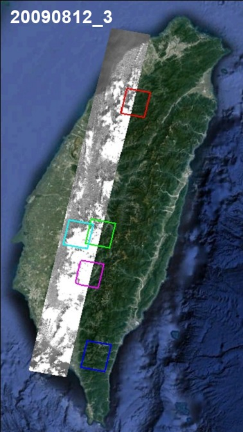
20090812_3

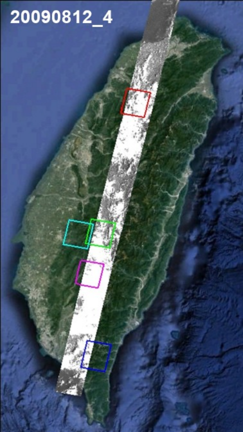
20090812_4

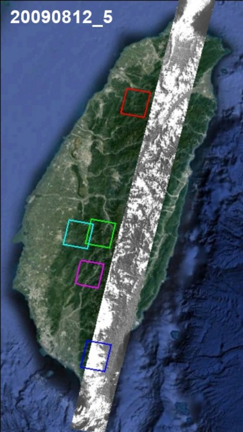
20090812_5

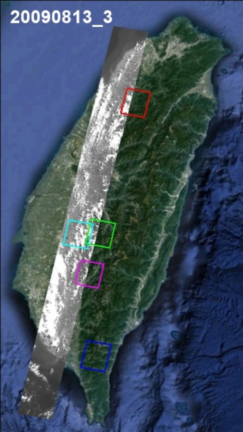
20090813_3

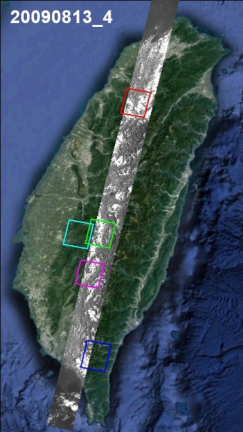
20090813_4

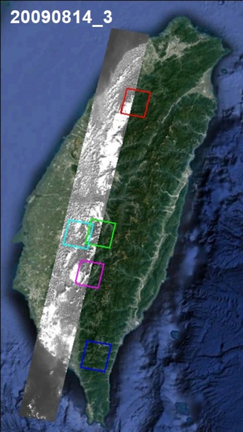
20090814_3

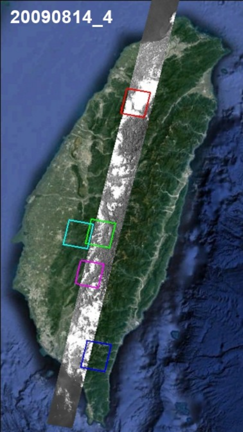
20090814_4

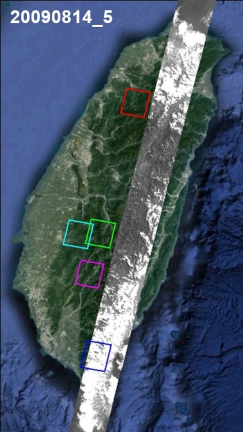
20090814_5

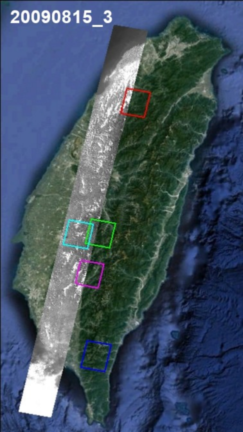
20090815_3

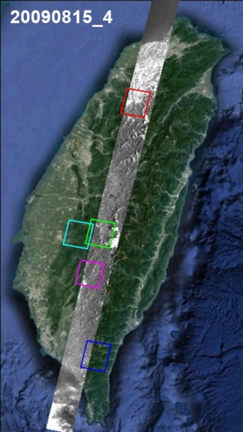
20090815_4

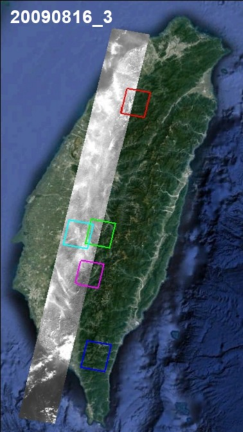
20090816_3

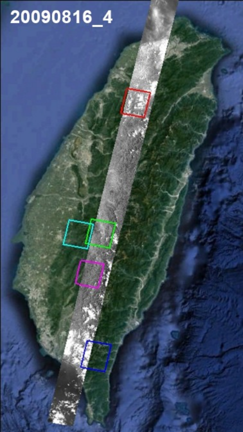
20090816_4

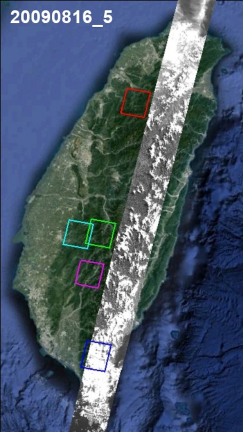
20090816_5

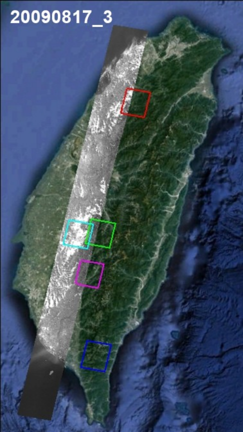
20090817_3

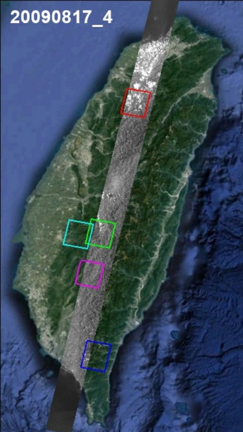
20090817_4

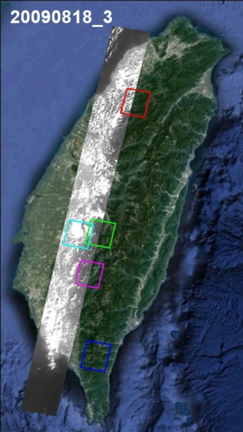
20090818_3

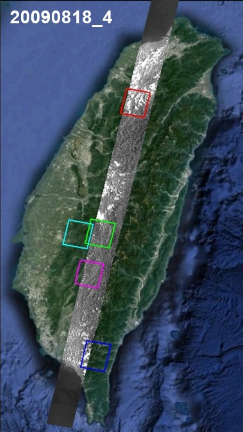
20090818_4

20090818_5

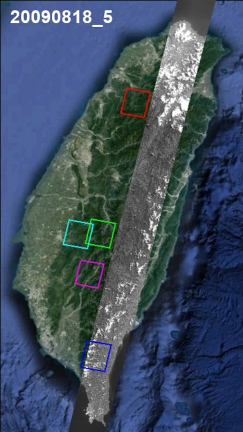

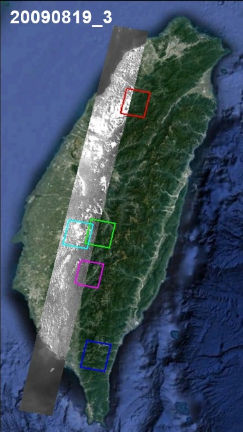
20090819_3

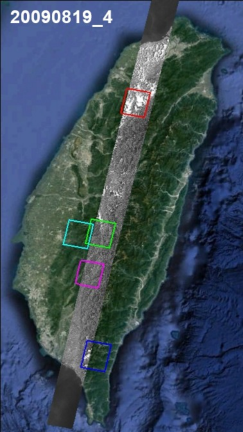
20090819_4

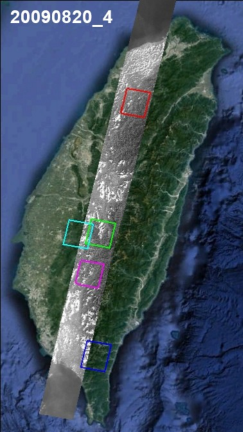
20090820_4

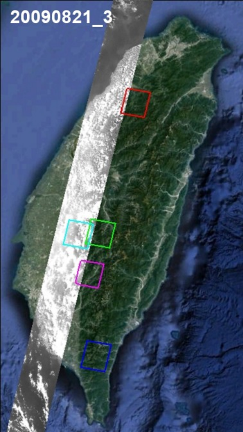
20090821_3

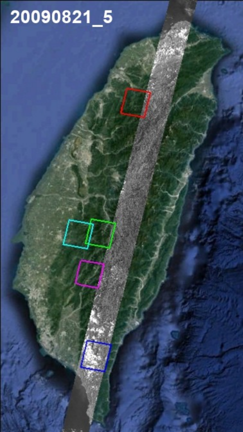
20090821_5

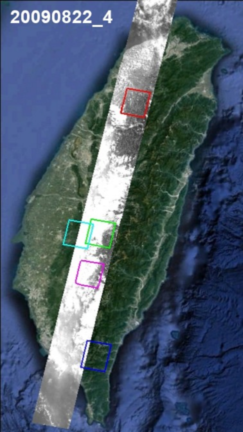
20090822_4

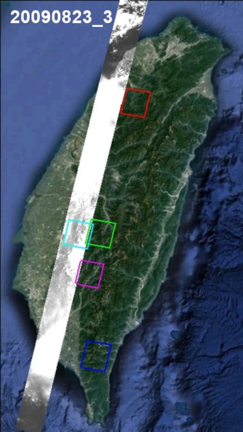
20090823_3

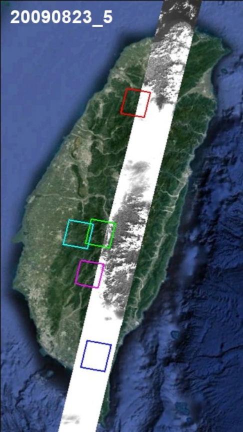
20090823_5

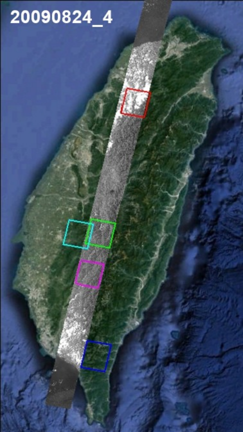
20090824_4

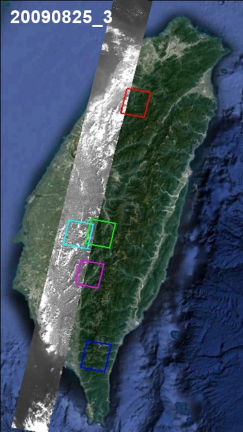
20090825_3

20090825_5

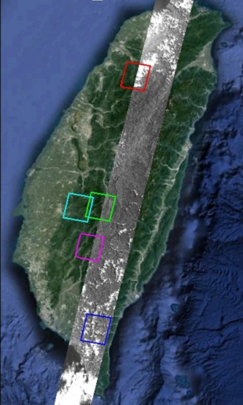

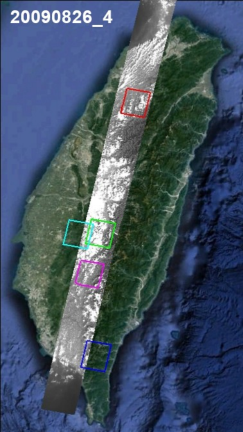
20090826_4

20090827_3

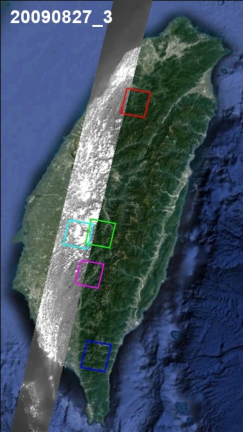

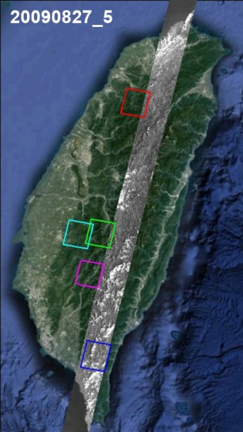
20090827_5

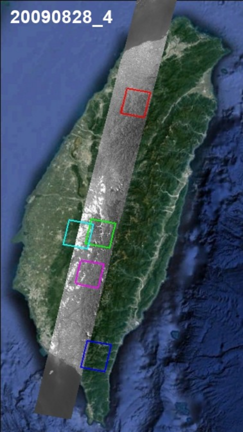
20090828_4

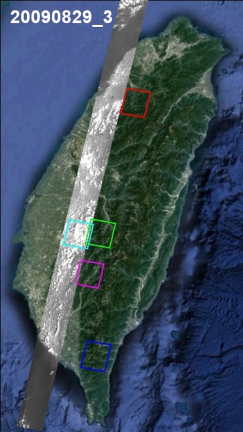
20090829_3

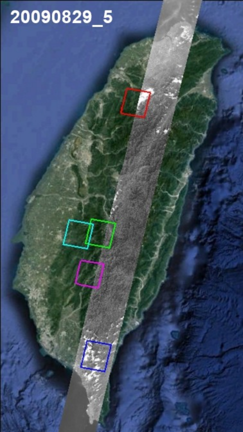
20090829_5

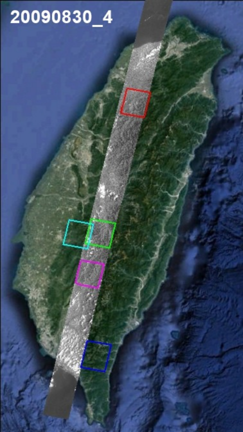
20090830_4

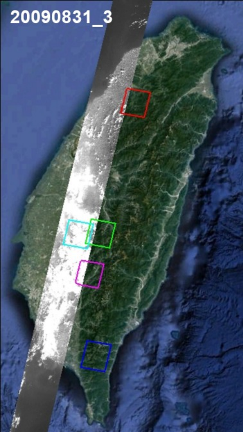
20090831_3

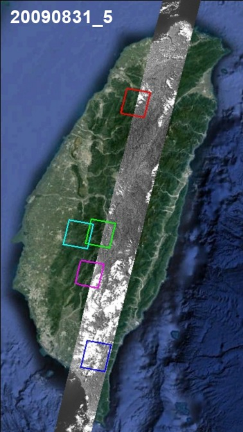
20090831_5

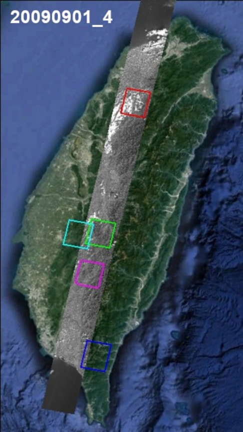
20090901_4

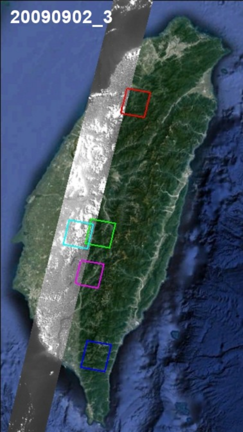
20090902_3

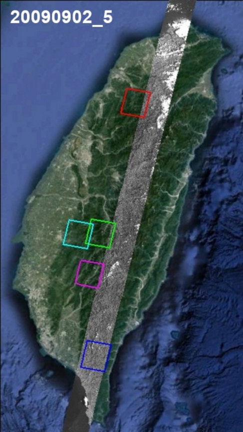
20090902_5

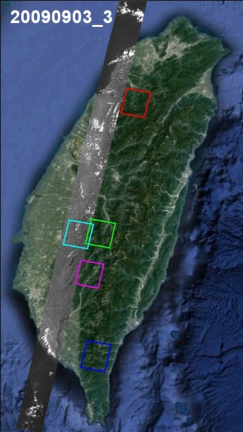
20090903_3

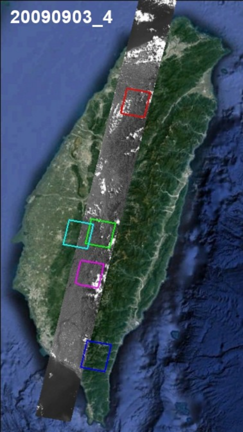
20090903_4

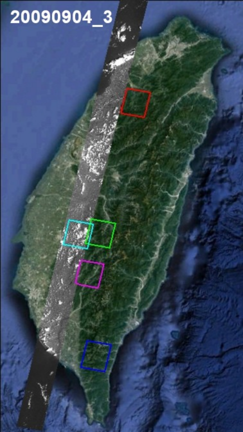
20090904_3

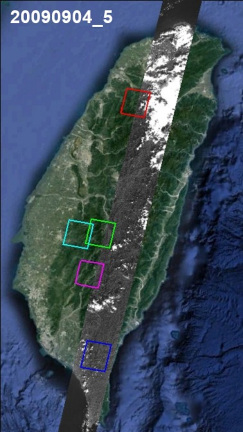
20090904_5

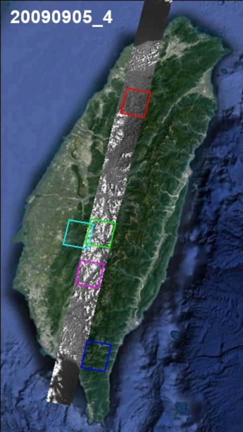
20090905_4

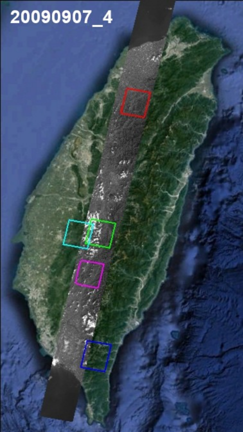
20090907_4

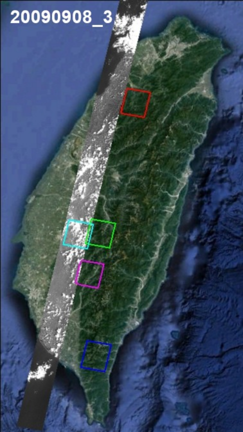
20090908_3

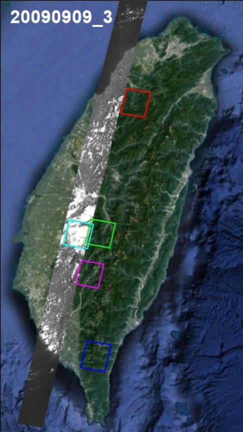
20090909_3

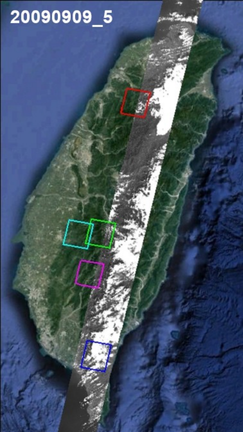
20090909_5

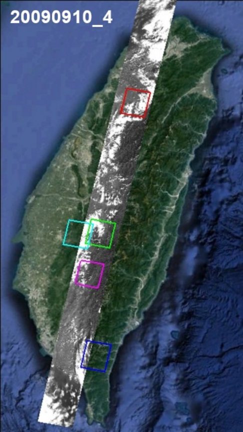
20090910_4

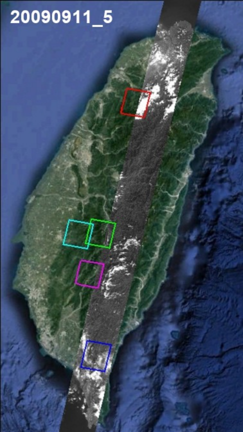
20090911_5

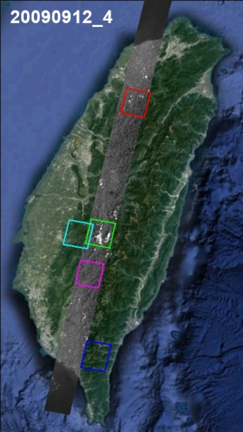
20090912_4

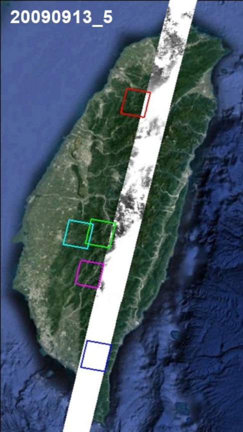
20090913_5

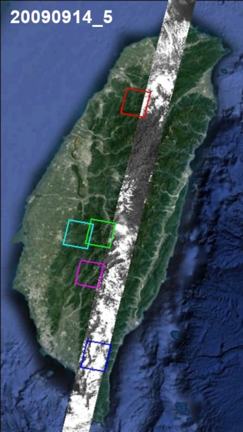
20090914_5

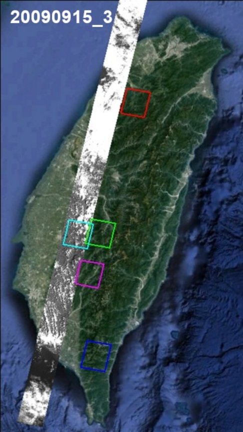
20090915_3

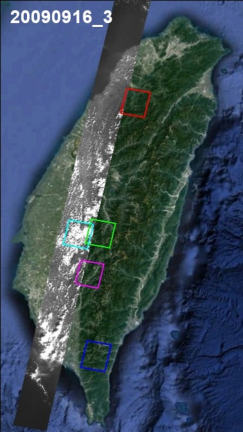
20090916_3

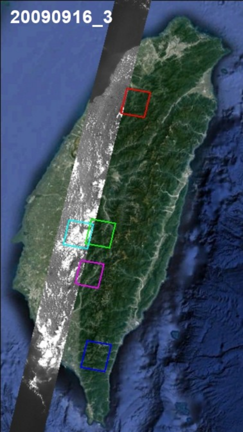
20090916_3

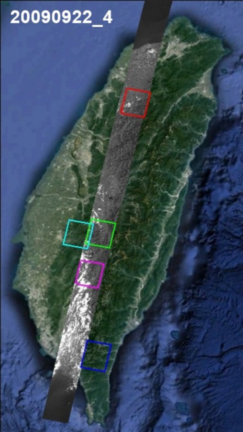
20090922_4

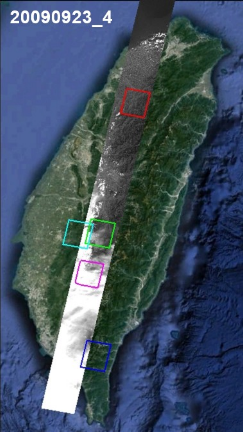
20090923_4

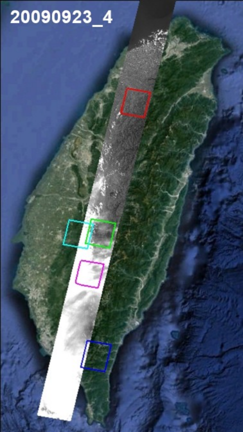
20090923_4

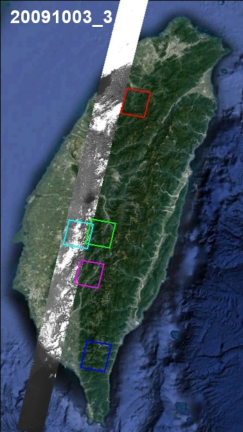
20091003_3

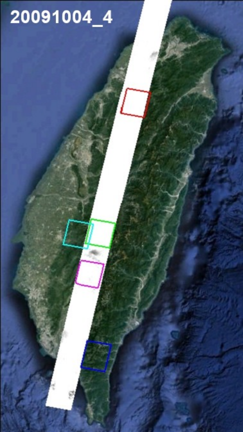
20091004_4

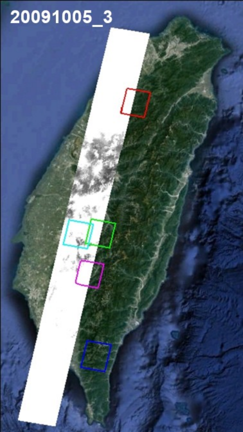
20091005_3

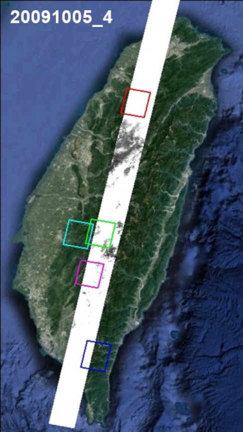
20091005_4

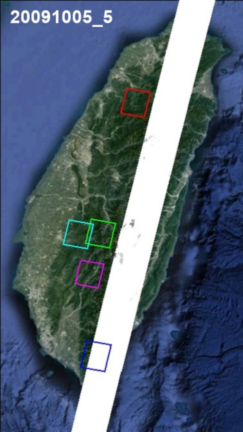
20091005_5

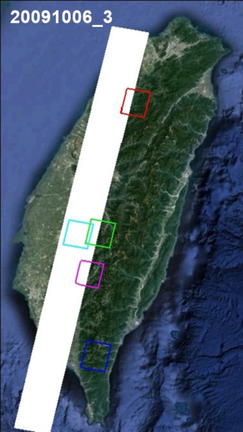
20091006_3

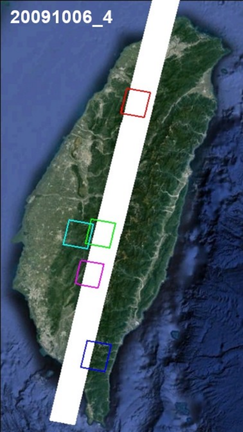
20091006_4

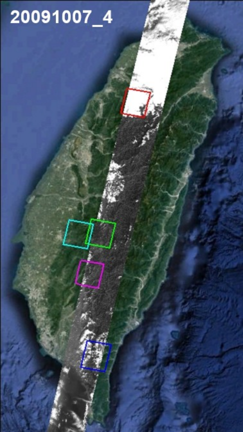
20091007_4

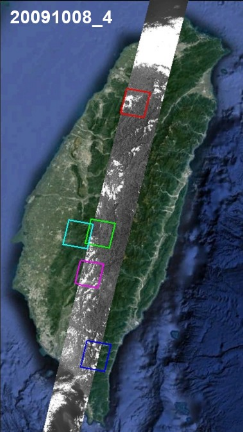
20091008_4

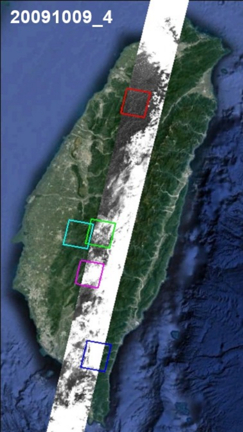
20091009_4

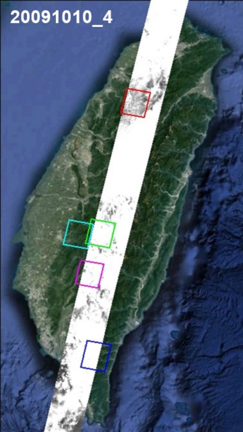
20091010_4

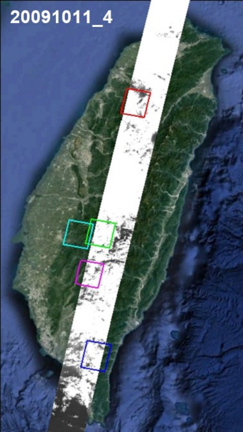
20091011_4

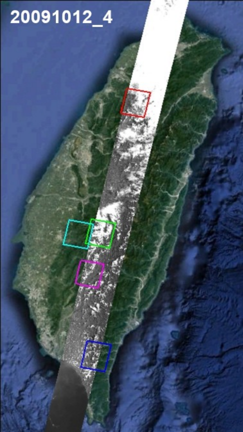
20091012_4

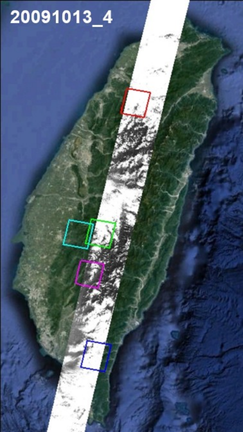
20091013_4

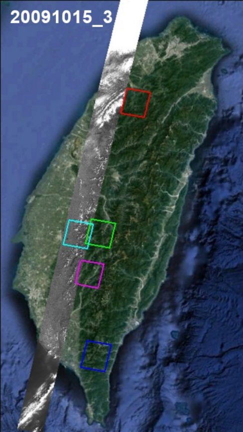
20091015_3

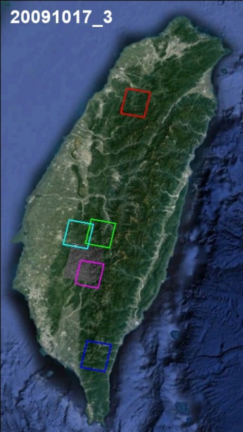
20091017_3

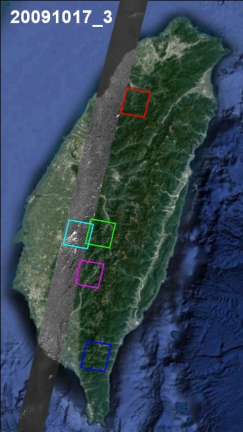
20091017_3

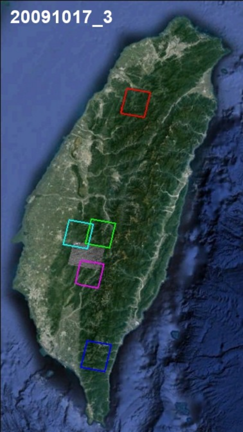
20091017_3

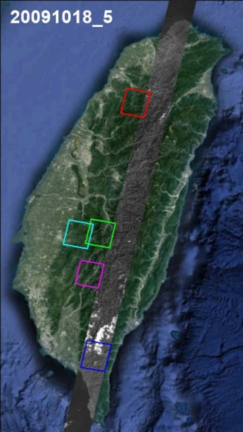
20091018_5

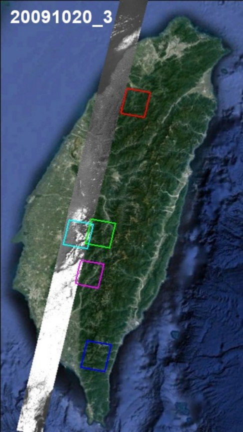
20091020_3

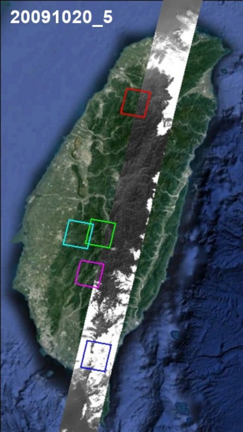
20091020_5

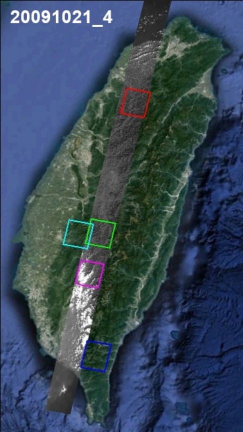
20091021_4

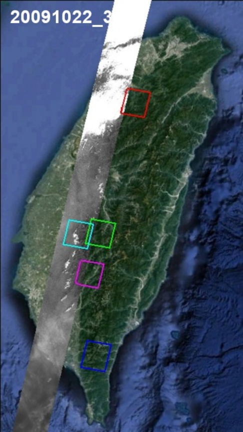
20091022_3

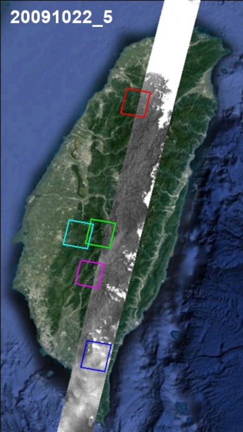
20091022_5

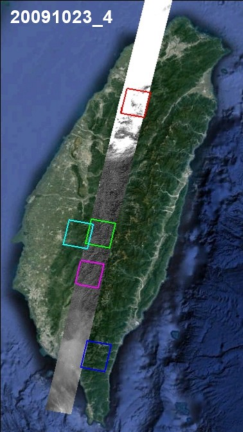
20091023_4

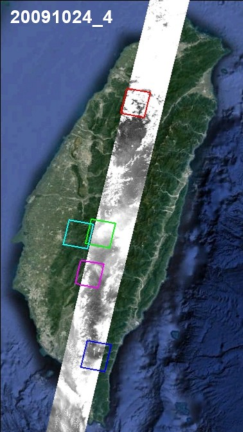
20091024_4

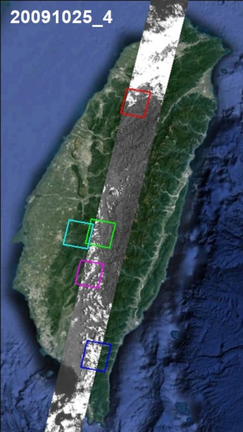
20091025_4

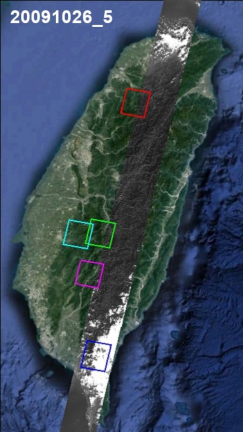
20091026_5

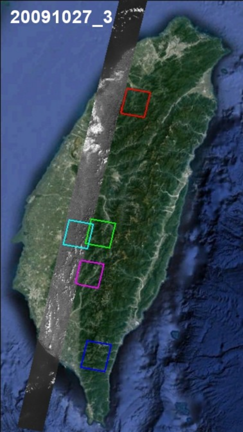
20091027_3

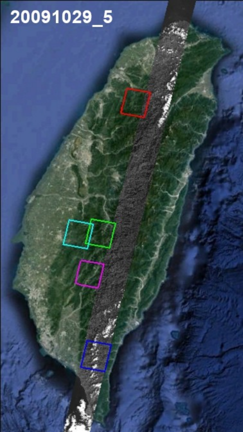
20091029_5

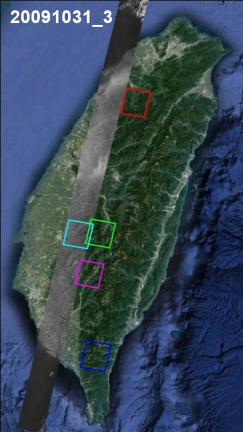
20091031_3

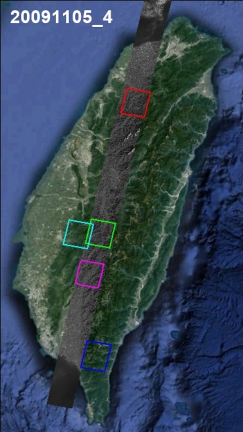
20091105_4

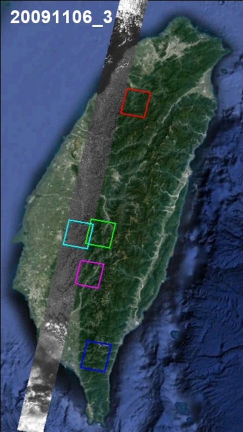
20091106_3

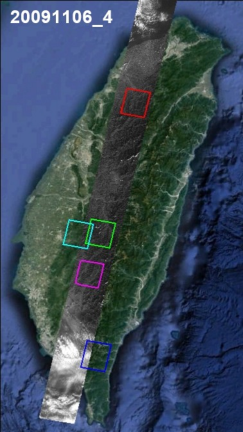
20091106_4

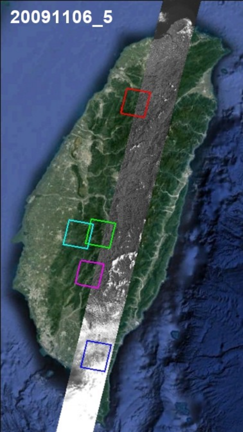
20091106_5

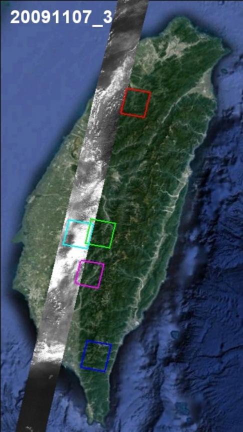
20091107_3

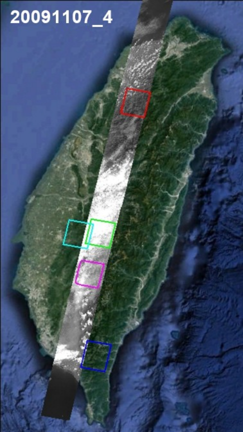
20091107_4

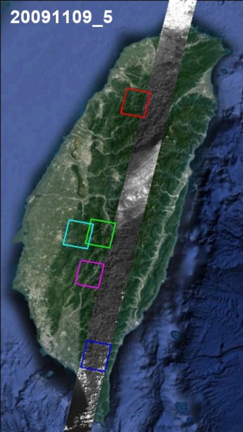
20091109_5

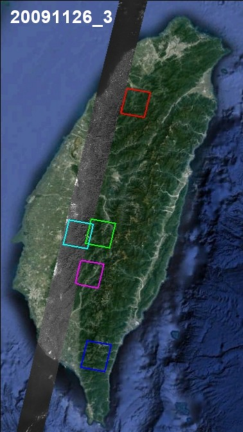
20091126_3

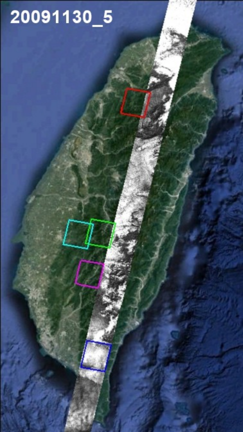
20091130_5

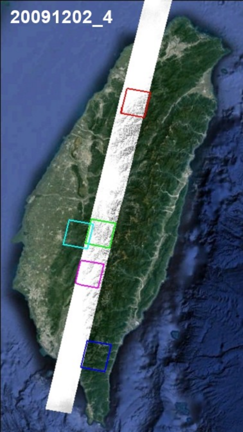
20091202_4

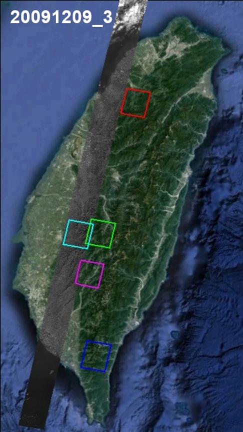
20091209_3

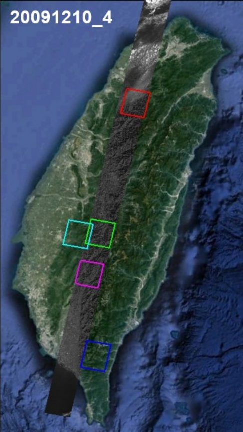
20091210_4

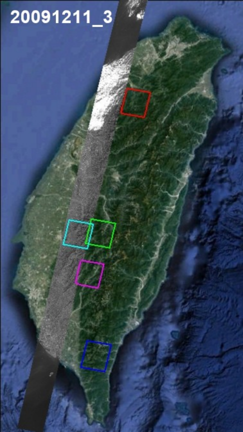
20091211_3

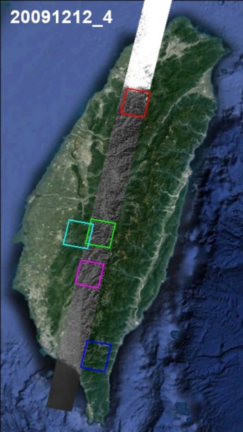
20091212_4

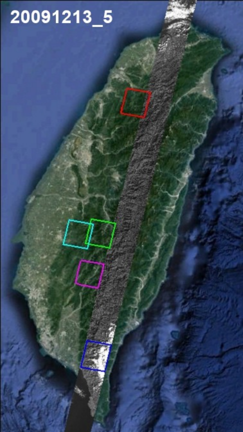
20091213_5

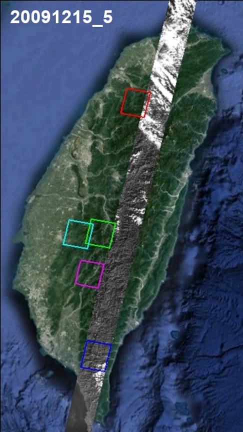
20091215_5

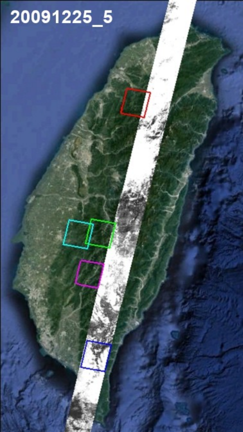
20091225_5

20091228_5

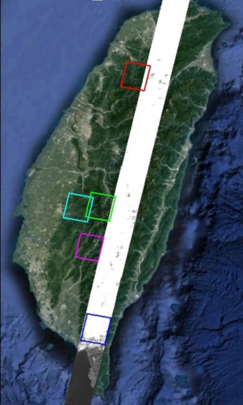

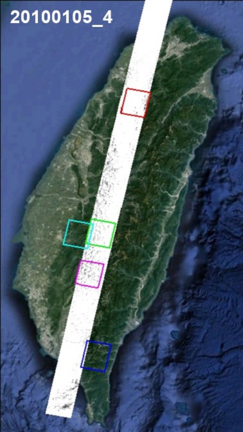
20100105_4

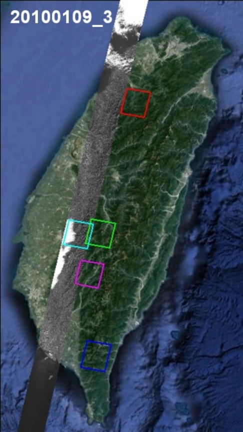
20100109_3

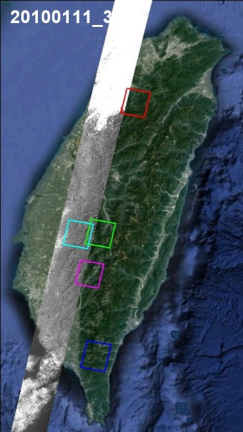
20100111_3

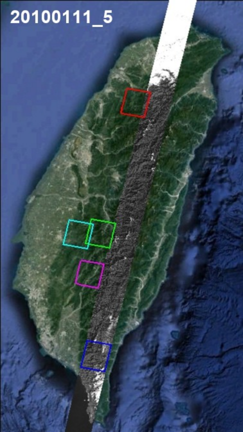
20100111_5

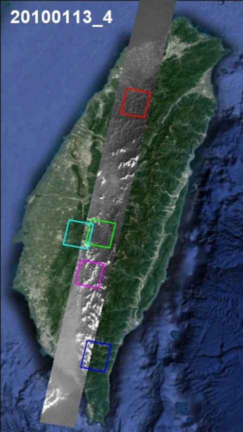
20100113_4

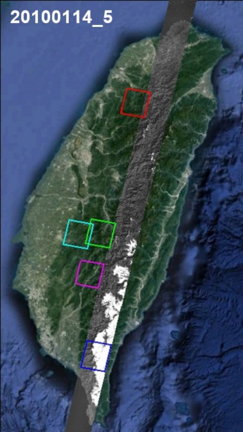
20100114_5

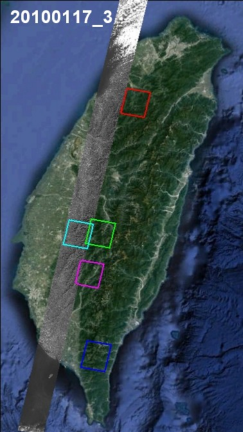
20100117_3

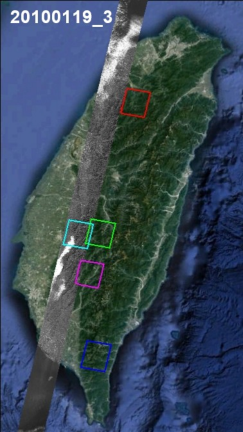
20100119_3

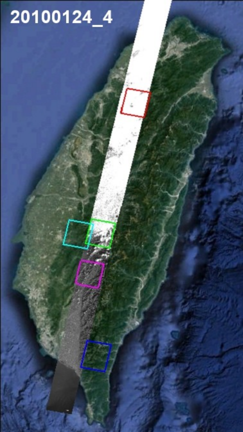
20100124_4

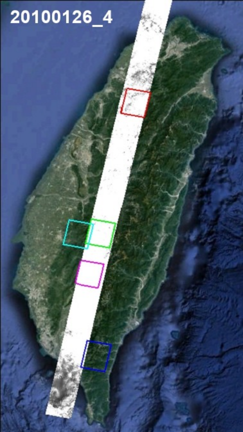
20100126_4

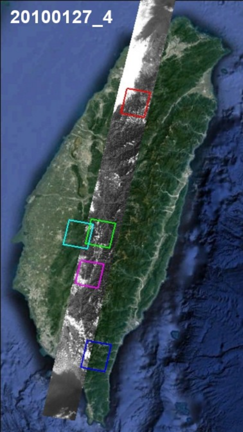
20100127_4

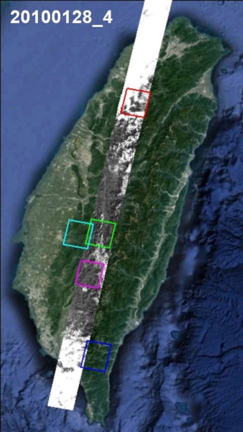
20100128_4

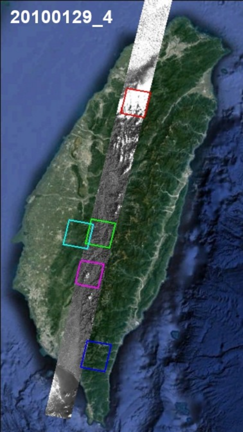
20100129_4

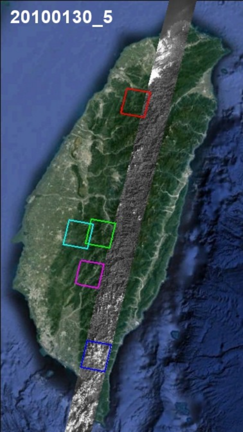
20100130_5

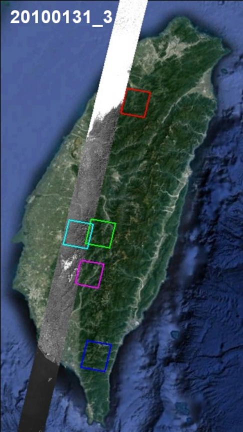
20100131_3

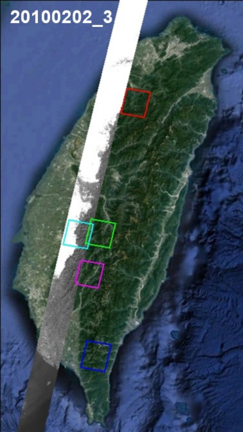
20100202_3

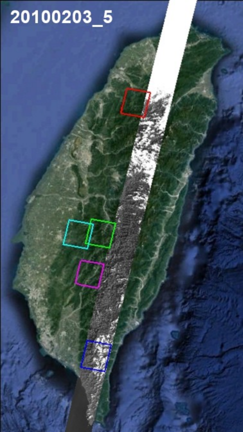
20100203_5

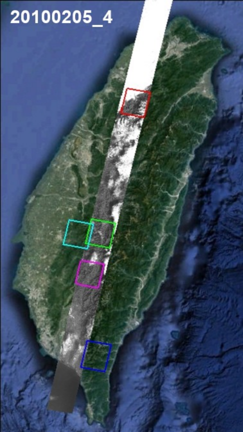
20100205_4

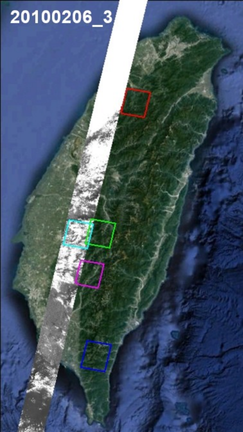
20100206_3

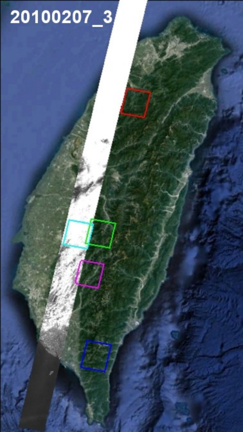
20100207_3

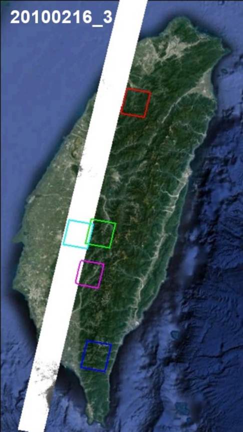
20100216_3

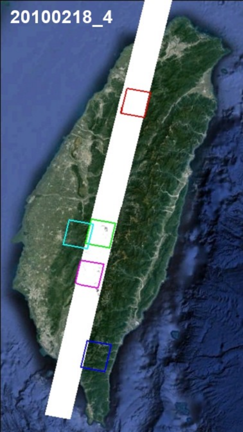
20100218_4

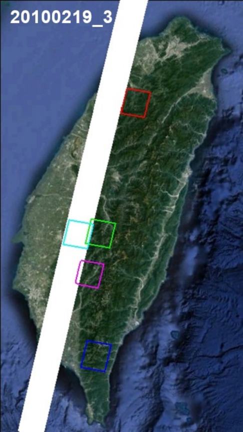
20100219_3

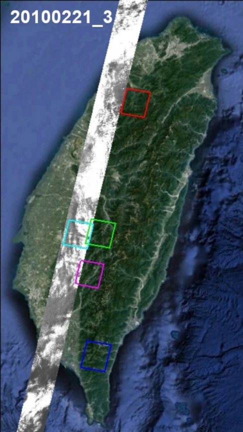
20100221_3

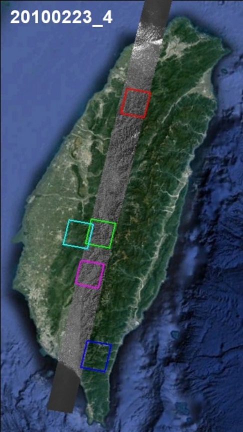
20100223_4

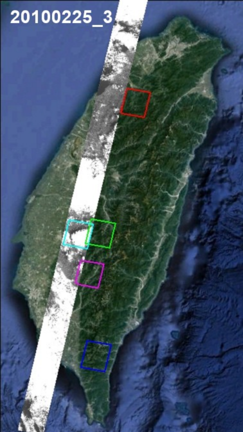
20100225_3

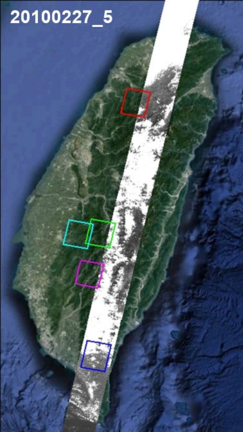
20100227_5

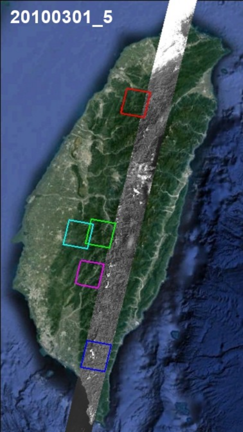
20100301_5

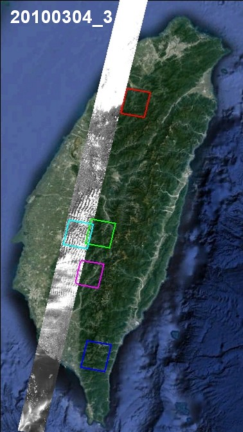
20100304_3

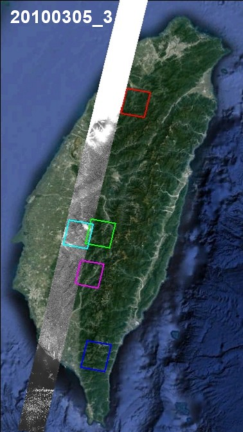
20100305_3

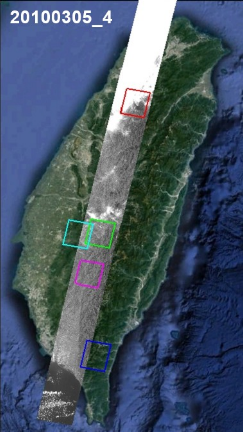
20100305_4

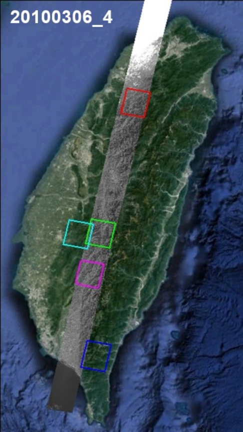
20100306_4

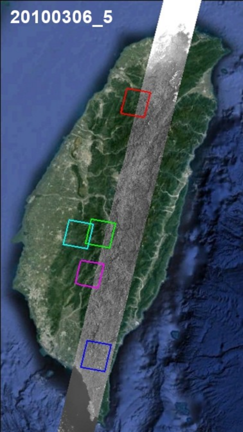
20100306_5

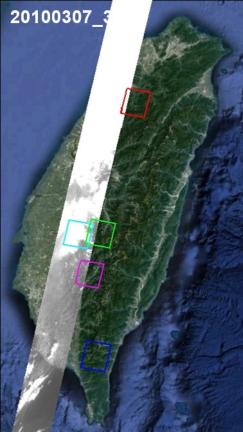
20100307_3

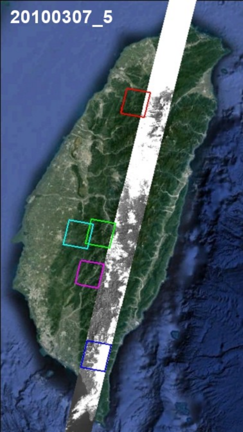
20100307_5

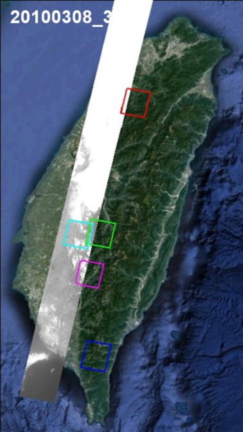
20100308_3

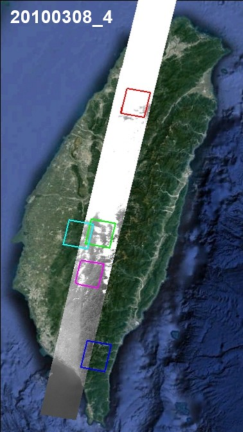
20100308_4

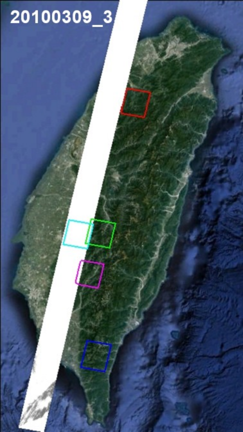
20100309_3

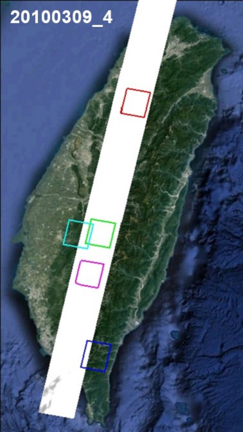
20100309_4

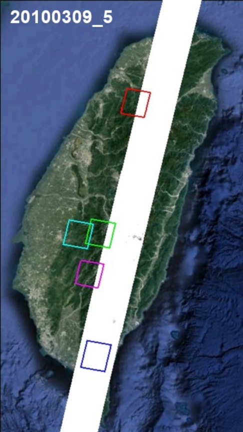
20100309_5

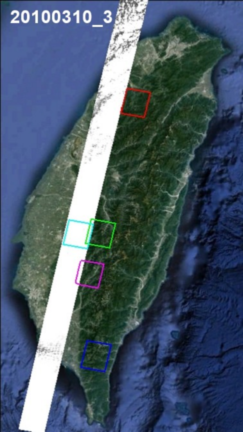
20100310_3

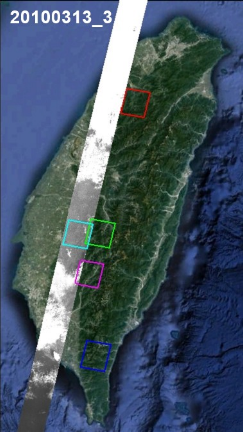
20100313_3

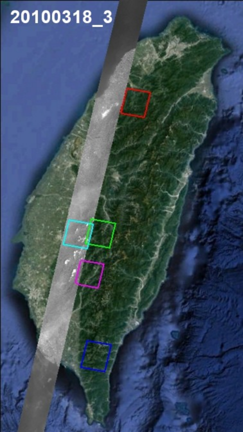
20100318_3

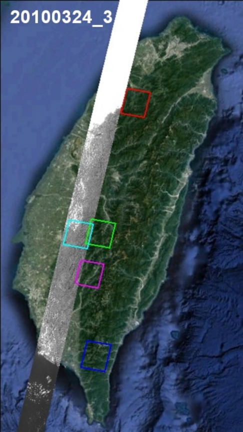
20100324_3

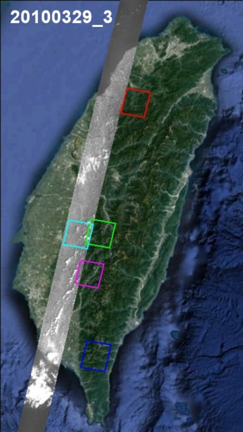
20100329_3

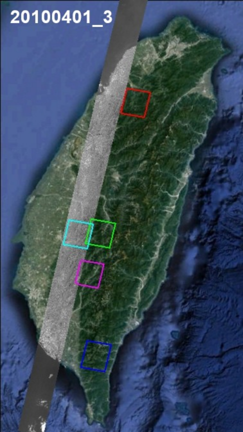
20100401_3

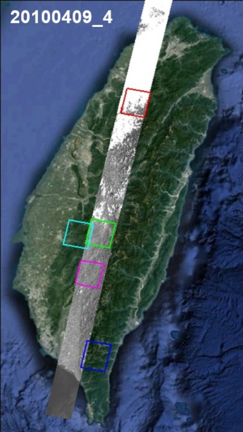
20100409_4

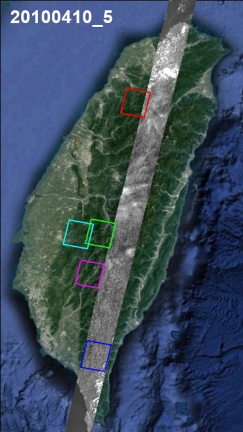
20100410_5

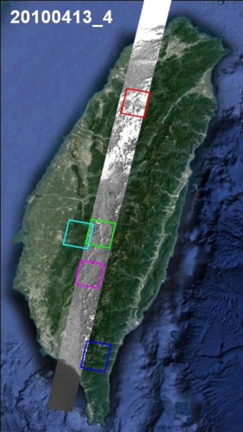
20100413_4

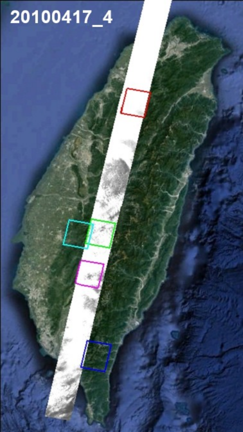
20100417_4

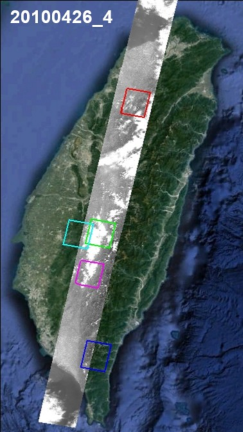
20100426_4

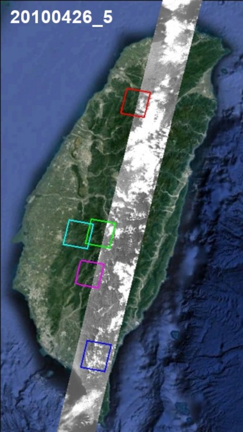
20100426_5

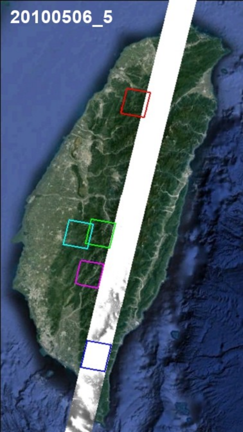
20100506_5

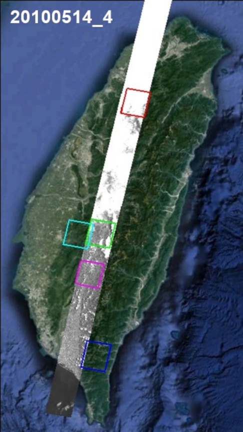
20100514_4

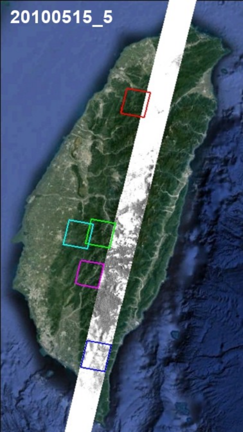
20100515_5

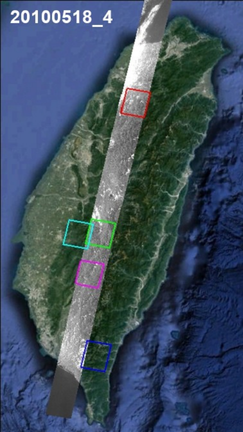
20100518_4

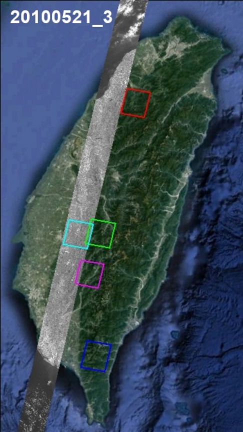
20100521_3

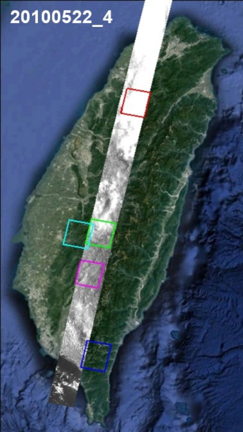
20100522_4

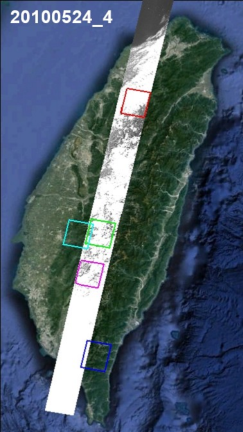
20100524_4

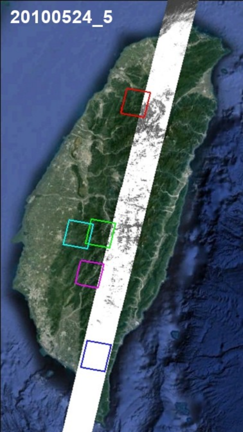
20100524_5

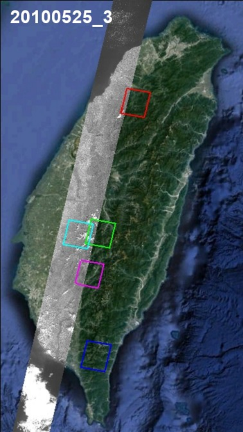
20100525_3

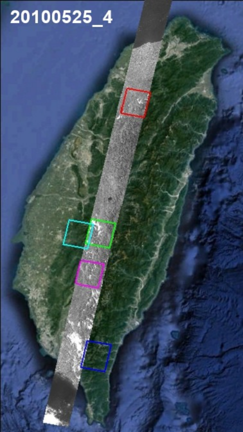
20100525_4

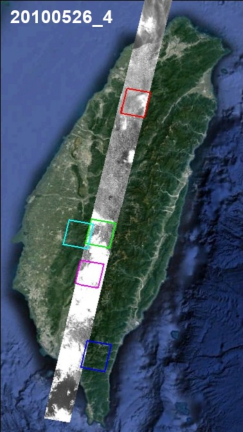
20100526_4

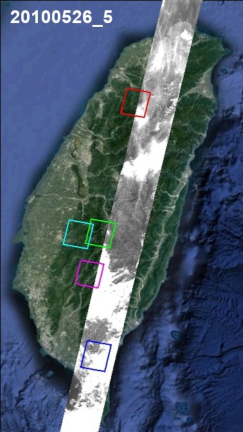
20100526_5

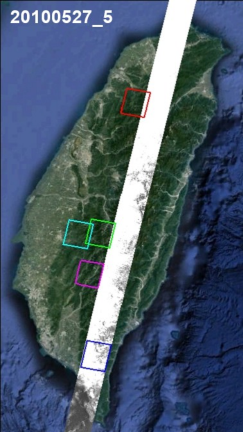
20100527_5

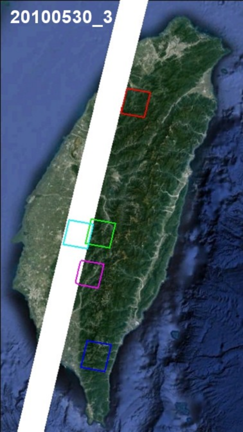
20100530_3

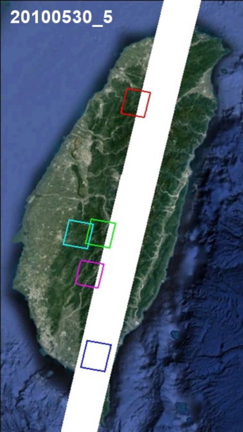
20100530_5

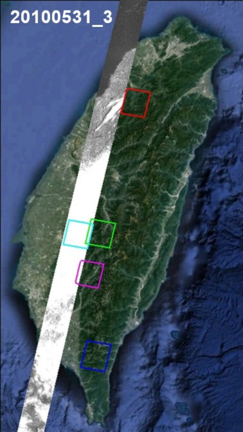
20100531_3

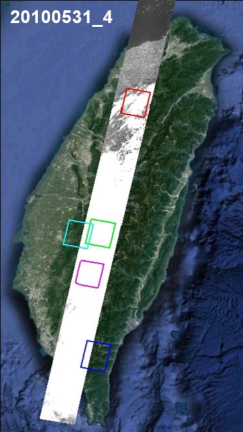
20100531_4

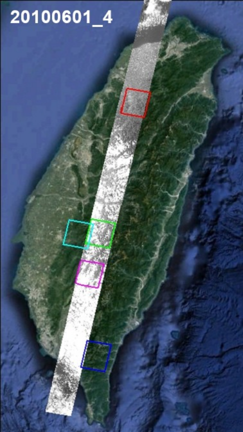
20100601_4

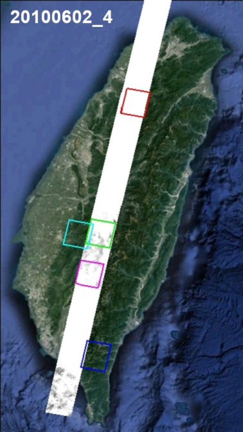
20100602_4

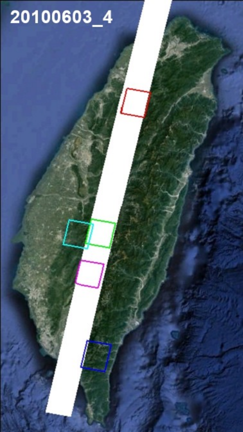
20100603_4

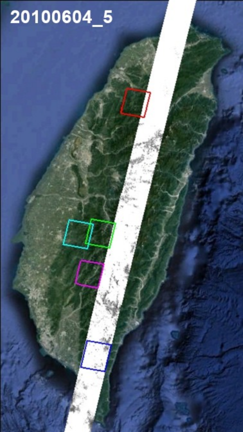
20100604_5

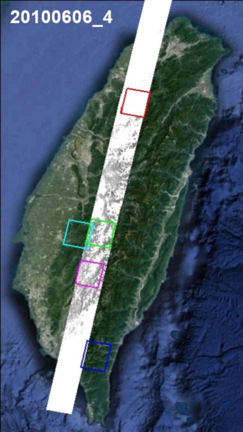
20100606_4

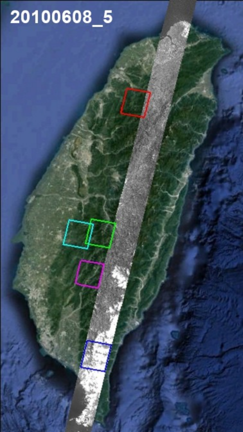
20100608_5

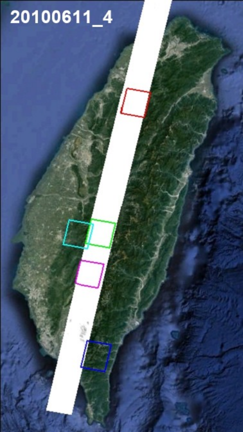
20100611_4

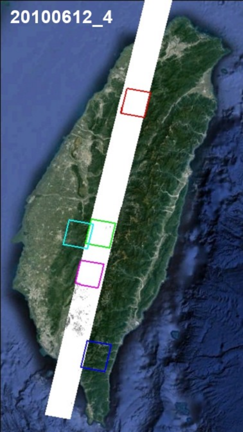
20100612_4

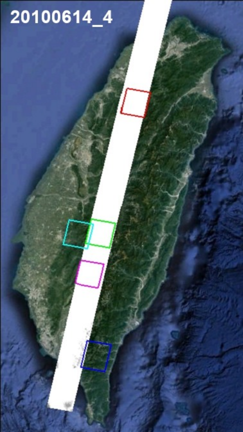
20100614_4

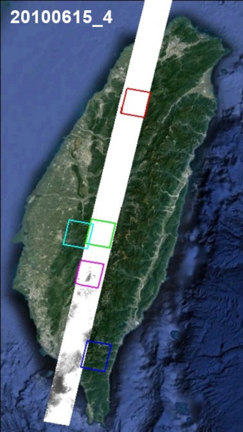
20100615_4

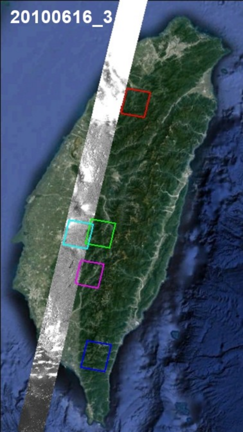
20100616_3

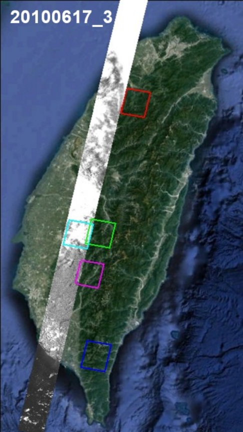
20100617_3

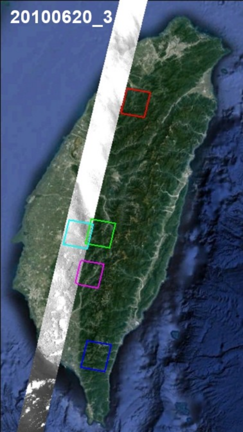
20100620_3

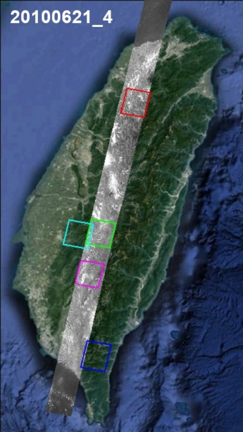
20100621_4

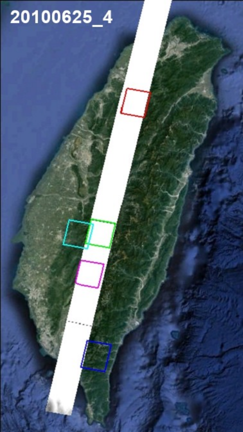
20100625_4

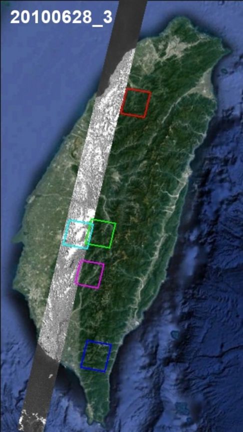
20100628_3

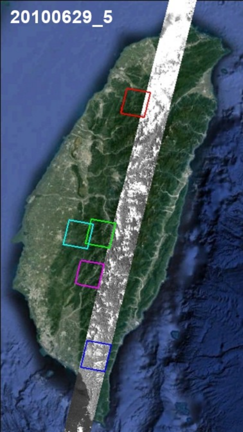
20100629_5

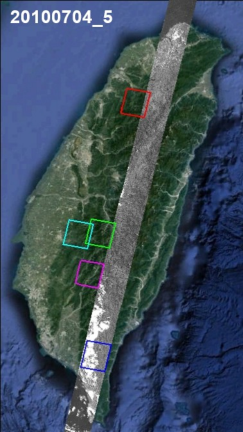
20100704_5

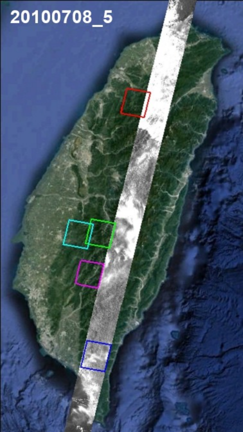
20100708_5

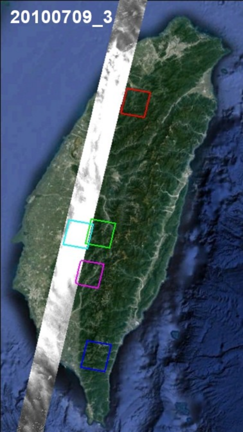
20100709_3

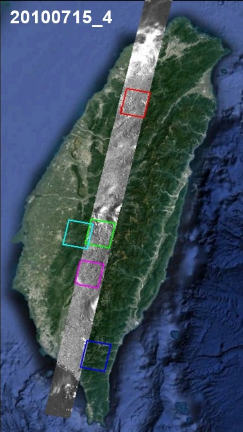
20100715_4

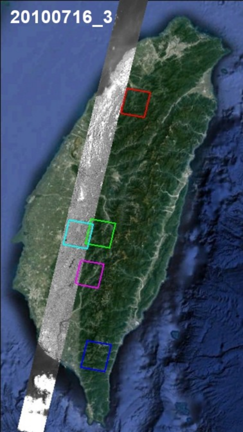
20100716_3

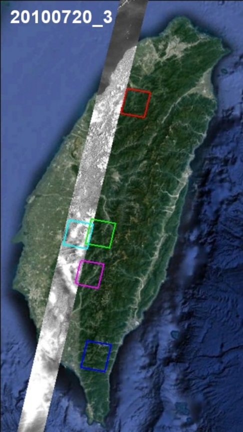
20100720_3

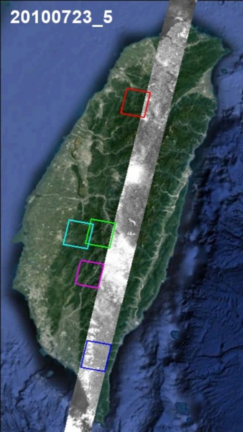
20100723_5

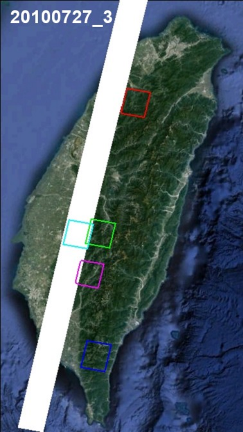
20100727_3

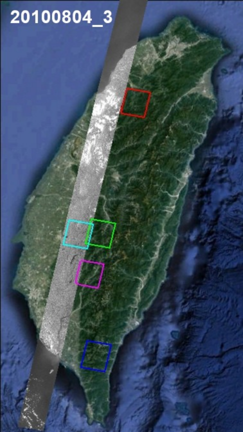
20100804_3

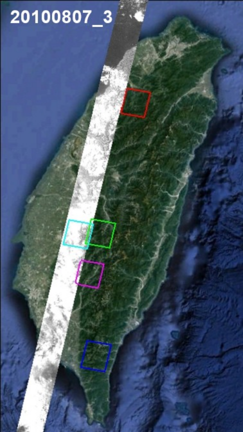
20100807_3

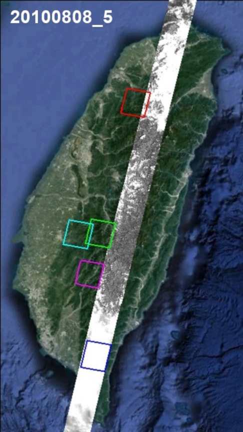
20100808_5

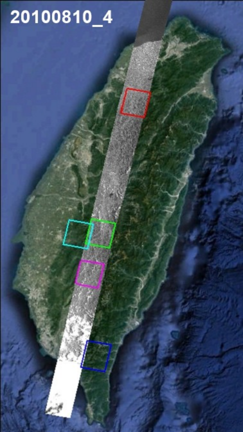
20100810_4

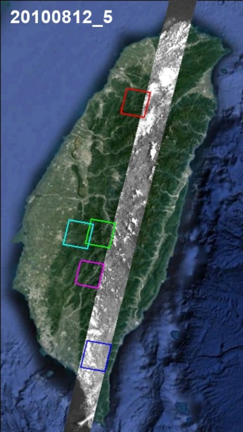
20100812_5

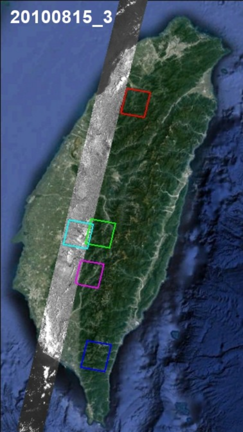
20100815_3

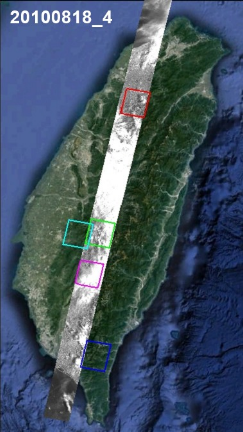
20100818_4

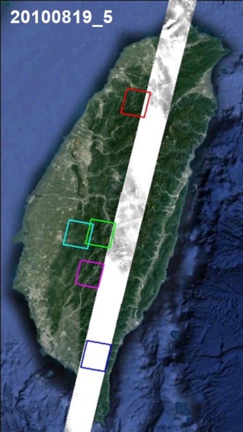
20100819_5

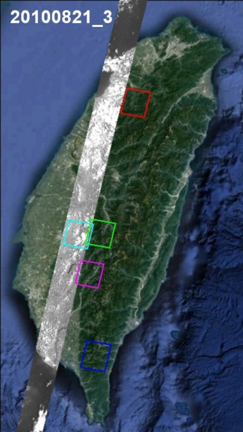
20100821_3

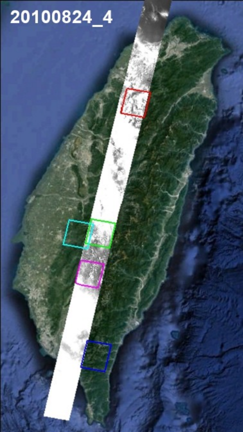
20100824_4

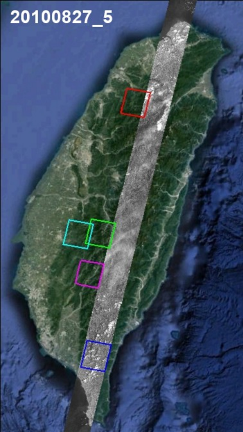
20100827_5

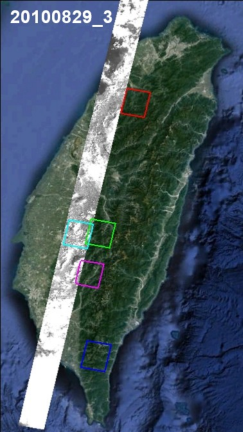
20100829_3

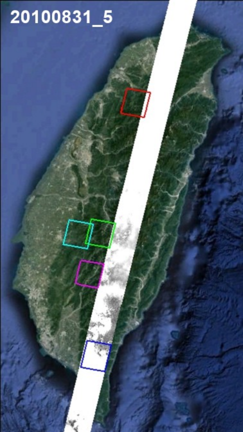
20100831_5

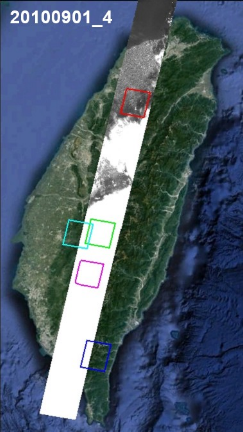
20100901_4

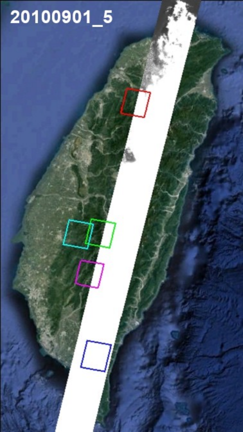
20100901_5

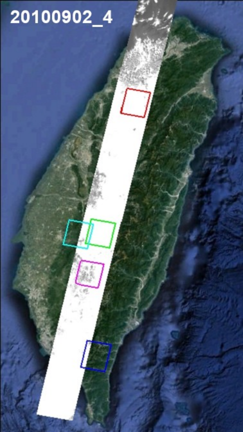
20100902_4

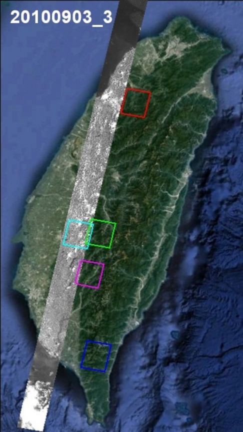
20100903_3

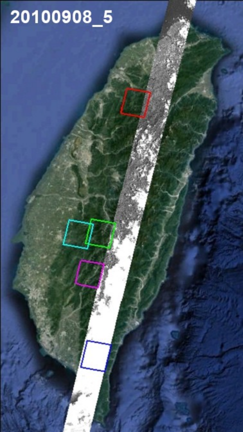
20100908_5

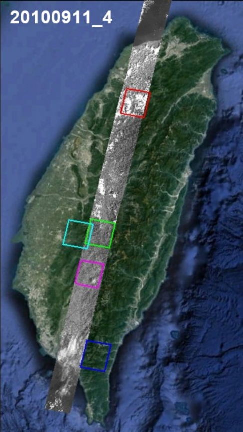
20100911_4

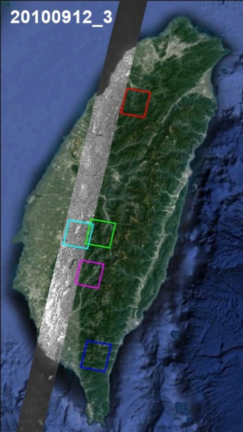
20100912_3

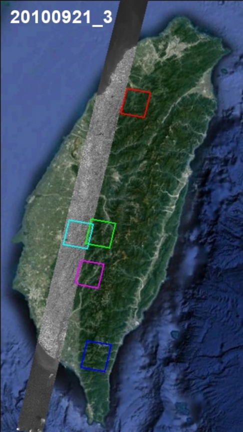
20100921_3

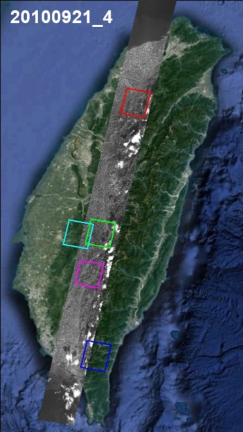
20100921_4

20100922_4

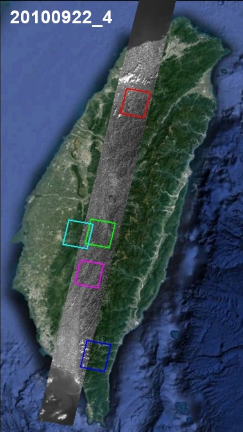

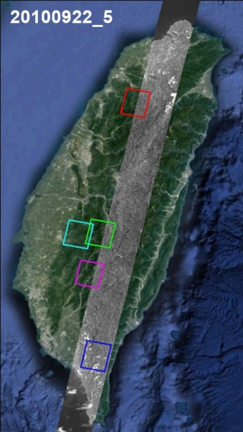
20100922_5

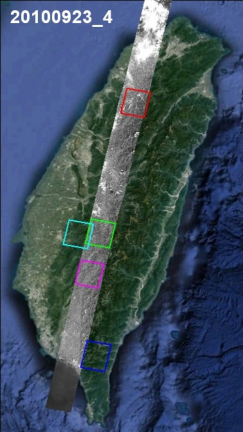
20100923_4

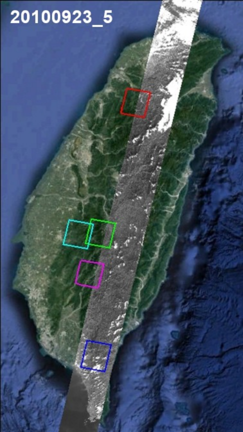
20100923_5

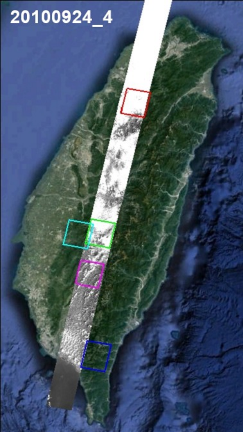
20100924_4

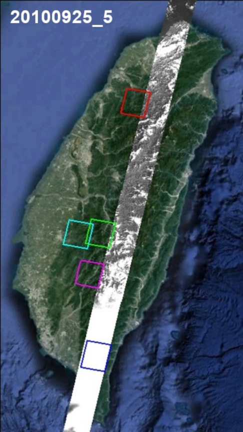
20100925_5

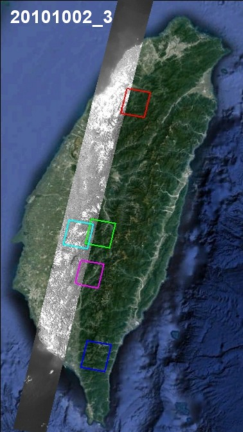
20101002_3

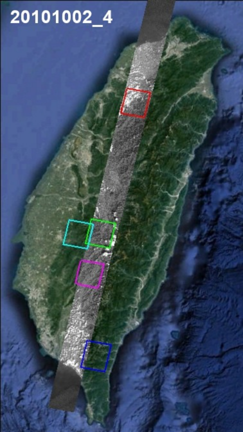
20101002_4

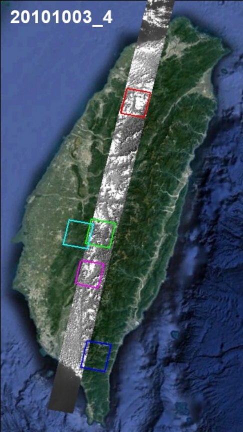
20101003_4

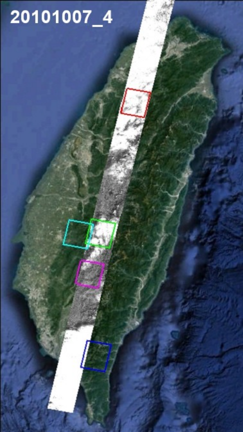
20101007_4

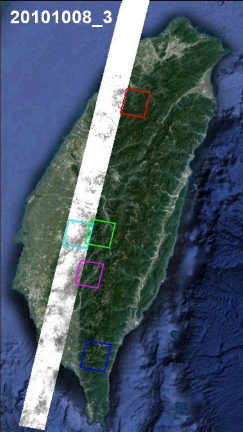
20101008_3

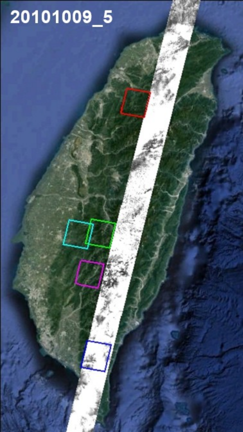
20101009_5

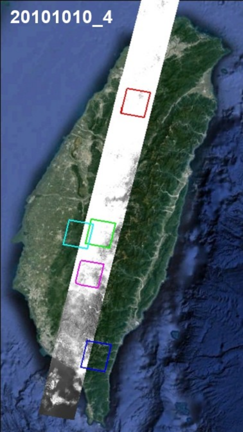
20101010_4

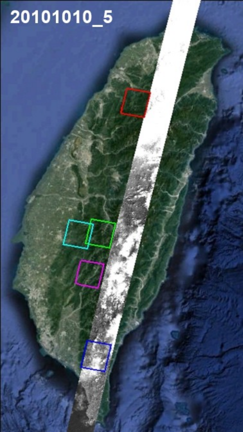
20101010_5

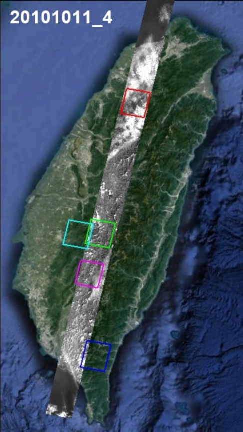
20101011_4

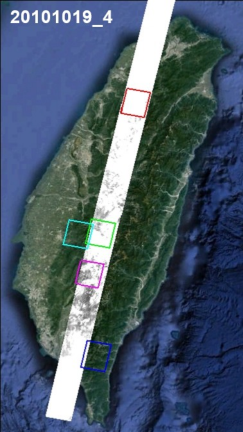
20101019_4

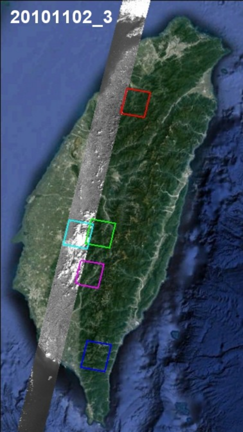
20101102_3

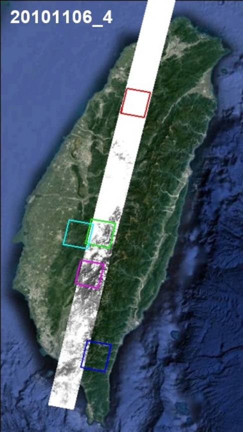
20101106_4

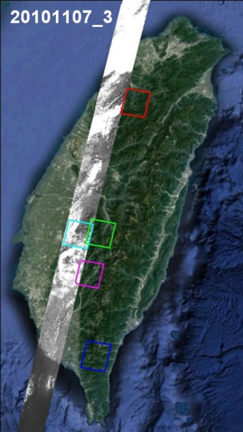
20101107_3

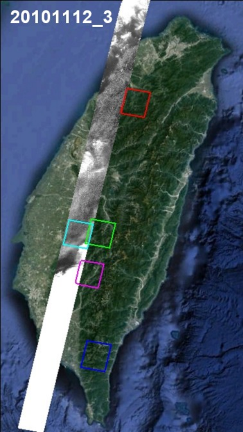
20101112_3

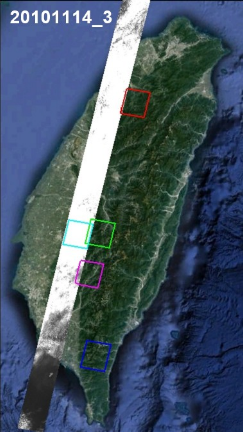
20101114_3

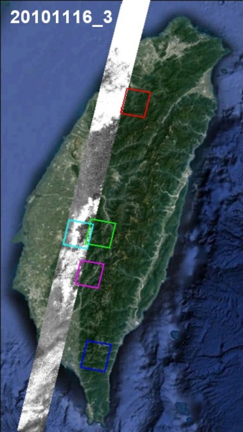
20101116_3

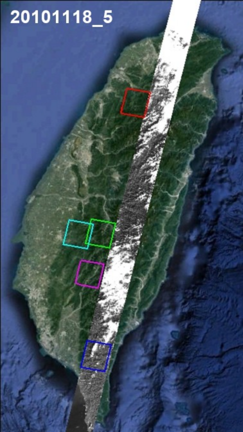
20101118_5

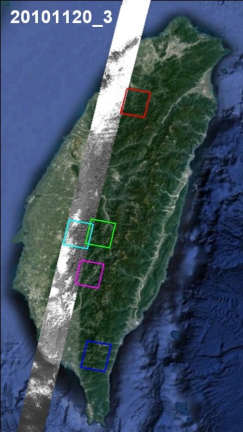
20101120_3

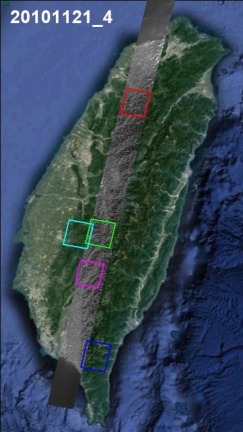
20101121_4

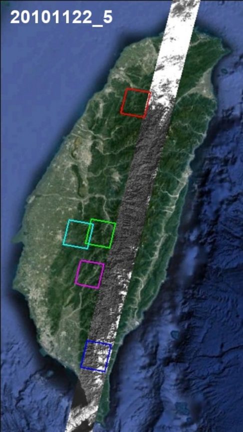
20101122_5

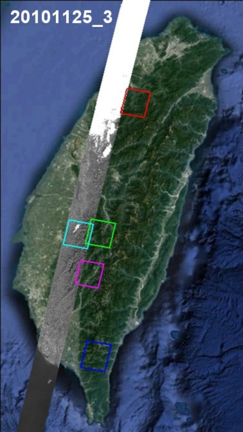
20101125_3

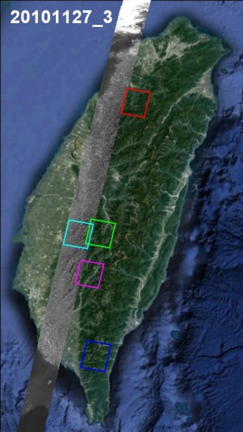
20101127_3

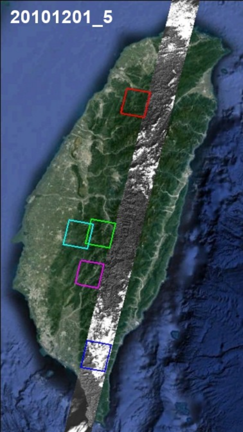
20101201_5

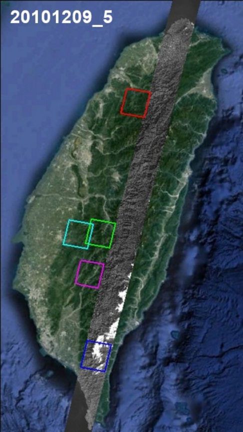
20101209_5

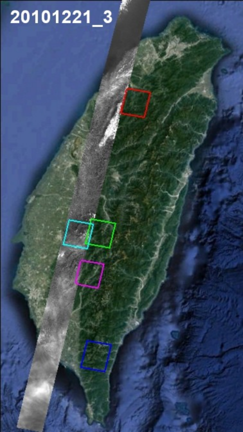
20101221_3

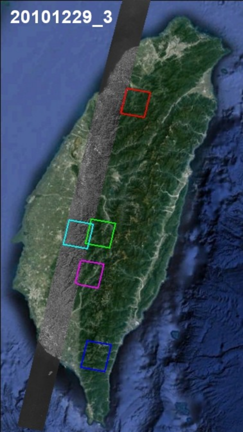
20101229_3

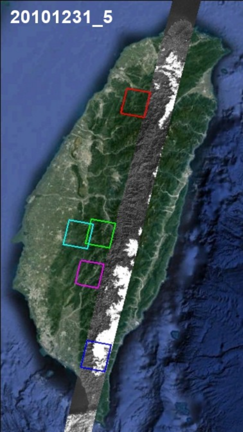
20101231_5

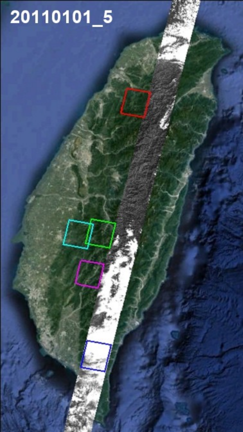
20110101_5

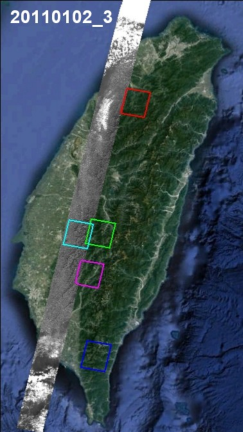
20110102_3

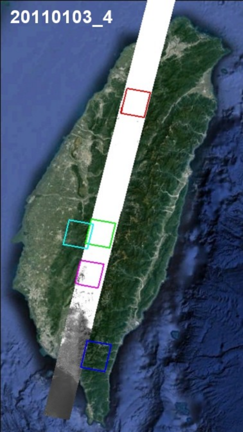
20110103_4

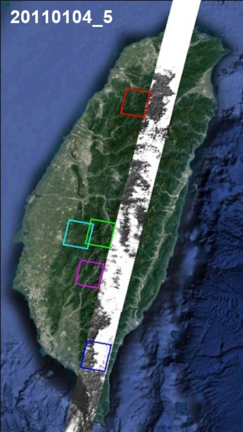
20110104_5

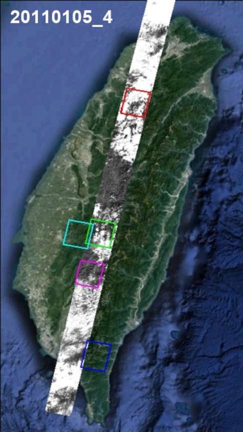
20110105_4

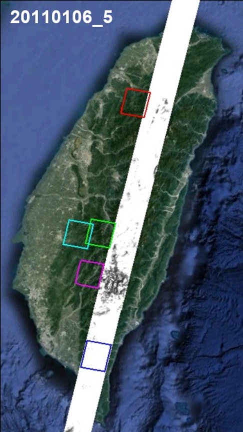
20110106_5

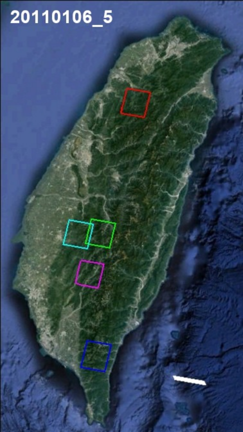
20110106_5

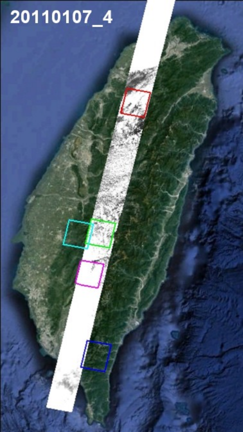
20110107_4

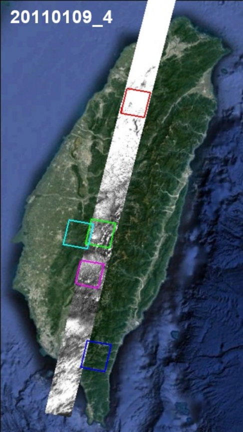
20110109_4

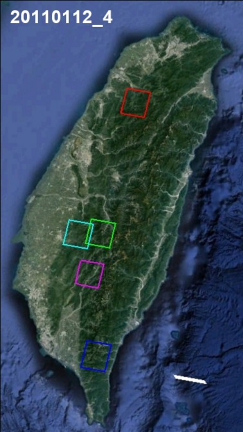
20110112_4

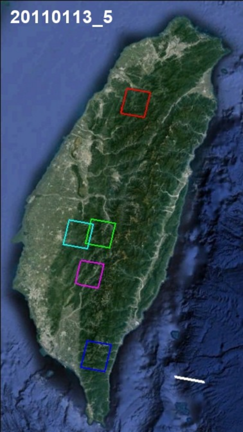
20110113_5

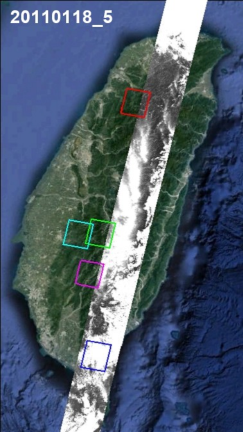
20110118_5

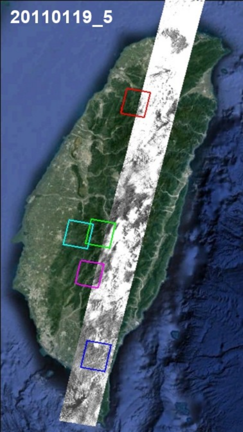
20110119_5

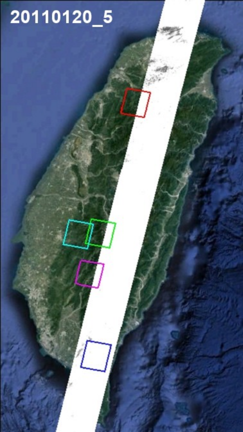
20110120_5

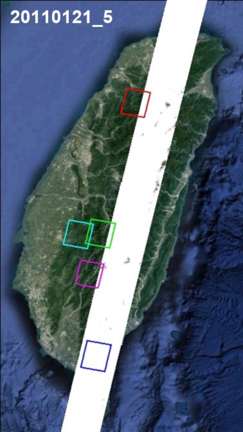
20110121_5

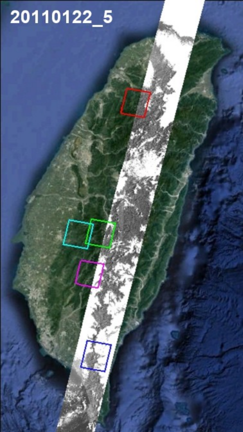
20110122_5

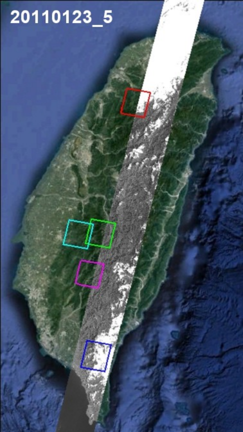
20110123_5

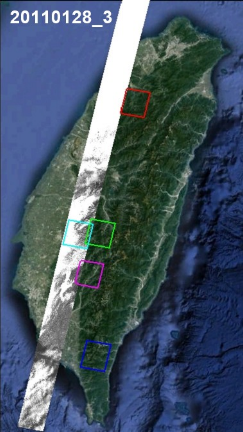
20110128_3

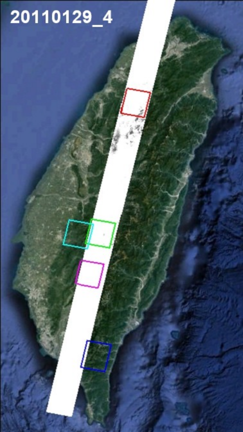
20110129_4

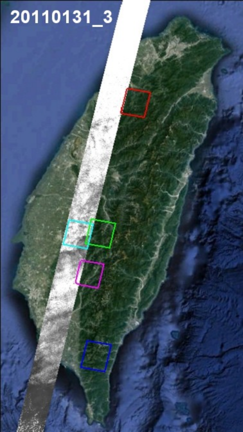
20110131_3

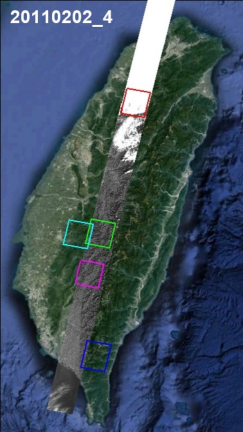
20110202_4

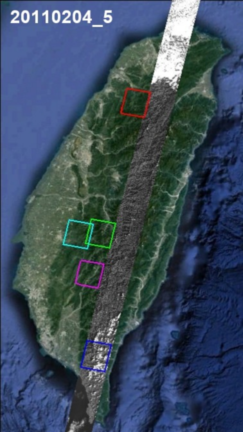
20110204_5

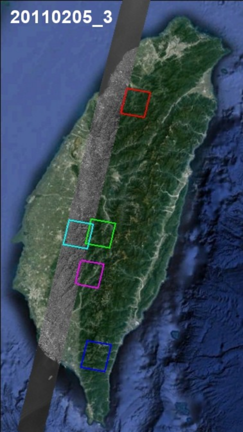
20110205_3

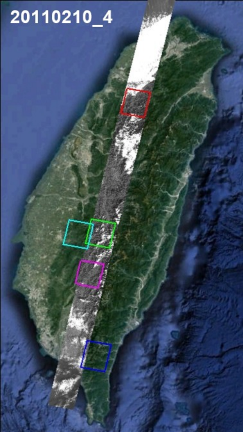
20110210_4

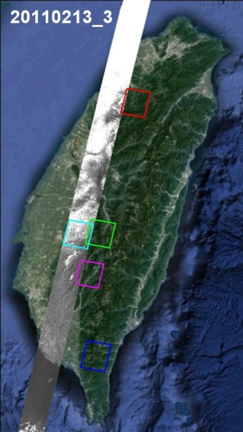
20110213_3

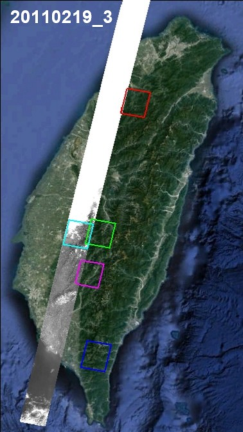
20110219_3

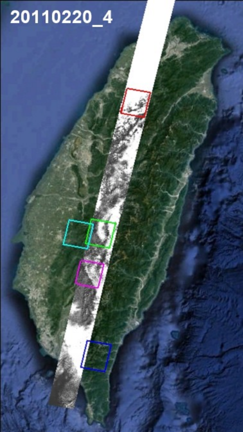
20110220_4

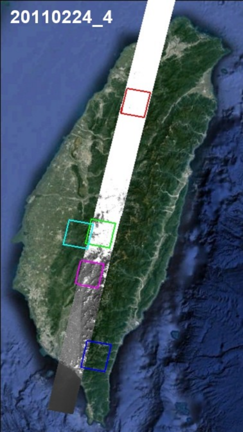
20110224_4

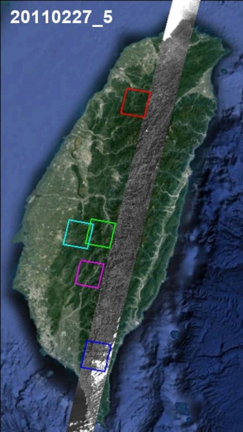
20110227_5

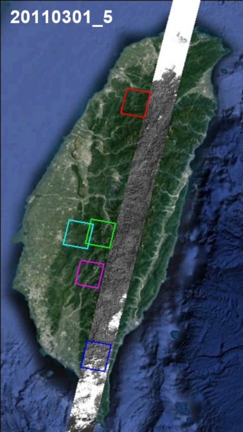
20110301_5

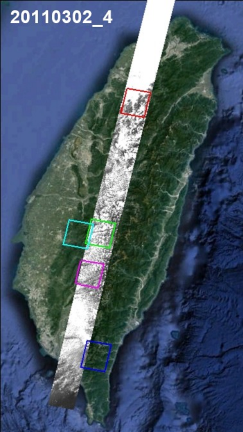
20110302_4

20110310_4

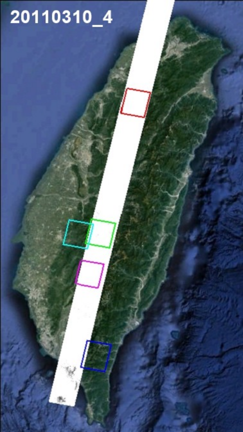

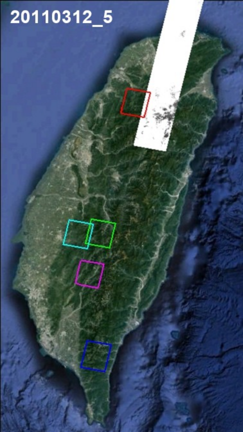
20110312_5

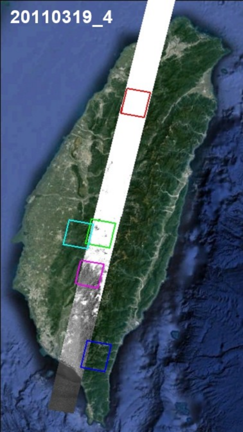
20110319_4

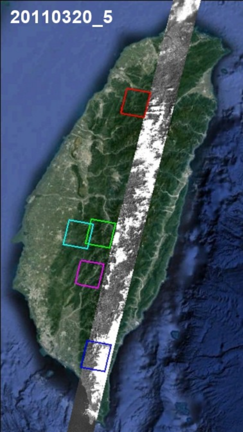
20110320_5

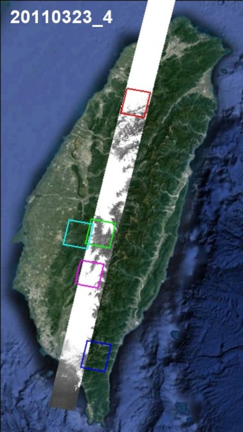
20110323_4

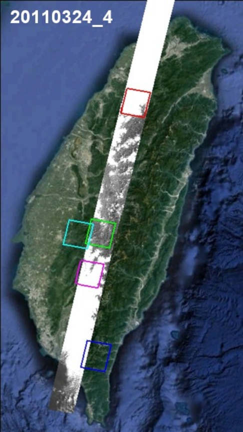
20110324_4

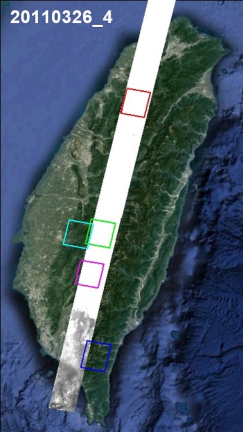
20110326_4

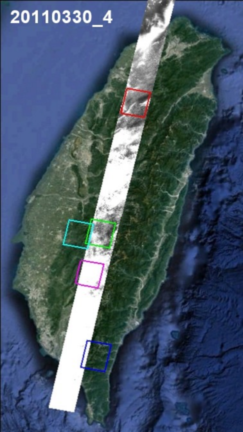
20110330_4

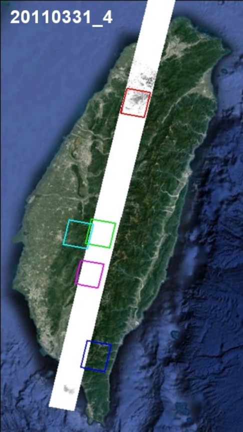
20110331_4

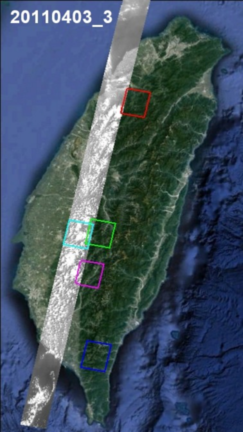
20110403_3

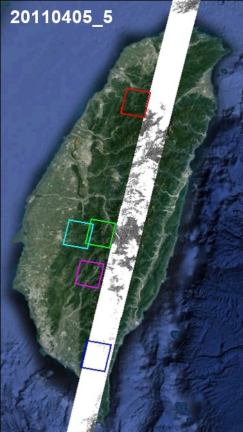
20110405_5

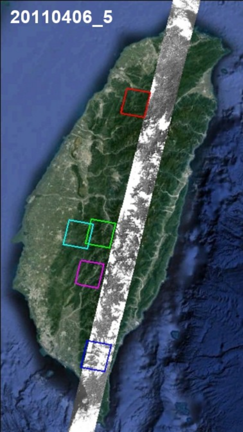
20110406_5

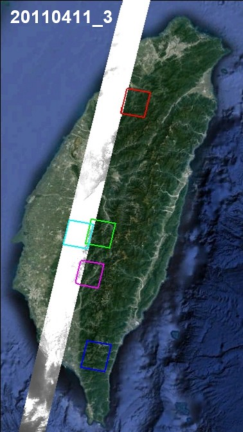
20110411_3

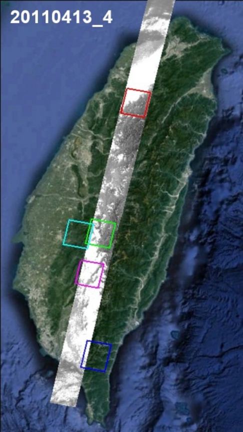
20110413_4

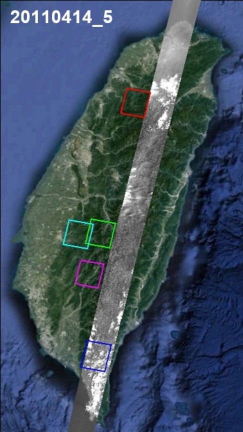
20110414_5

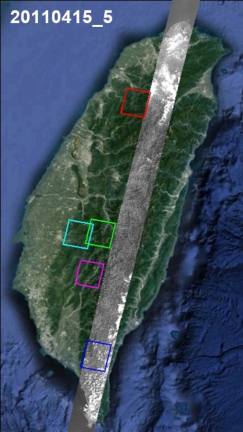
20110415_5

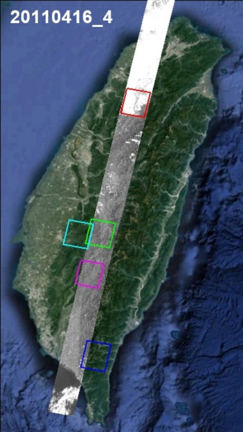
20110416_4

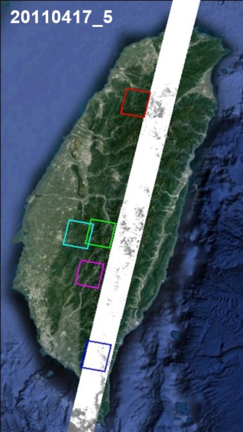
20110417_5

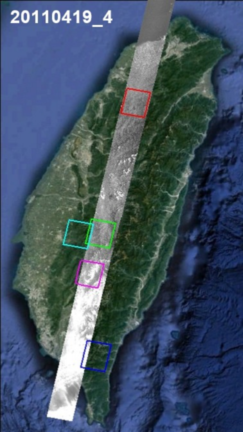
20110419_4

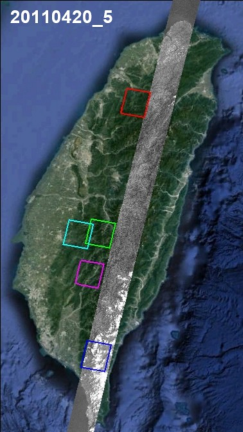
20110420_5

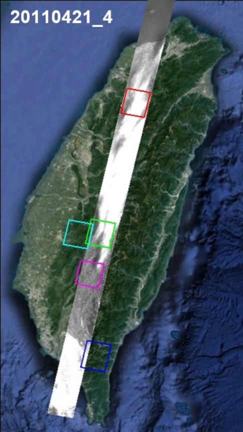
20110421_4

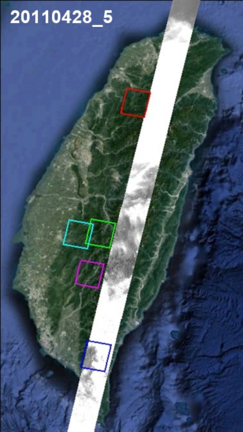
20110428_5

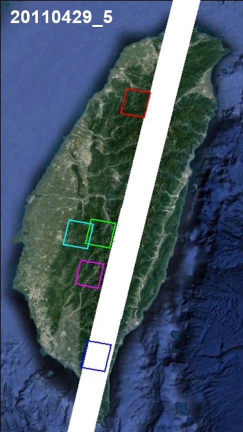
20110429_5

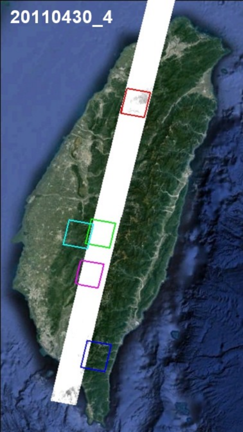
20110430_4

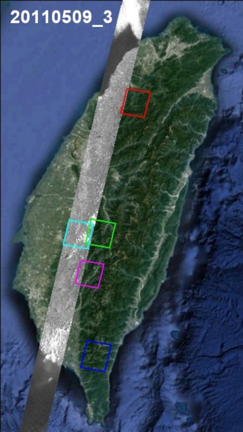
20110509_3

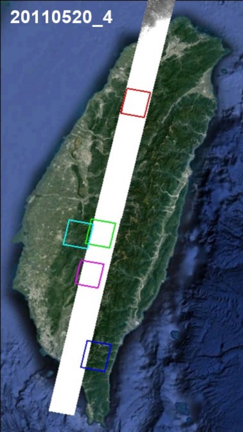
20110520_4

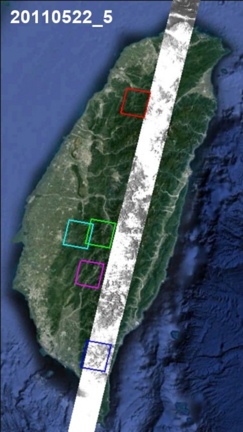
20110522_5

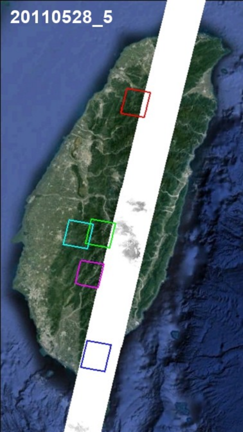
20110528_5

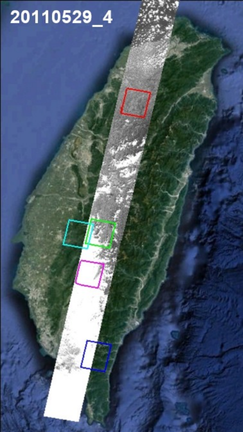
20110529_4

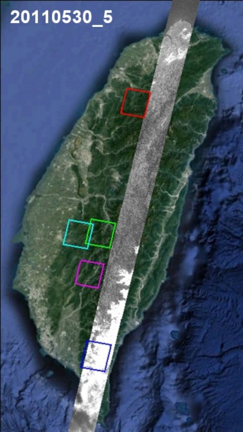
20110530_5

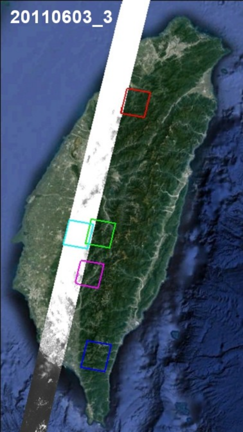
20110603_3

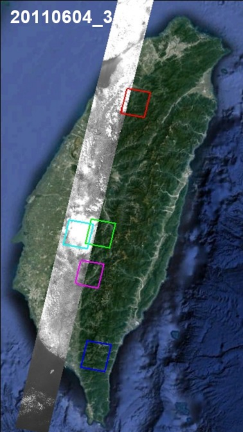
20110604_3

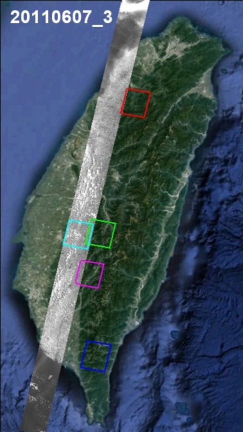
20110607_3

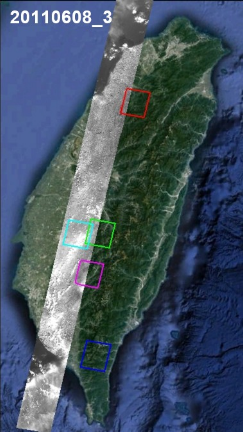
20110608_3

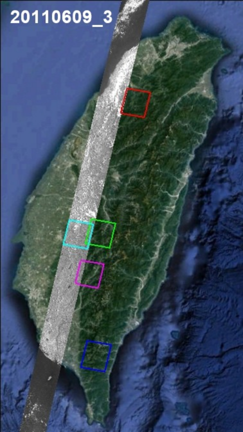
20110609_3

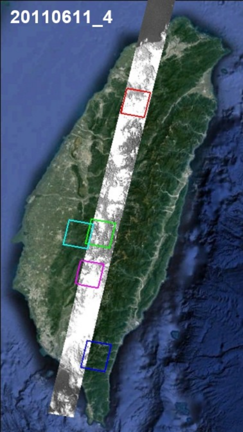
20110611_4

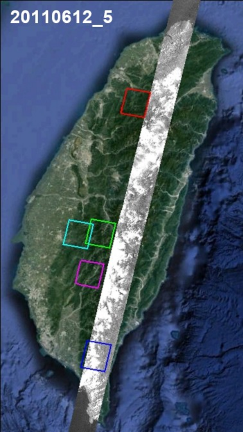
20110612_5

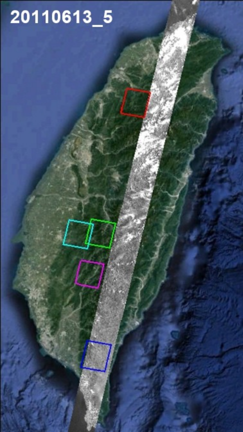
20110613_5

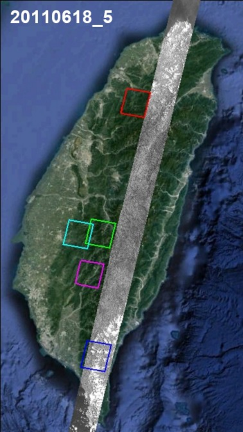
20110618_5

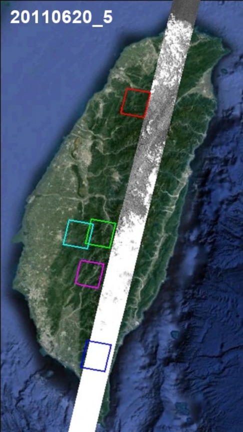
20110620_5

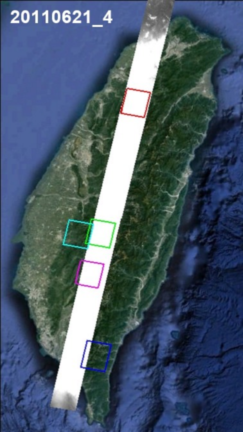
20110621_4

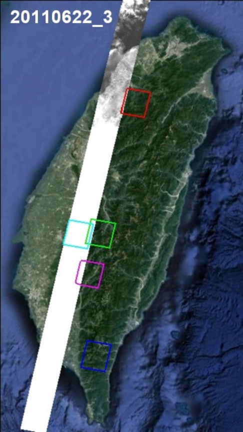
20110622_3

20110623_4

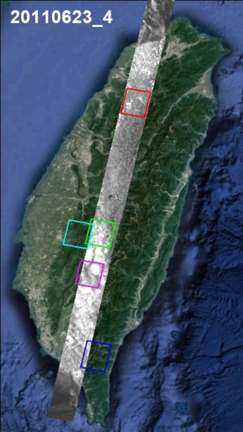

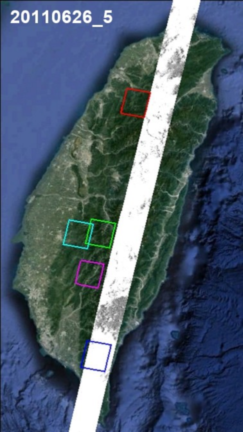
20110626_5

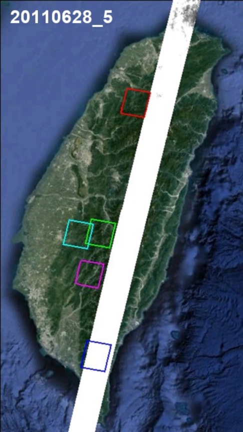
20110628_5

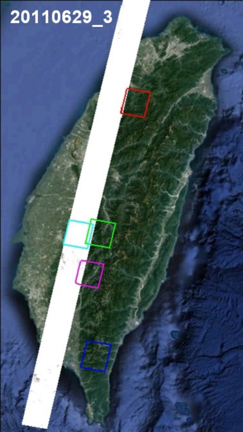
20110629_3

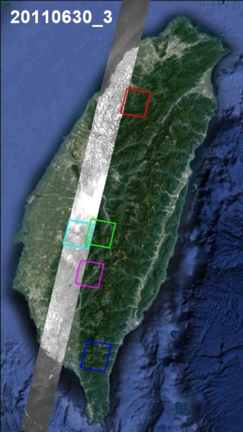
20110630_3

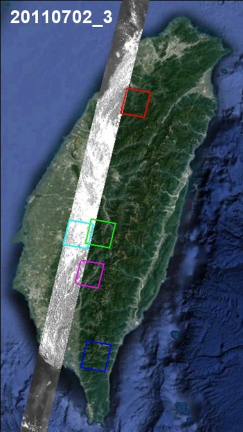
20110702_3

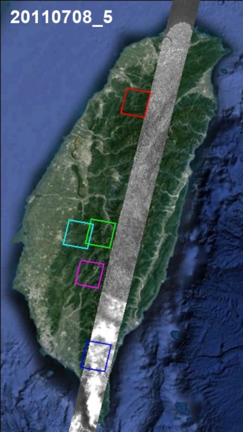
20110708_5

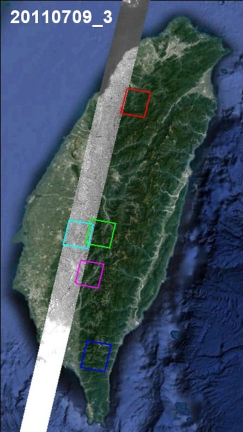
20110709_3

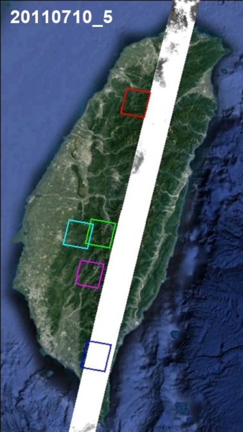
20110710_5

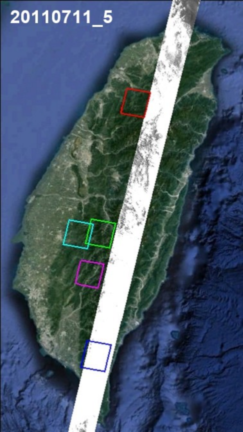
20110711_5

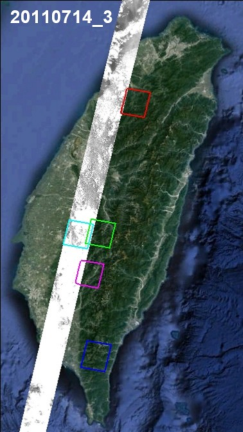
20110714_3

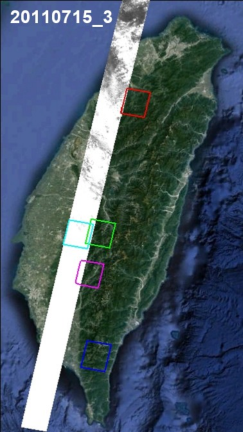
20110715_3

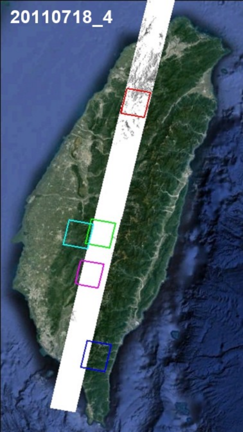
20110718_4

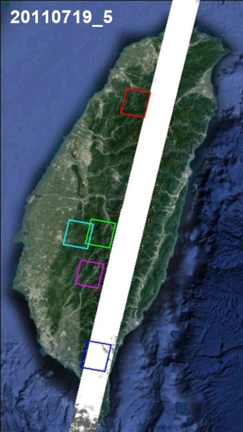
20110719_5

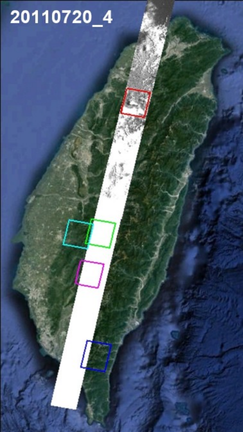
20110720_4

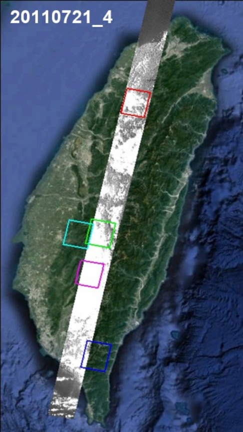
20110721_4

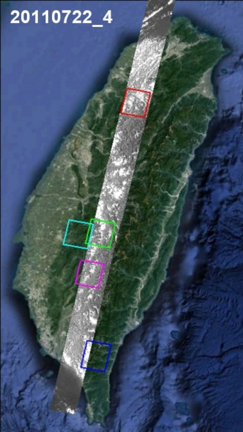
20110722_4

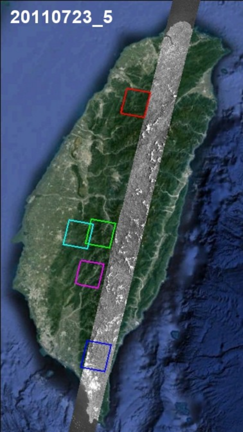
20110723_5

20110726_3

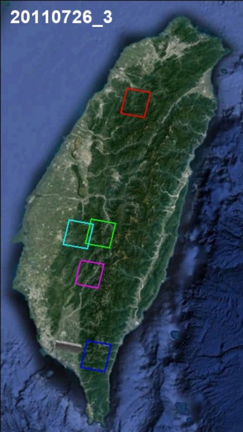

20110728_4

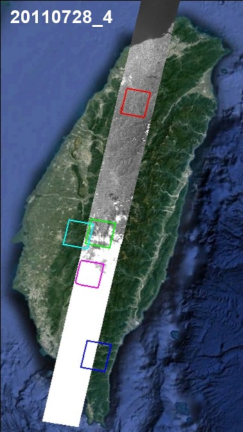

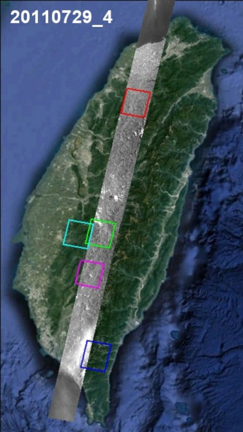
20110729_4

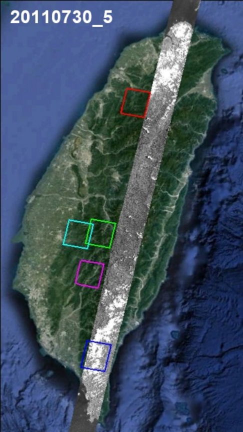
20110730_5

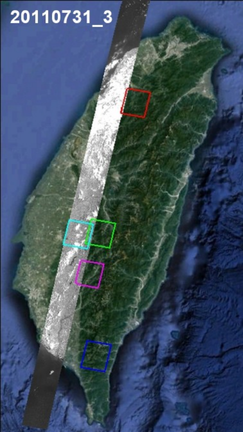
20110731_3

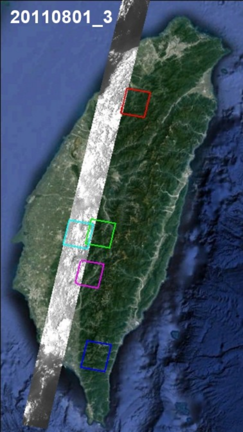
20110801_3

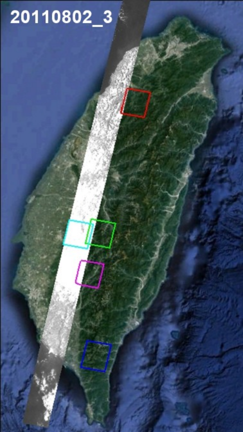
20110802_3

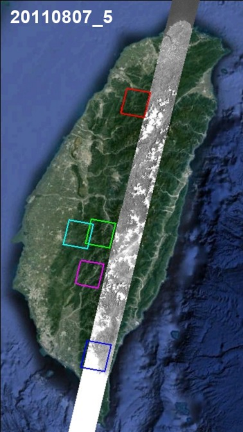
20110807_5

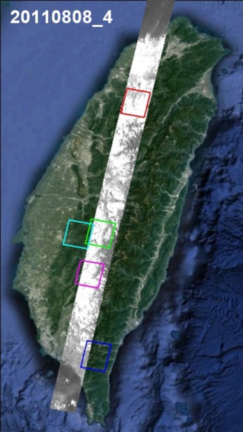
20110808_4

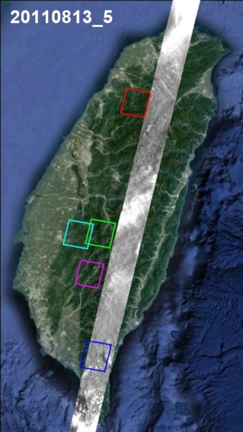
20110813_5

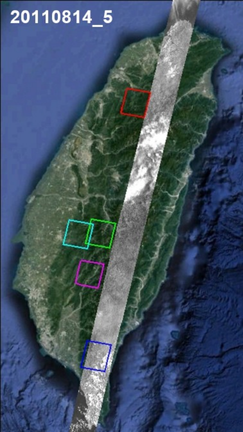
20110814_5

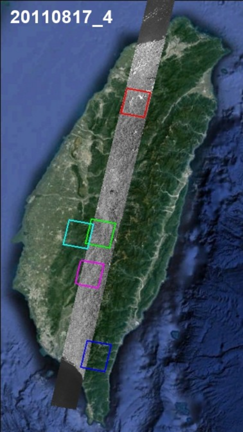
20110817_4

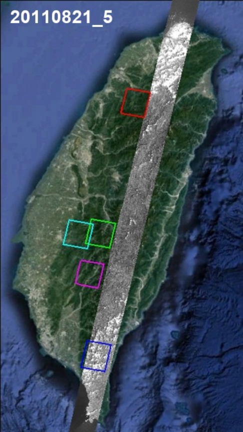
20110821_5

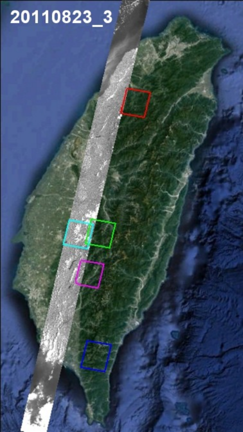
20110823_3

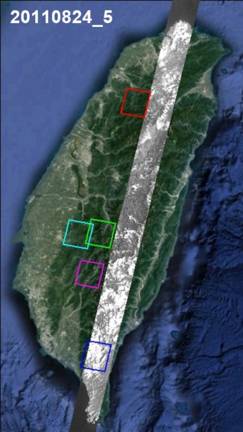
20110824_5

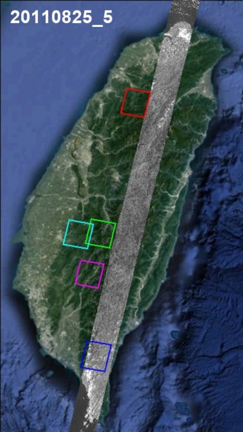
20110825_5

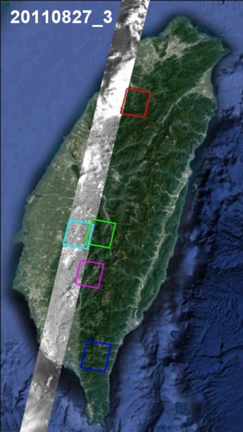
20110827_3

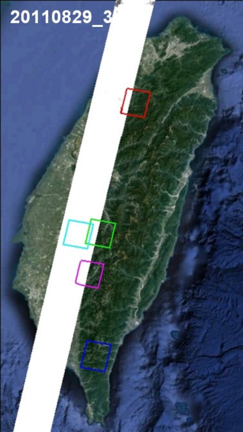
20110829_3

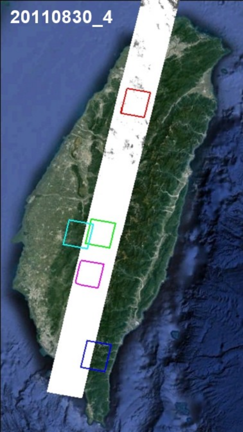
20110830_4

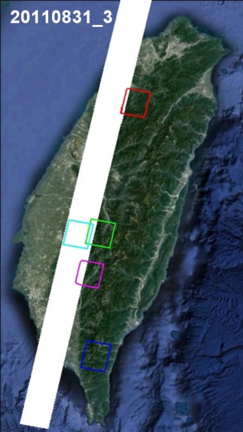
20110831_3

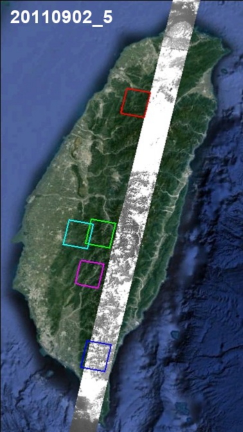
20110902_5

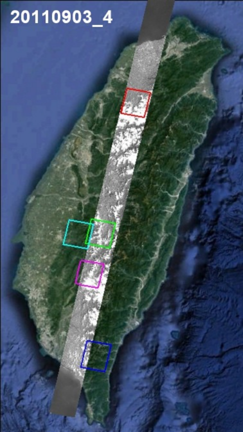
20110903_4

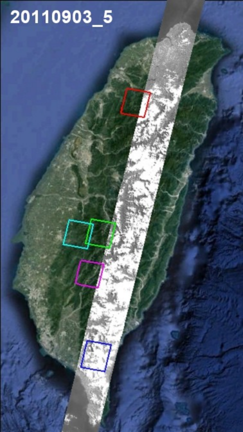
20110903_5

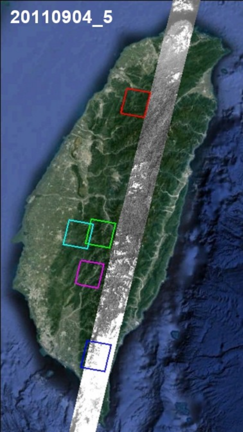
20110904_5

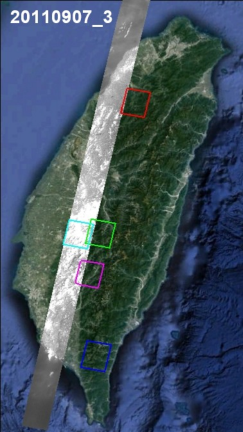
20110907_3

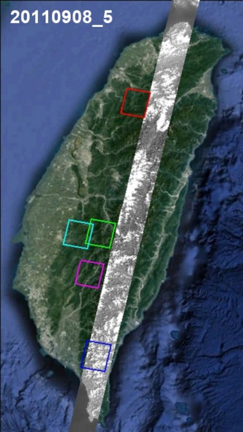
20110908_5

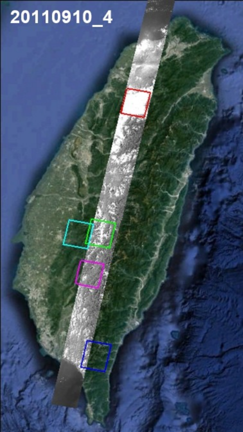
20110910_4

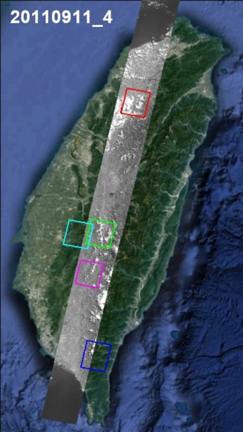
20110911_4

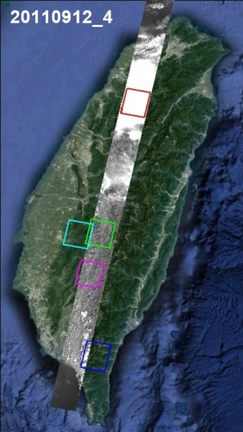
20110912_4

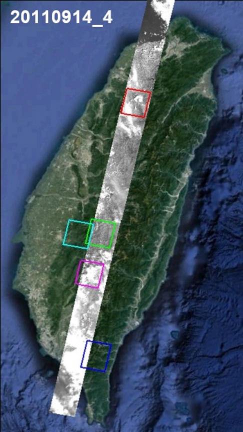
20110914_4

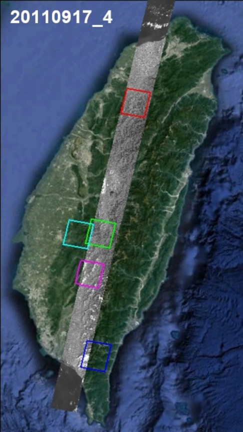
20110917_4

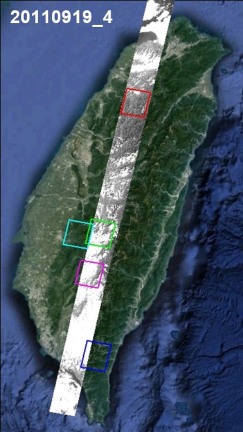
20110919_4

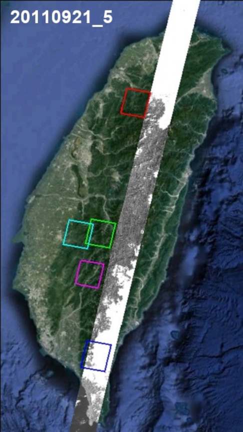
20110921_5

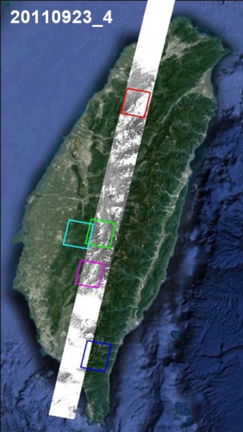
20110923_4

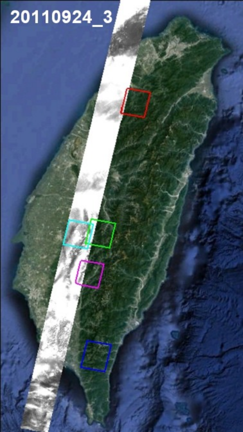
20110924_3

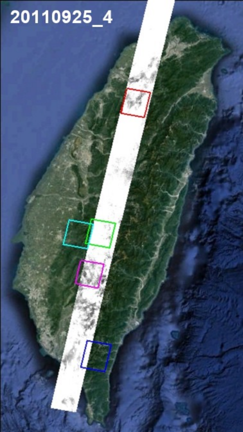
20110925_4

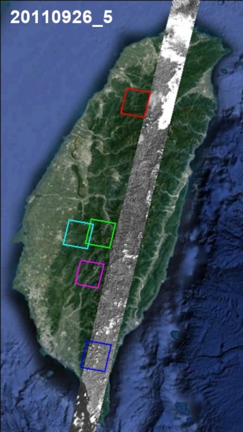
20110926_5

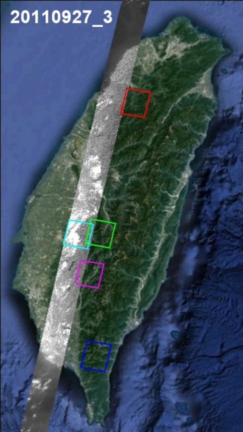
20110927_3

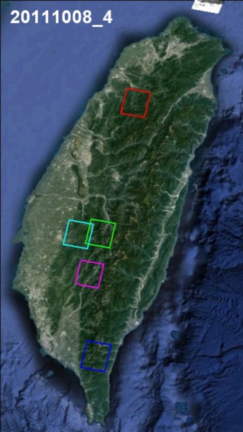
20111008_4

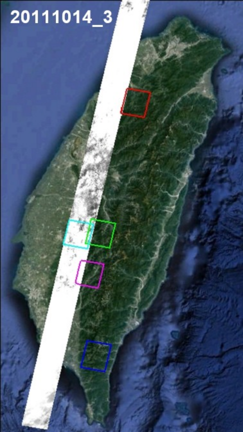
20111014_3

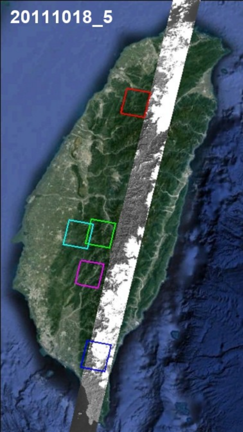
20111018_5

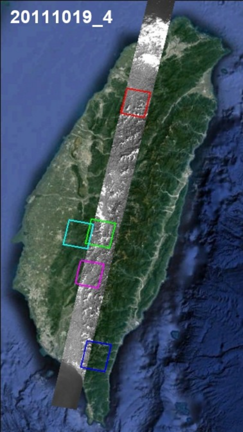
20111019_4

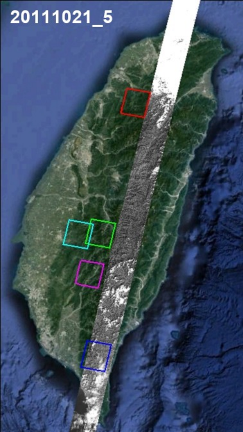
20111021_5

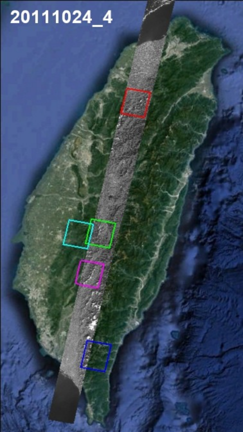
20111024_4

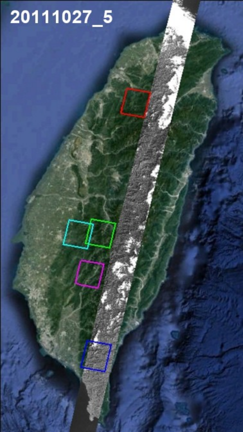
20111027_5

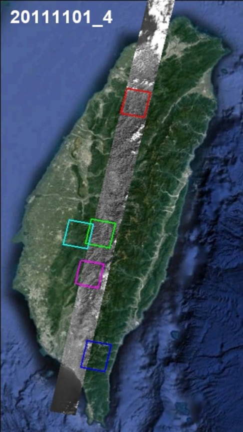
20111101_4

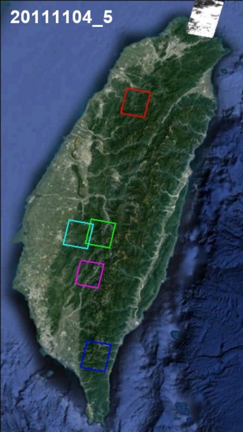
20111104_5

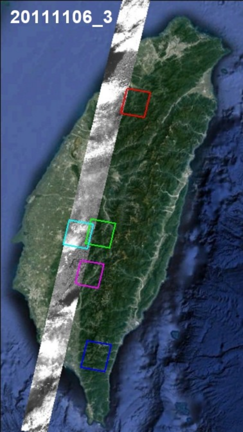
20111106_3

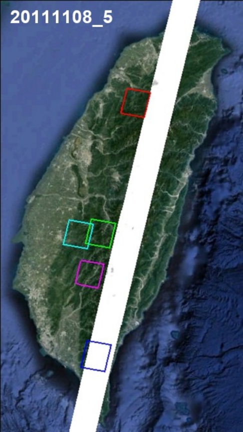
20111108_5

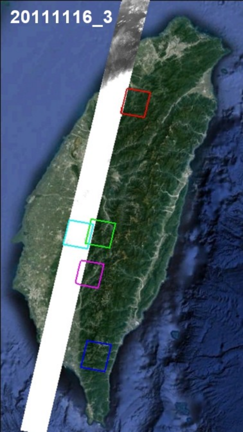
20111116_3

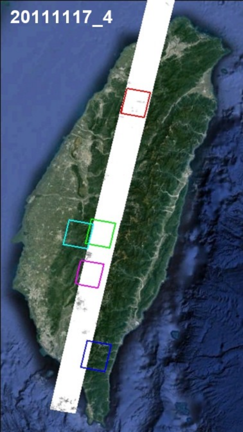
20111117_4

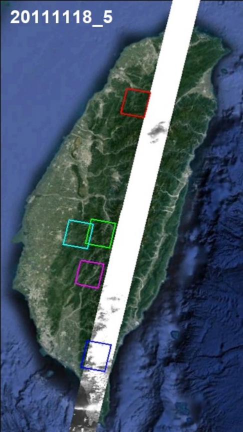
20111118_5

20111121_5

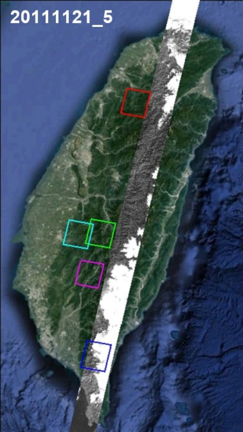

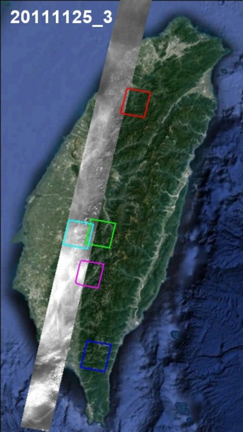
20111125_3

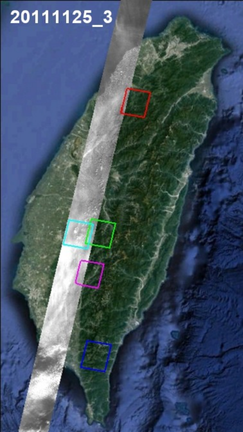
20111125_3

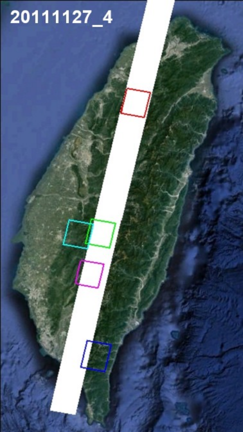
20111127_4

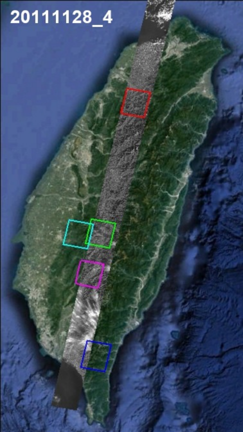
20111128_4

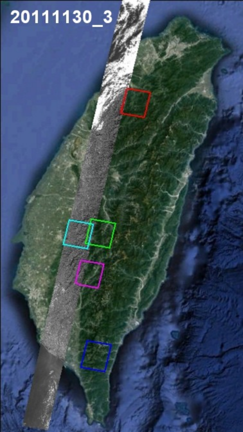
20111130_3

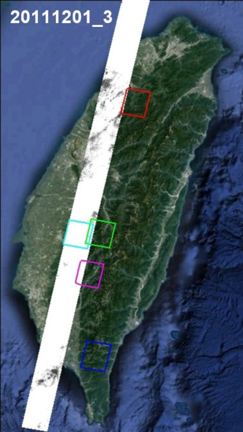
20111201_3

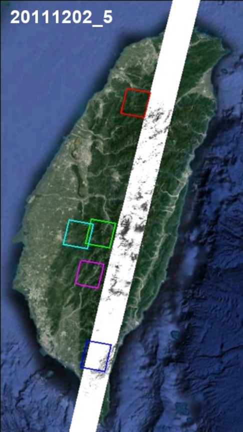
20111202_5

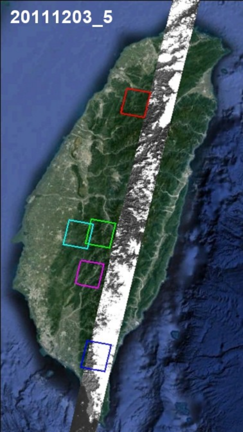
20111203_5

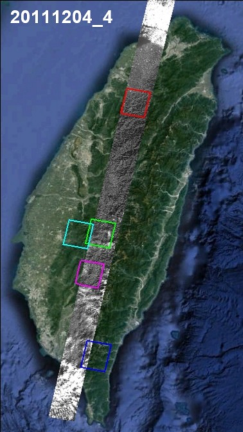
20111204_4

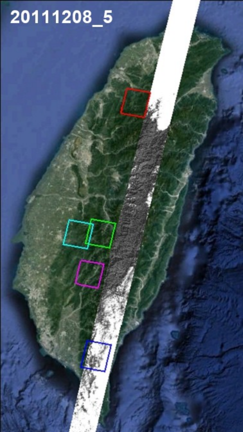
20111208_5

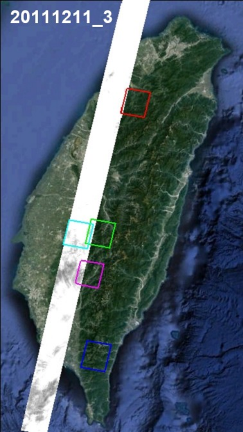
20111211_3

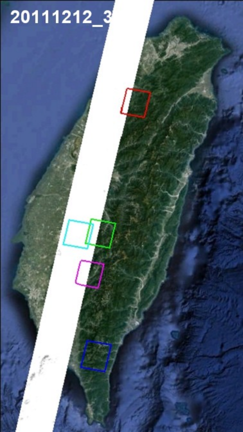
20111212_3

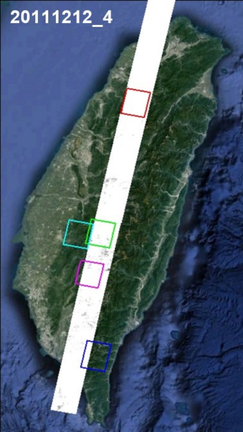
20111212_4

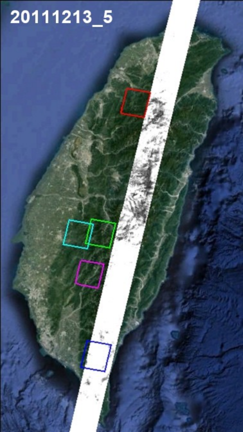
20111213_5

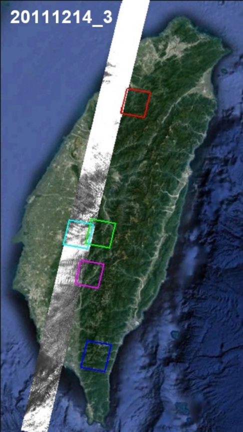
20111214_3

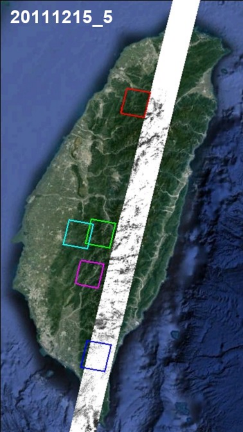
20111215_5

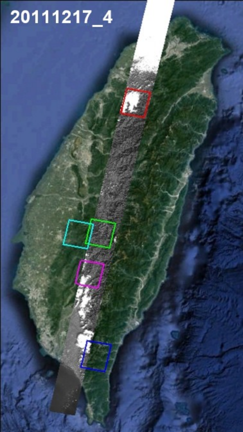
20111217_4

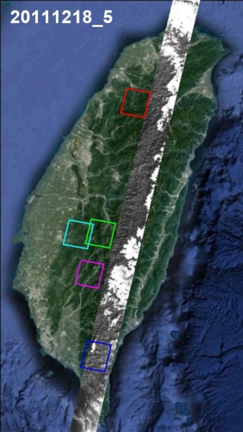
20111218_5

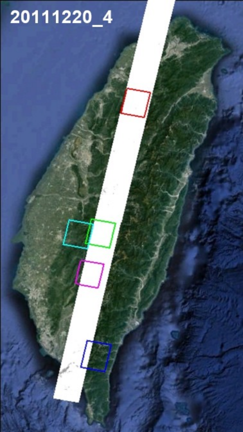
20111220_4

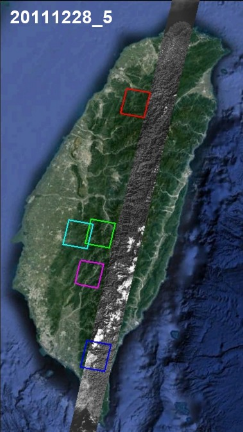
20111228_5

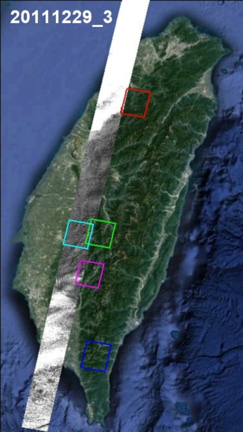
20111229_3

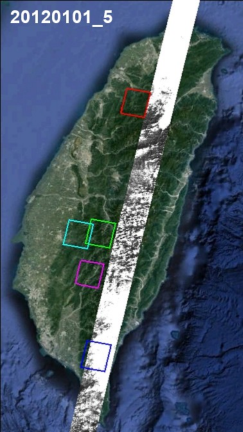
20120101_5

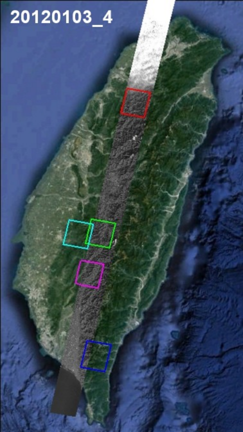
20120103_4

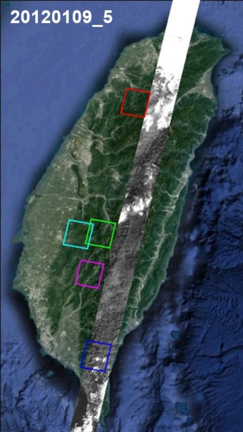
20120109_5

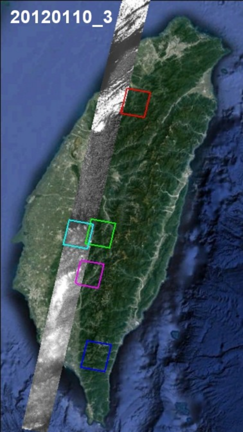
20120110_3

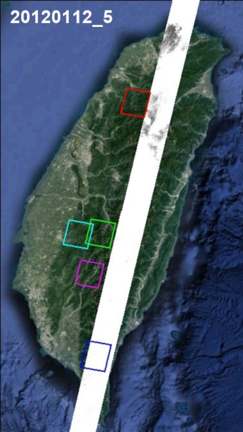
20120112_5

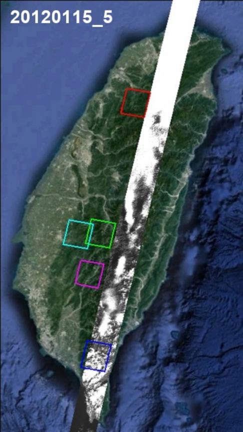
20120115_5

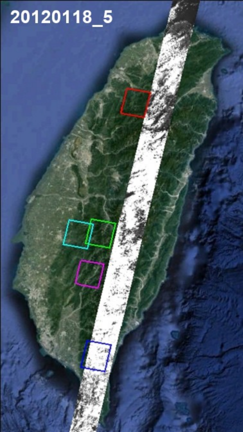
20120118_5

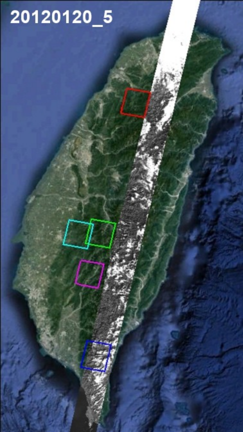
20120120_5

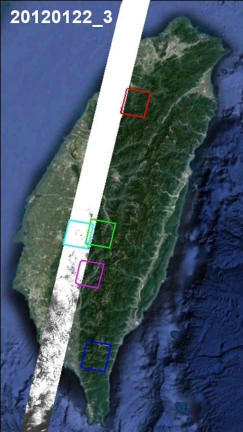
20120122_3

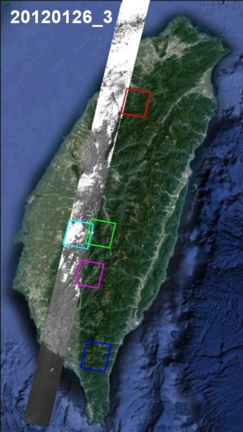
20120126_3

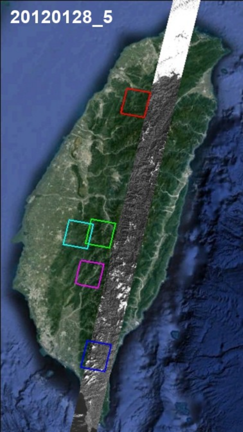
20120128_5

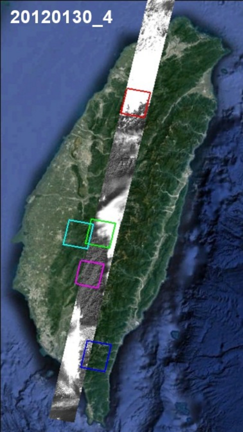
20120130_4

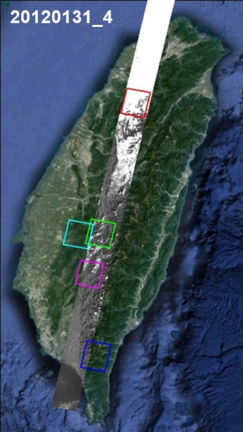
20120131_4

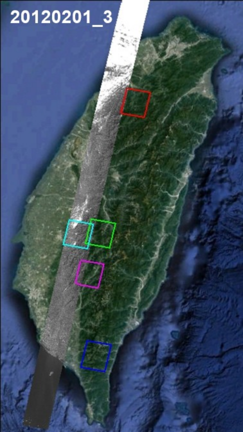
20120201_3

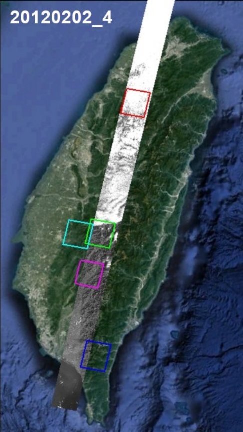
20120202_4

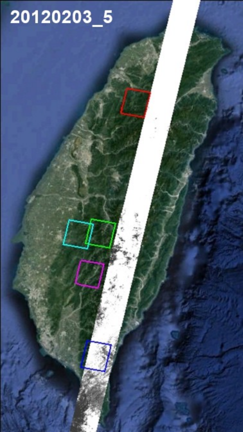
20120203_5

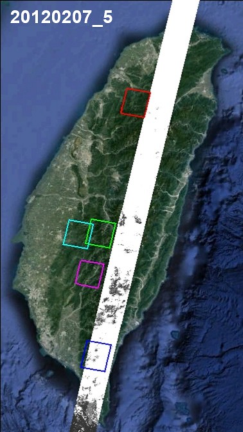
20120207_5

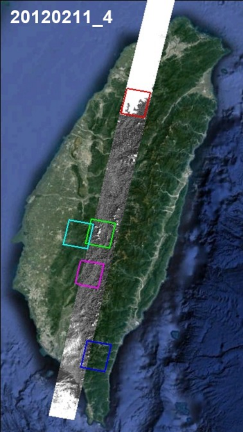
20120211_4

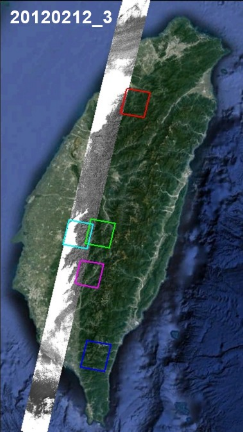
20120212_3

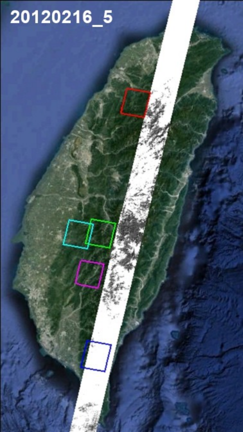
20120216_5

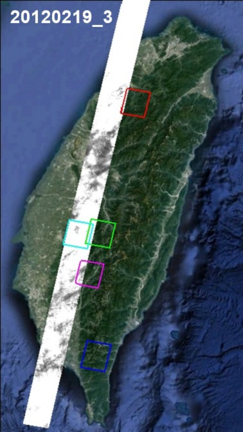
20120219_3

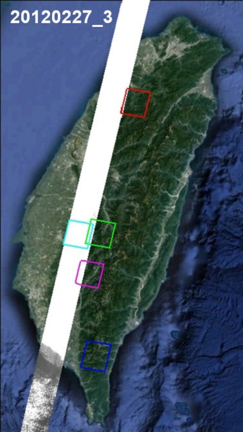
20120227_3

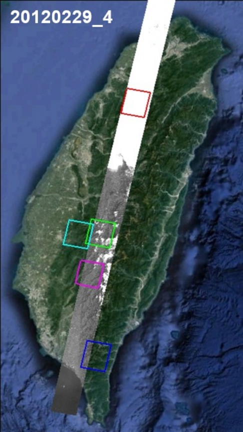
20120229_4

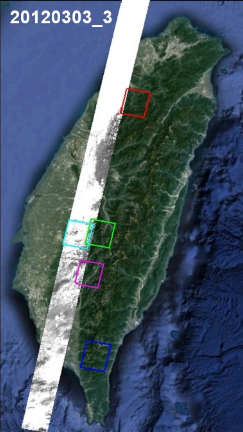
20120303_3

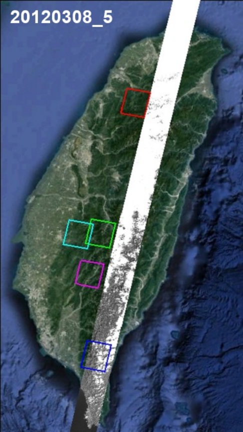
20120308_5

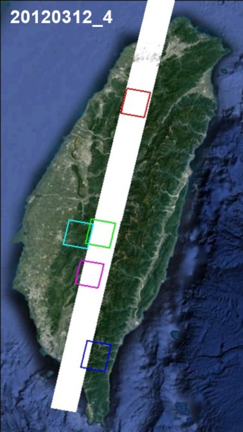
20120312_4

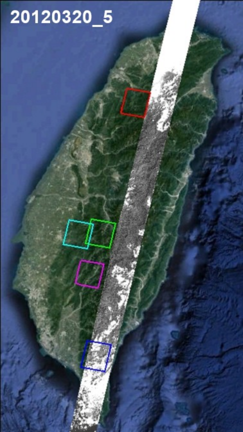
20120320_5

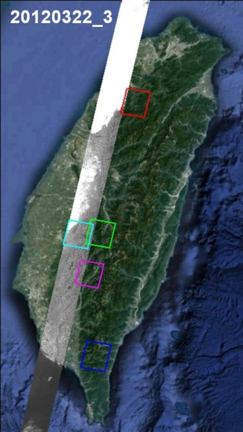
20120322_3

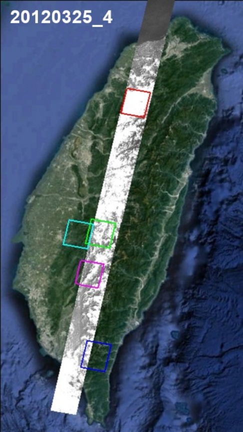

20120325_4

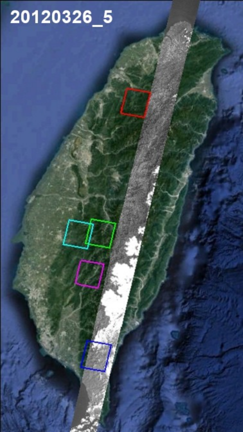
20120326_5

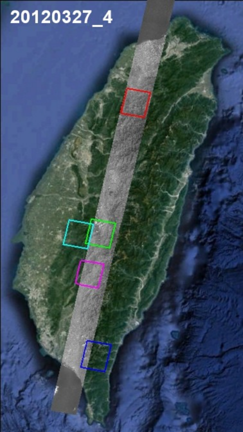

20120327_4

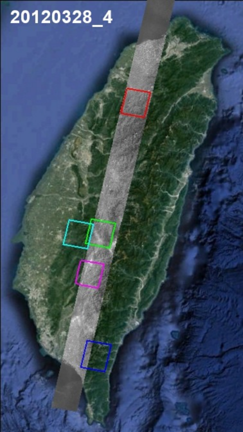
20120328_4

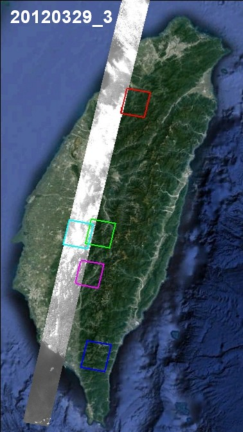
20120329_3

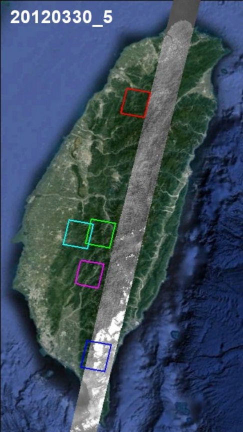
20120330_5

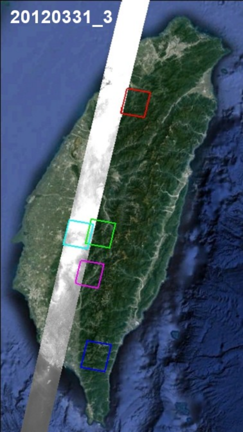
20120331_3

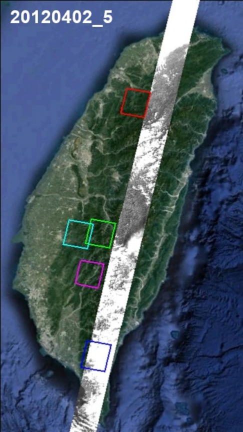
20120402_5

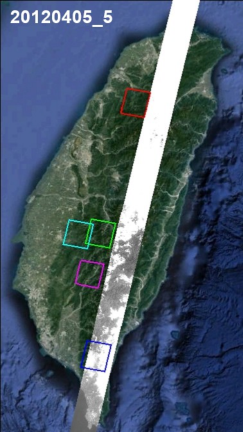
20120405_5

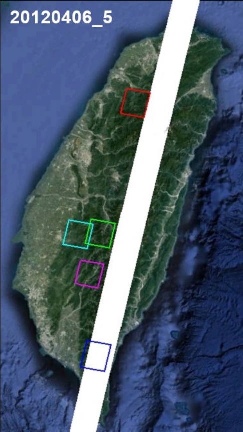
20120406_5

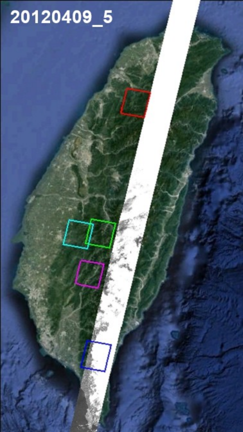
20120409_5

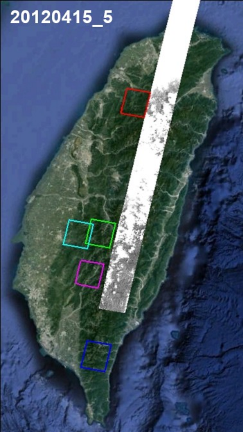
20120415_5

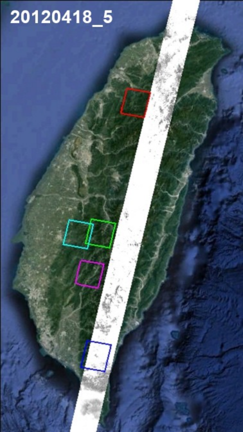
20120418_5

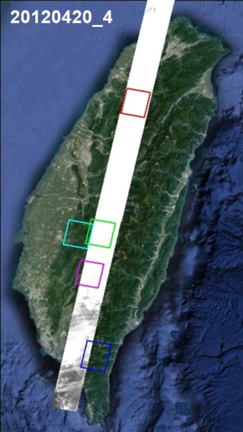
20120420_4

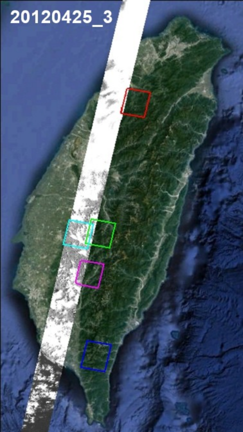
20120425_3

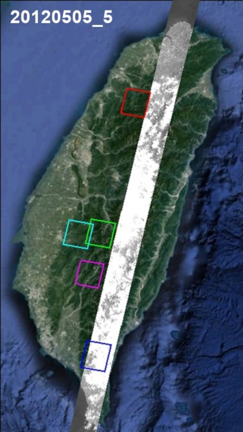
20120505_5

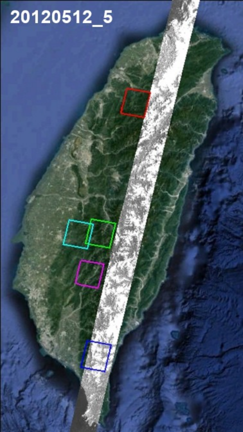
20120512_5

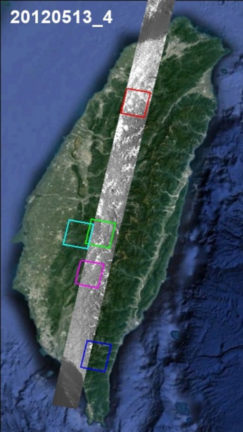
20120513_4

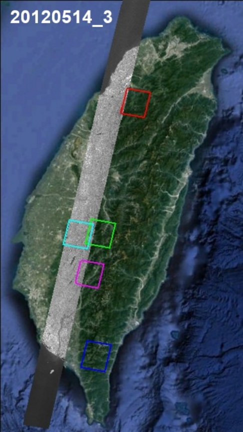
20120514_3

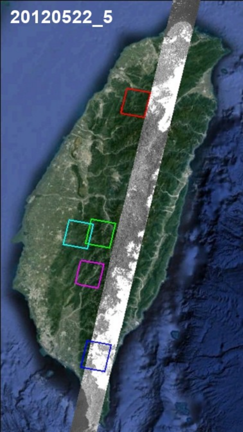
20120522_5

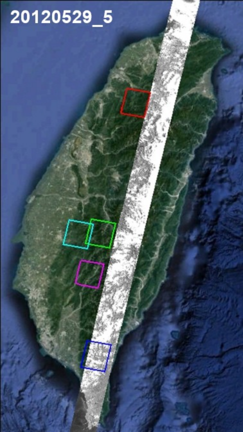
20120529_5

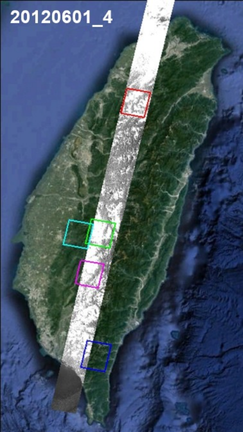
20120601_4

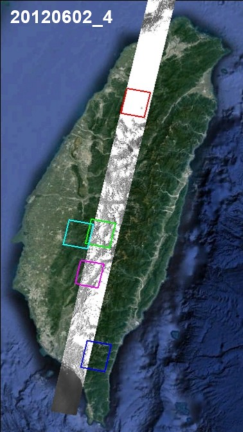
20120602_4

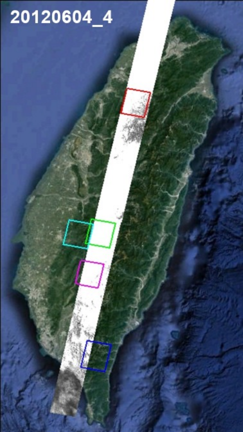
20120604_4

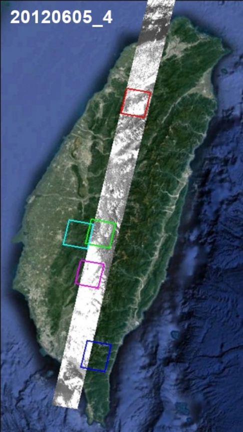
20120605_4

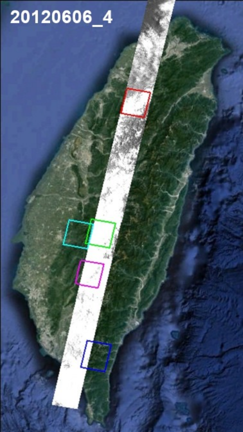
20120606_4

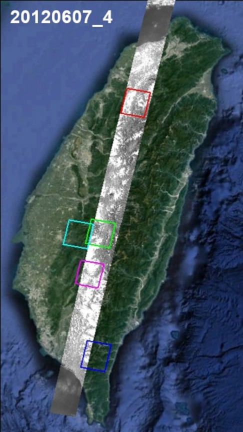
20120607_4

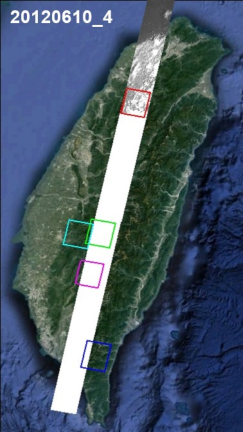
20120610_4

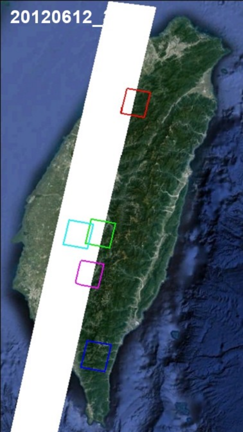
20120612_

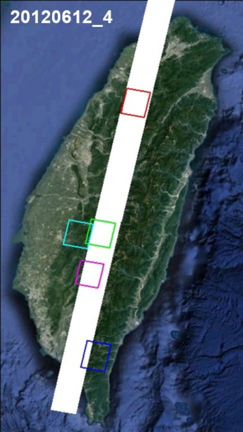
20120612_4

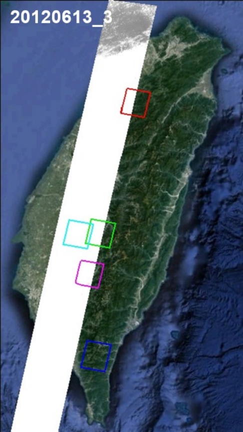
20120613_3

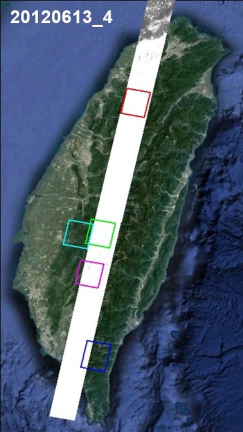
20120613_4

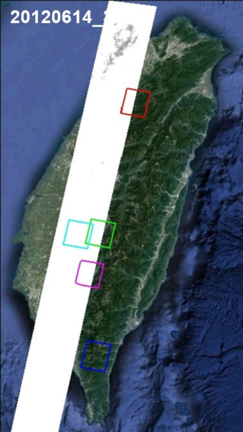
20120614_

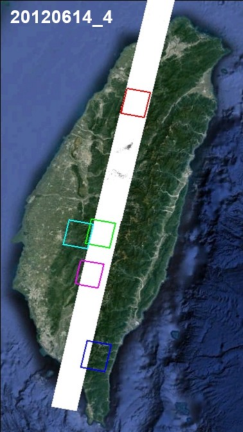
20120614_4

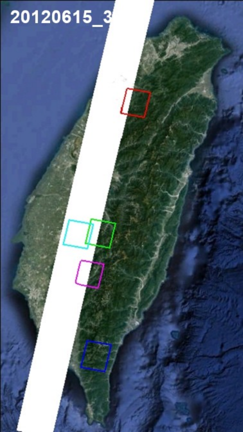
20120615_3

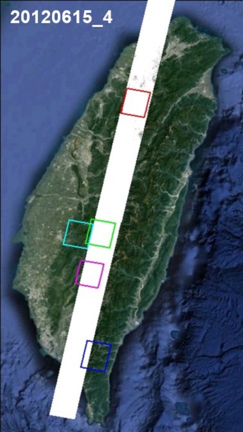
20120615_4

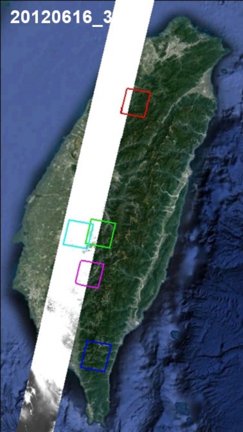
20120616_3

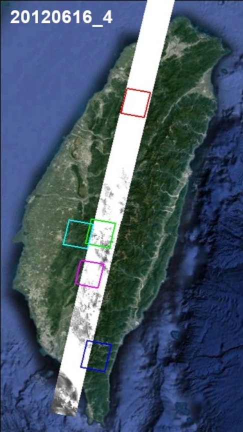
20120616_4

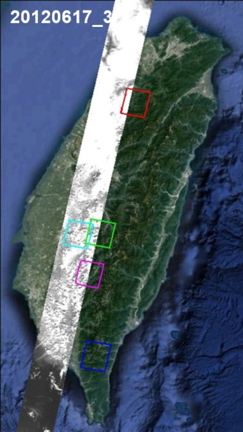
20120617_3

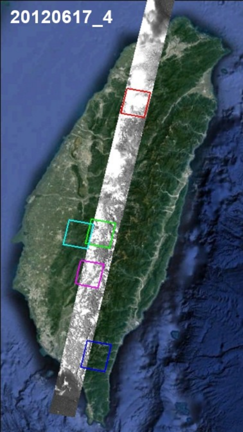
20120617_4

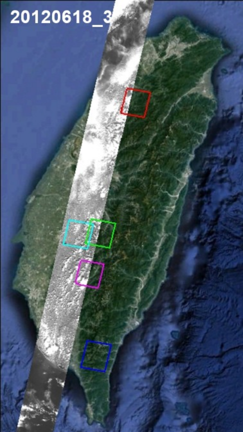
20120618_3

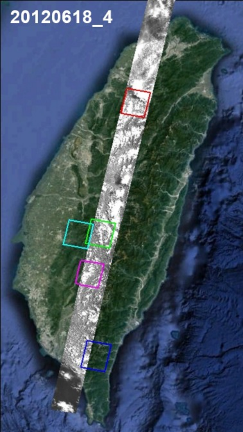
20120618_4

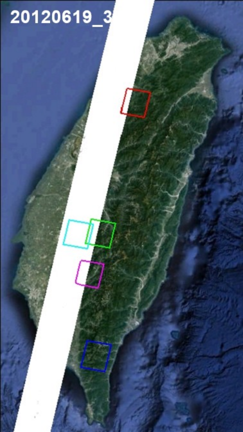
20120619_3

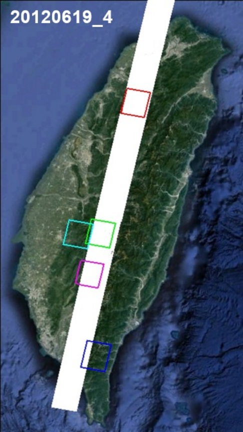
20120619_4

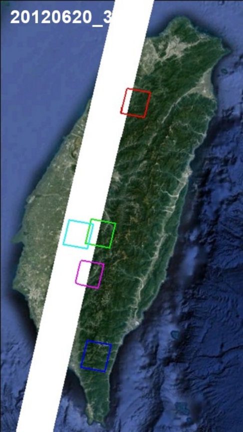
20120620_3

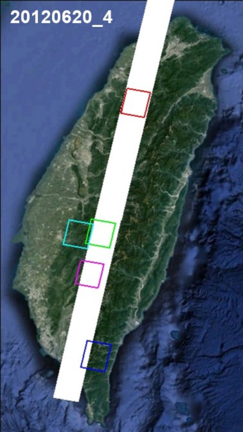
20120620_4

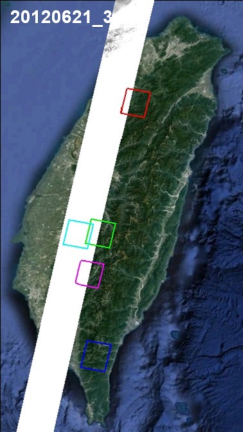
20120621_3

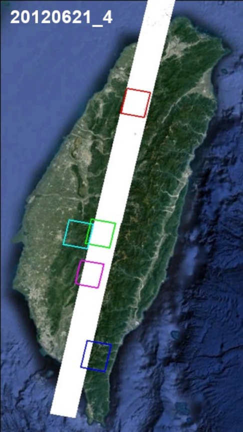
20120621_4

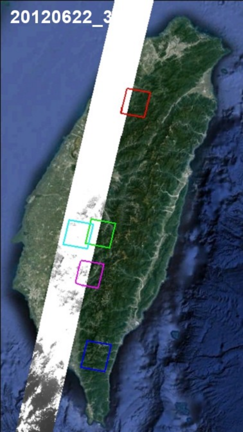
20120622_3

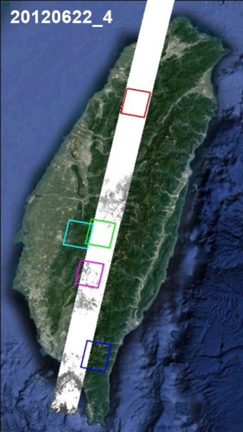
20120622_4

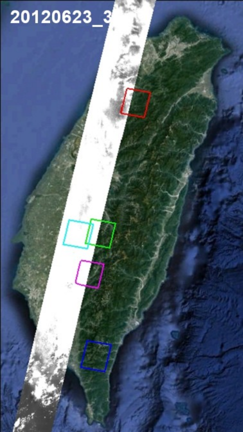
20120623_3

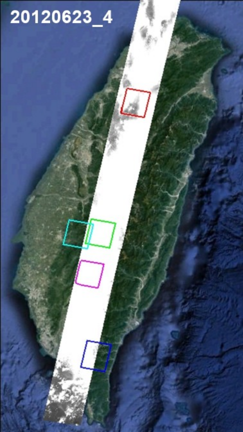
20120623_4

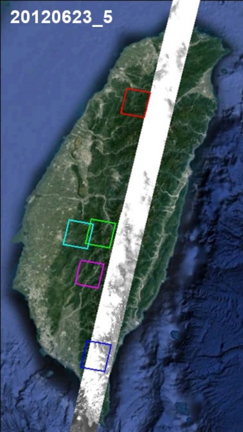
20120623_5

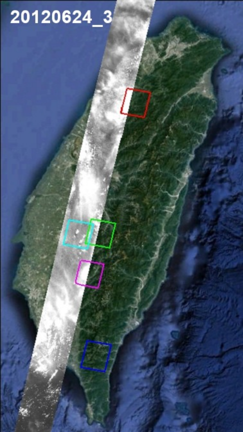
20120624_3

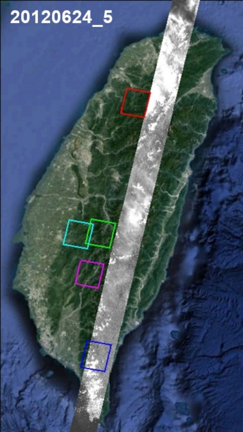
20120624_5

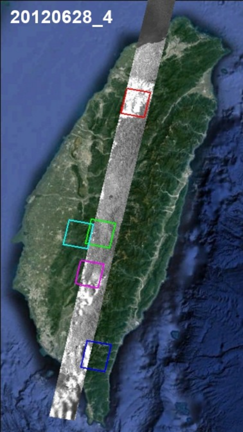
20120628_4

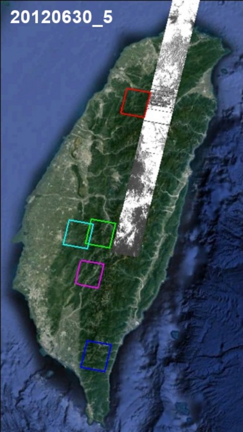
20120630_5

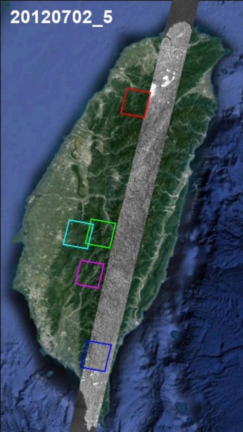
20120702_5

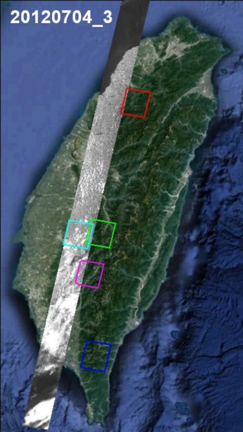
20120704_3

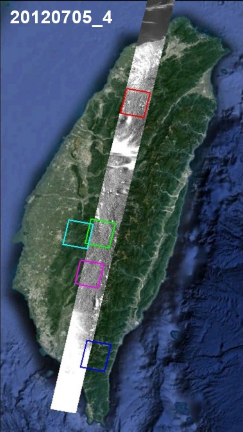
20120705_4

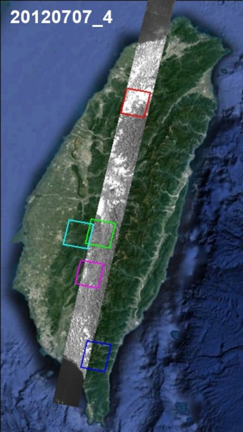
20120707_4

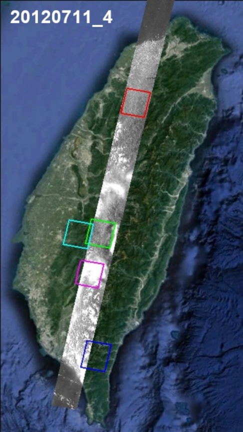
20120711_4

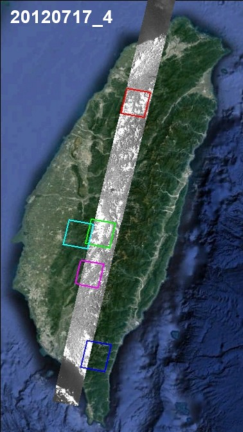
20120717_4

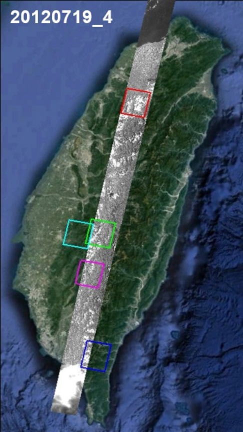
20120719_4

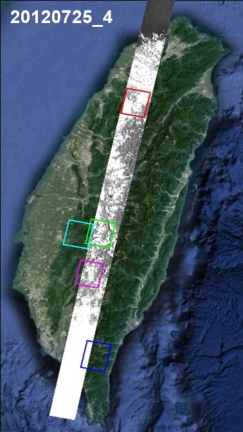
20120725_4

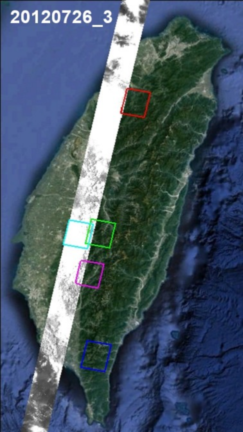
20120726_3

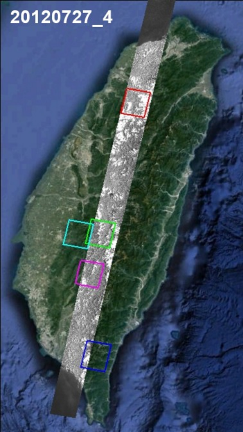
20120727_4

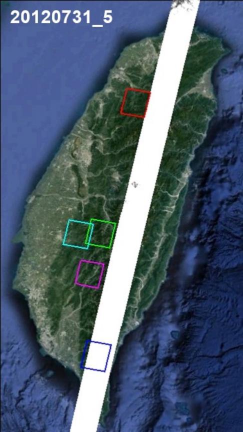
20120731_5

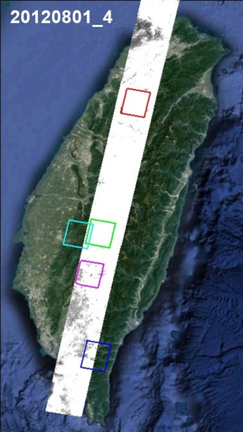
20120801_4

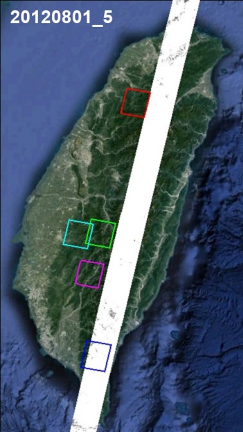
20120801_5

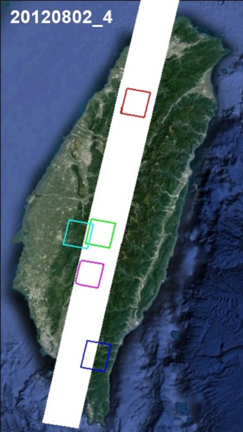
20120802_4

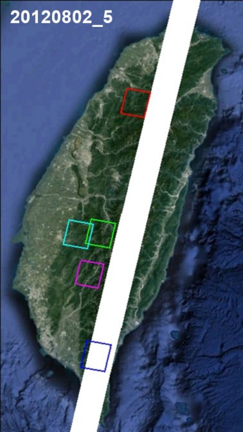
20120802_5

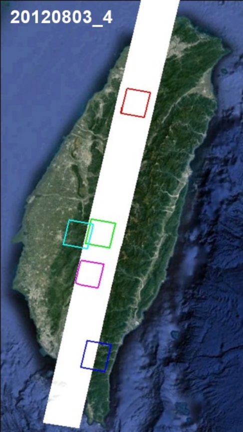
20120803_4

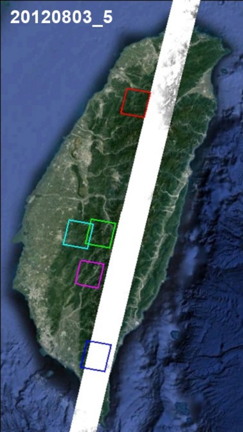
20120803_5

20120804_4

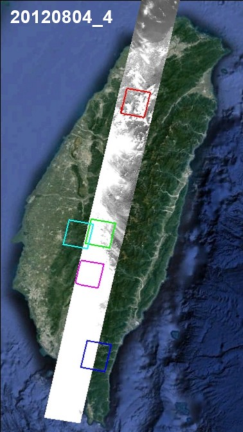

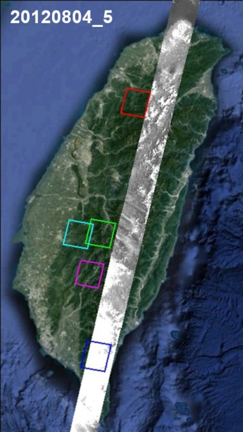
20120804_5

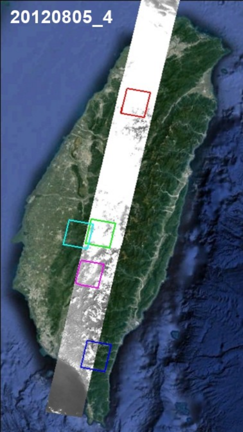
20120805_4

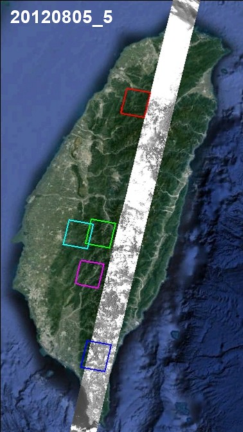
20120805_5

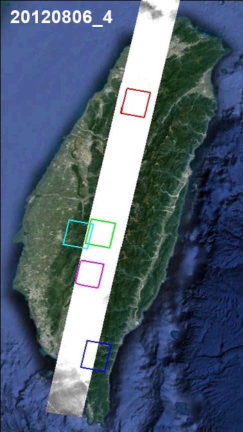
20120806_4

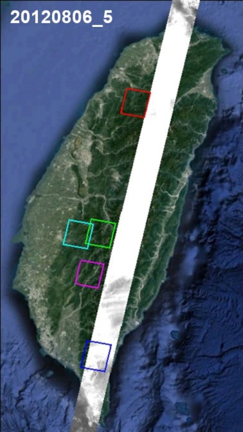
20120806_5

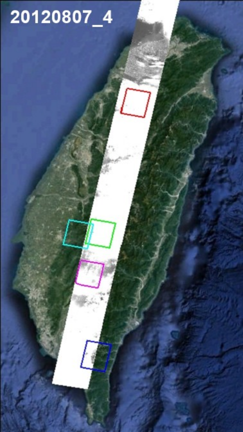
20120807_4

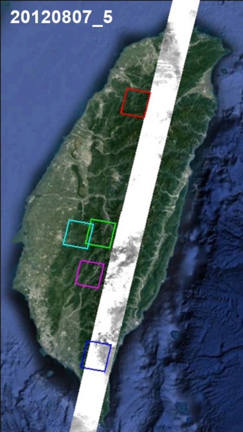
20120807_5

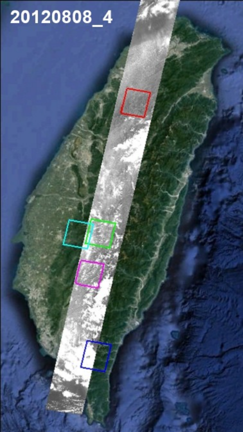
20120808_4

20120808_5

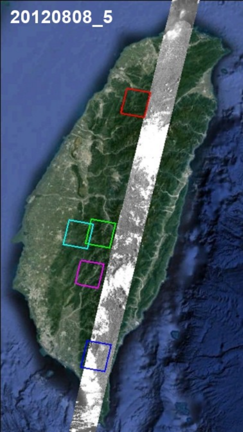

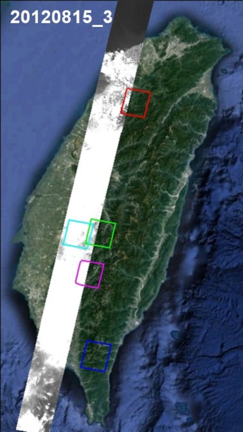
20120815_3

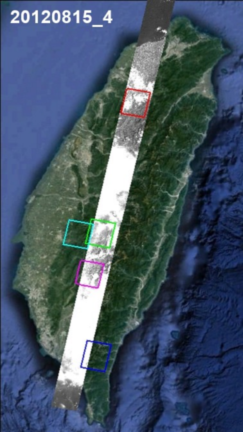
20120815_4

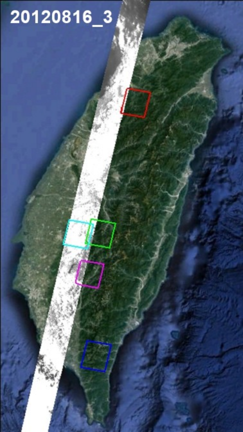
20120816_3

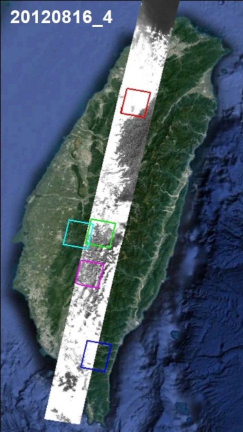
20120816_4

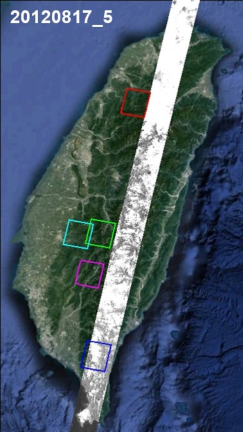
20120817_5

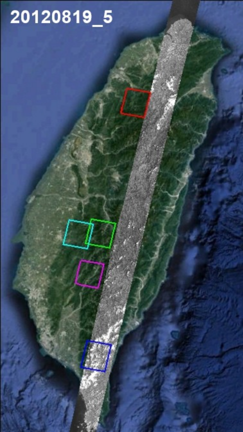
20120819_5

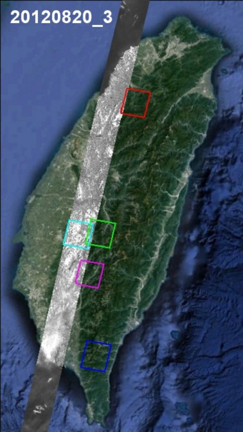
20120820_3

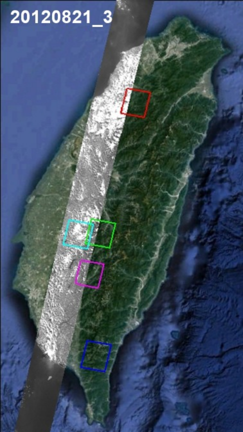
20120821_3

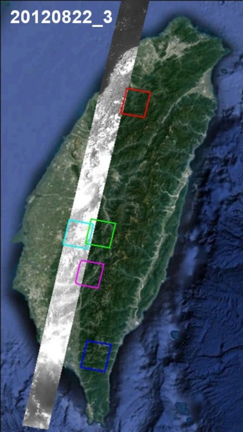
20120822_3

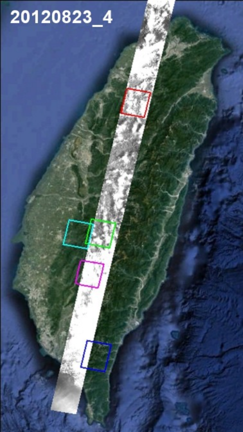
20120823_4

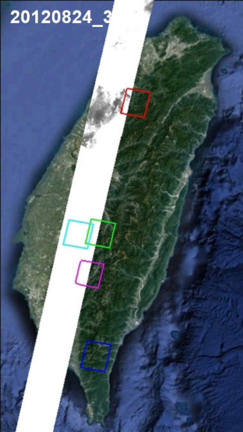
20120824_3

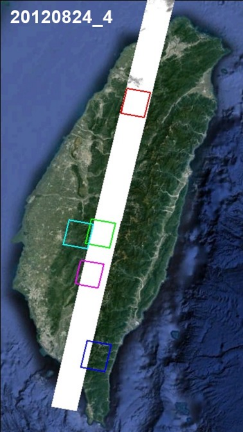
20120824_4

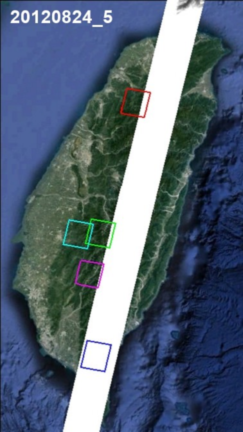
20120824_5

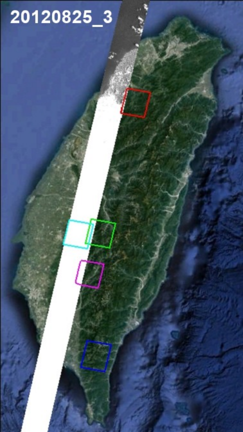
20120825_3

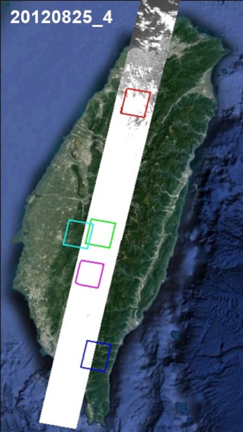
20120825_4

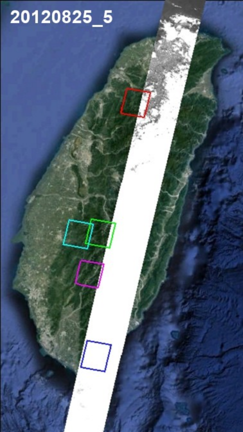
20120825_5

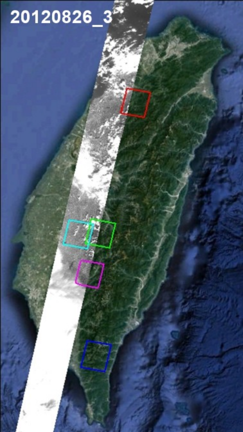
20120826_3

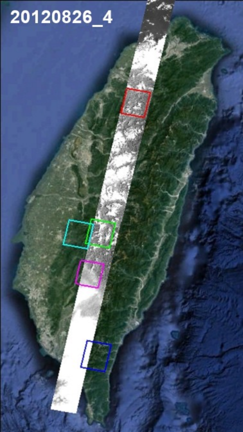

20120826_4

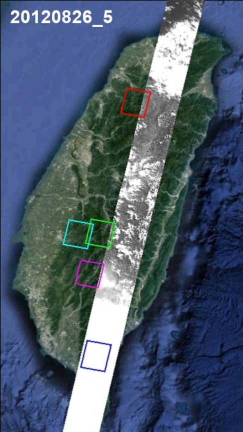
20120826_5

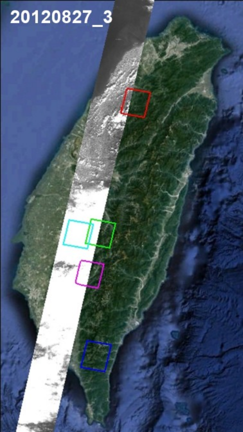
20120827_3

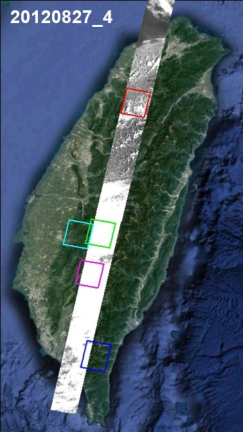
20120827_4

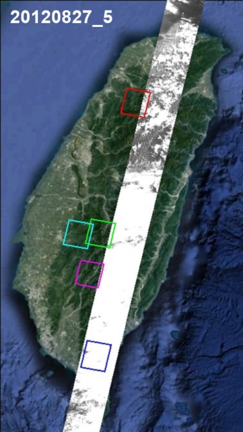
20120827_5

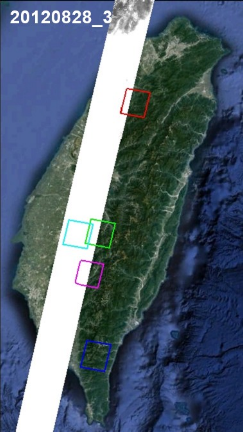
20120828_3

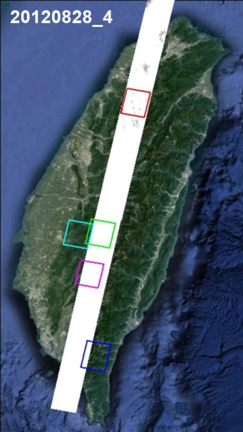
20120828_4

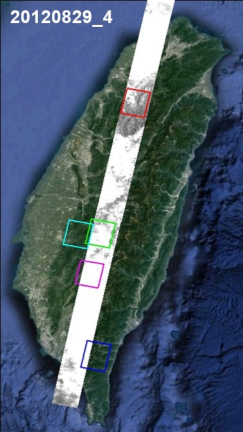
20120829_4

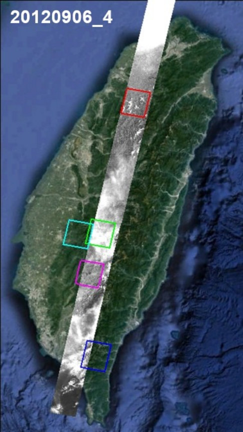
20120906_4

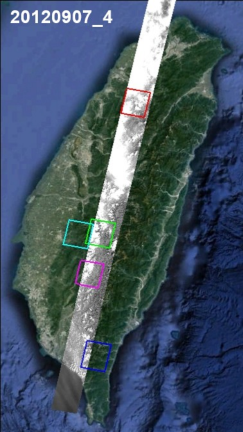
20120907_4

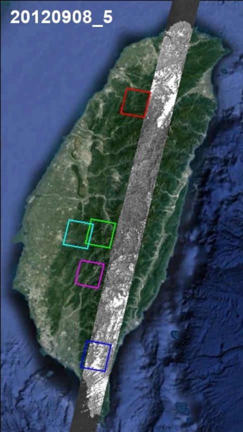
20120908_5

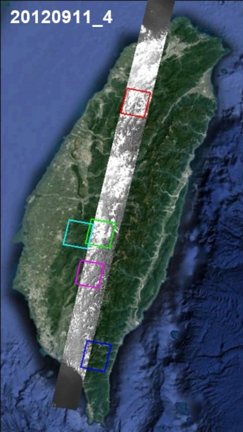
20120911_4

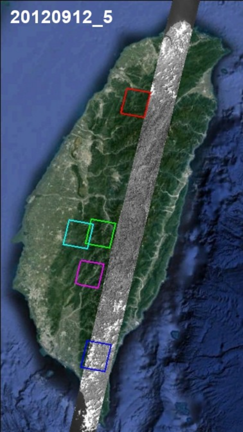
20120912_5

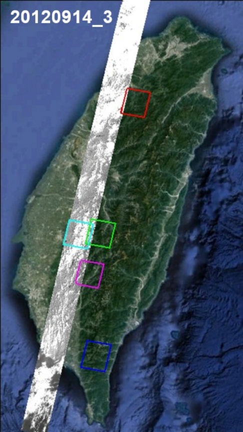
20120914_3

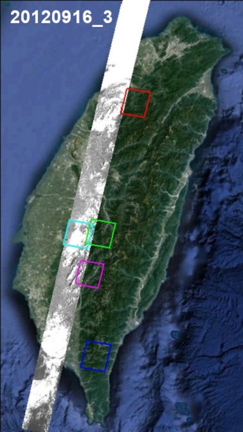
20120916_3

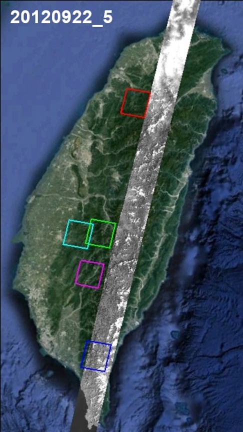
20120922_5

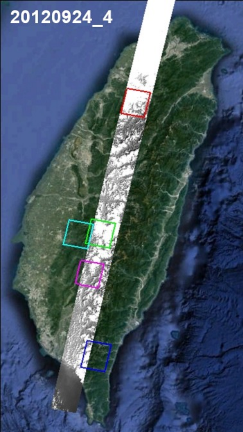
20120924_4

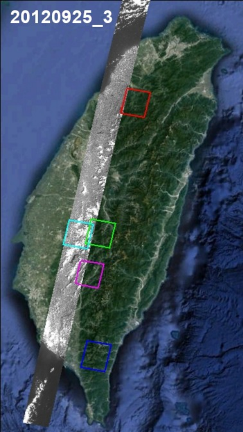
20120925_3

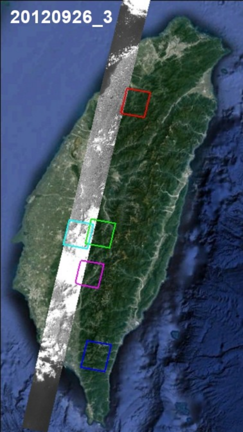
20120926_3

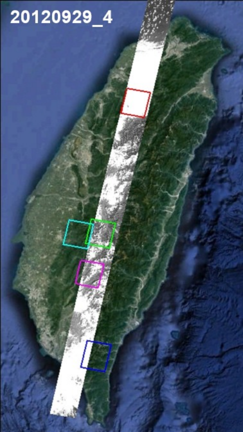
20120929_4

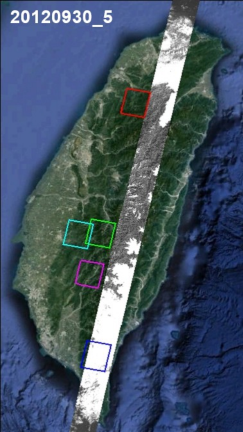
20120930_5

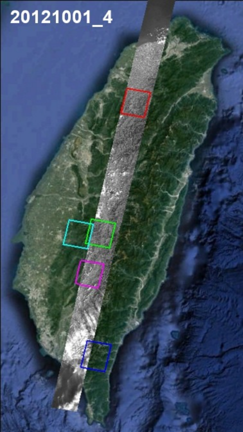
20121001_4

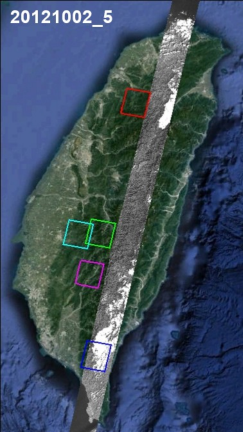
20121002_5

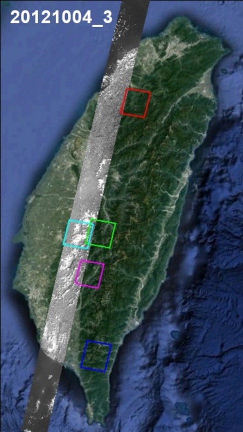
20121004_3

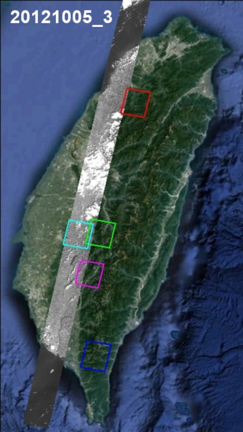
20121005_3

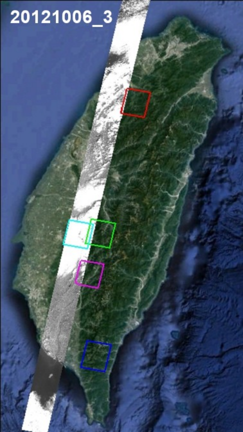
20121006_3

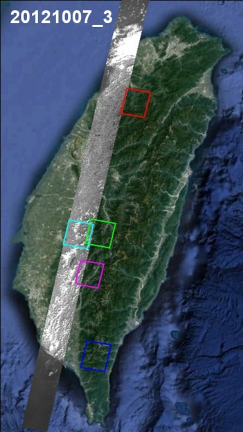
20121007_3

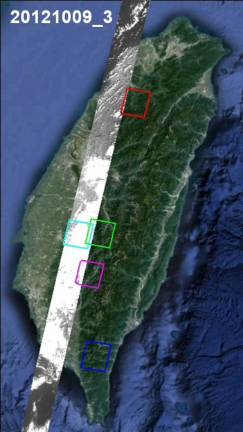
20121009_3

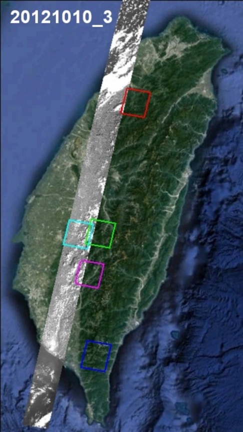
20121010_3

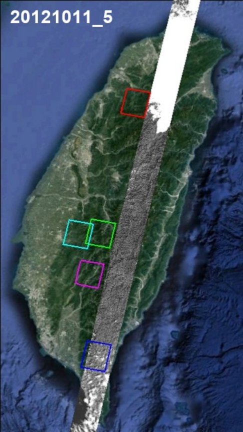
20121011_5

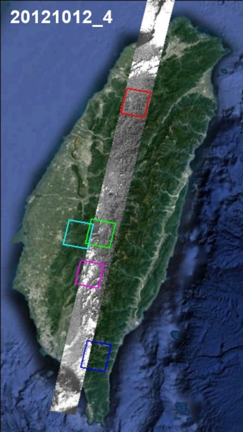
20121012_4

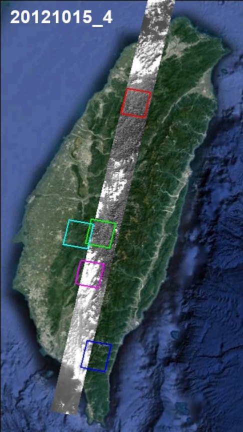
20121015_4

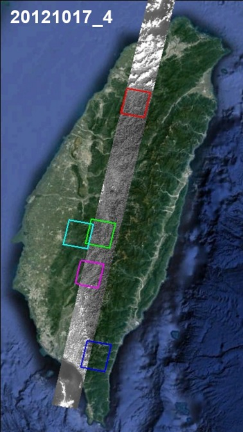
20121017_4

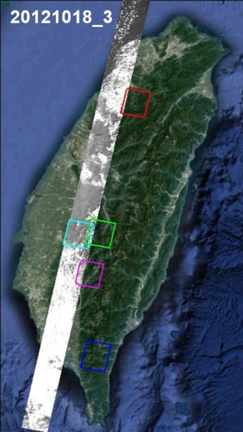
20121018_3

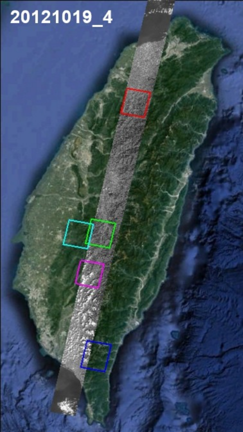
20121019_4

20121020_4

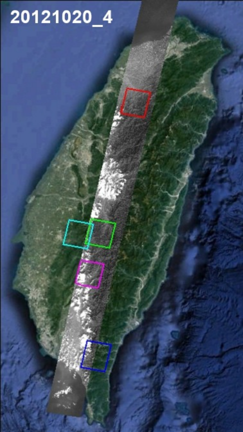

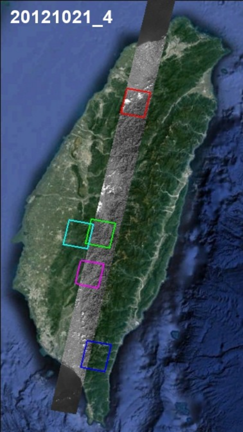
20121021_4

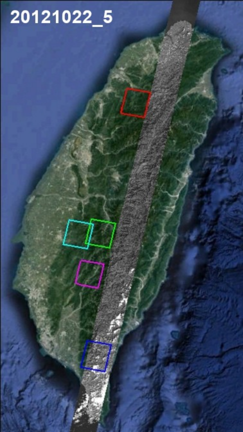
20121022_5

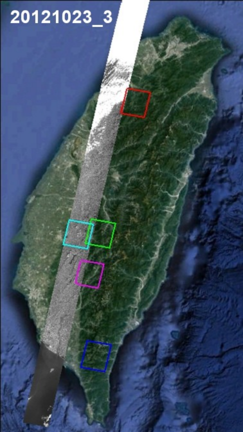
20121023_3

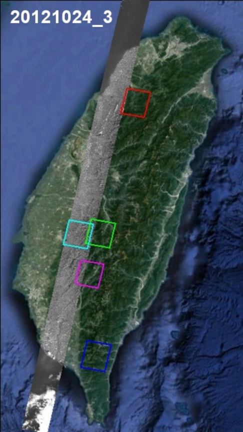
20121024_3

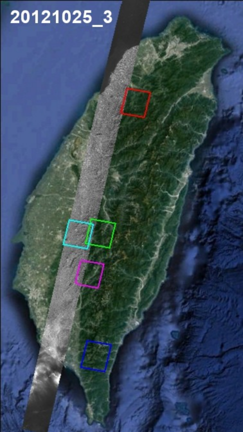
20121025_3

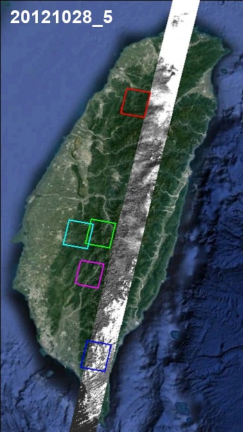
20121028_5

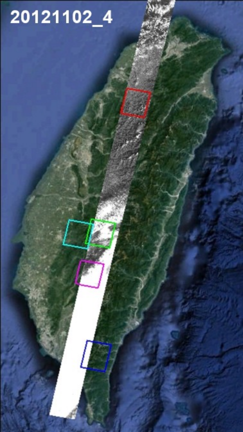
20121102_4

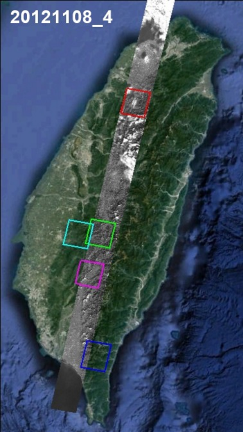
20121108_4

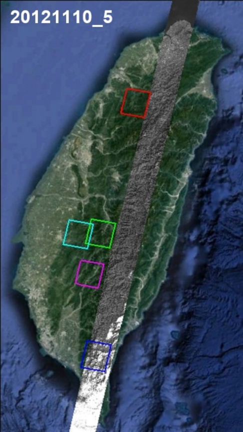
20121110_5

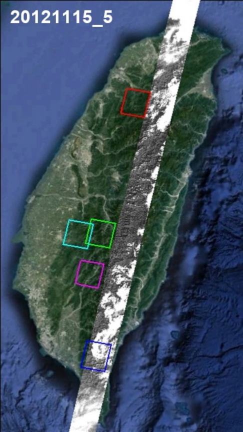
20121115_5

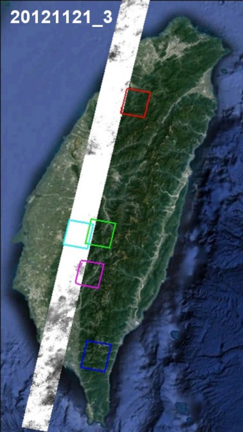
20121121_3

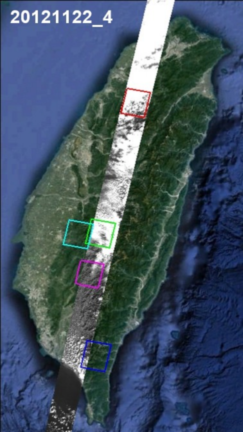
20121122_4

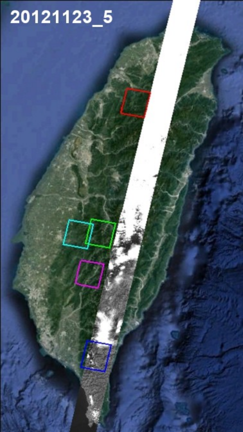
20121123_5

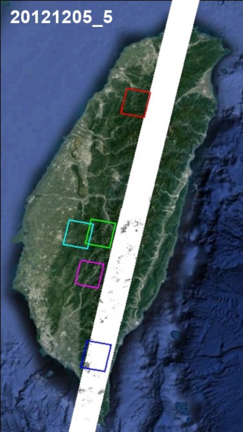
20121205_5

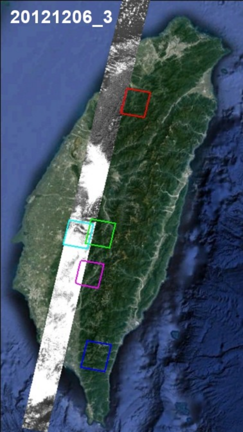
20121206_3

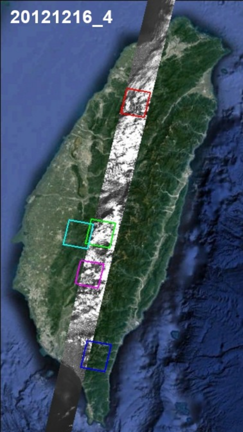
20121216_4

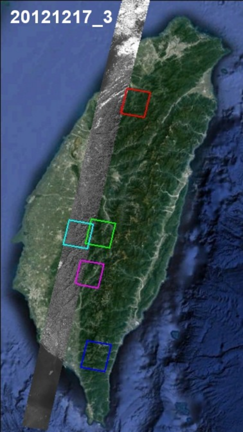
20121217_3

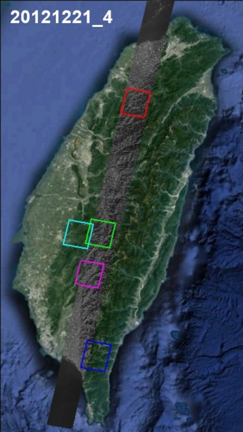
20121221_4

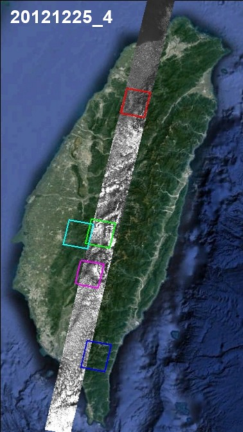
20121225_4

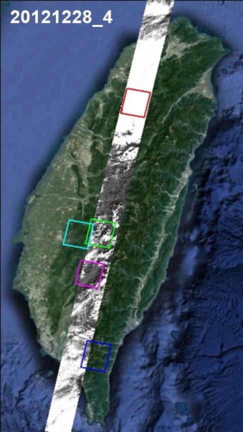
20121228_4

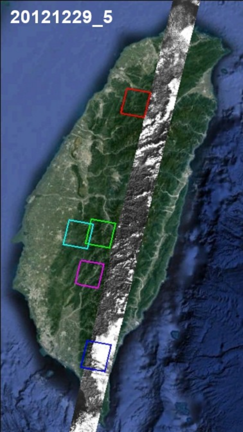
20121229_5

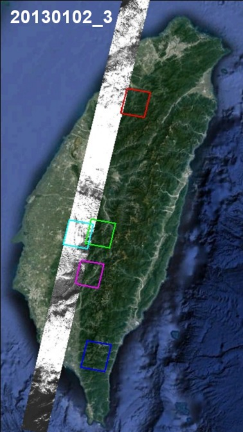
20130102_3

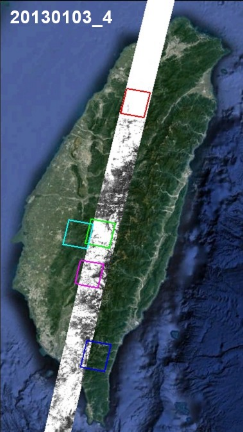
20130103_4

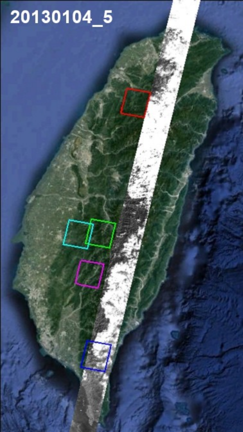
20130104_5

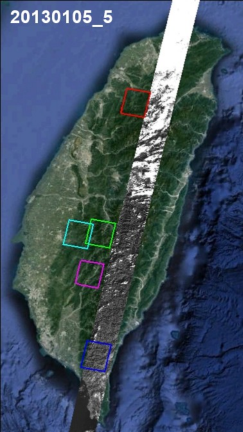
20130105_5

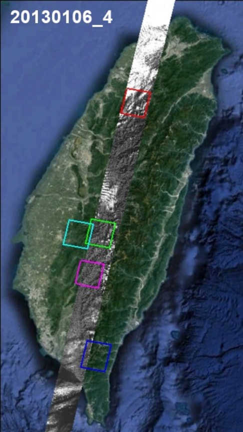
20130106_4

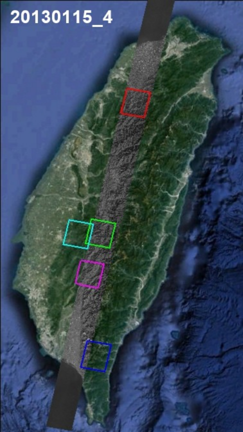
20130115_4

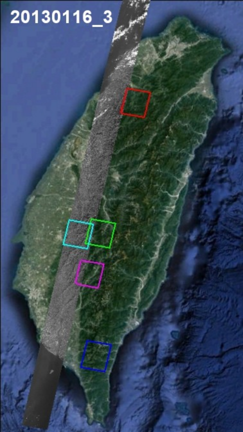
20130116_3

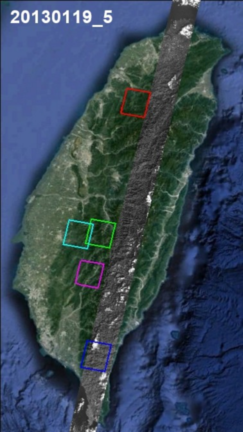
20130119_5

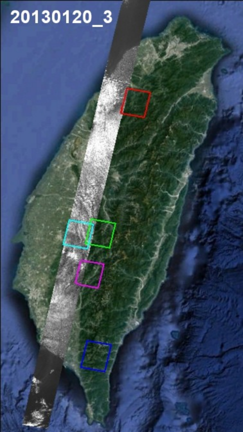
20130120_3

20130121_3

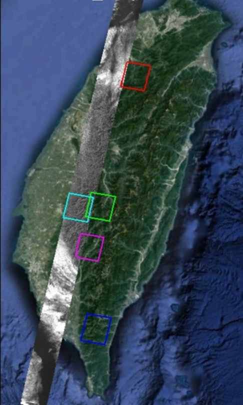

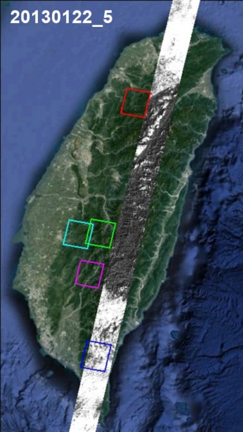
20130122_5

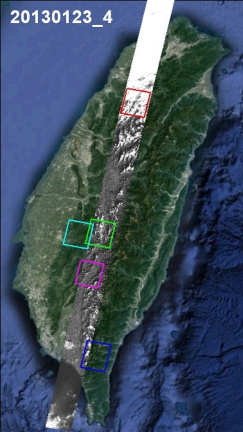
20130123_4

20130128_3

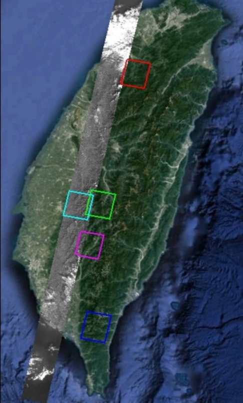

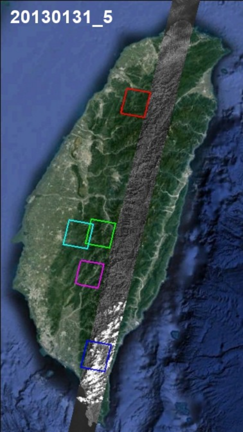
20130131_5

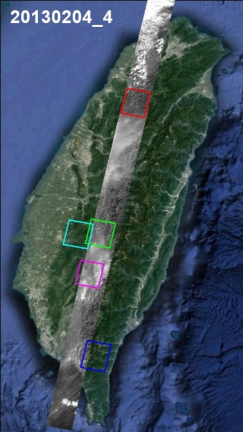
20130204_4

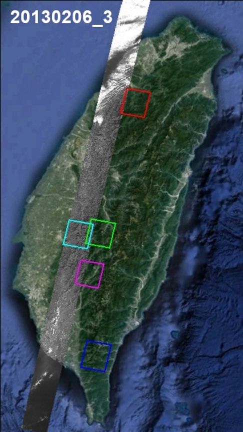
20130206_3

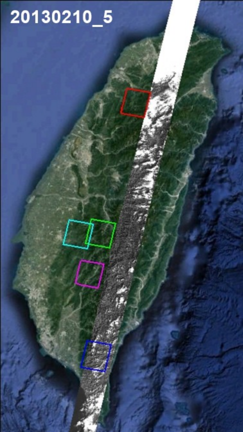
20130210_5

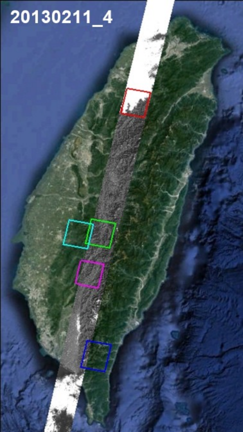
20130211_4

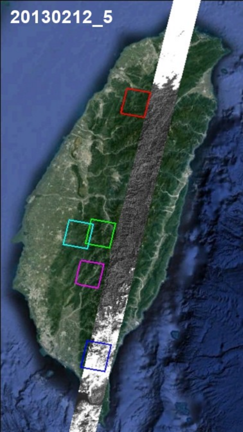
20130212_5

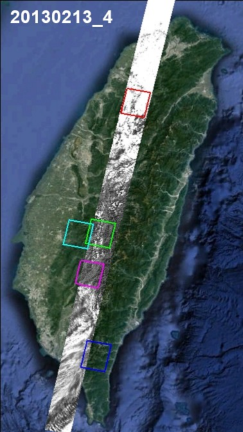
20130213_4

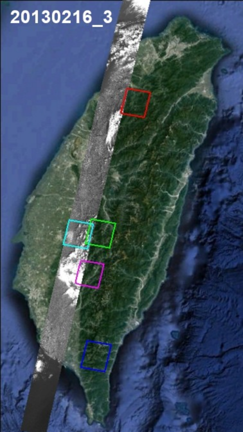
20130216_3

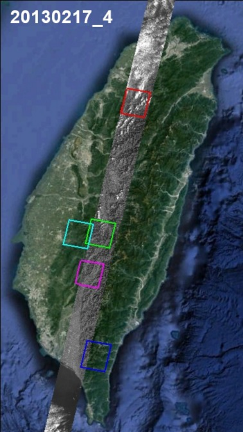
20130217_4

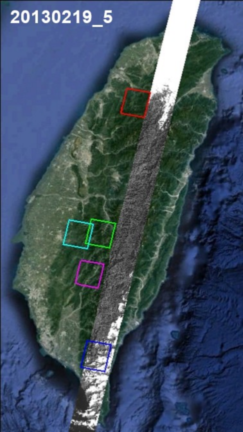
20130219_5

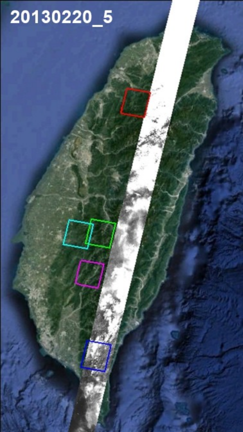
20130220_5

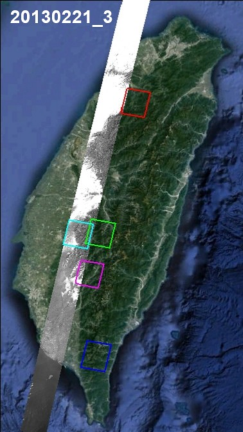
20130221_3

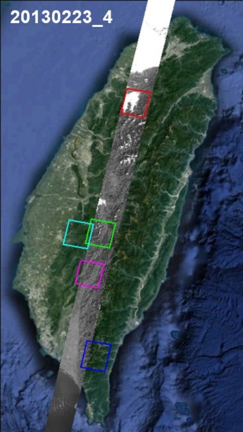
20130223_4

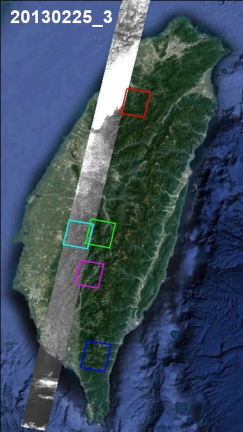
20130225_3

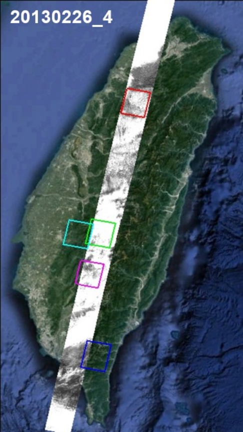
20130226_4

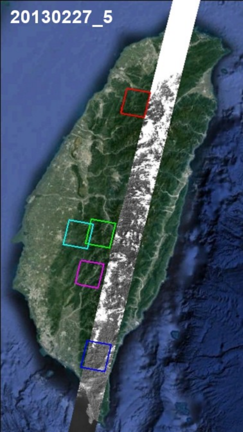
20130227_5

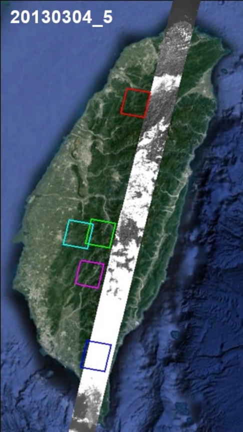
20130304_5

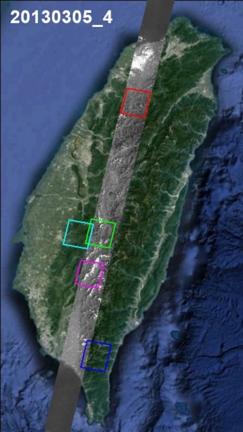
20130305_4

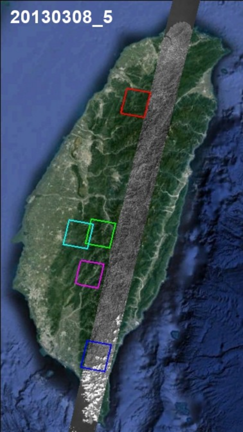
20130308_5

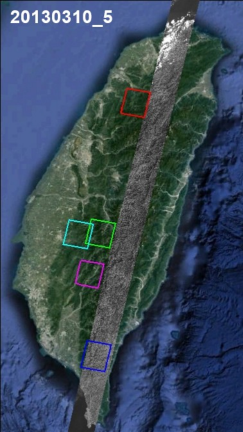
20130310_5

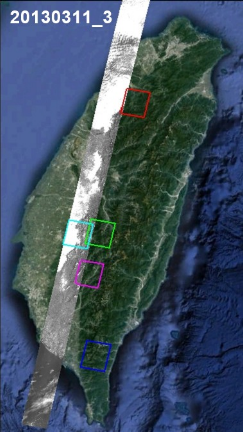
20130311_3

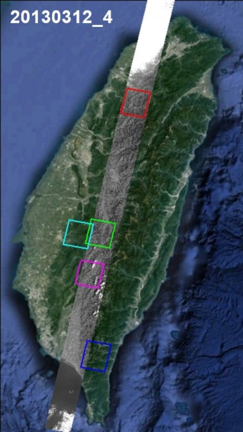
20130312_4

20130313_5

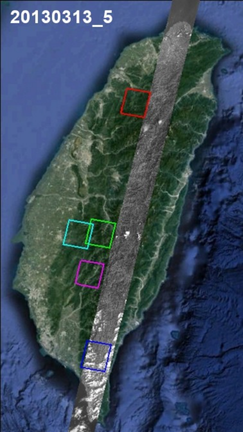

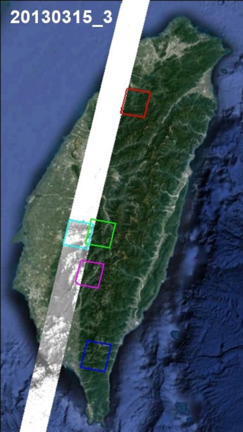
20130315_3

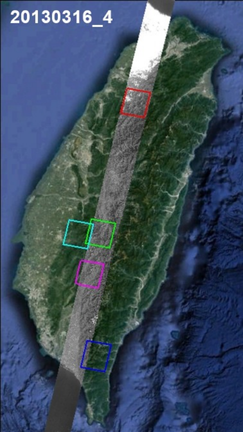
20130316_4

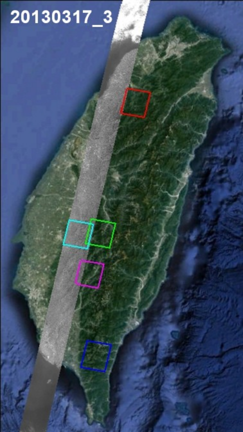
20130317_3

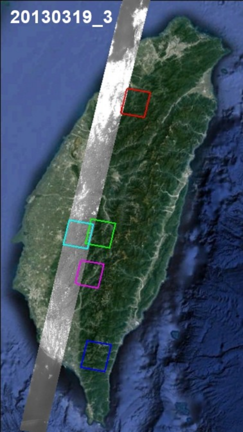
20130319_3

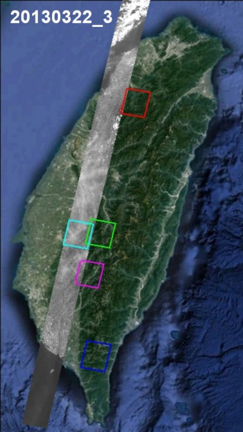
20130322_3

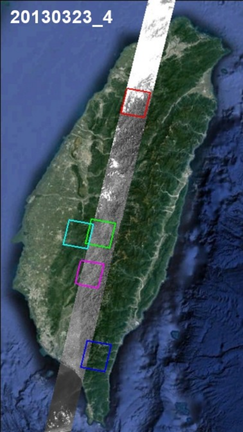
20130323_4

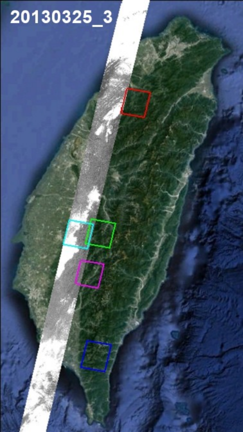
20130325_3

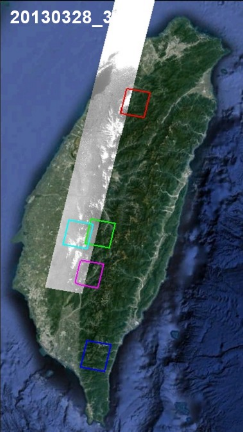
20130328_3

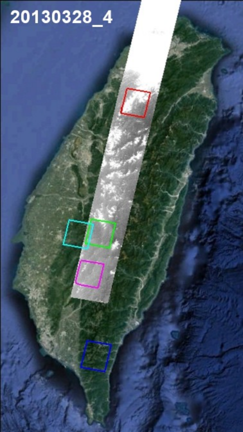
20130328_4

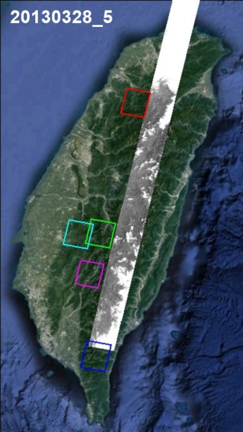
20130328_5

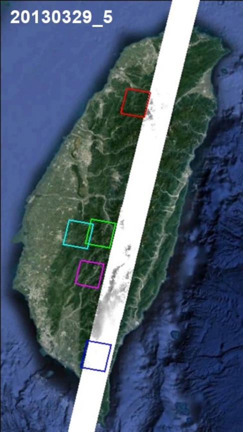
20130329_5

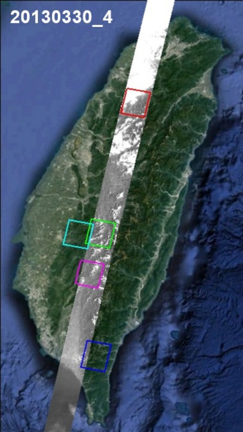
20130330_4

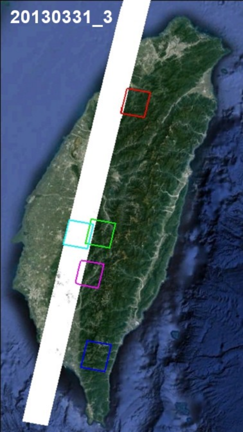
20130331_3

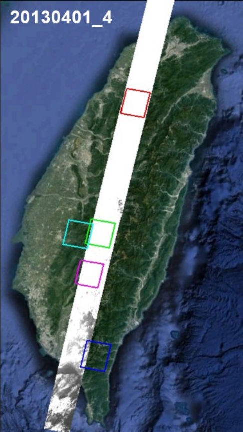
20130401_4

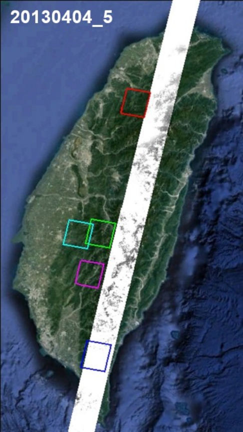
20130404_5

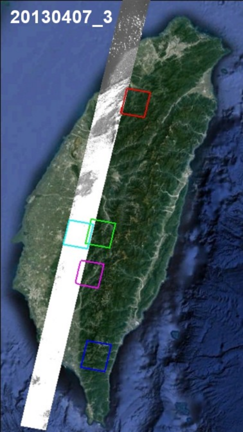
20130407_3

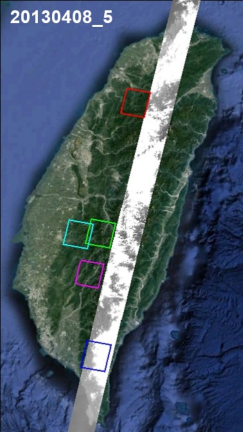
20130408_5

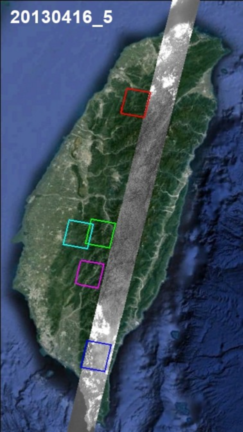
20130416_5

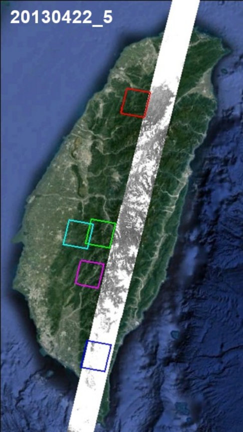
20130422_5

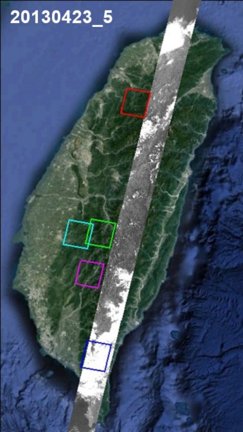
20130423_5

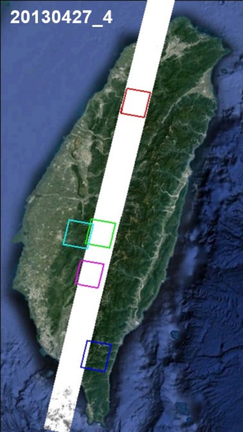
20130427_4

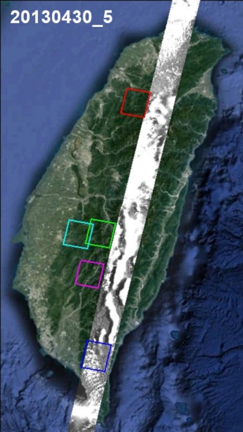
20130430_5

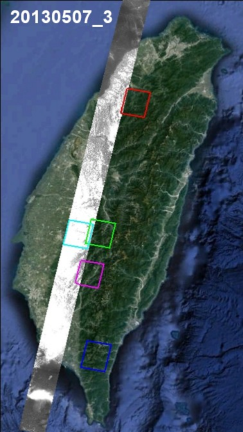
20130507_3

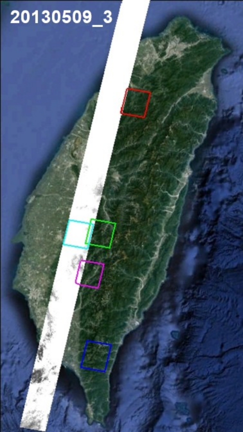
20130509_3

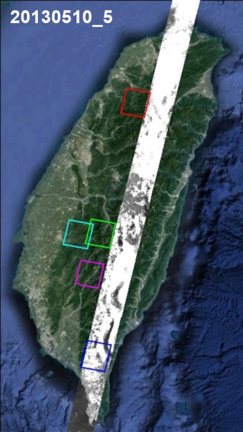
20130510_5

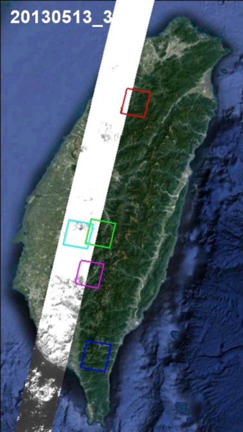
20130513_3

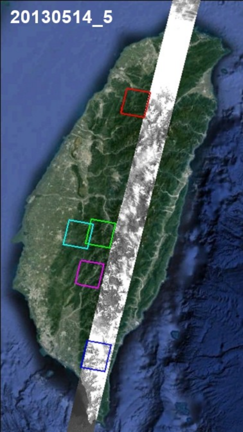
20130514_5

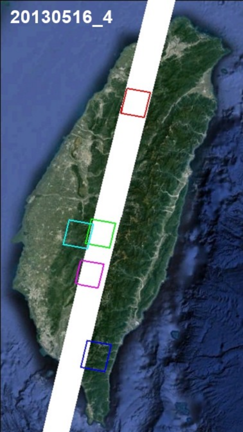
20130516_4

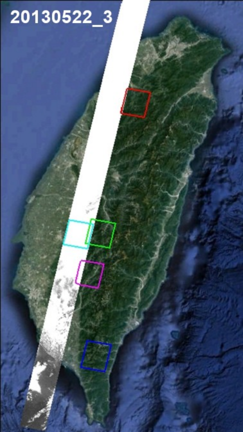
20130522_3

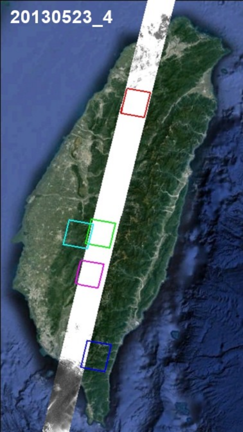
20130523_4

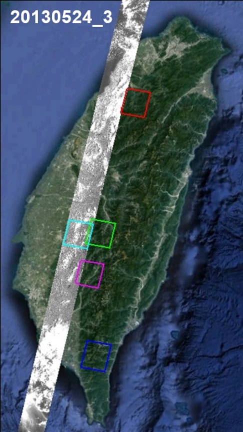
20130524_3

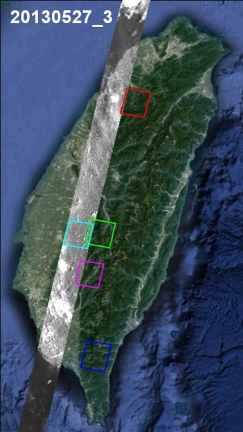
20130527_3

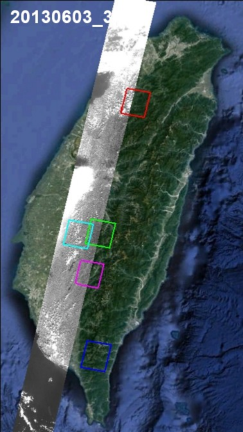
20130603_3

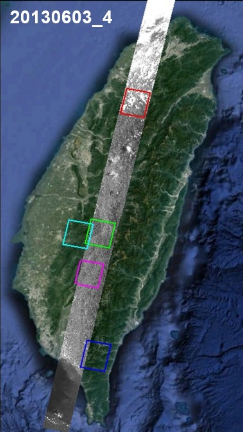
20130603_4

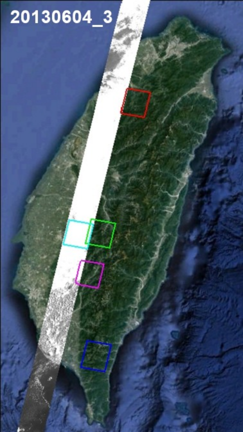
20130604_3

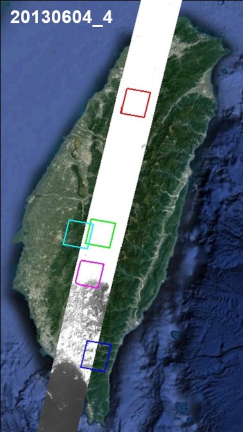
20130604_4

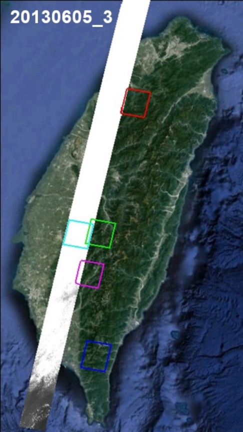
20130605_3

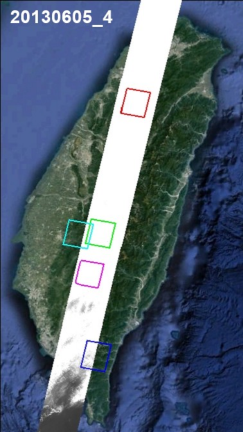
20130605_4

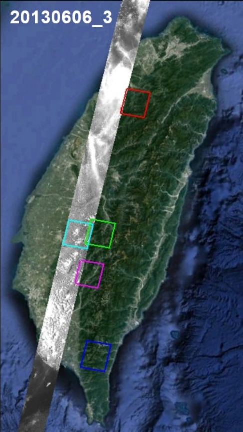
20130606_3

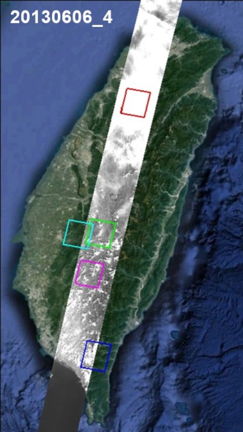
20130606_4

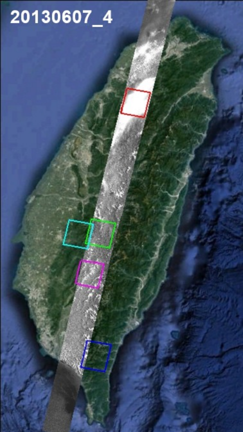
20130607_4

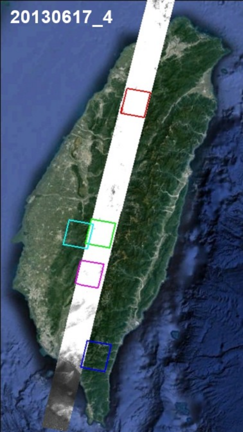
20130617_4

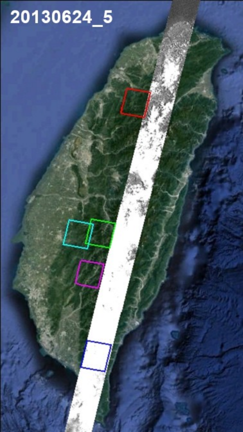
20130624_5

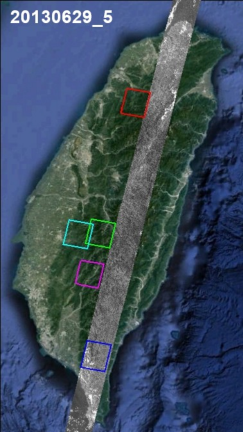
20130629_5

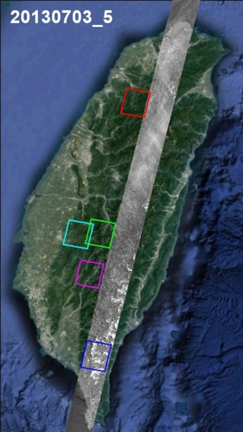
20130703_5

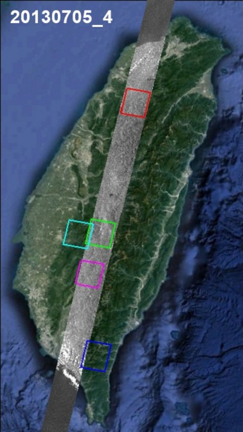
20130705_4

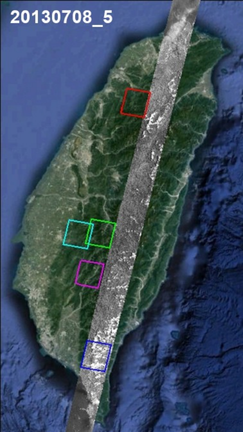
20130708_5

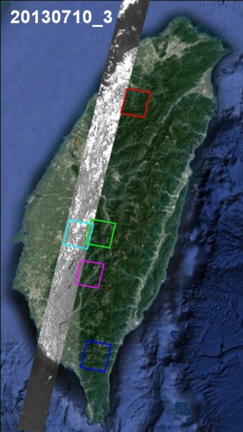
20130710_3

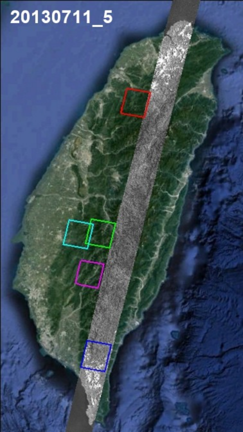
20130711_5

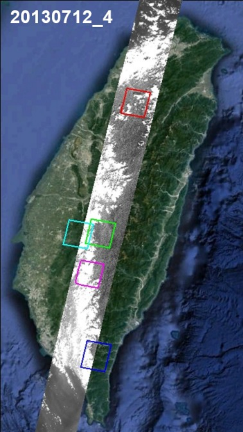
20130712_4

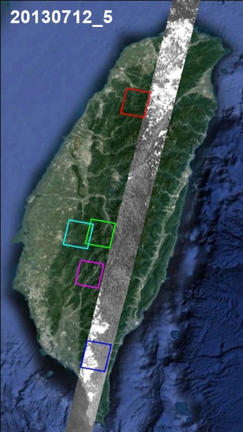
20130712_5

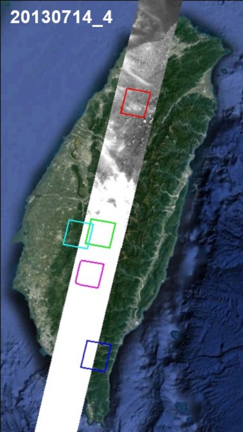
20130714_4

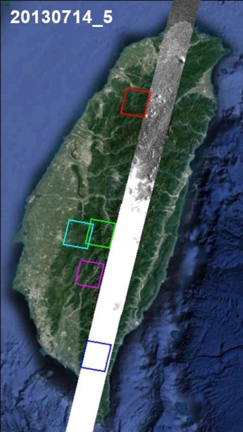
20130714_5

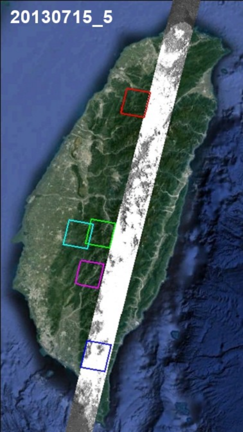
20130715_5

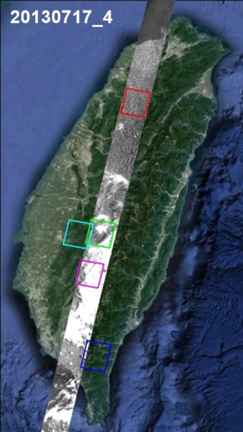
20130717_4

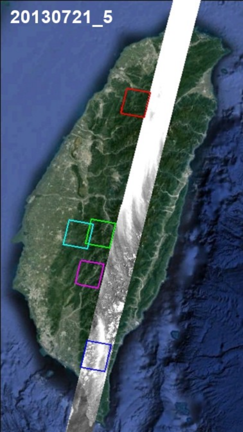
20130721_5

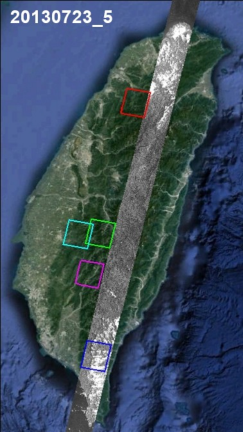
20130723_5

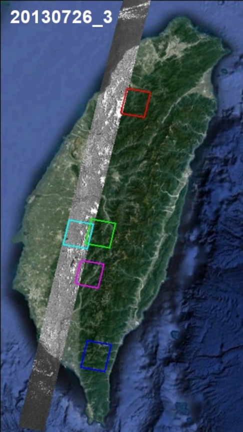
20130726_3

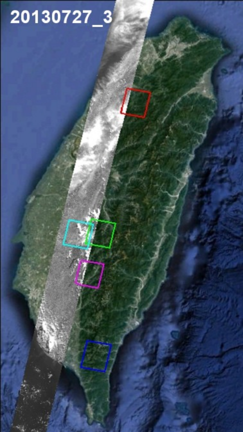
20130727_3

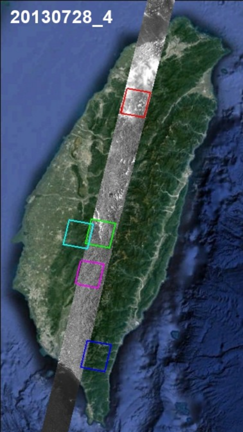
20130728_4

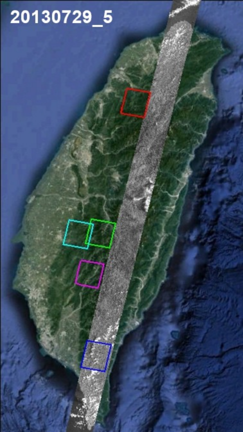
20130729_5

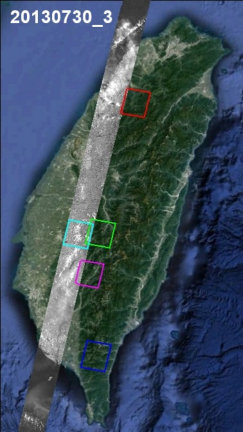
20130730_3

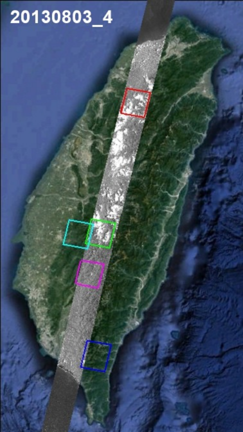
20130803_4

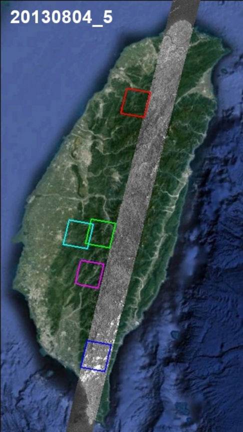
20130804_5

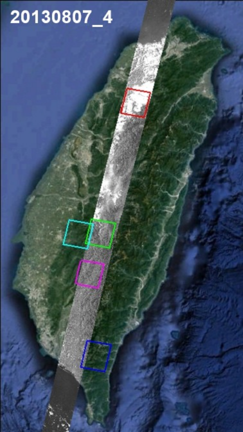
20130807_4

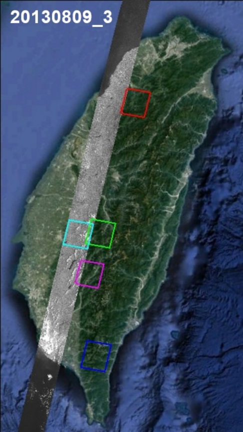
20130809_3

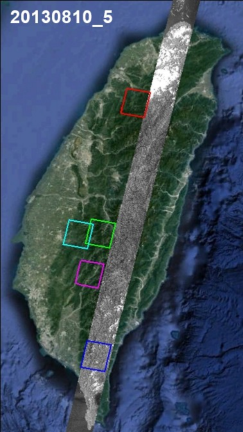
20130810_5

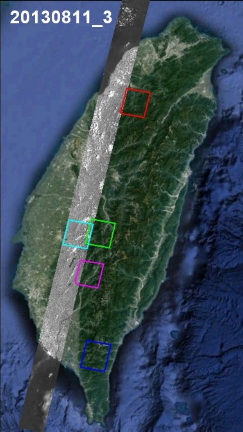
20130811_3

20130812_5

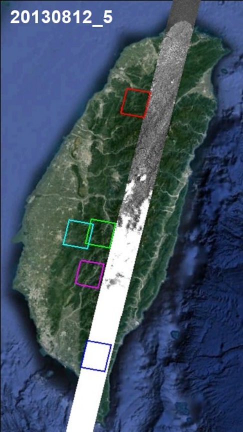

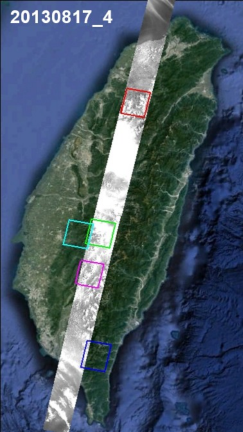
20130817_4

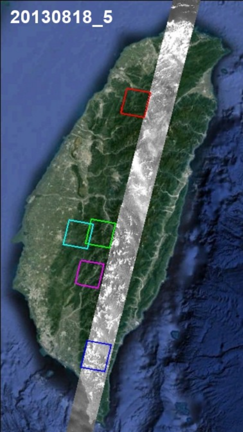
20130818_5

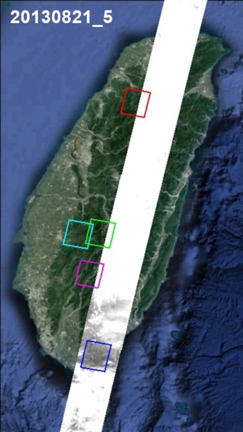
20130821_5

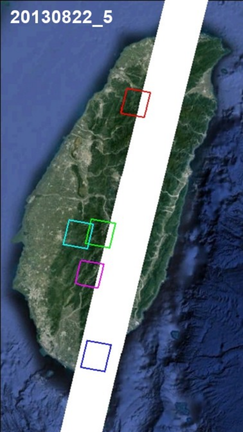
20130822_5

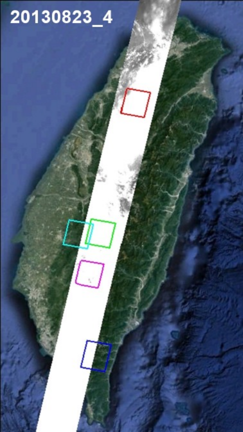
20130823_4

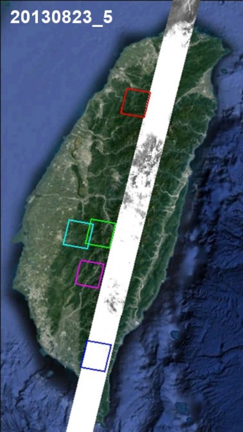
20130823_5

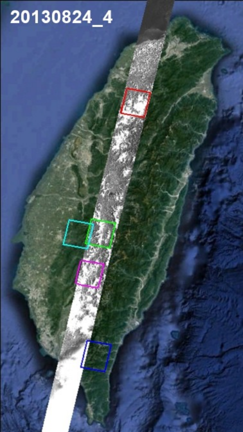
20130824_4

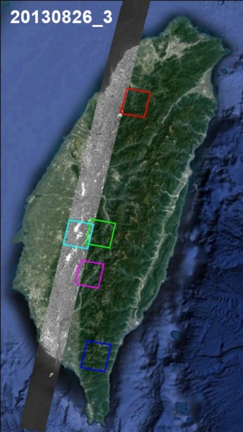
20130826_3

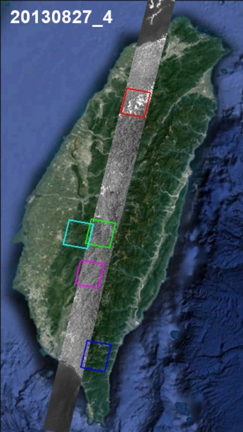
20130827_4

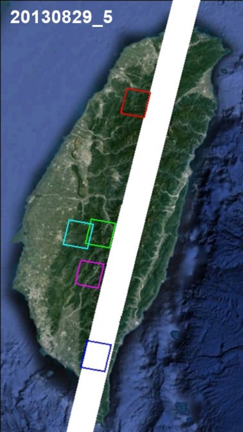
20130829_5

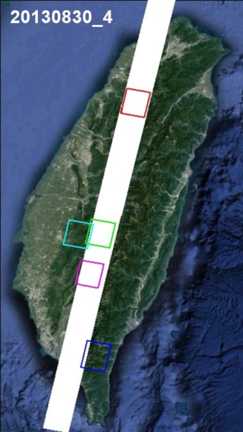
20130830_4

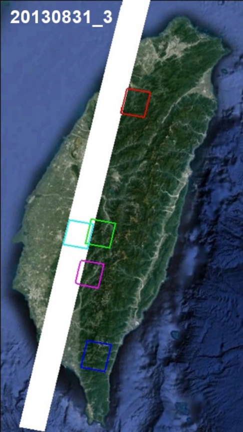
20130831_3

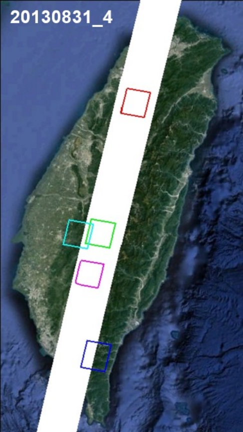
20130831_4

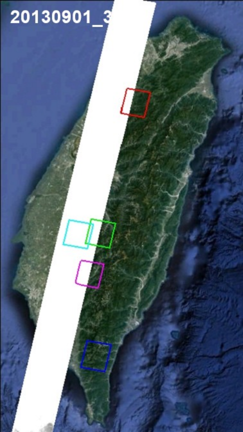
20130901_3

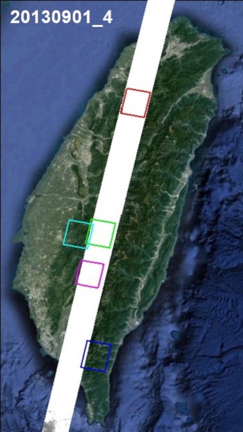
20130901_4

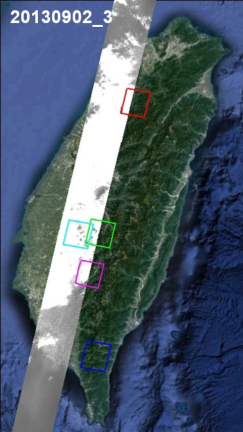
20130902_3

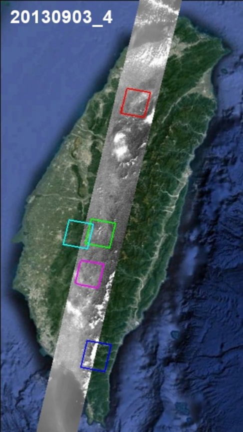
20130903_4

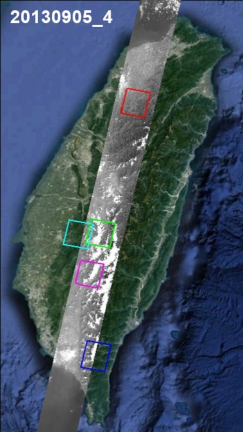
20130905_4

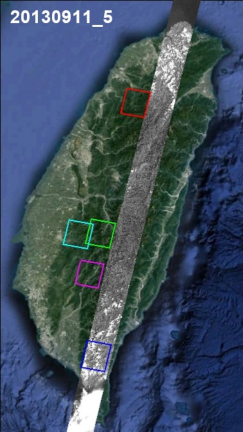
20130911_5

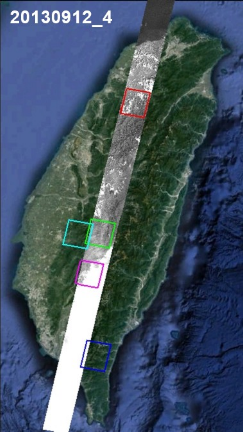
20130912_4

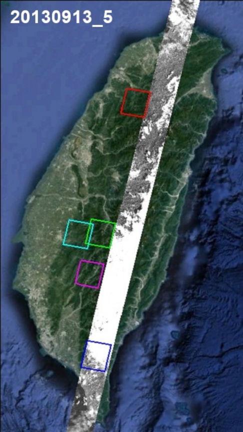
20130913_5

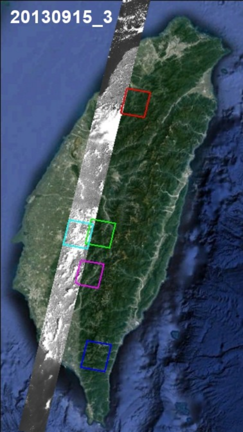
20130915_3

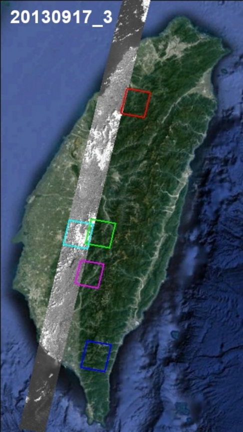
20130917_3

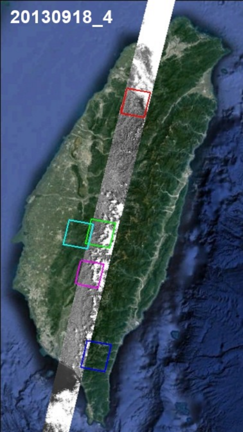
20130918_4

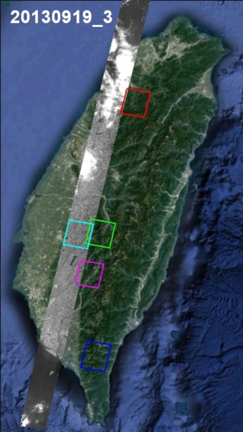
20130919_3

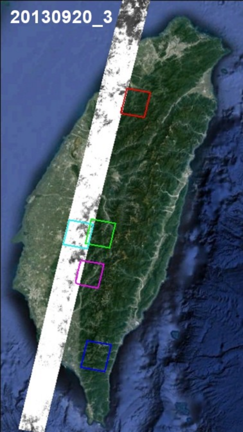
20130920_3

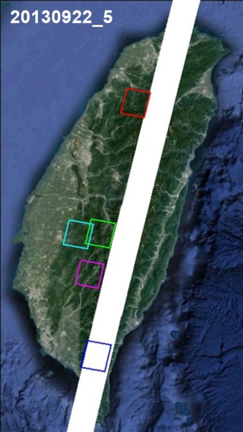
20130922_5

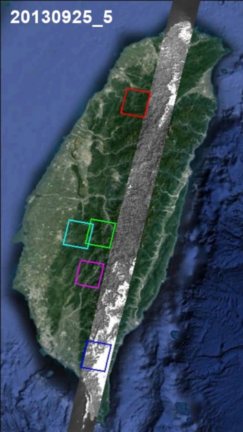
20130925_5

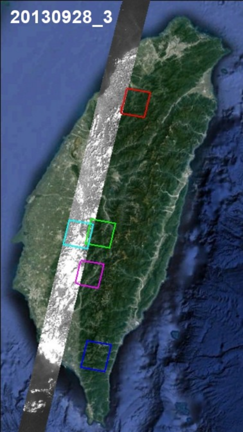
20130928_3

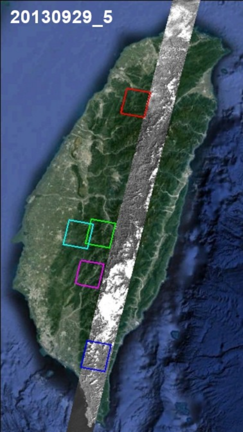
20130929_5

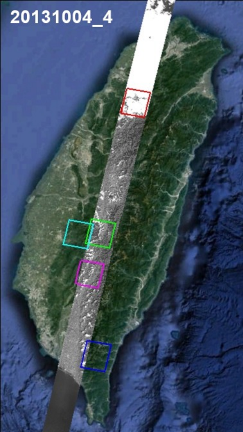
20131004_4

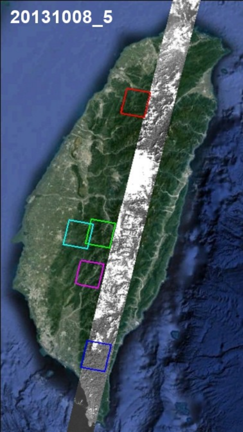
20131008_5

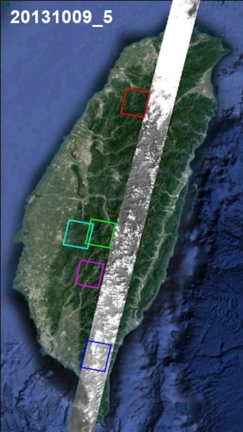
20131009_5

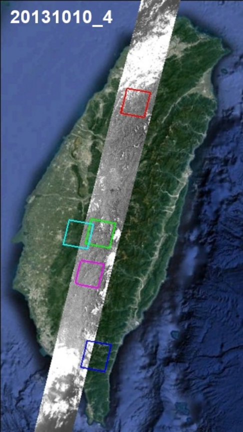
20131010_4

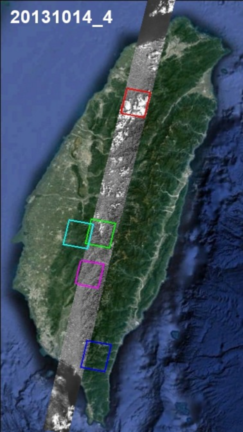
20131014_4

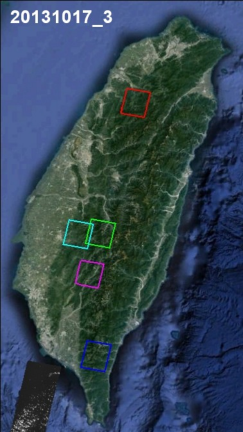
20131017_3

20131017_5

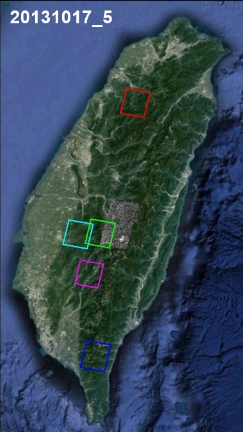

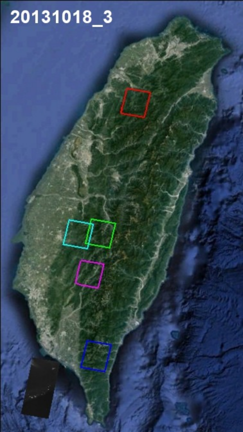
20131018_3

20131019_5

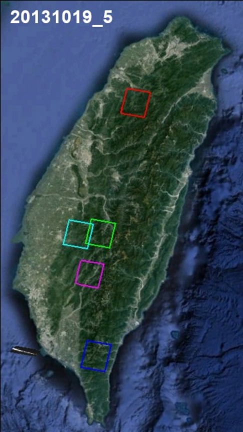

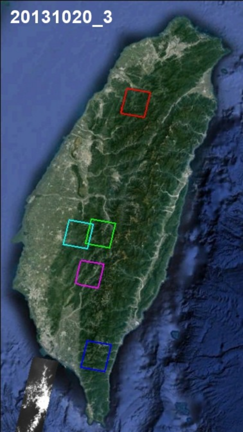
20131020_3

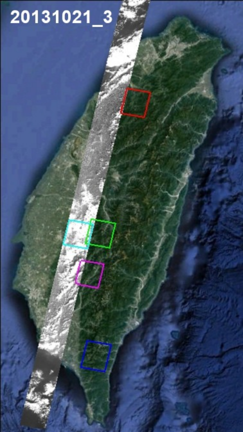
20131021_3

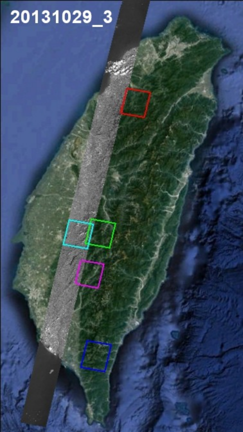
20131029_3

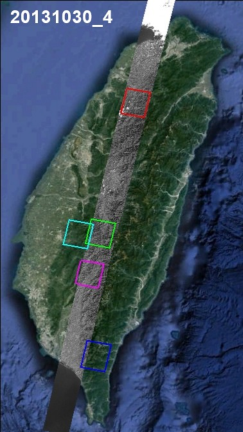
20131030_4

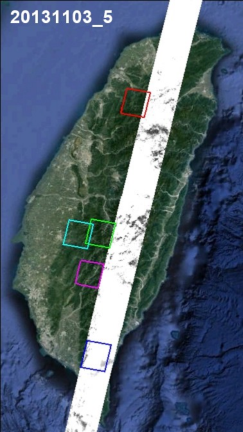
20131103_5

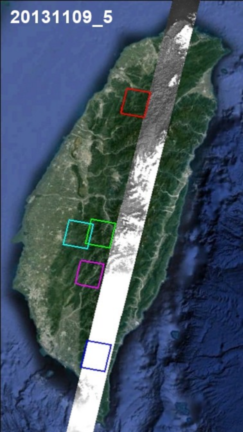
20131109_5

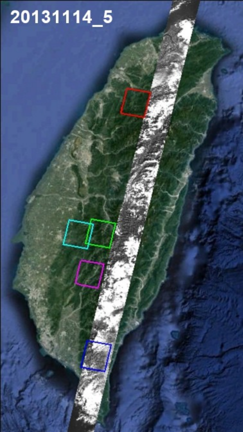
20131114_5

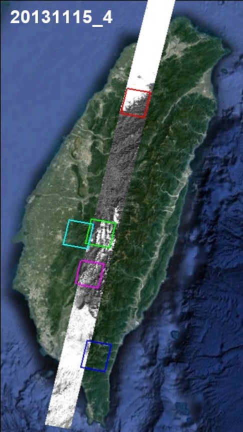
20131115_4

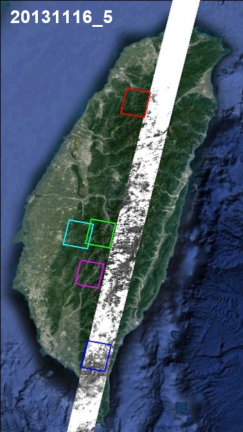
20131116_5

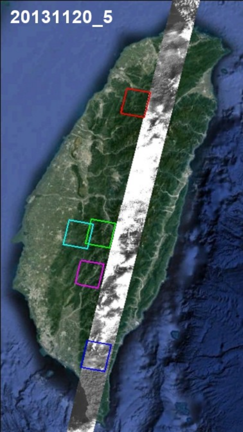
20131120_5

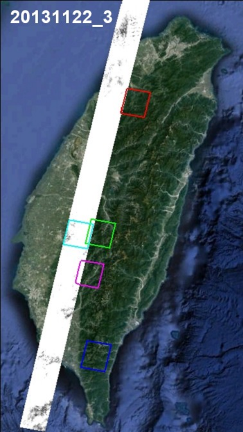
20131122_3

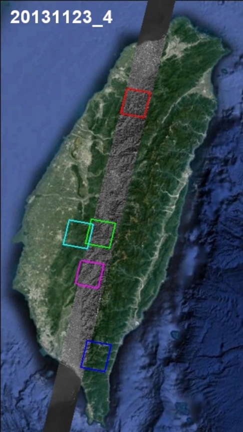
20131123_4

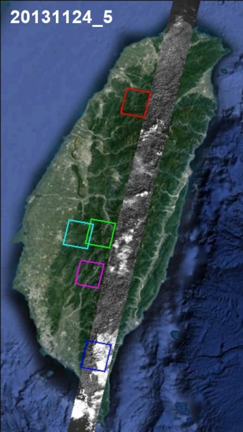
20131124_5

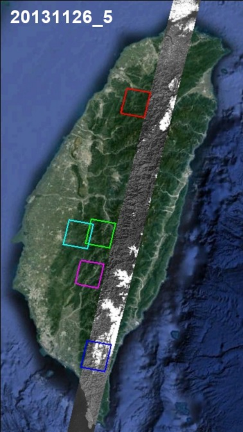

20131126_5

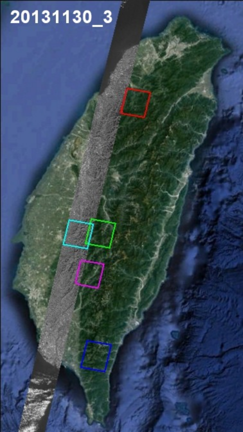
20131130_3

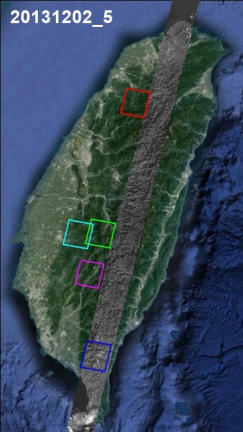
20131202_5

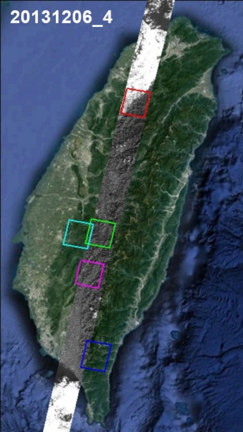
20131206_4

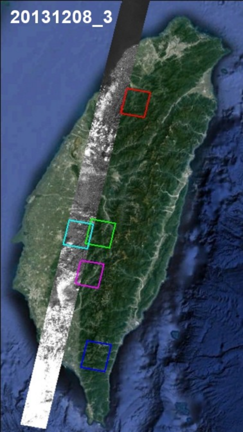
20131208_3

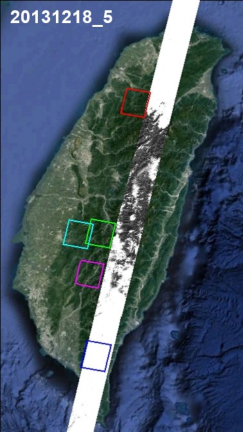
20131218_5

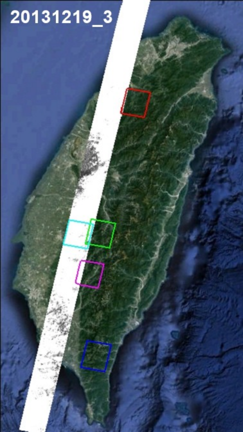
20131219_3

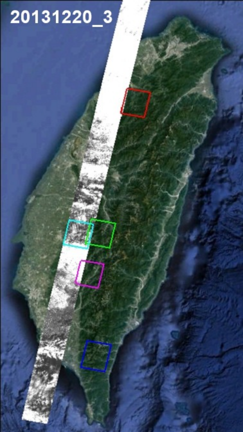
20131220_3

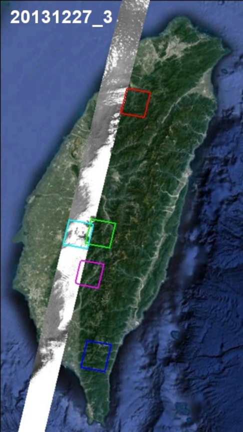
20131227_3

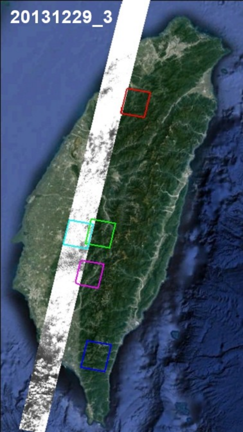
20131229_3

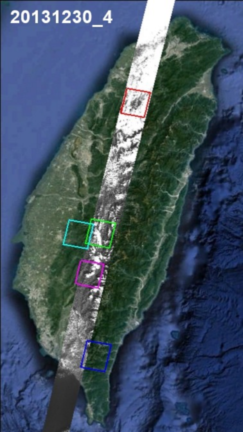
20131230_4

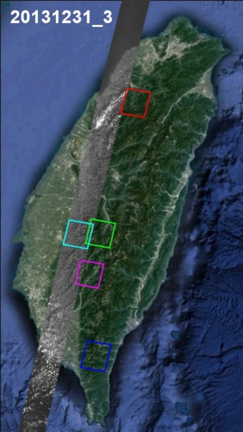
20131231_3

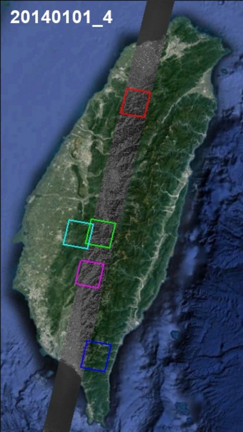
20140101_4

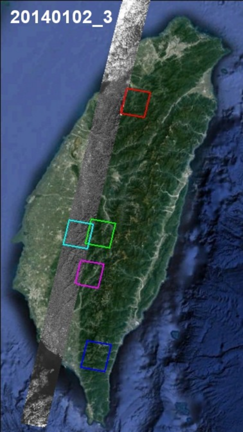
20140102_3

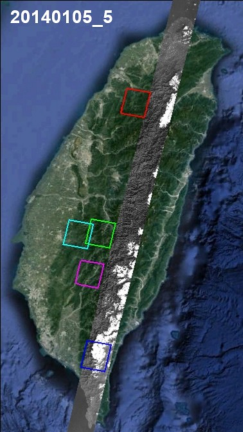
20140105_5

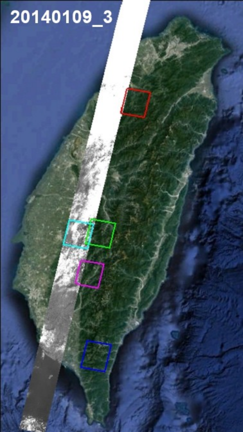
20140109_3

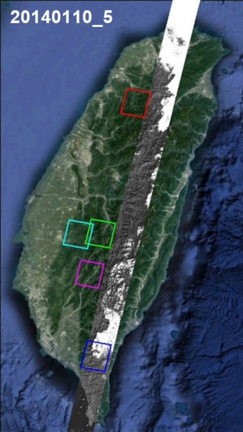
20140110_5

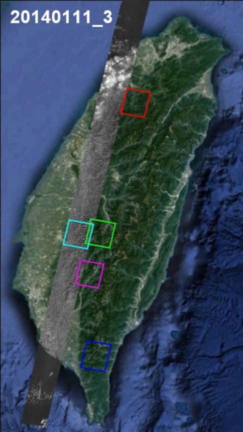
20140111_3

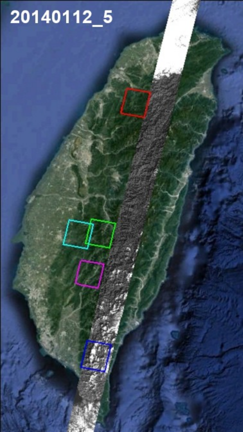
20140112_5

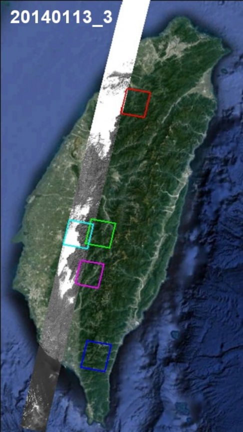
20140113_3

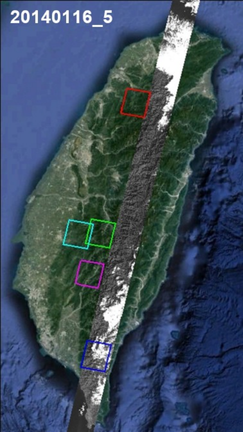
20140116_5

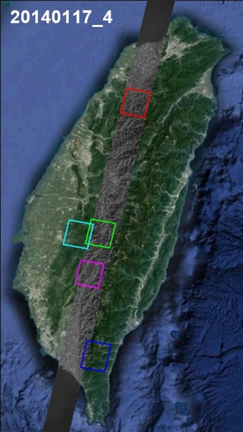
20140117_4

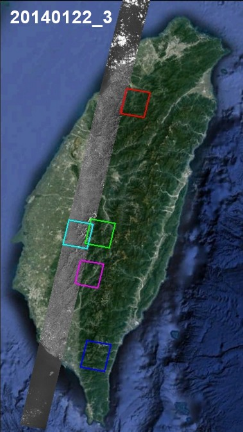
20140122_3

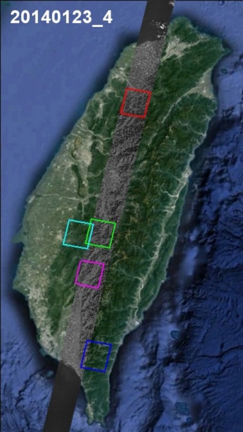
20140123_4

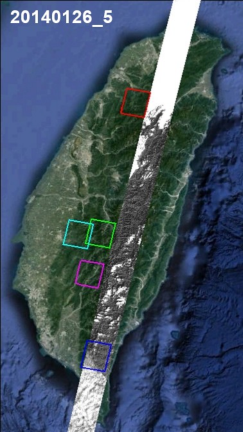
20140126_5

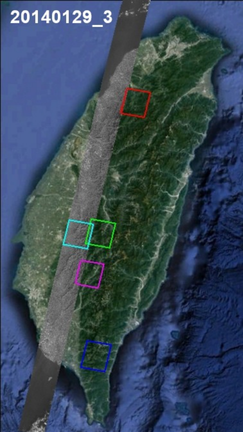
20140129_3

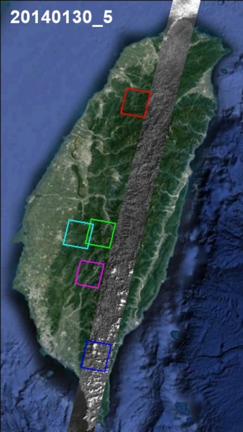
20140130_5

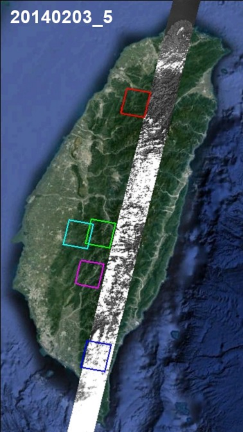
20140203_5

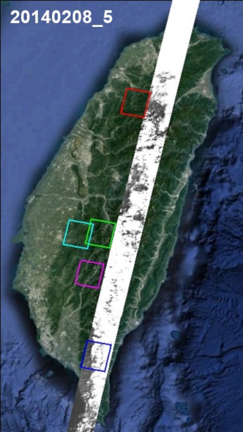
20140208_5

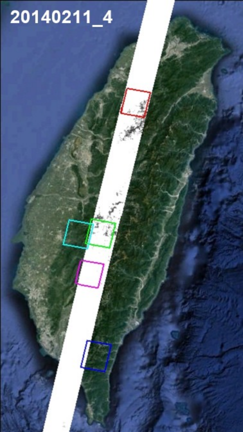
20140211_4

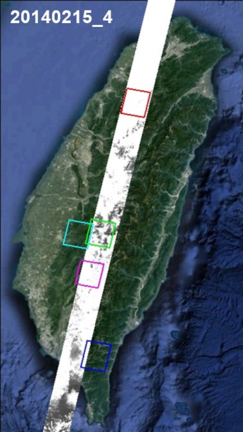
20140215_4

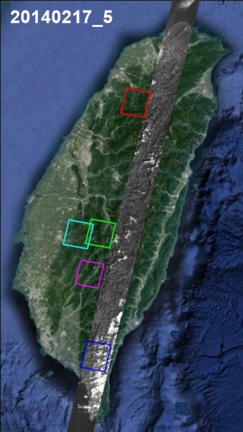
20140217_5

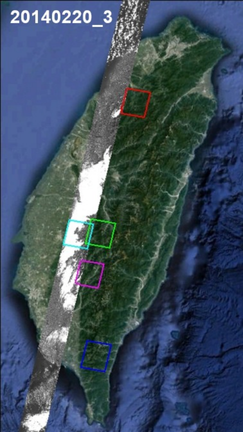
20140220_3

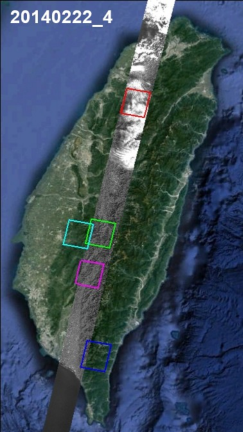
20140222_4

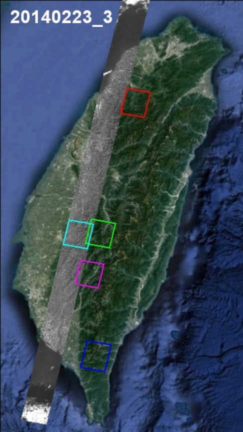
20140223_3

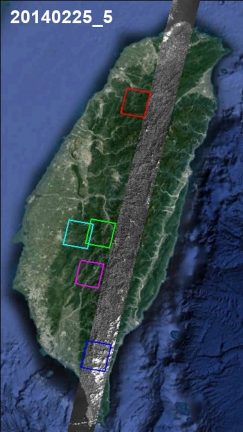
20140225_5

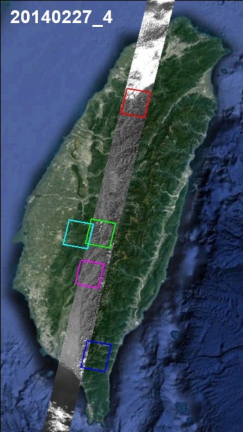
20140227_4

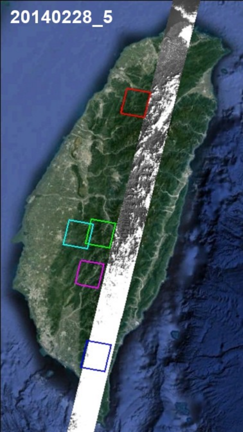
20140228_5

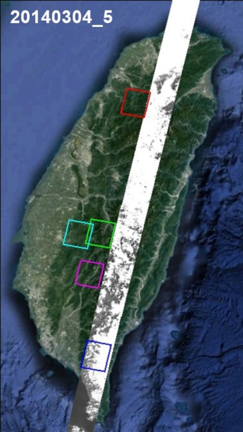
20140304_5

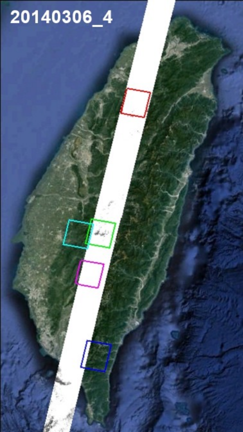
20140306_4

20140307_4

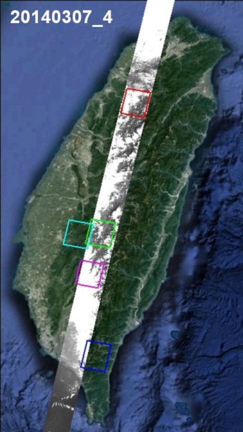

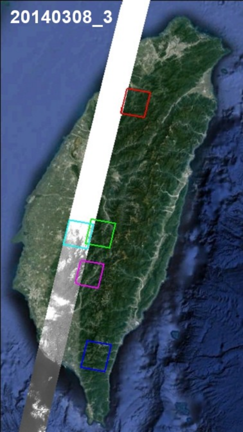
20140308_3

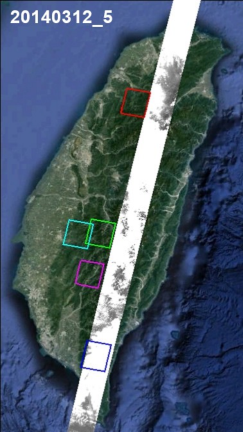
20140312_5

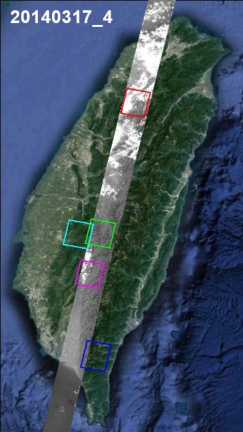
20140317_4

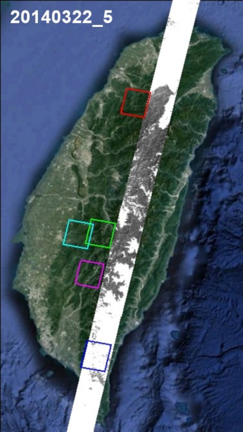
20140322_5

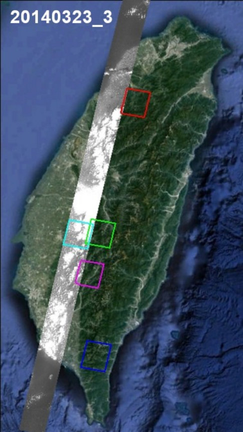
20140323_3

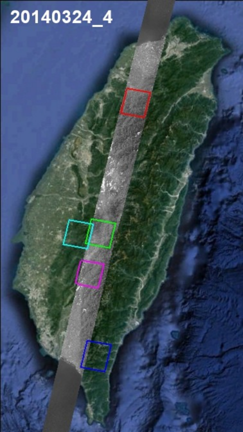
20140324_4

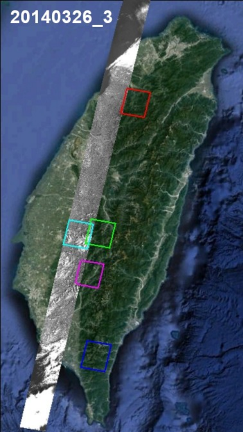
20140326_3

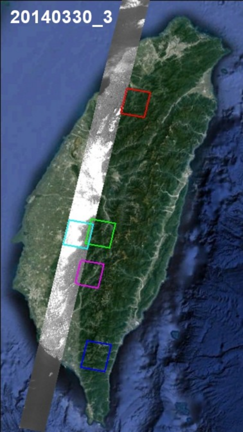
20140330_3

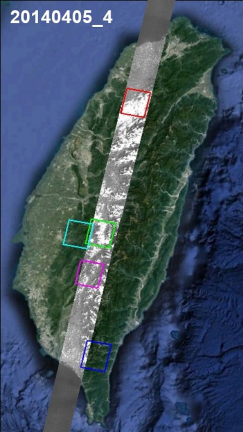
20140405_4

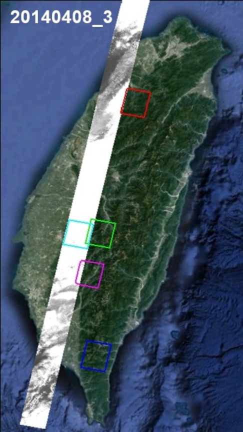
20140408_3

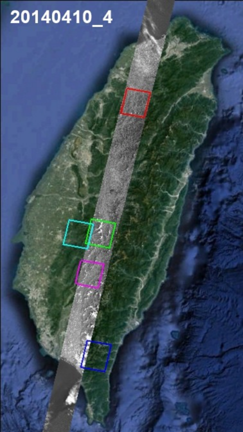
20140410_4

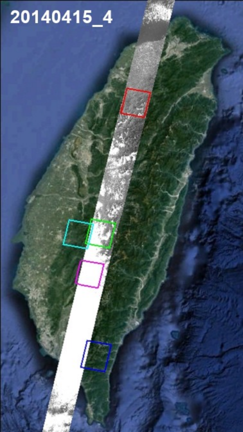
20140415_4

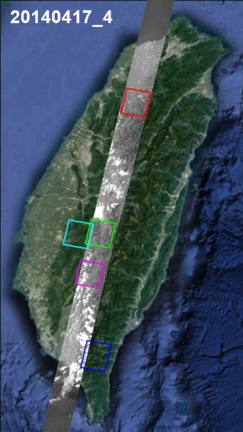
20140417_4

20140418_5

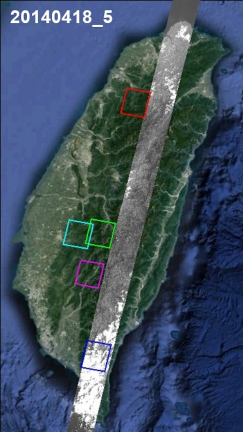

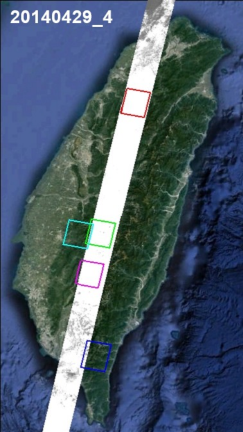
20140429_4

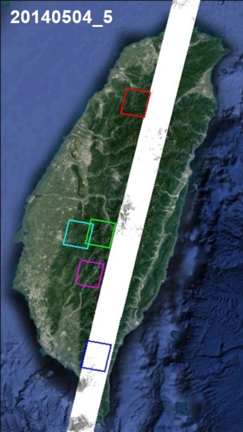
20140504_5

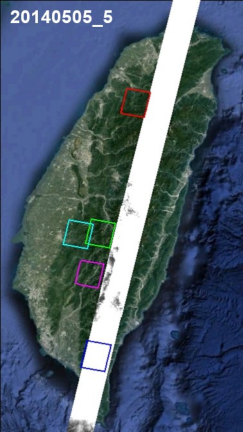
20140505_5

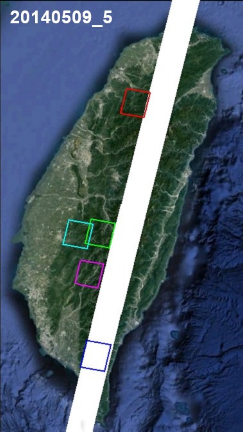
20140509_5

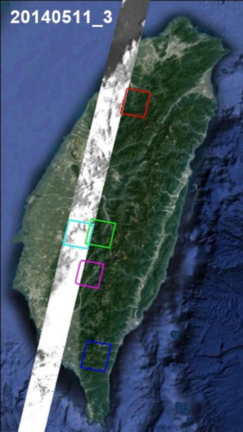
20140511_3

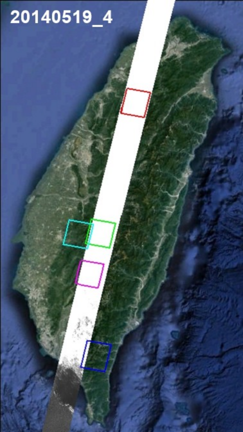
20140519_4

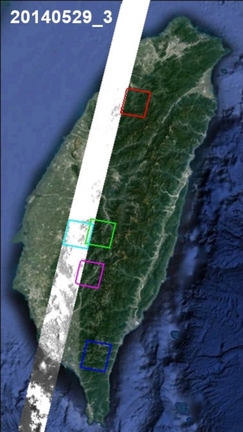
20140529_3

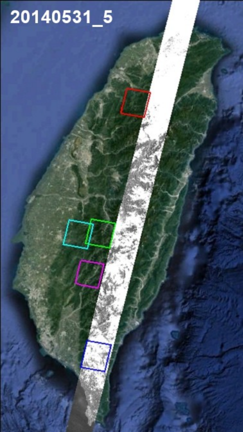
20140531_5

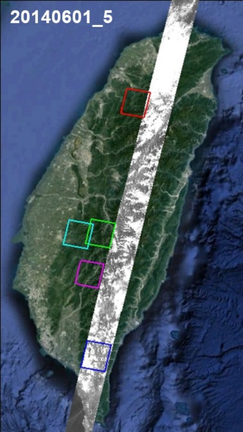
20140601_5

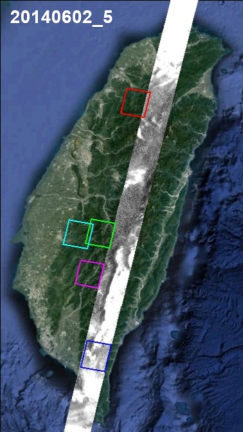
20140602_5

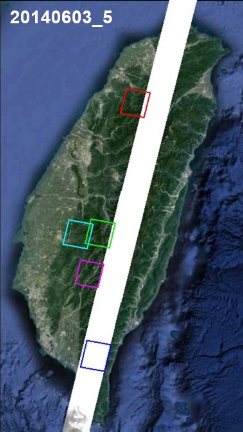
20140603_5

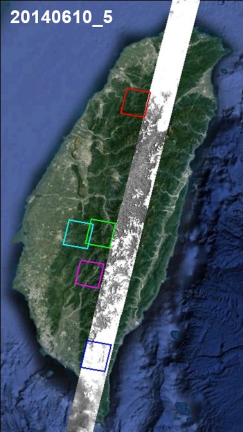
20140610_5

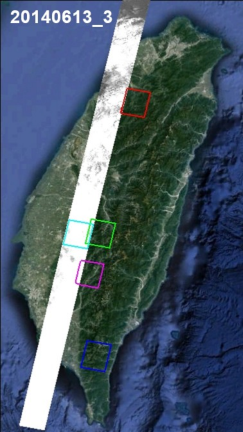
20140613_3

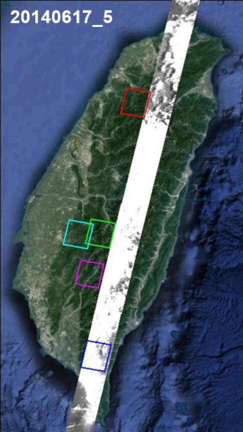
20140617_5

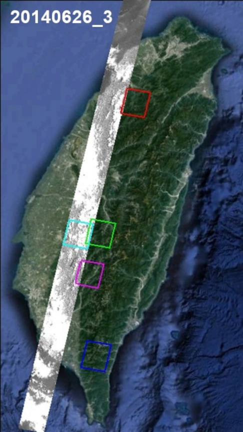
20140626_3

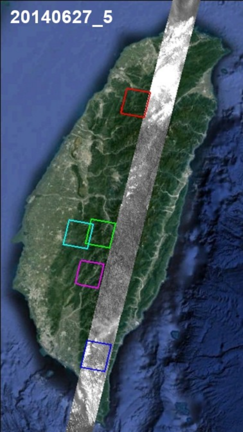
20140627_5

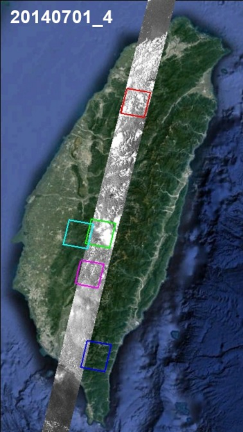
20140701_4

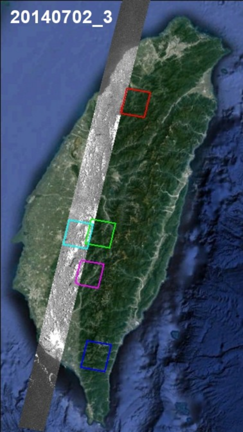
20140702_3

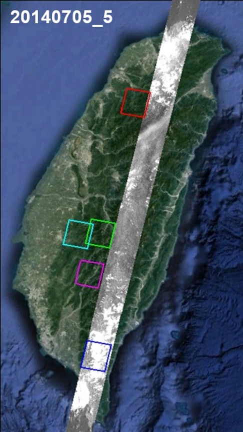
20140705_5

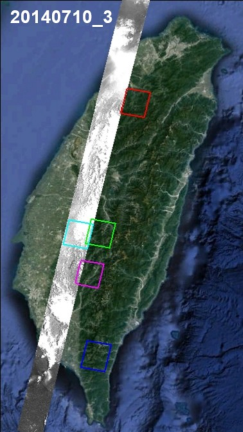
20140710_3

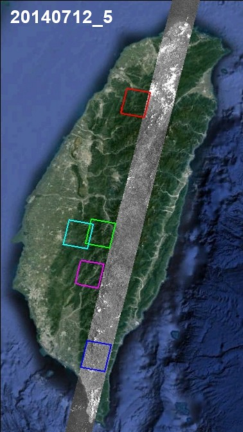
20140712_5

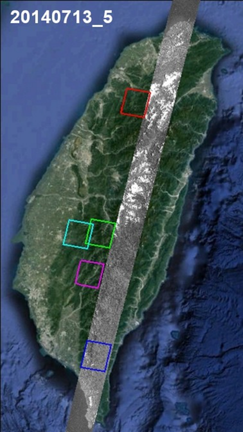
20140713_5

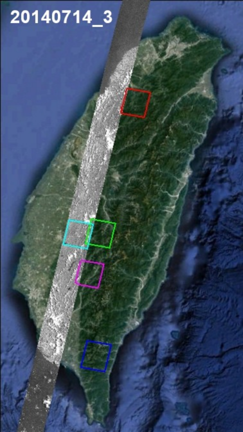
20140714_3

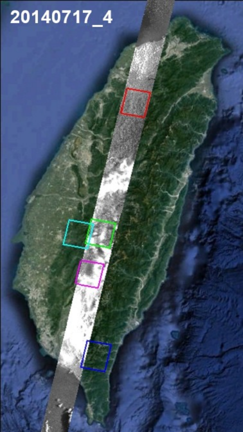
20140717_4

20140723_5

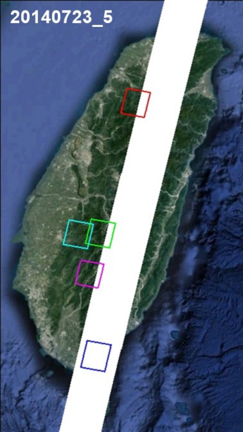

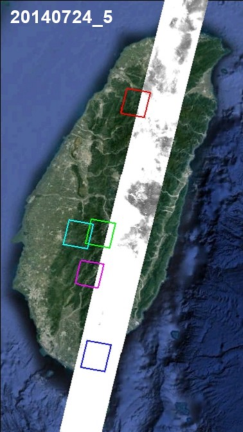
20140724_5

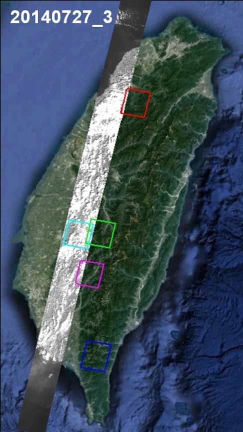
20140727_3

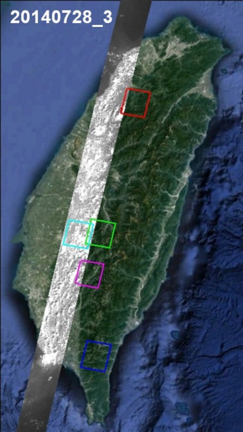
20140728_3

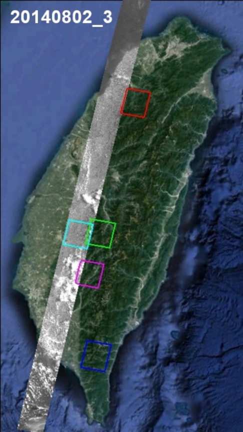
20140802_3

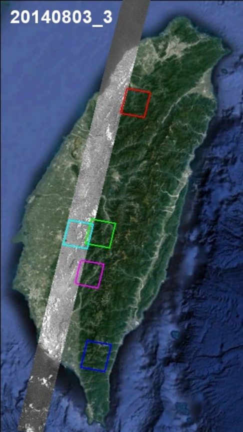
20140803_3

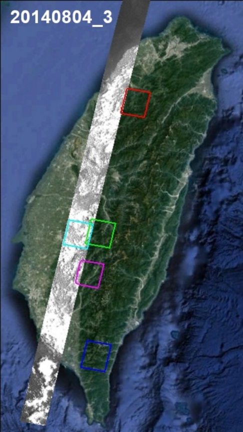
20140804_3

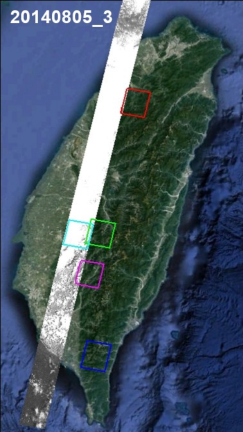
20140805_3

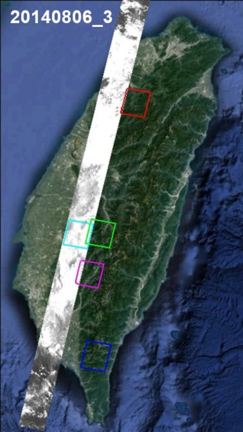
20140806_3

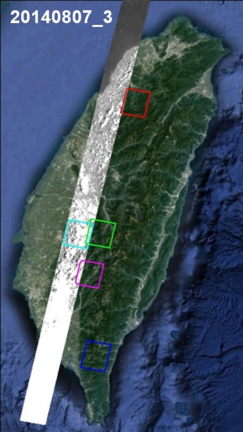
20140807_3

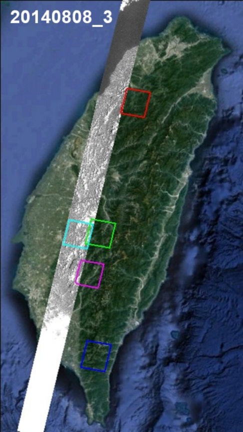
20140808_3

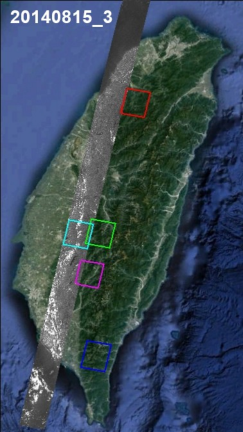
20140815_3

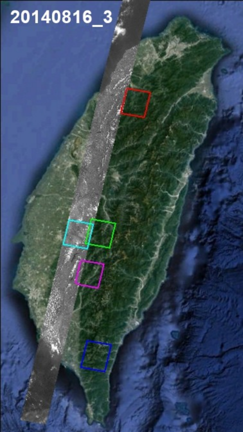
20140816_3

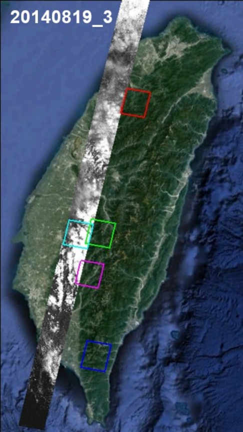
20140819_3

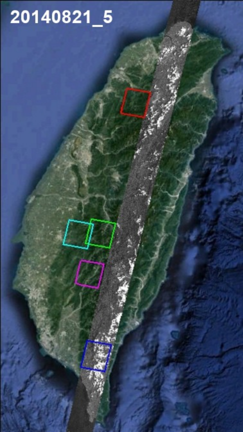
20140821_5

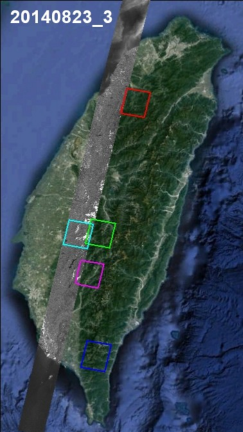
20140823_3

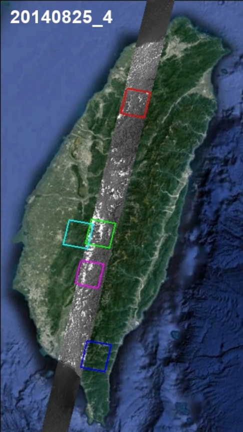
20140825_4

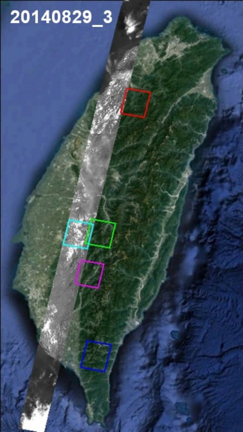
20140829_3

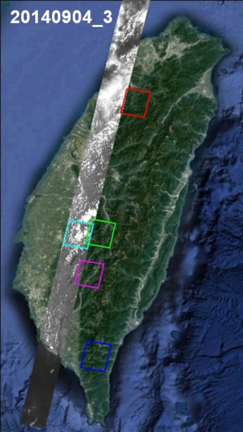
20140904_3

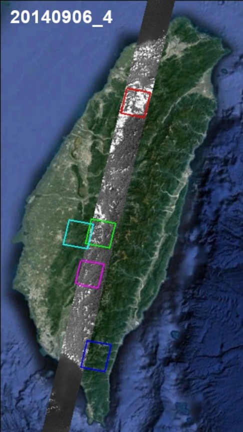
20140906_4

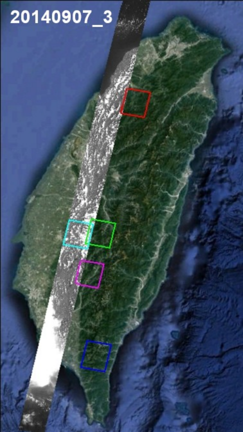
20140907_3

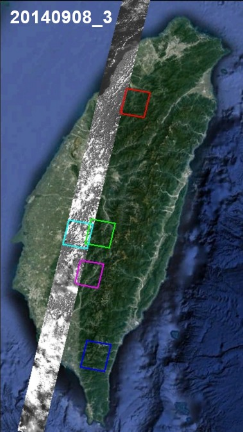
20140908_3

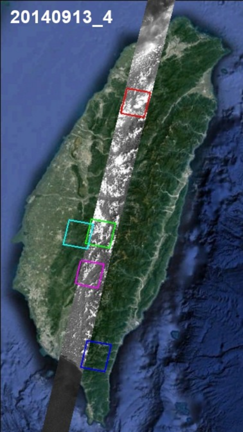

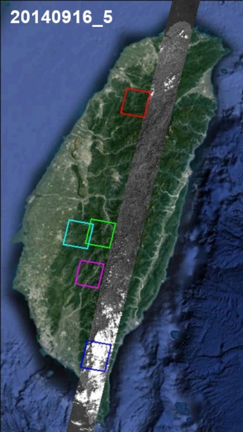
20140916_5

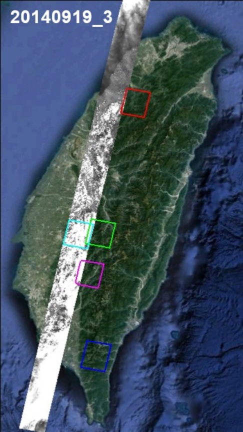
20140919_3

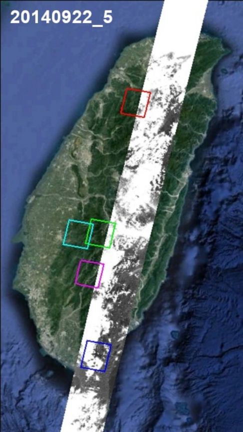
20140922_5

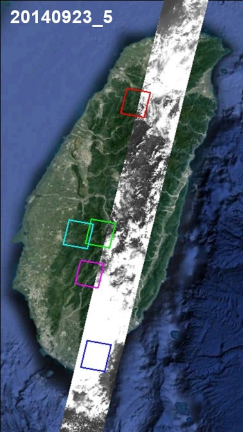
20140923_5

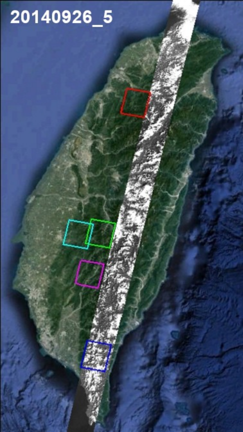
20140926_5

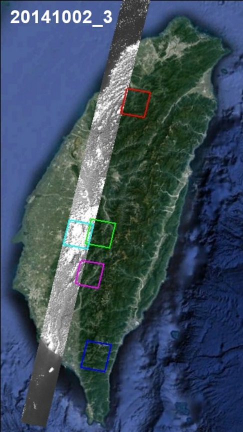
20141002_3

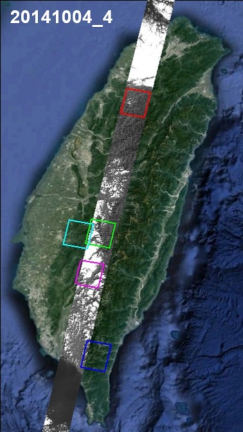
20141004_4

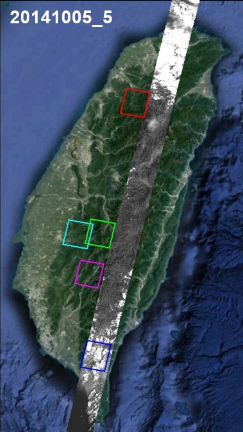
20141005_5

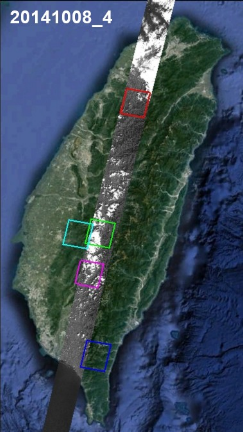
20141008_4

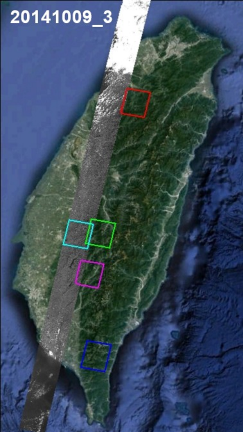
20141009_3

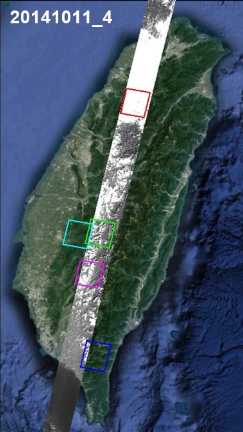
20141011_4

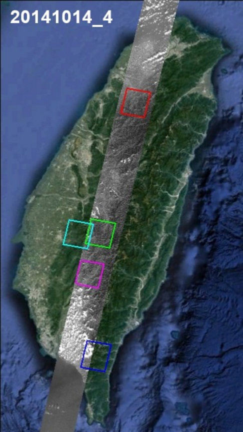
20141014_4

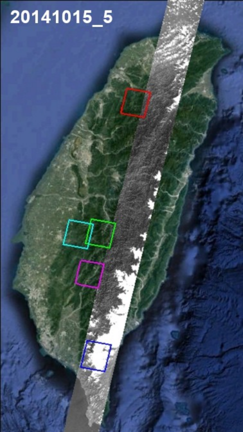
20141015_5

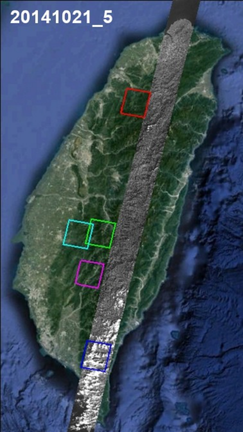
20141021_5

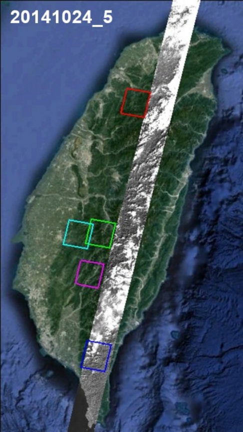
20141024_5

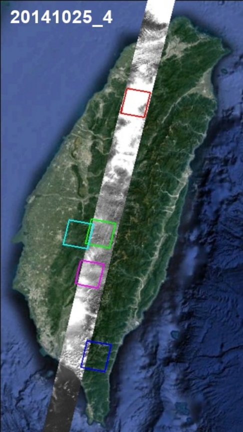
20141025_4

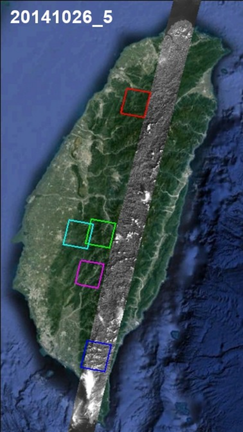

20141026_5

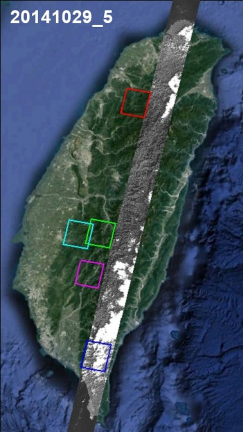
20141029_5

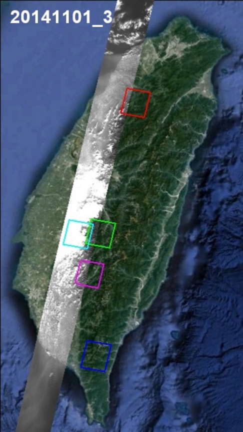
20141101_3

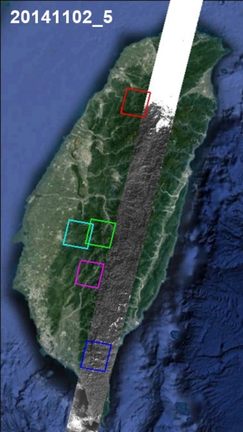
20141102_5

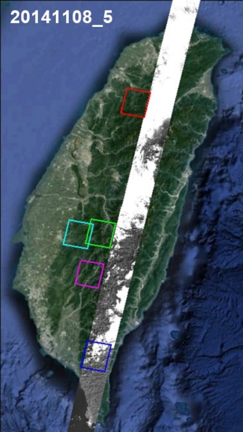
20141108_5

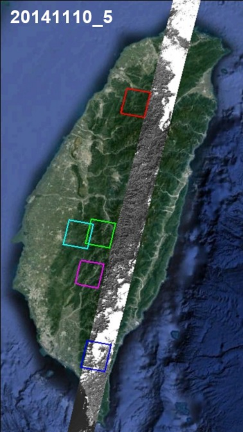
20141110_5

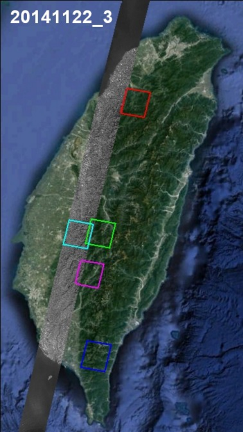
20141122_3

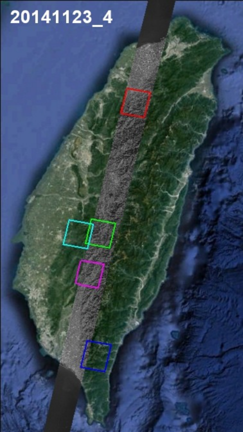
20141123_4

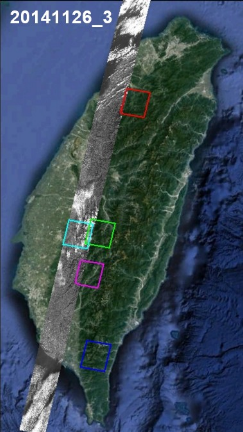
20141126_3

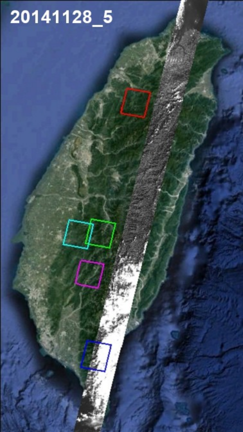
20141128_5

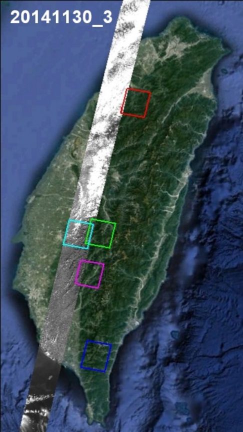
20141130_3

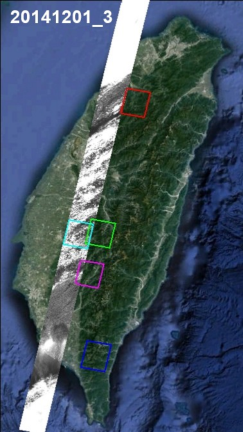
20141201_3

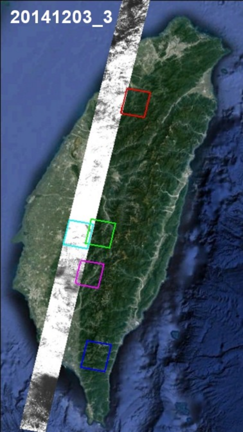
20141203_3

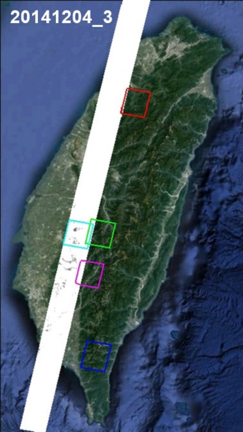
20141204_3

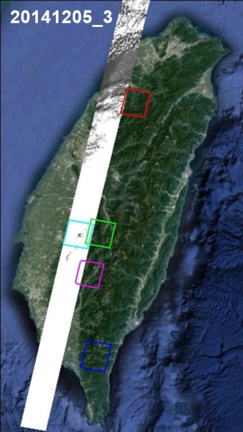
20141205_3

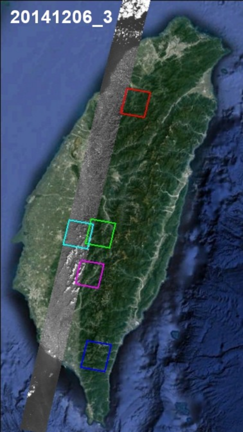

20141206_3

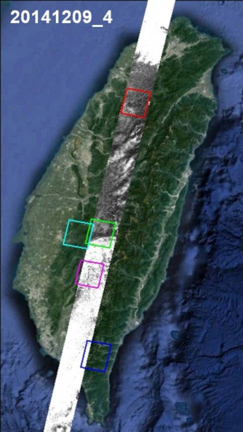
20141209_4

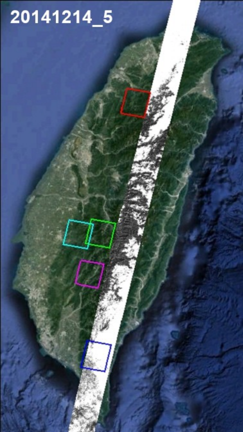
20141214_5

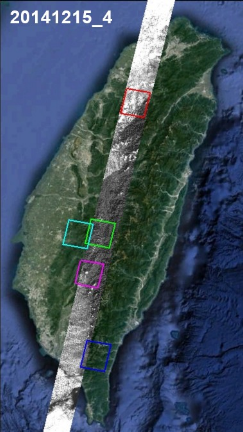
20141215_4

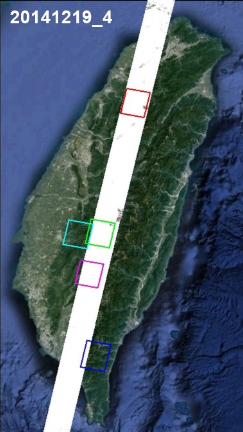
20141219_4

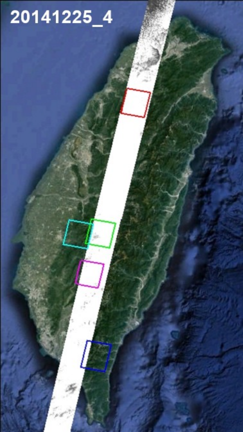
20141225_4

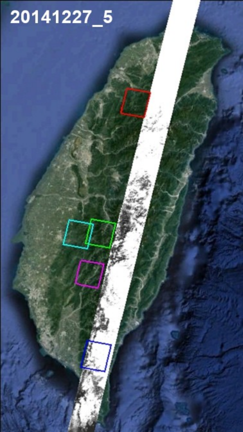
20141227_5

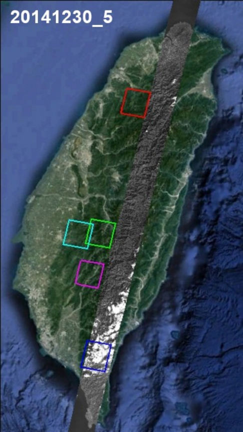
20141230_5

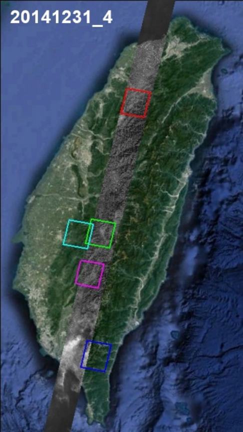
20141231_4

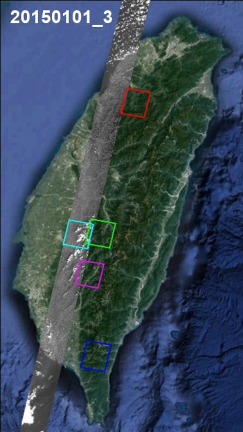
20150101_3

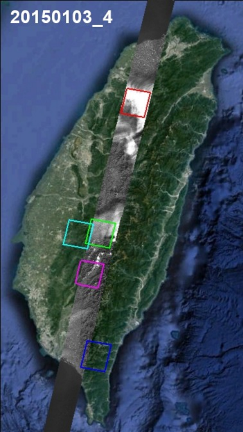
20150103_4

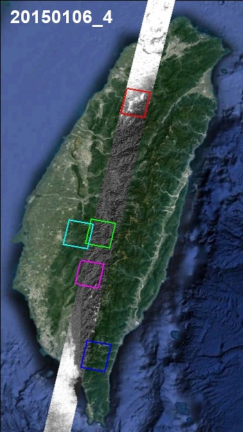
20150106_4

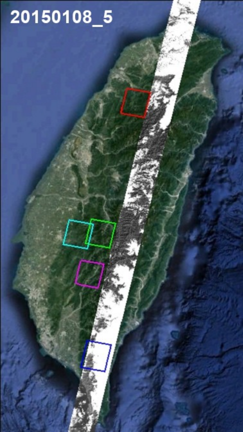
20150108_5

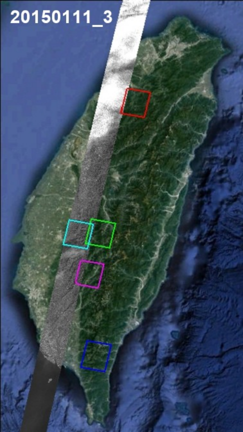
20150111_3

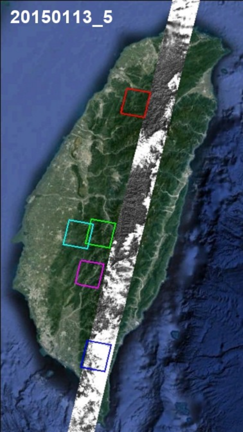
20150113_5

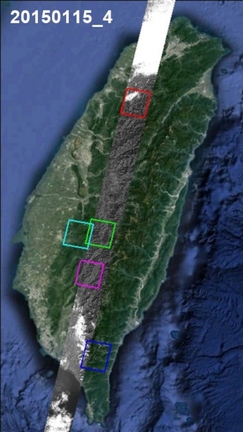
20150115_4

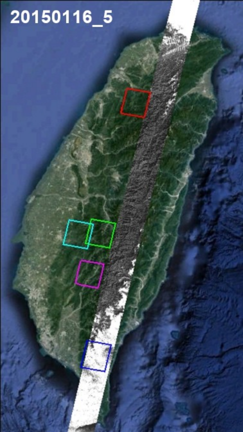
20150116_5

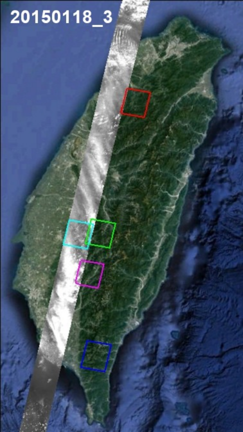
20150118_3

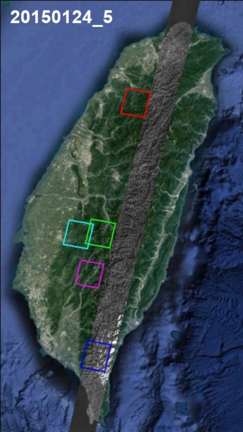
20150124_5

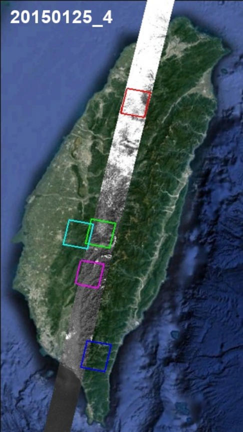
20150125_4

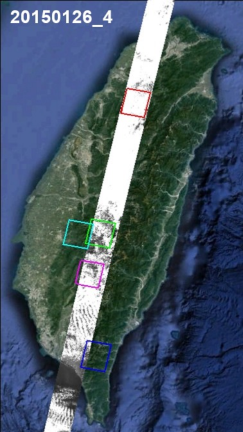
20150126_4

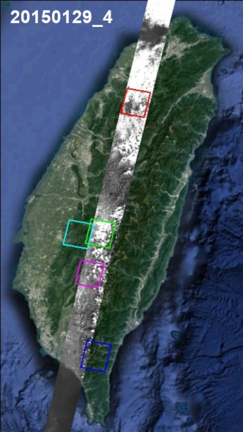
20150129_4

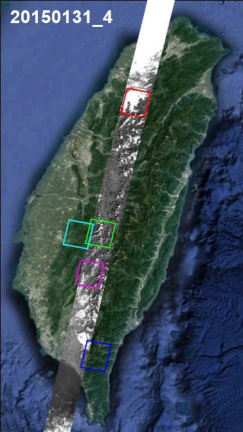
20150131_4

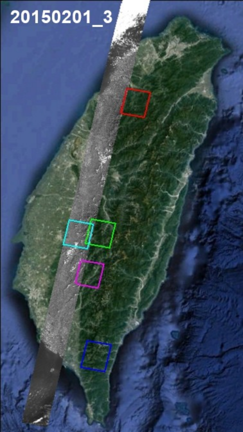
20150201_3

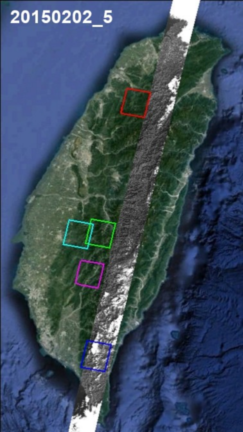
20150202_5

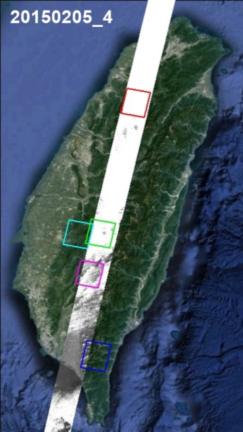
20150205_4

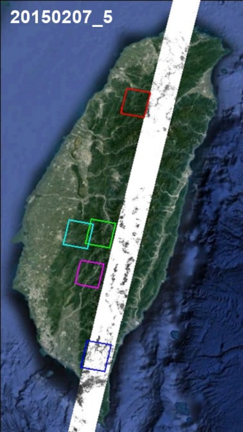
20150207_5

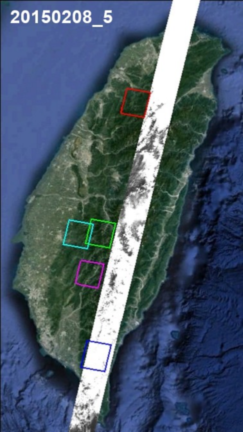
20150208_5

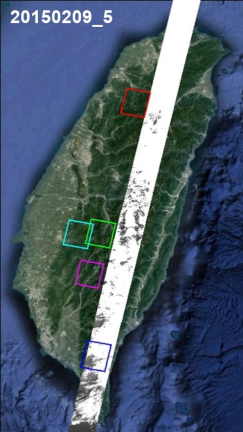
20150209_5

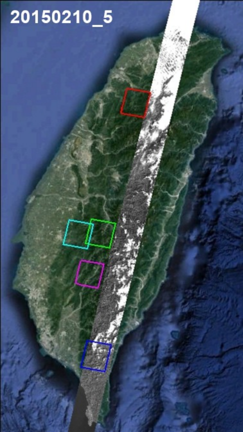

20150210_5

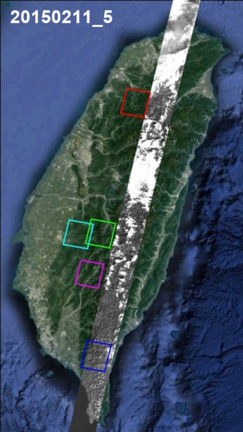
20150211_5

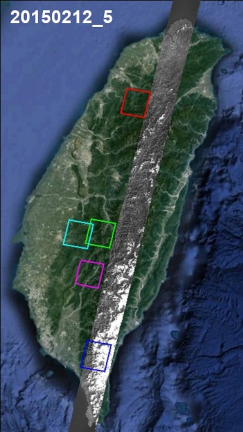
20150212_5

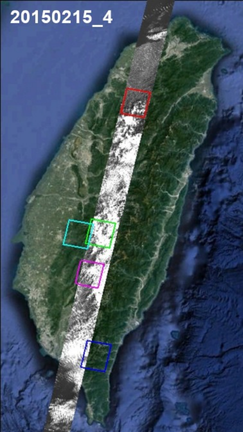
20150215_4

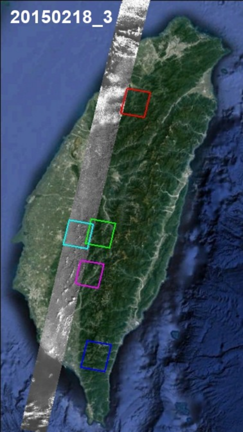
20150218_3

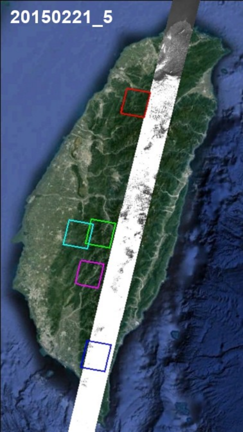
20150221_5

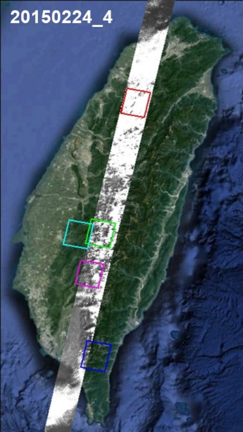
20150224_4

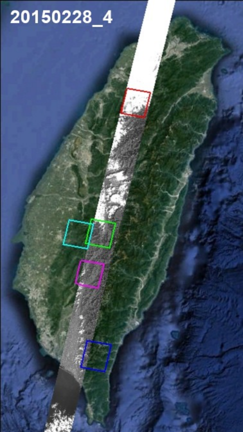
20150228_4

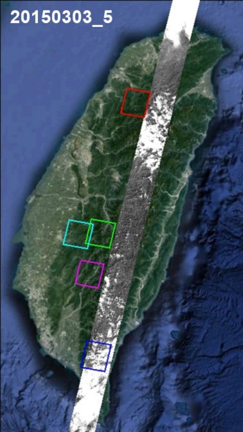
20150303_5

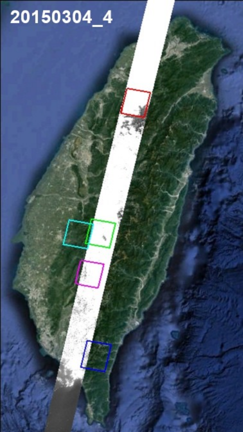
20150304_4

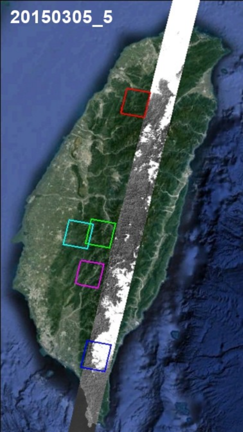
20150305_5

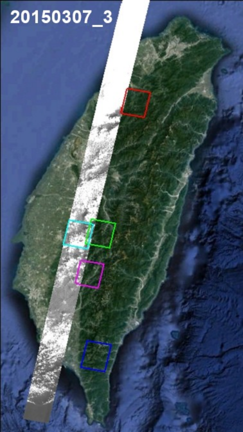
20150307_3

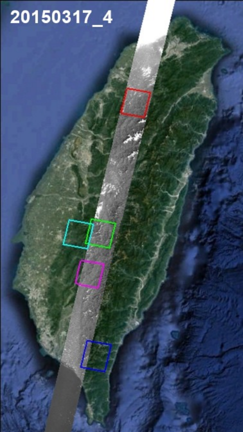
20150317_4

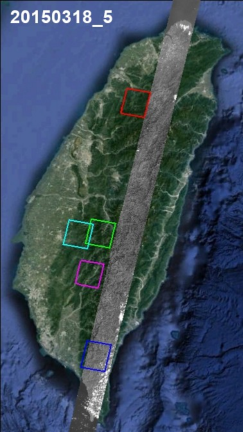
20150318_5

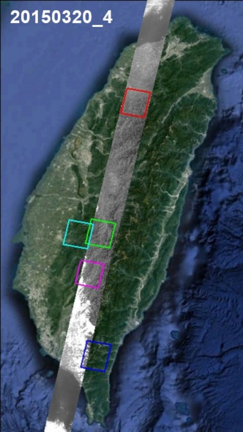
20150320_4

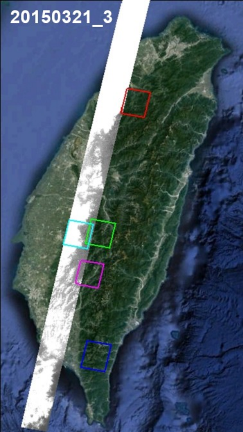
20150321_3

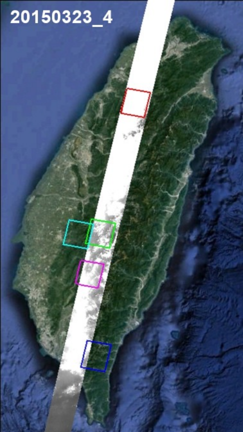
20150323_4

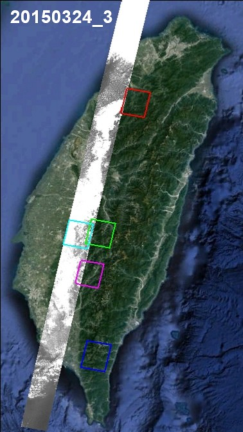
20150324_3

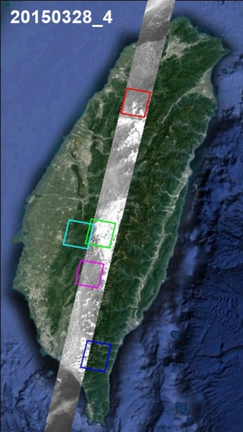
20150328_4

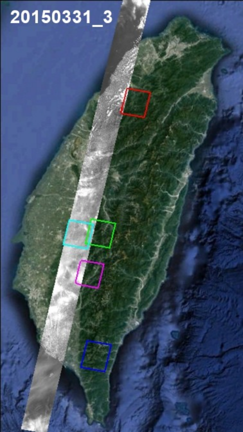
20150331_3

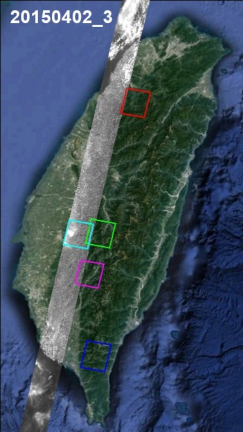
20150402_3

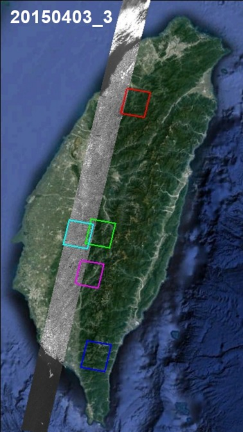
20150403_3

20150406_4

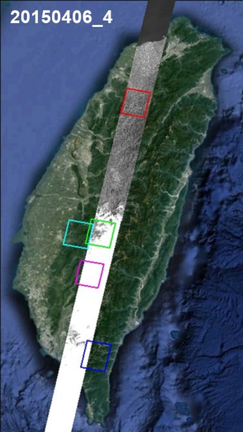

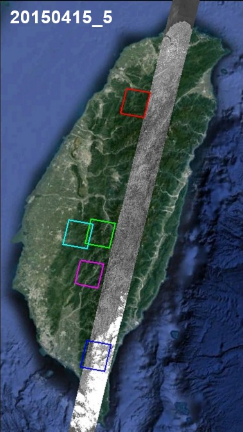
20150415_5

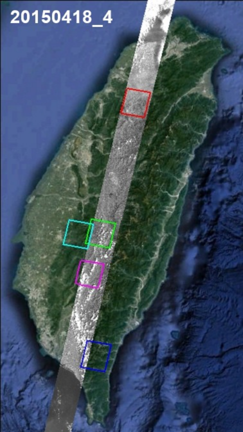
20150418_4

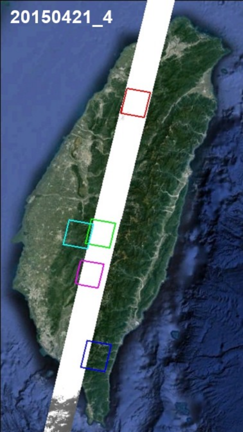
20150421_4

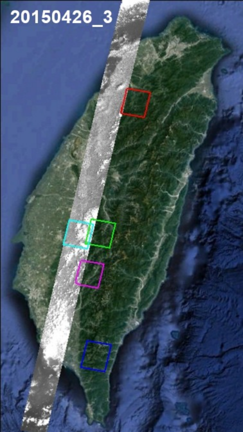
20150426_3

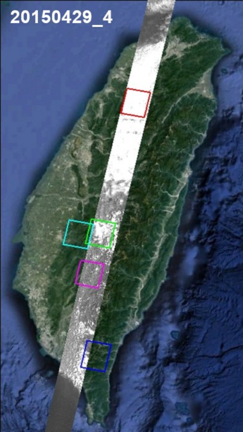
20150429_4

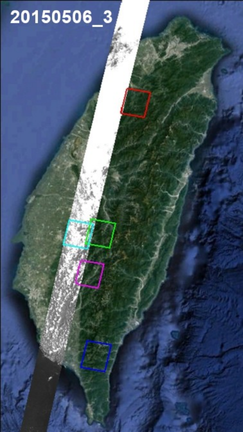
20150506_3

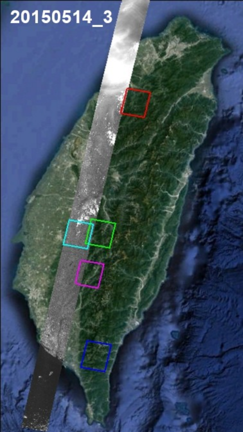
20150514_3

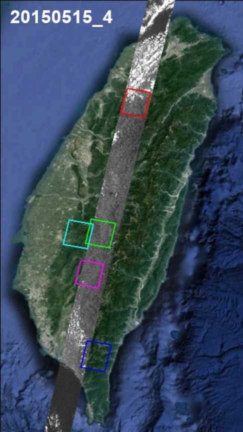

20150515_4

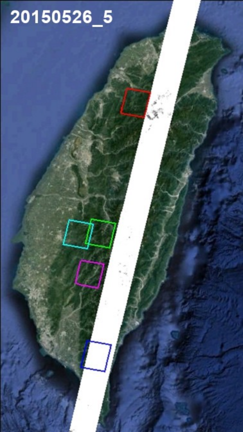
20150526_5

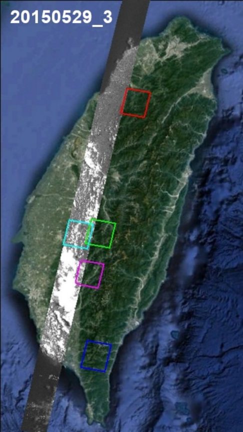
20150529_3

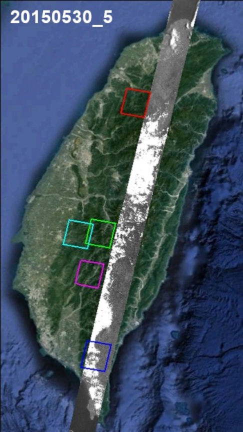
20150530_5

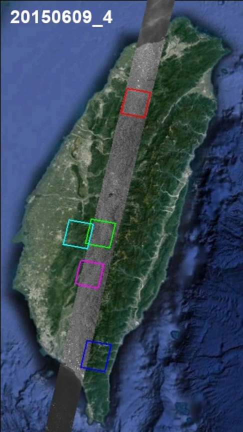
20150609_4

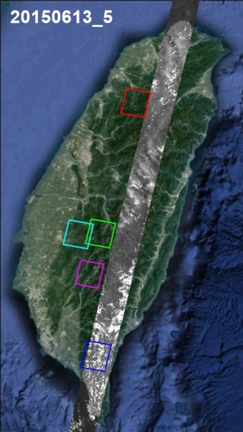
20150613_5

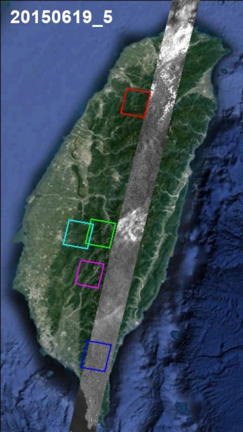
20150619_5

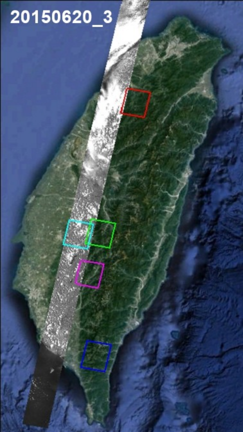
20150620_3

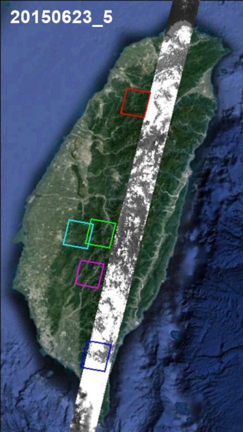
20150623_5

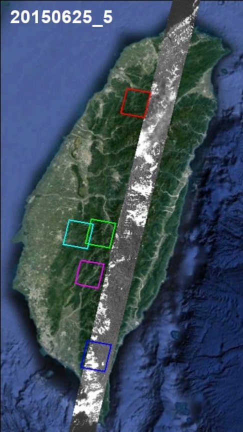

20150625_5

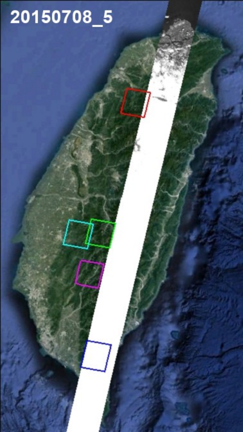
20150708_5

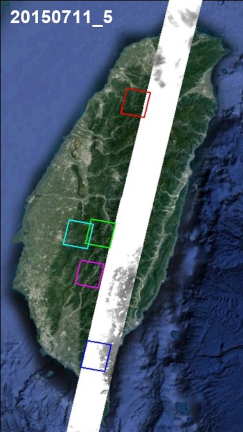
20150711_5

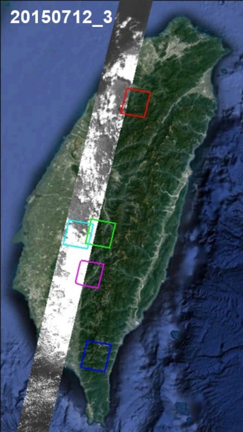
20150712_3

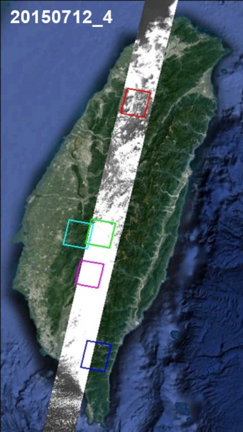
20150712_4

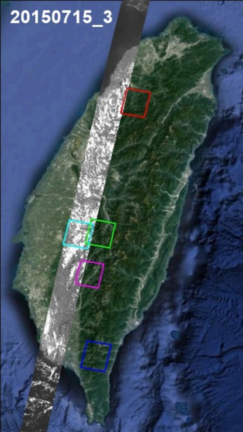
20150715_3

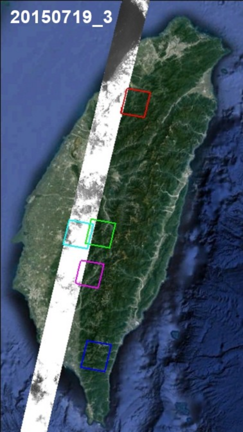
20150719_3

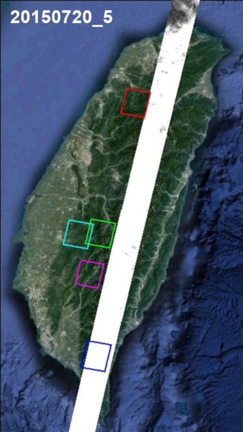
20150720_5

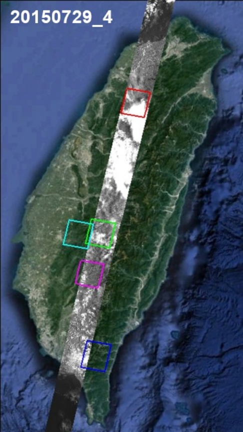
20150729_4

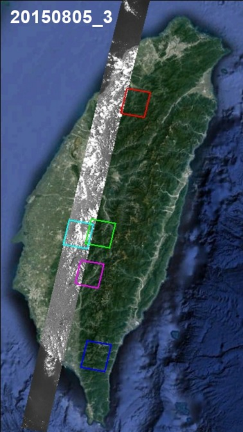
20150805_3

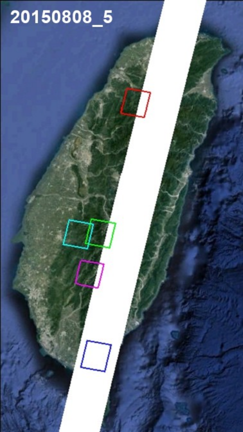
20150808_5

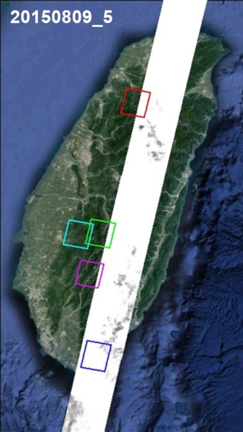
20150809_5

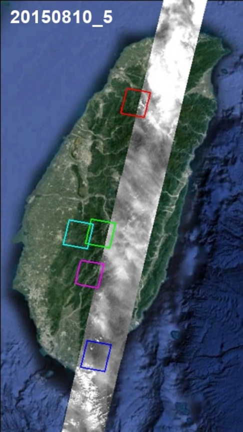
20150810_5

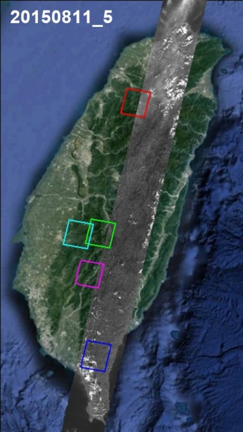
20150811_5

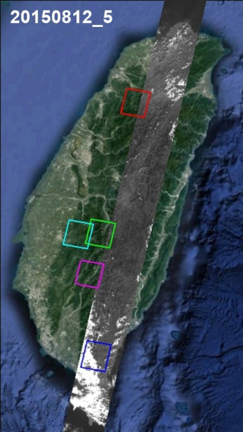
20150812_5

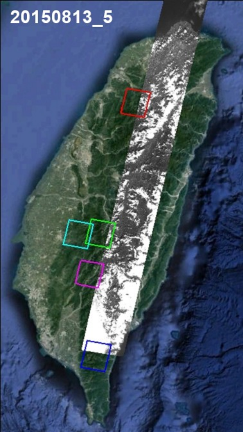
20150813_5

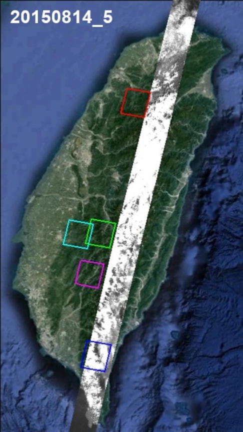
20150814_5

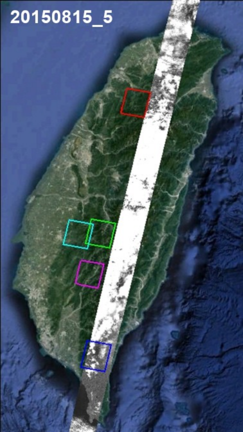
20150815_5

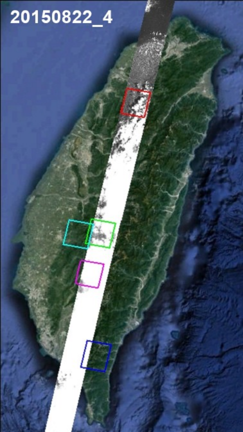
20150822_4

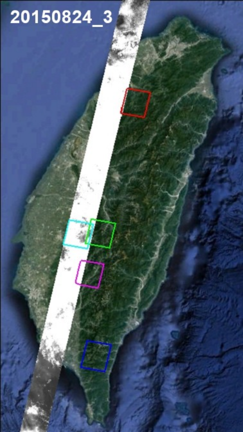
20150824_3

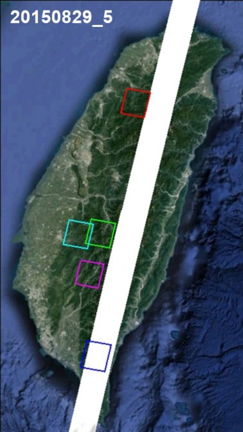
20150829_5

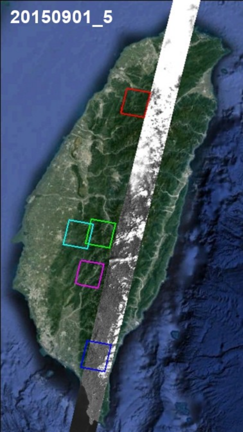
20150901_5

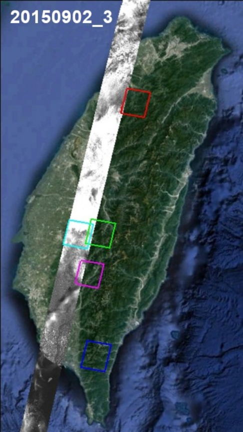
20150902_3

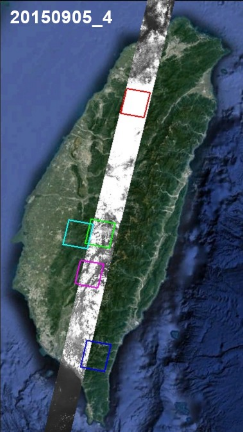
20150905_4

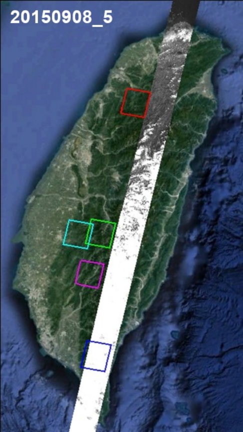
20150908_5

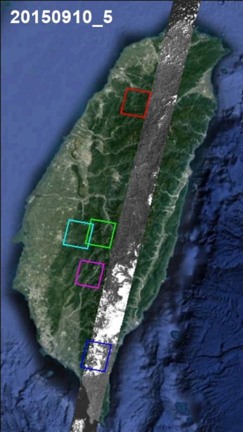
20150910_5

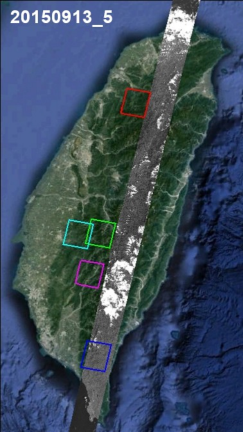
20150913_5

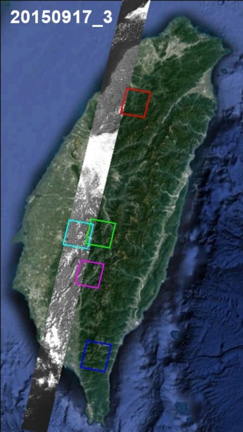
20150917_3

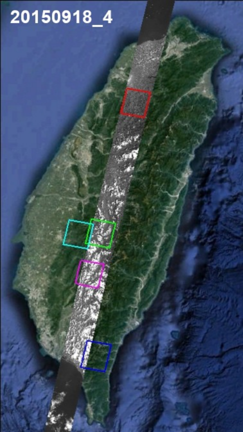
20150918_4

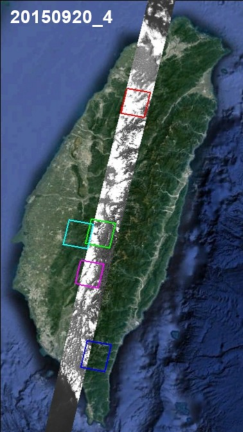
20150920_4

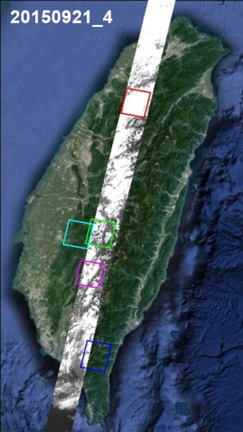
20150921_4

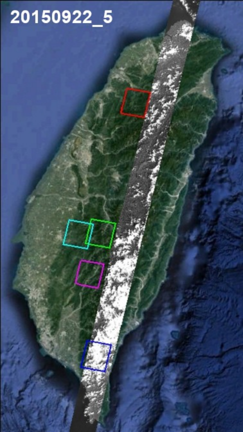
20150922_5

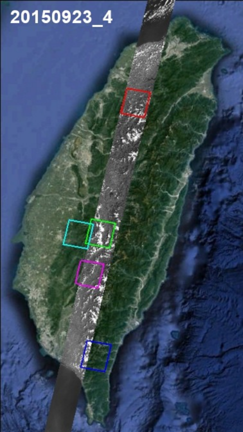
20150923_4

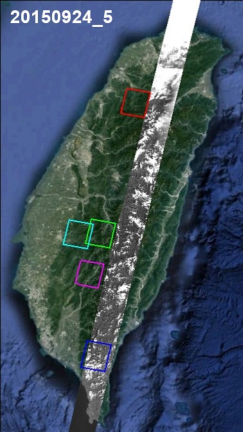
20150924_5

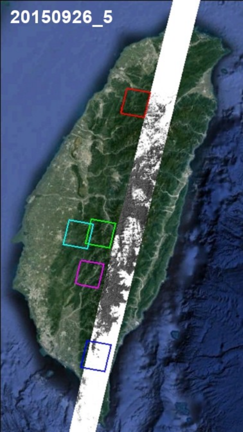
20150926_5

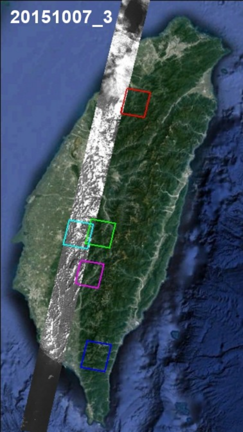
20151007_3

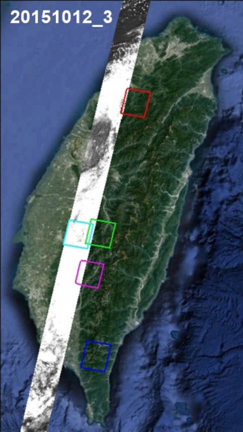
20151012_3

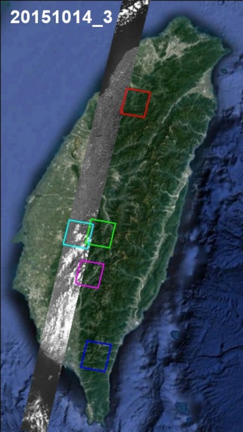
20151014_3

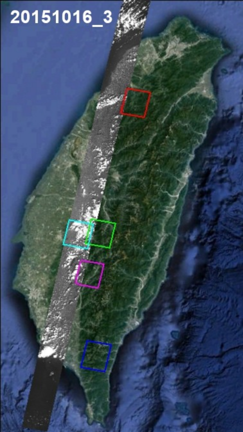
20151016_3

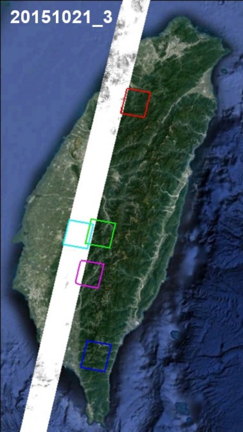
20151021_3

20151023_3

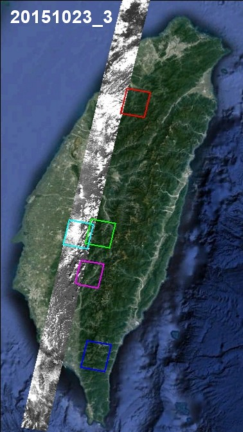

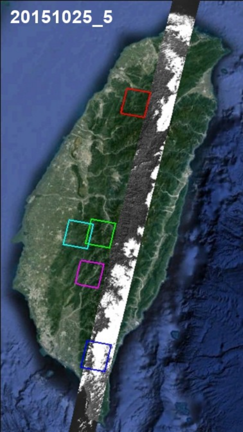
20151025_5

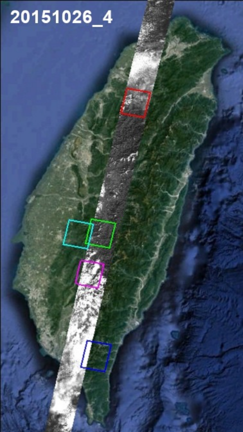
20151026_4

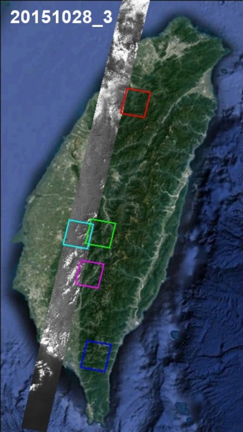
20151028_3

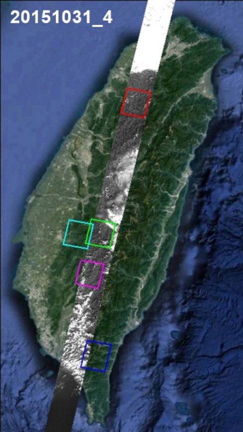
20151031_4

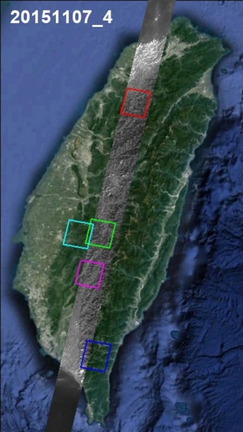
20151107_4

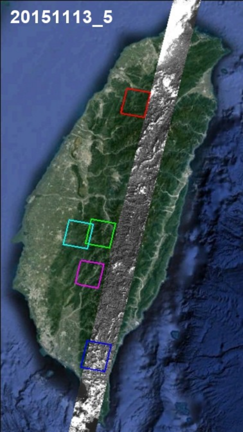
20151113_5

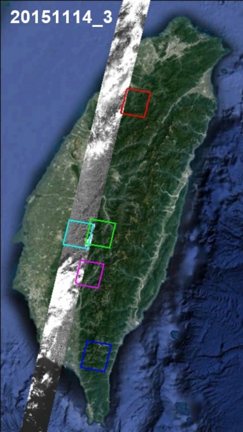
20151114_3

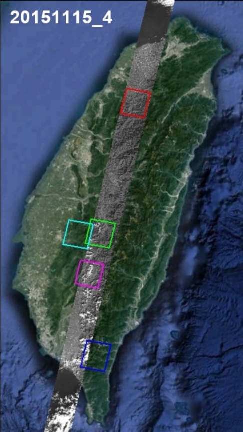
20151115_4

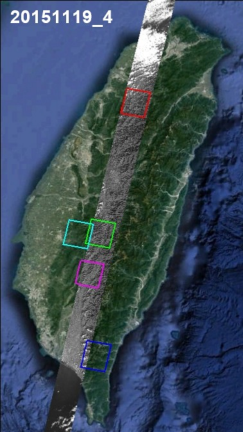
20151119_4

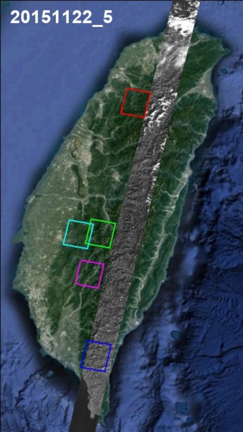
20151122_5

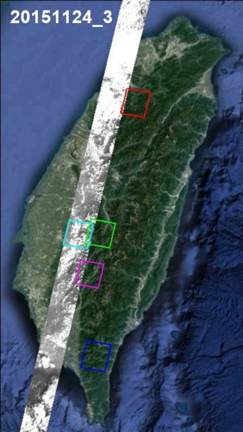
20151124_3

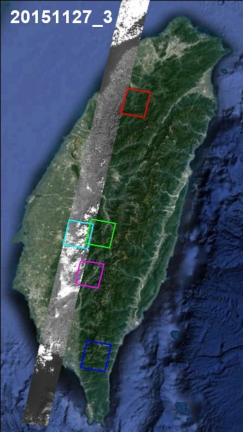
20151127_3

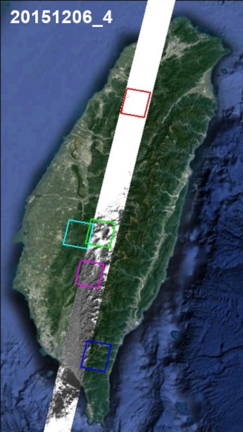
20151206_4

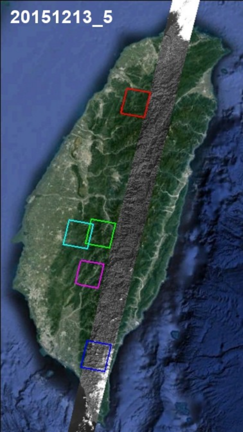
20151213_5

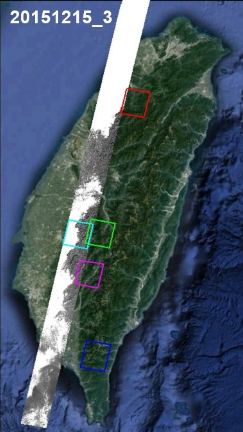
20151215_3

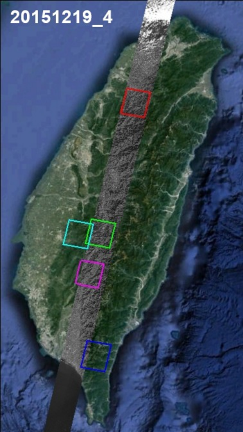
20151219_4

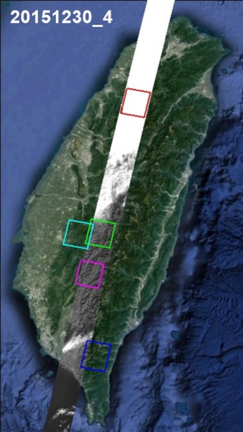
20151230_4

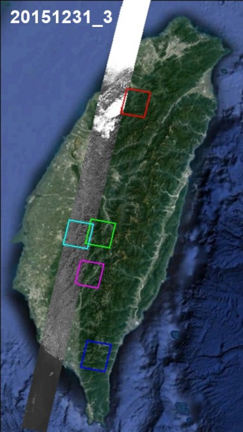
20151231_3

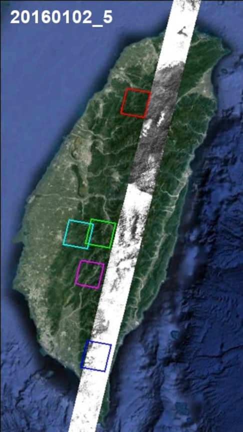
20160102_5

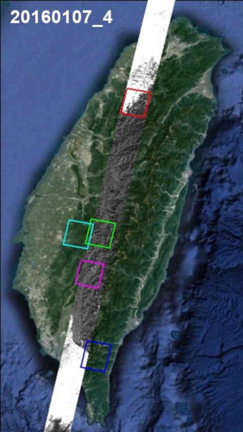
20160107_4

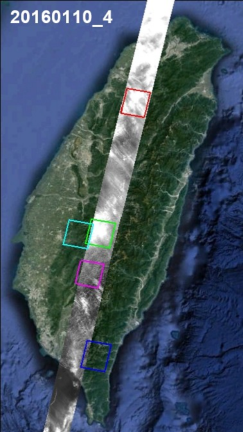
20160110_4

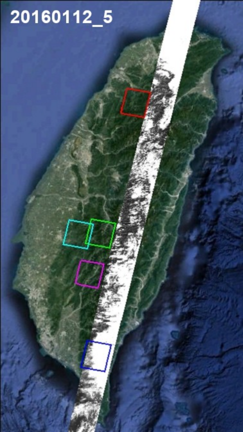
20160112_5

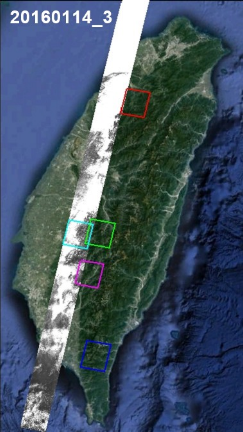
20160114_3

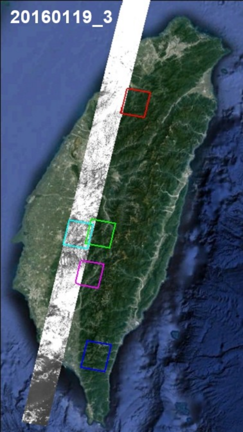
20160119_3

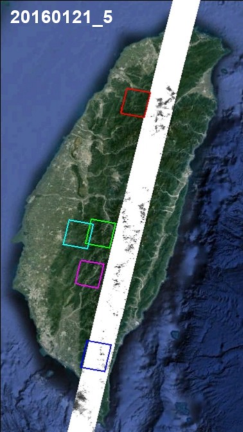
20160121_5

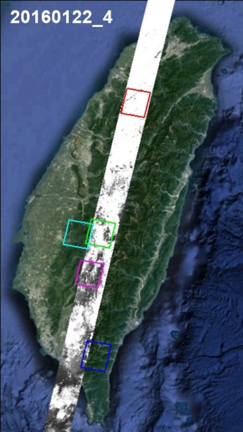
20160122_4

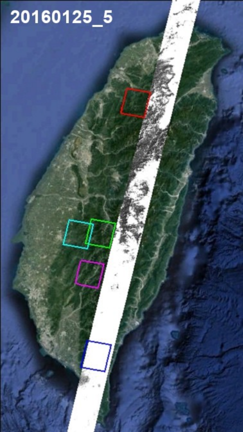
20160125_5

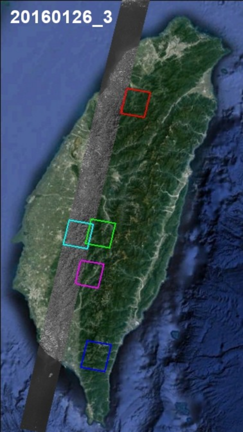
20160126_3

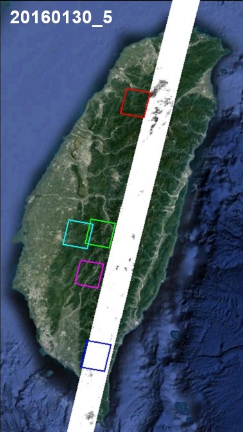
20160130_5

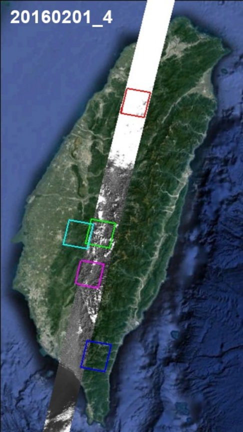
20160201_4

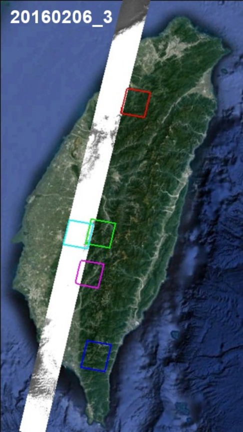
20160206_3

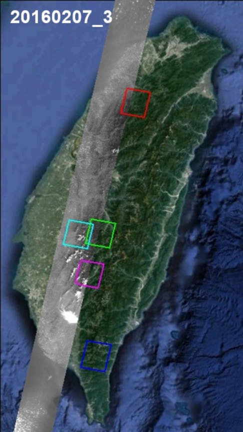
20160207_3

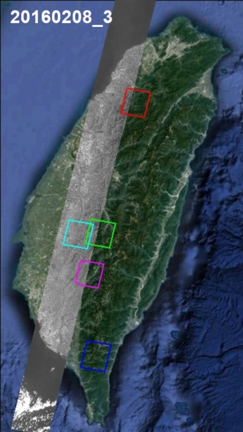
20160208_3

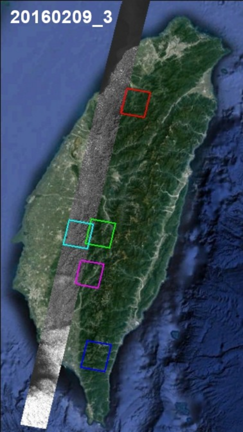
20160209_3

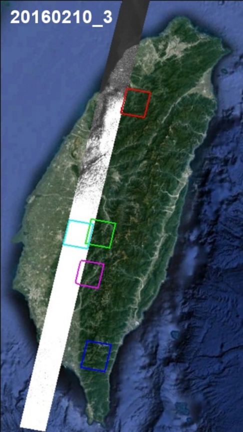
20160210_3

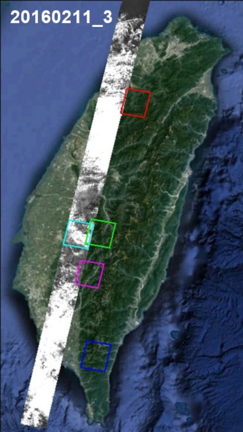
20160211_3

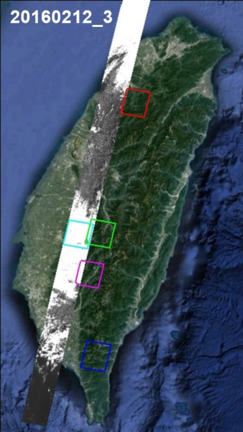
20160212_3

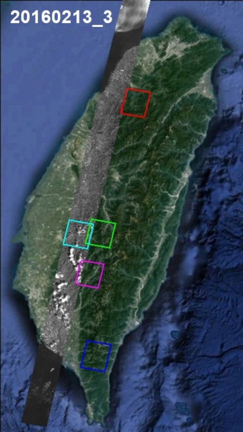
20160213_3

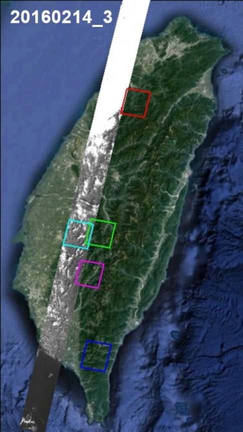
20160214_3

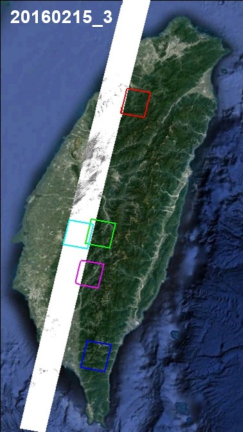
20160215_3

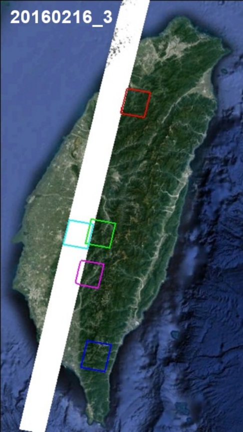
20160216_3

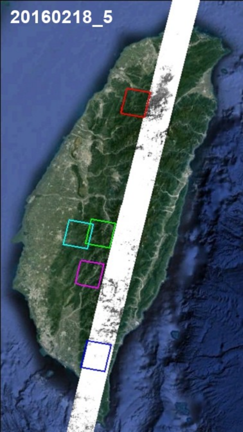
20160218_5

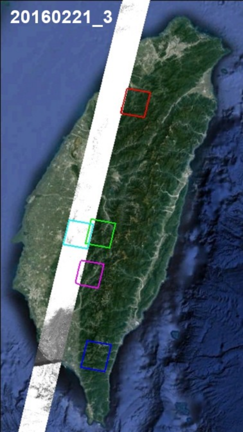
20160221_3

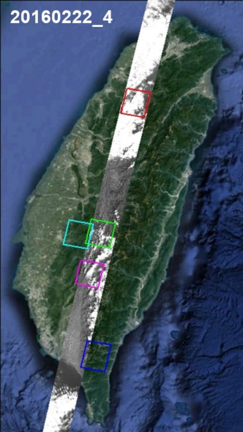
20160222_4

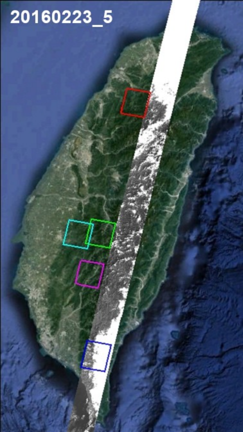
20160223_5

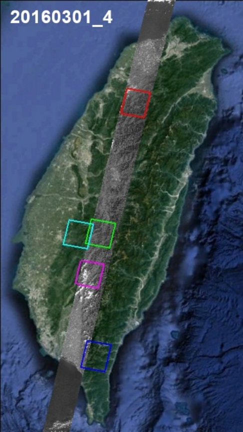
20160301_4

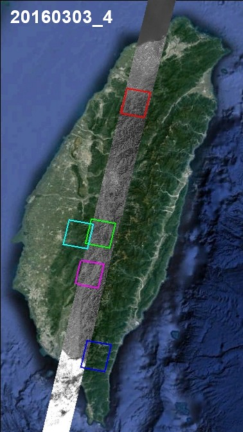
20160303_4

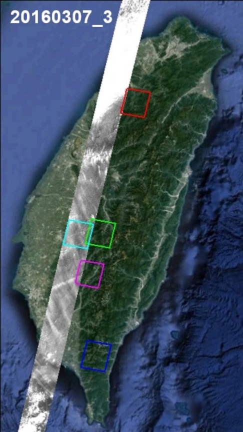
20160307_3

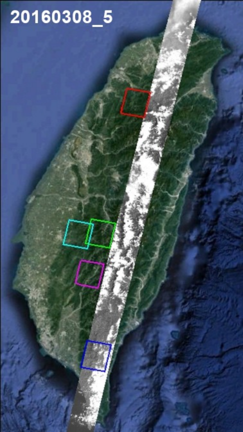
20160308_5

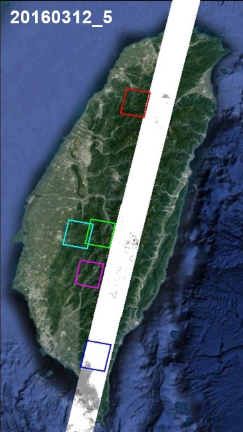
20160312_5

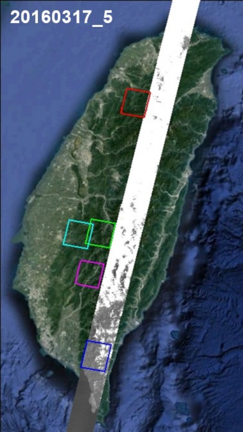
20160317_5

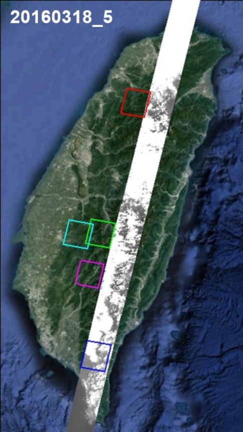
20160318_5

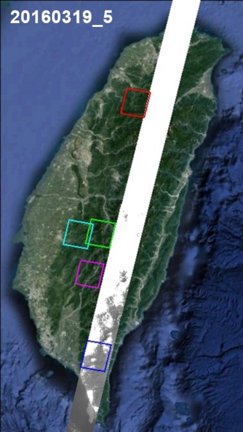
20160319_5

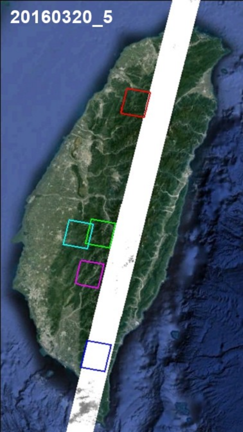
20160320_5

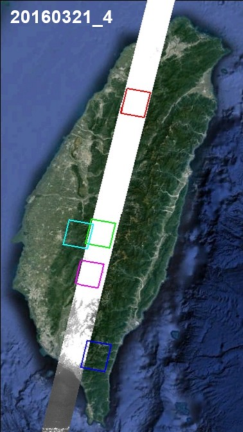
20160321_4

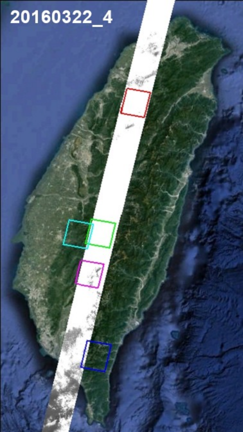
20160322_4

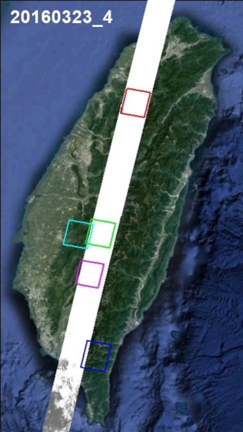
20160323_4

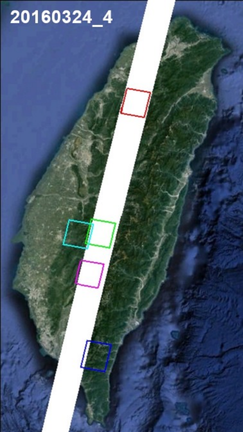
20160324_4

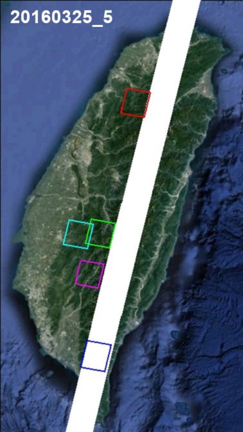
20160325_5

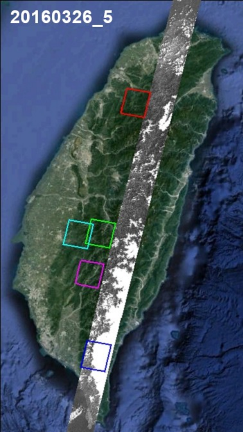
20160326_5

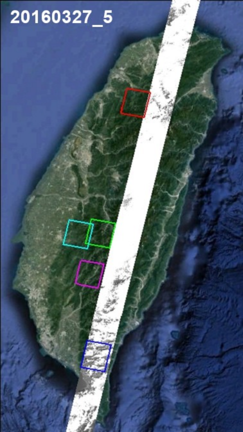
20160327_5

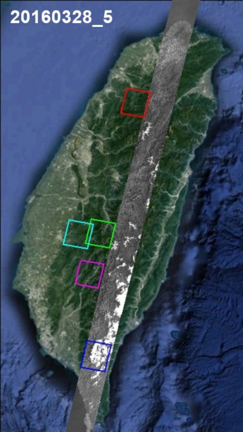
20160328_5

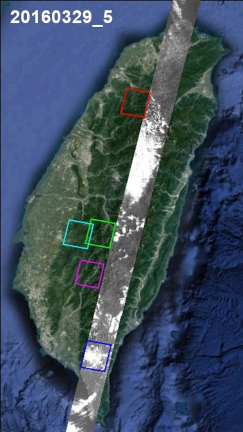
20160329_5

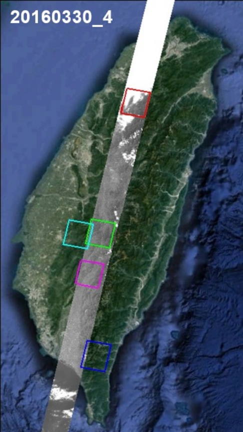
20160330_4

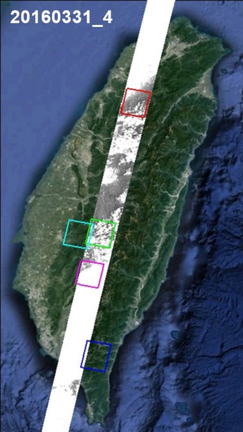
20160331_4

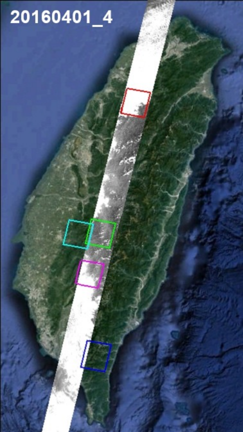
20160401_4

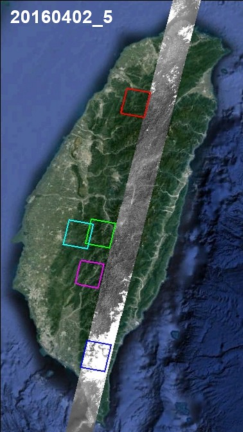
20160402_5

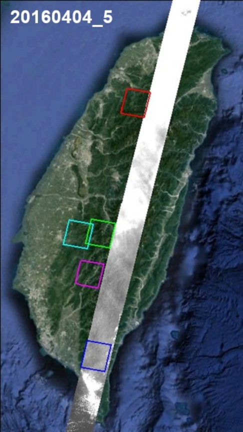
20160404_5

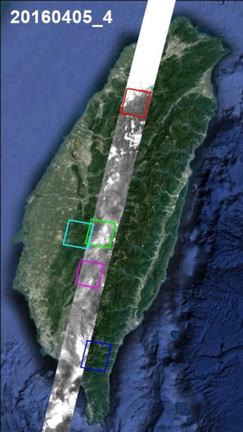
20160405_4

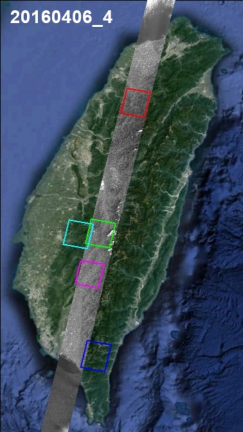
20160406_4

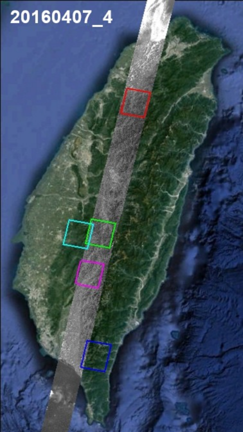
20160407_4

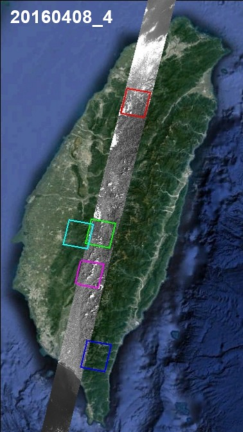
20160408_4

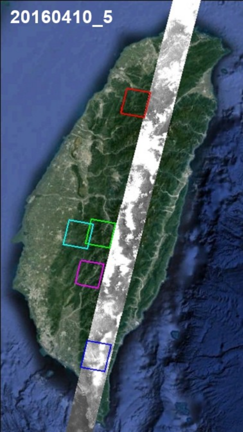
20160410_5

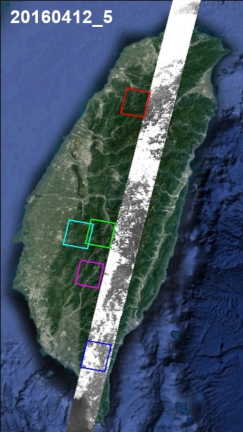
20160412_5

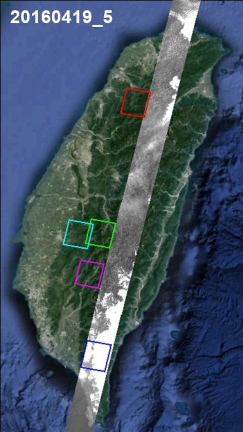
20160419_5

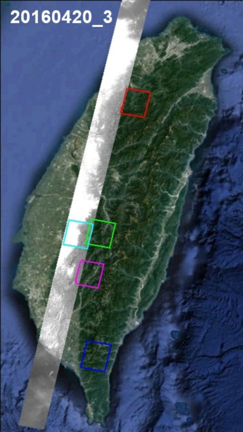
20160420_3

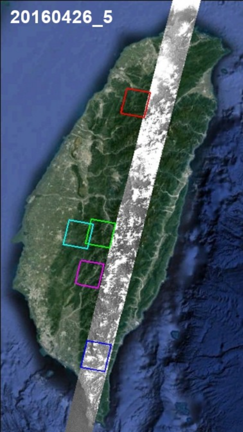
20160426_5

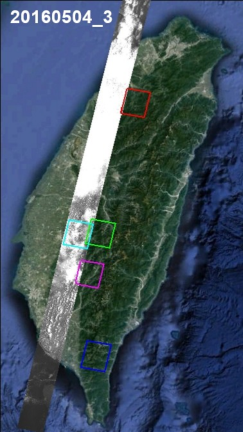
20160504_3

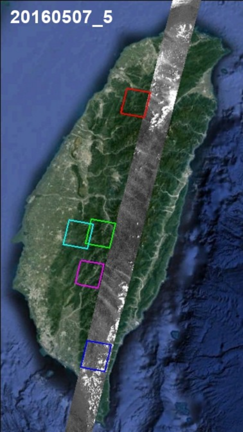
20160507_5

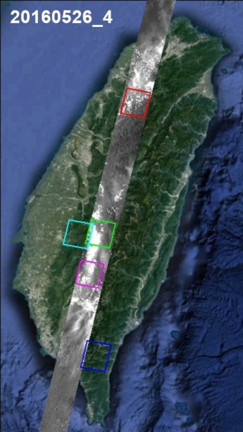
20160526_4

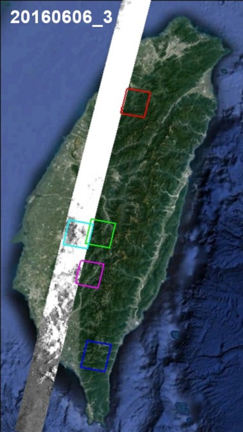
20160606_3

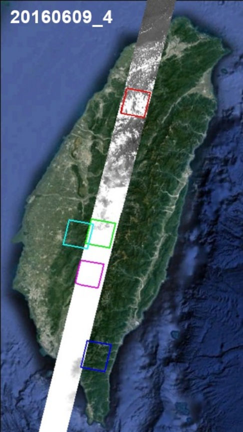
20160609_4

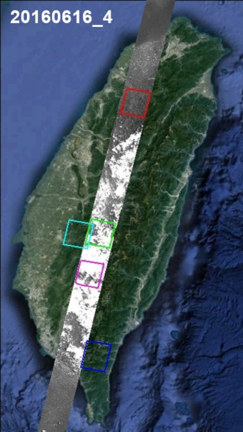
20160616_4

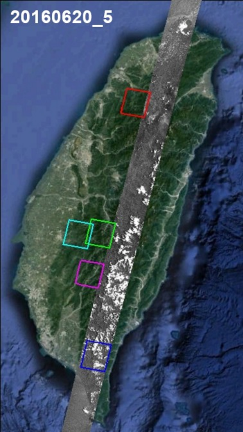
20160620_5

Supplement B. All scatter plots of the other 34 images, of which a total of 19 images with poor quality are excluded from the time series analysis.

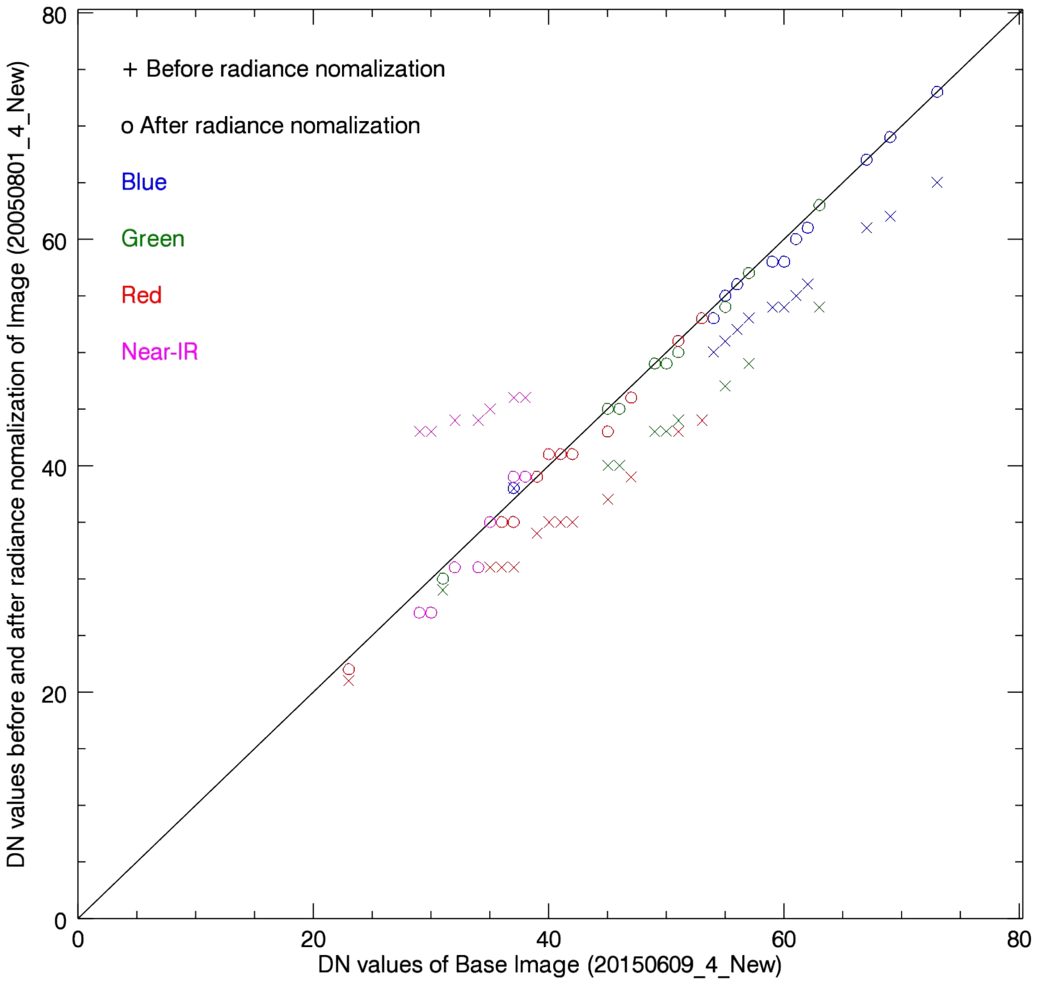
+ Before radiance nomalization
o After radiance nomalization
Blue
Green
Red
Near-IR
DN values before and after radiance nomalization of Image (20050801_4_New)
DN values of Base Image (20150609_4_New)
0
20
40
60
80
0
20
40
60
80

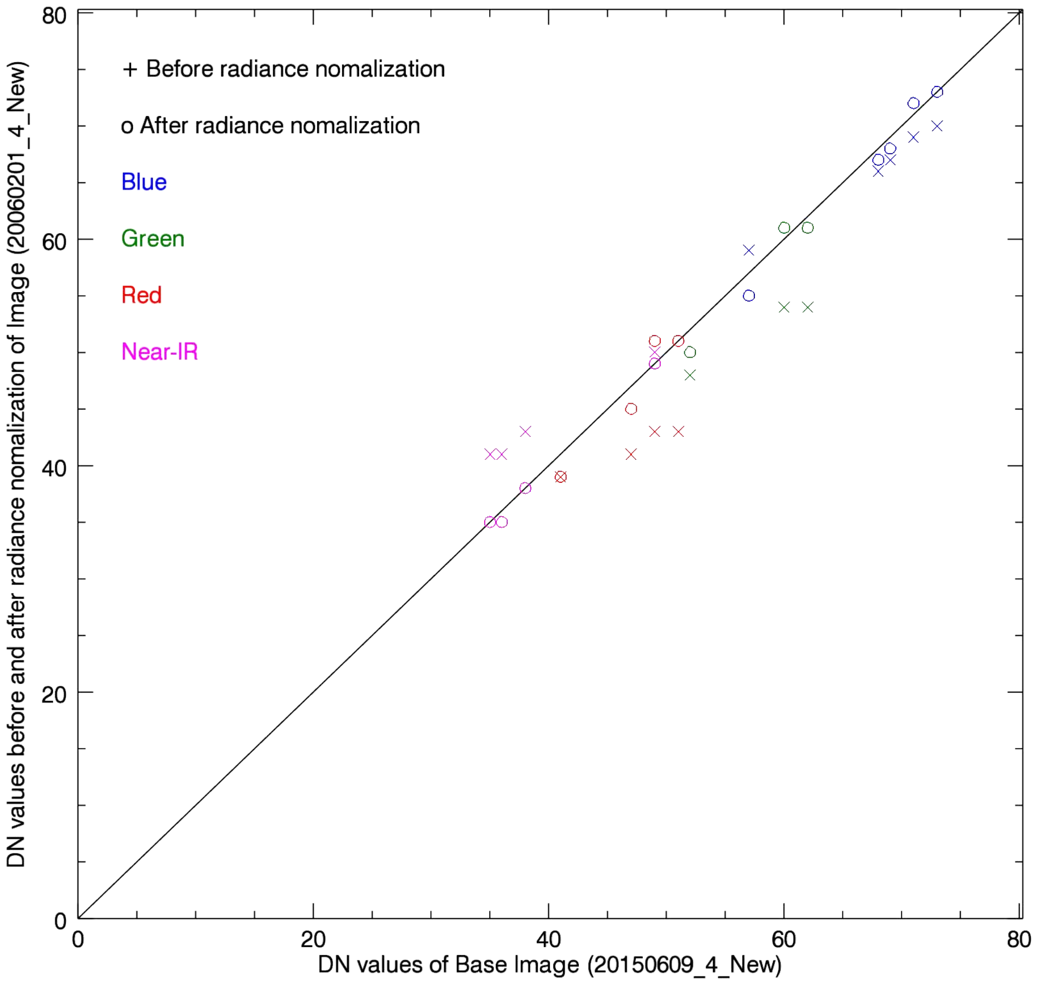

+ Before radiance nomalization
o After radiance nomalization
Blue
Green
Red
Near-IR
0
20
40
60
80
DN values of Base Image (20150609_4_New)
DN values before and after radiance nomalization of Image (20060201_4_New)

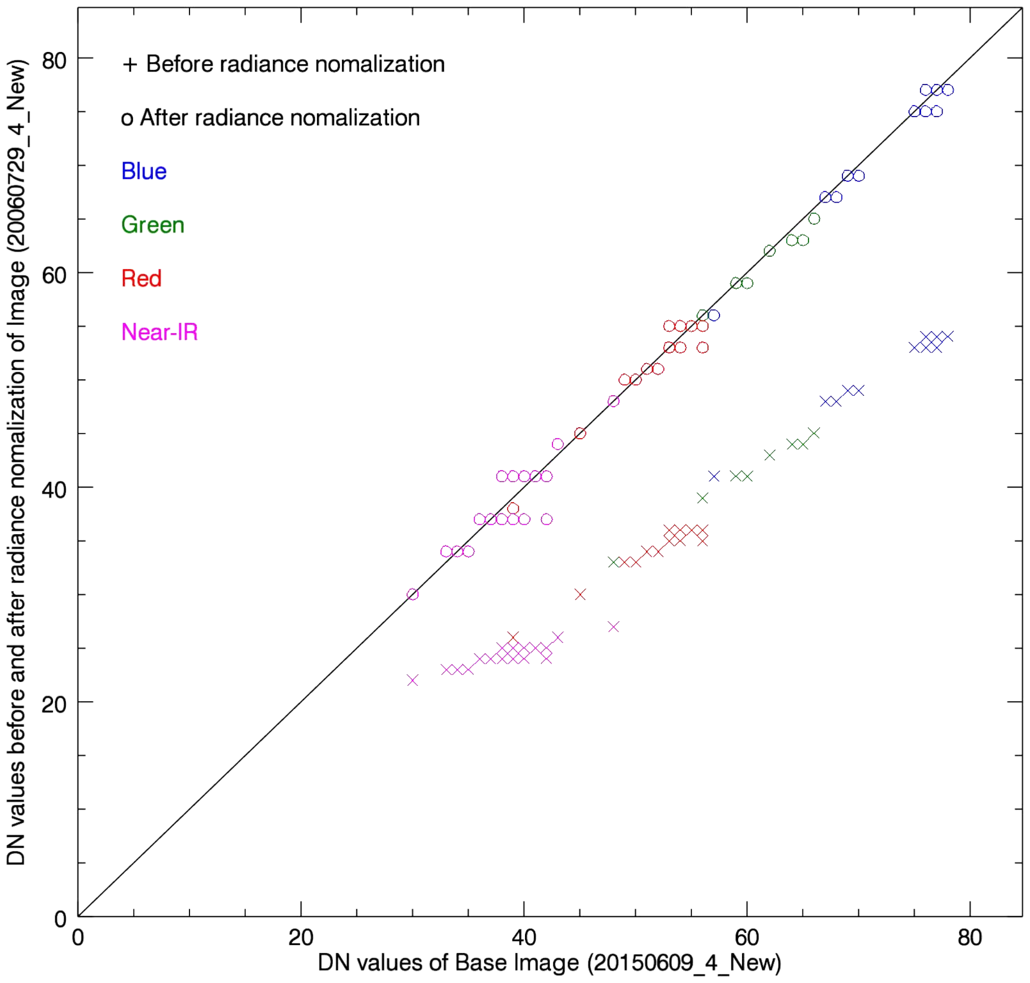

+ Before radiance nomalization
o After radiance nomalization
Blue
Green
Red
Near-IR
DN values before and after radiance nomalization of Image (20060729_4_New)
DN values of Base Image (20150609_4_New)
0
20
40
60
80

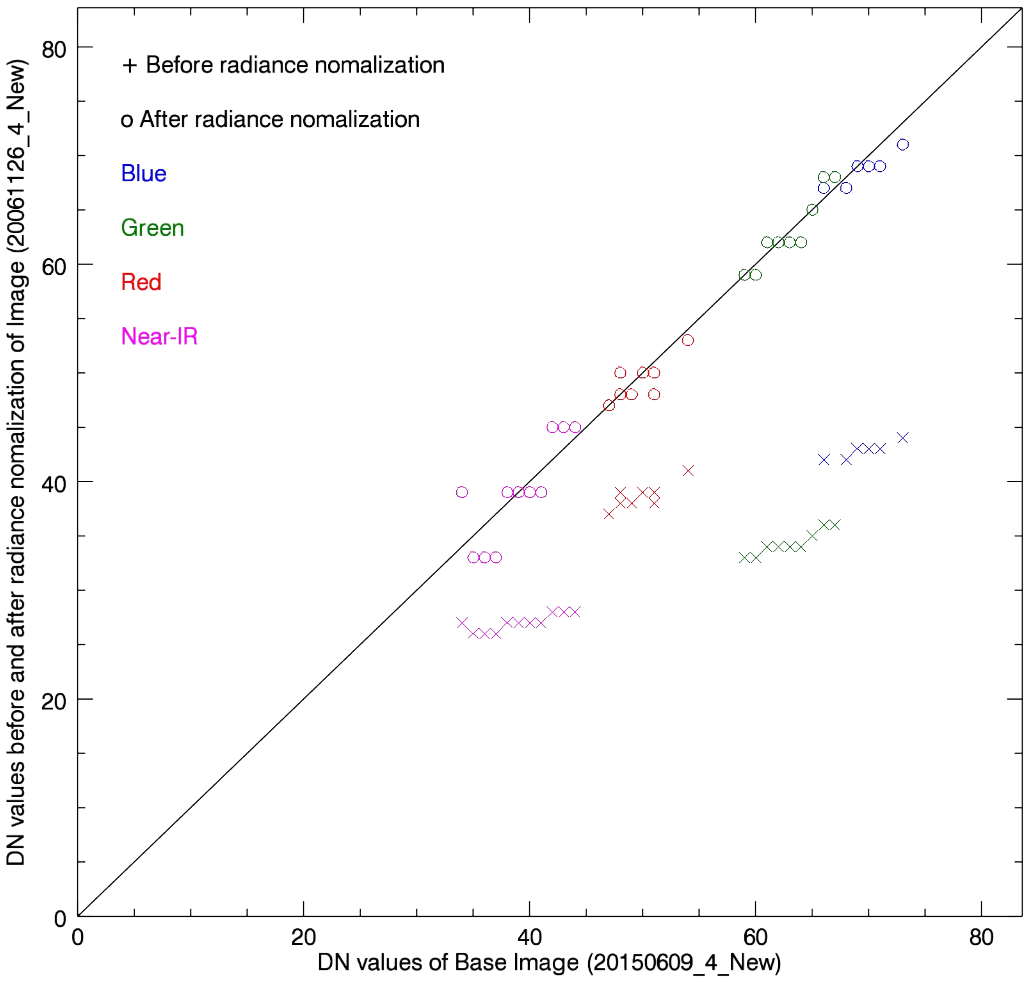
+ Before radiance nomalization
o After radiance nomalization
Blue
Green
Red
Near-IR
DN values before and after radiance nomalization of Image (20061126_4_New)
DN values of Base Image (20150609_4_New)
0
20
40
60
80
0
20
40
60
80

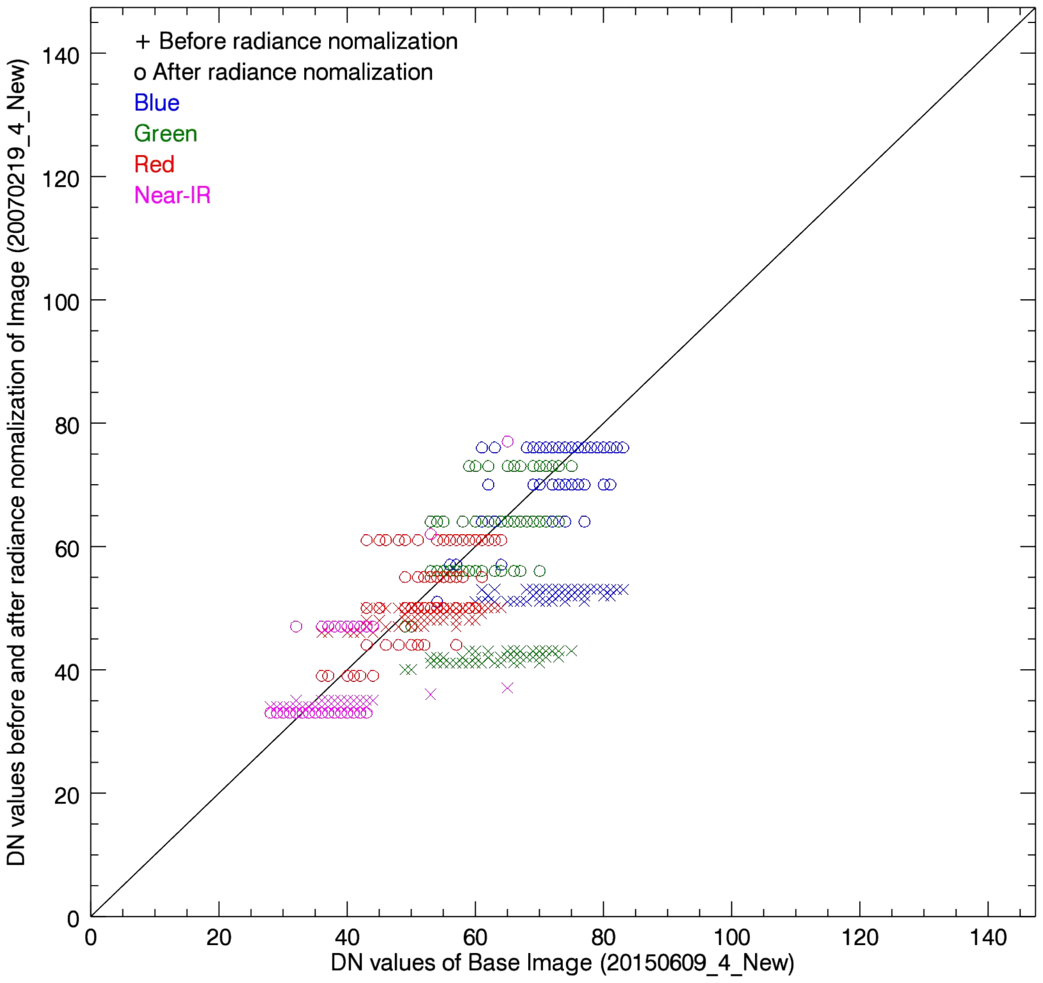

+ Before radiance nomalization
o After radiance nomalization
Blue
Green
Red
Near-IR
DN values before and after radiance nomalization of Image (20070219_4_New)
DN values of Base Image (20150609_4_New)
0
20
40
60
80
100
120
140

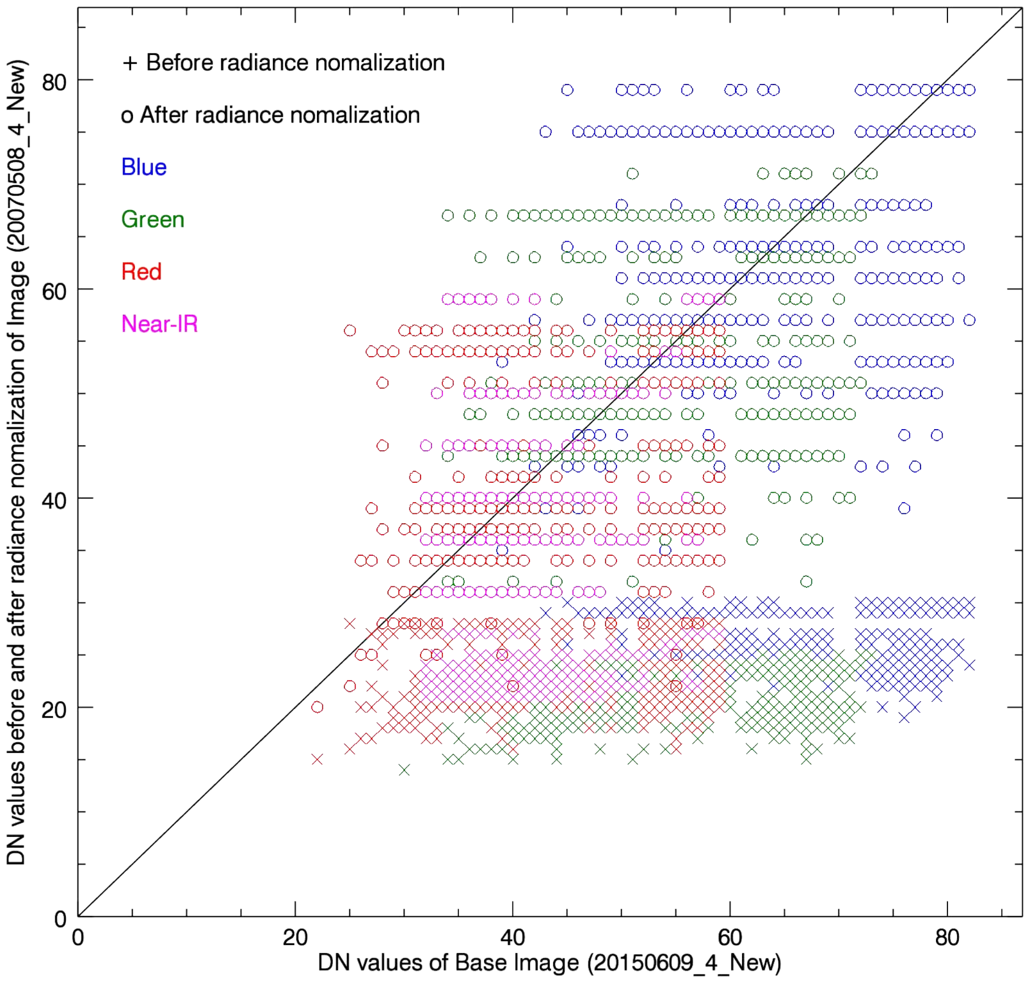

+ Before radiance nomalization
o After radiance nomalization
Blue
Green
Red
Near-IR
DN values before and after radiance nomalization of Image (20070508_4_New)
DN values of Base Image (20150609_4_New)
0
20
40
60
80

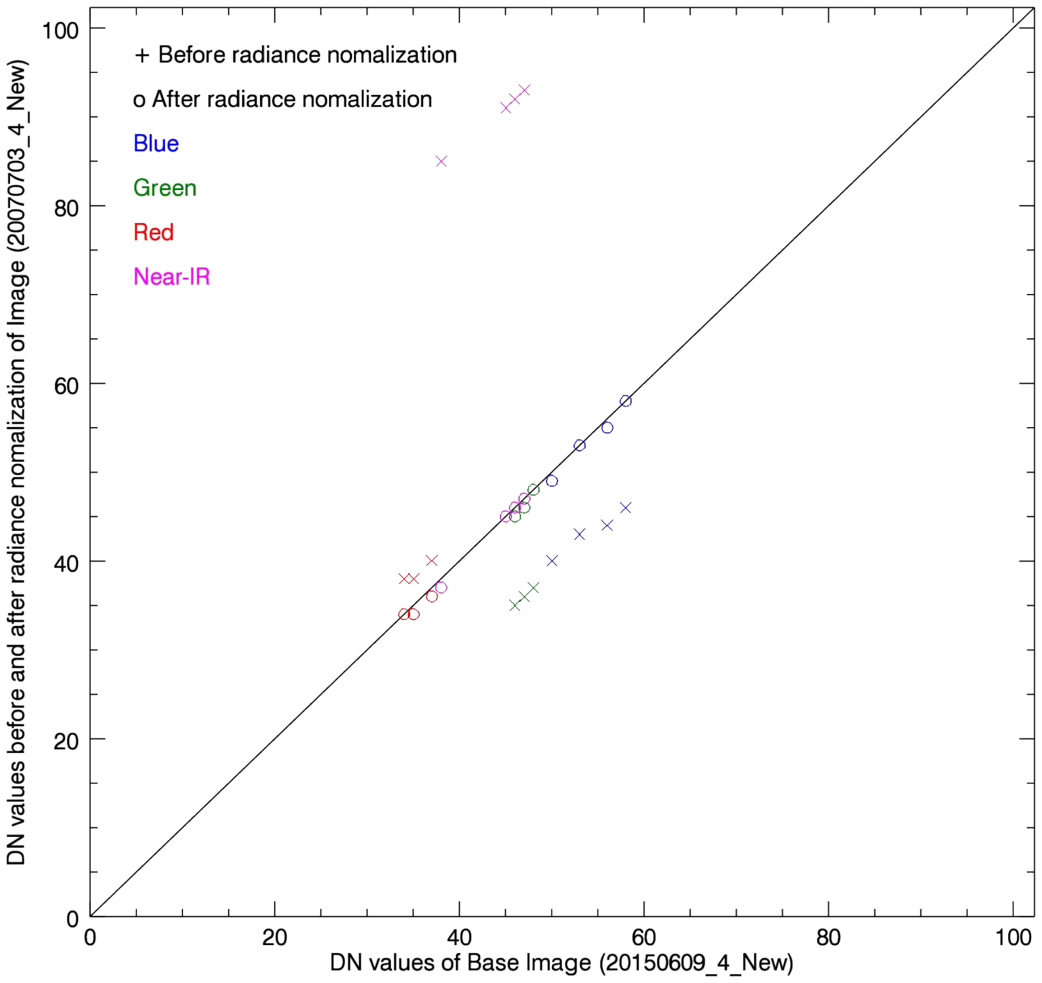
+ Before radiance nomalization
o After radiance nomalization
Blue
Green
Red
Near-IR
DN values before and after radiance nomalization of Image (20070703_4_New)
DN values of Base Image (20150609_4_New)
0
20
40
60
80
100
0
20
40
60
80
100

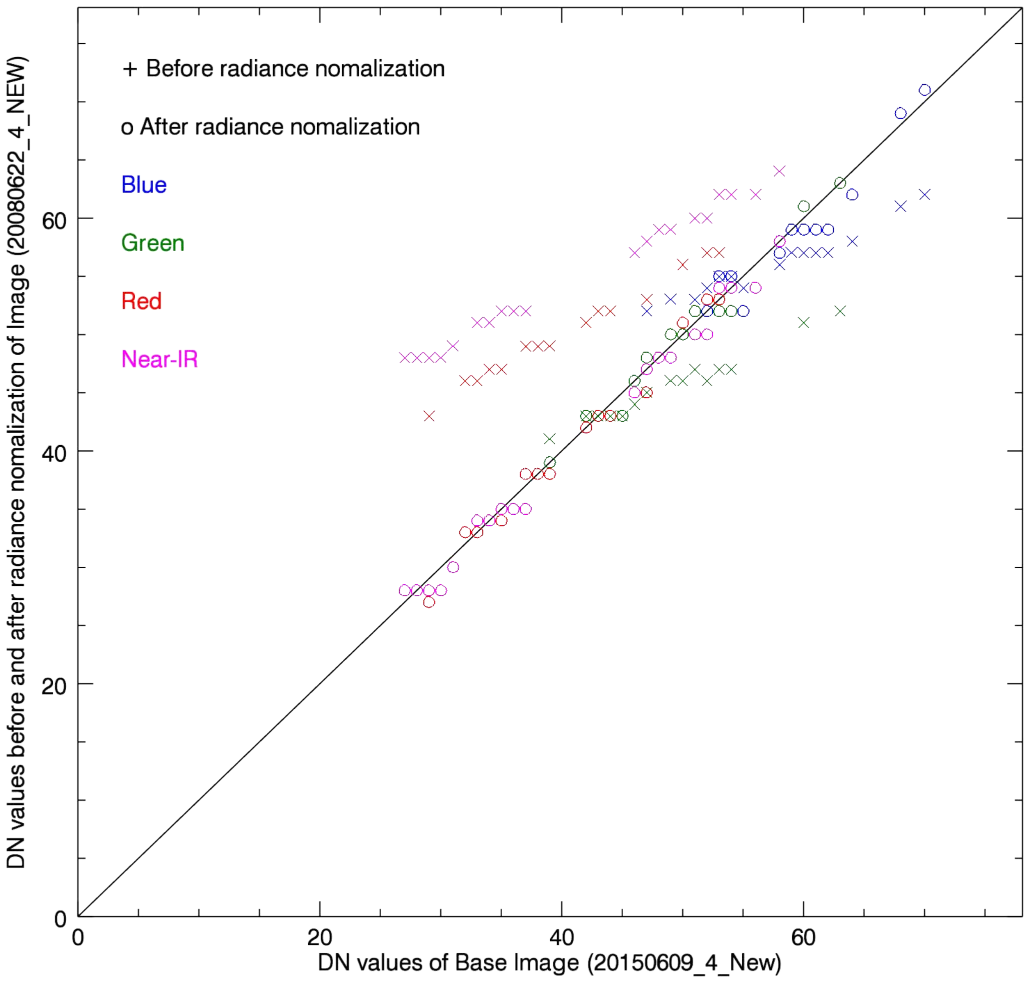
+ Before radiance nomalization
o After radiance nomalization
Blue
Green
Red
Near-IR
DN values before and after radiance nomalization of Image (20080622_4_NEW)
DN values of Base Image (20150609_4_New)
0
20
40
60
0
20
40
60

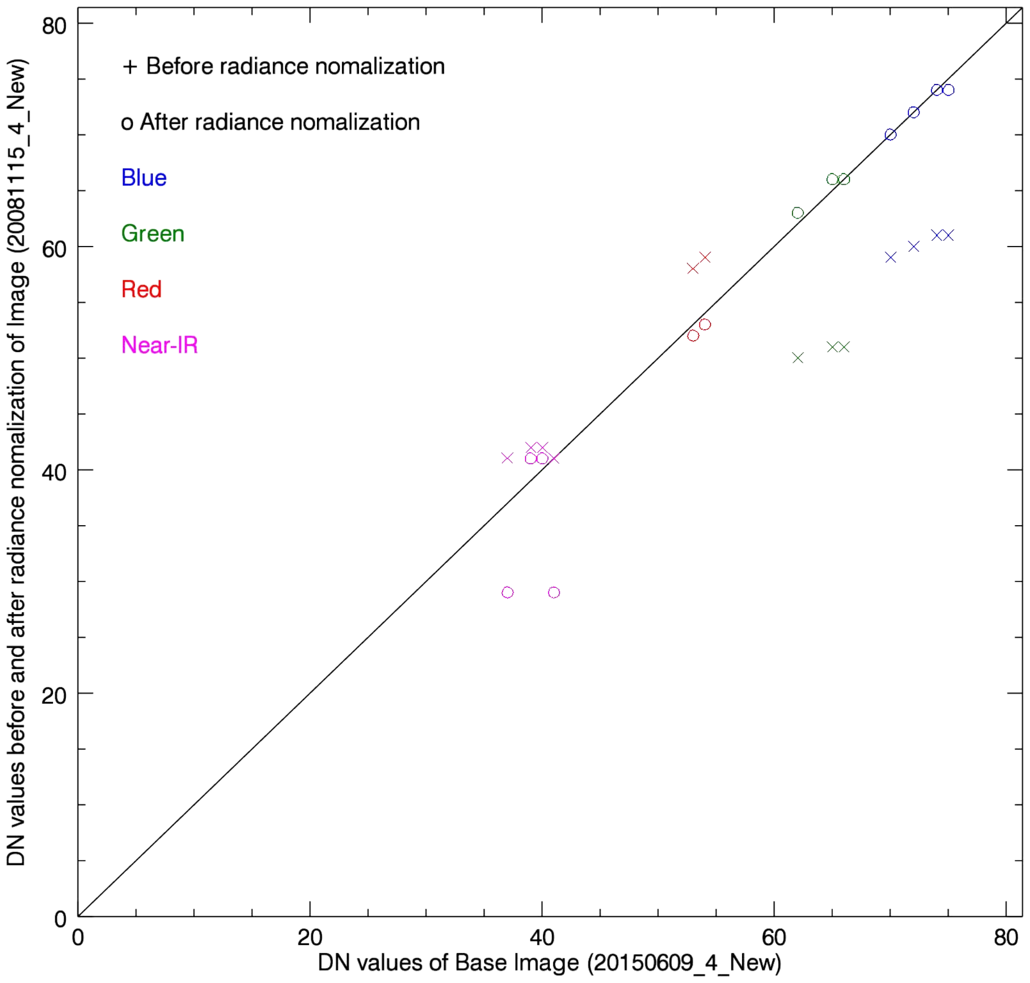

+ Before radiance nomalization
o After radiance nomalization
Blue
Green
Red
Near-IR
DN values before and after radiance nomalization of Image (20081115_4_New)
DN values of Base Image (20150609_4_New)
0
20
40
60
80

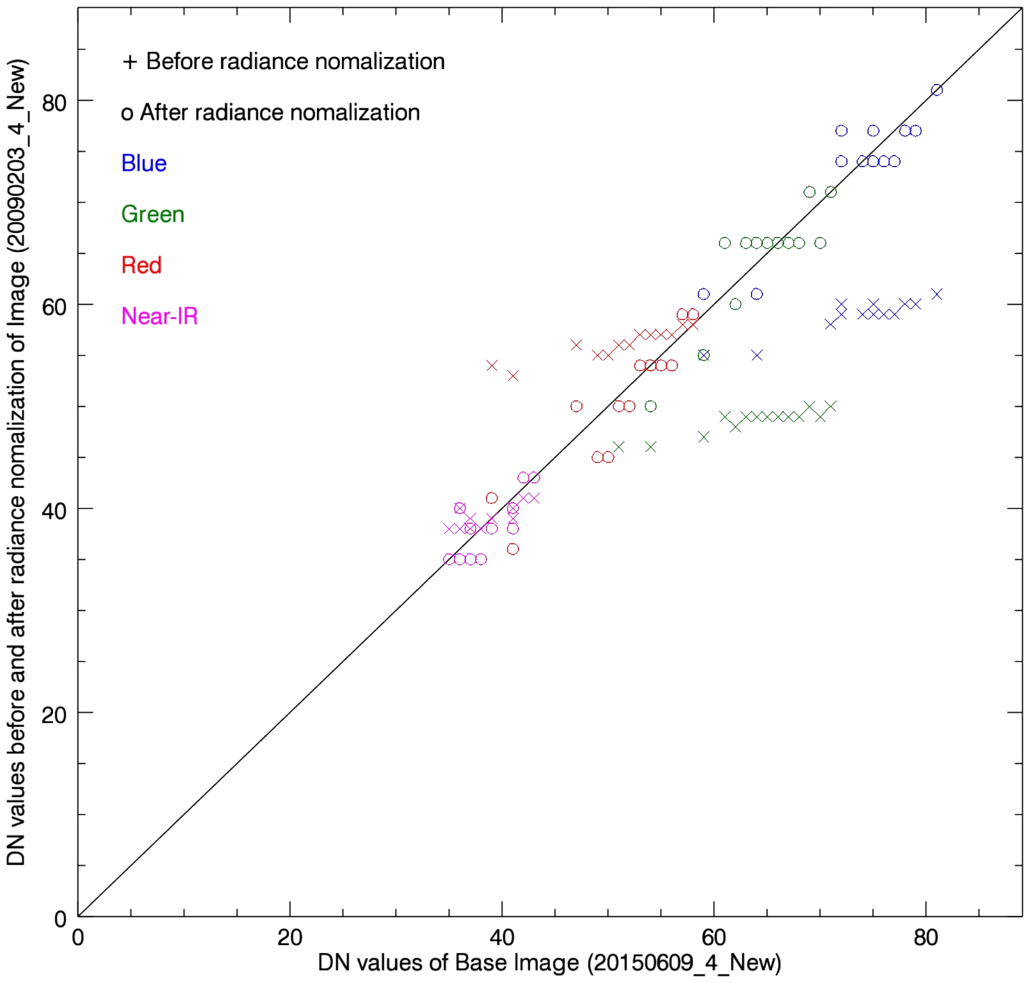
+ Before radiance nomalization
o After radiance nomalization
Blue
Green
Red
Near-IR
DN values before and after radiance nomalization of Image (20090203_4_New)
DN values of Base Image (20150609_4_New)
0
20
40
60
80
0
20
40
60
80

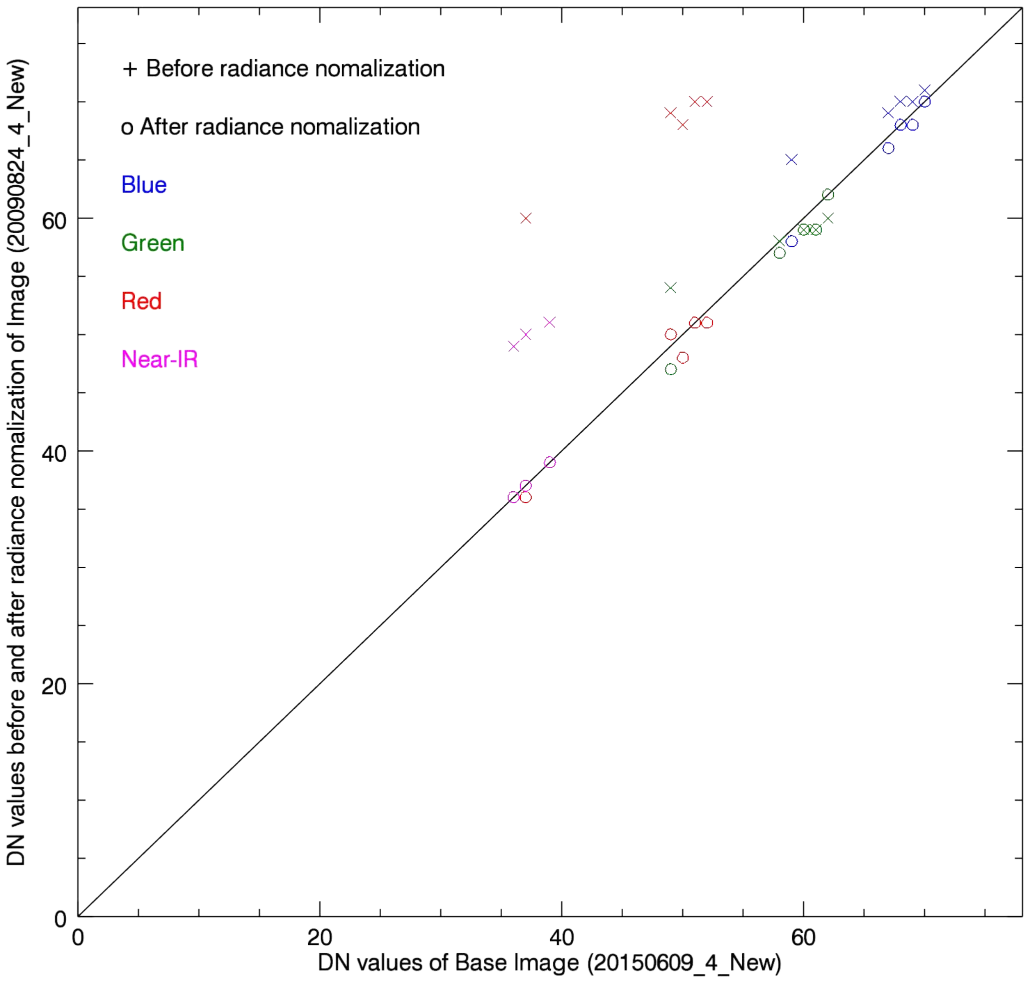
+ Before radiance nomalization
o After radiance nomalization
Blue
Green
Red
Near-IR
0
20
40
60
0
20
40
60
DN values of Base Image (20150609_4_New)
DN values before and after radiance nomalization of Image (20090824_4_New)

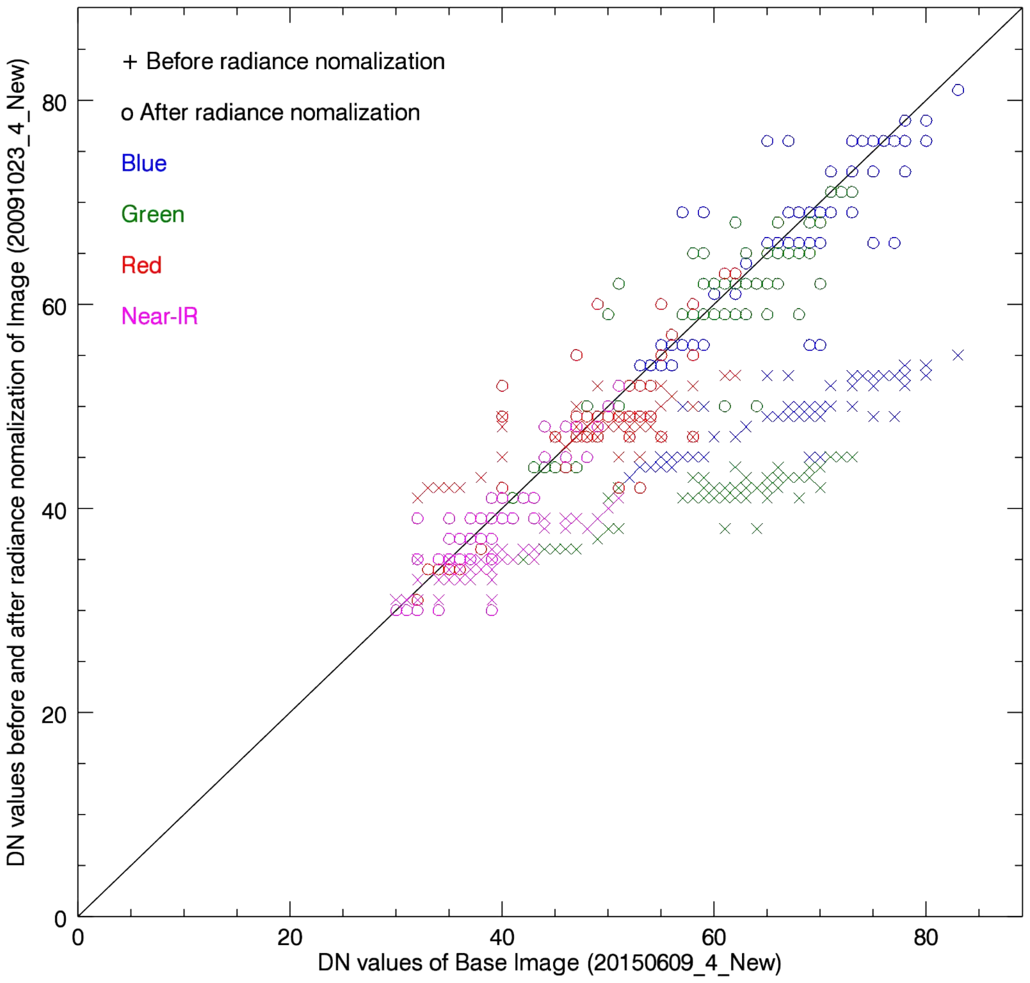
+ Before radiance nomalization
o After radiance nomalization
Blue
Green
Red
Near-IR
DN values before and after radiance nomalization of Image (20091023_4_New)
DN values of Base Image (20150609_4_New)
0
20
40
60
80

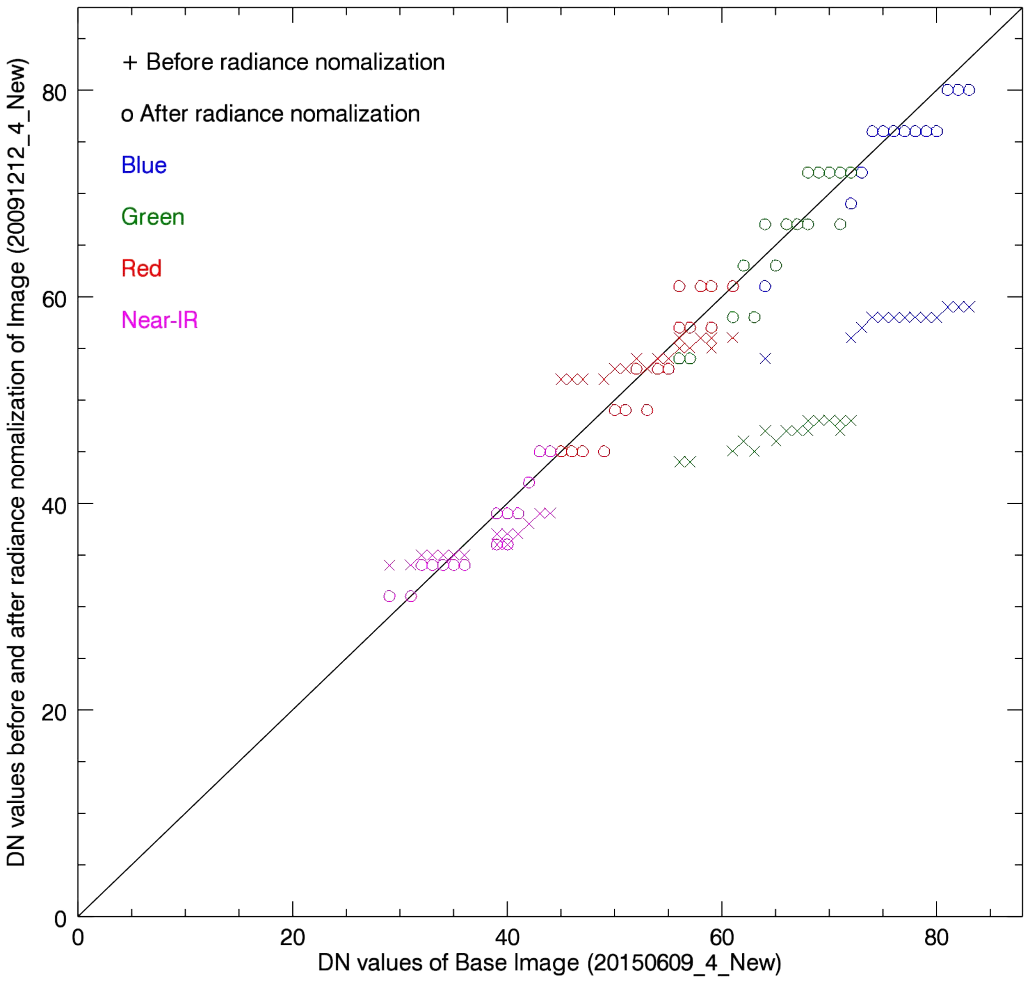
+ Before radiance nomalization
o After radiance nomalization
Blue
Green
Red
Near-IR
DN values before and after radiance nomalization of Image (20091212_4_New)
DN values of Base Image (20150609_4_New)
0
20
40
60
80

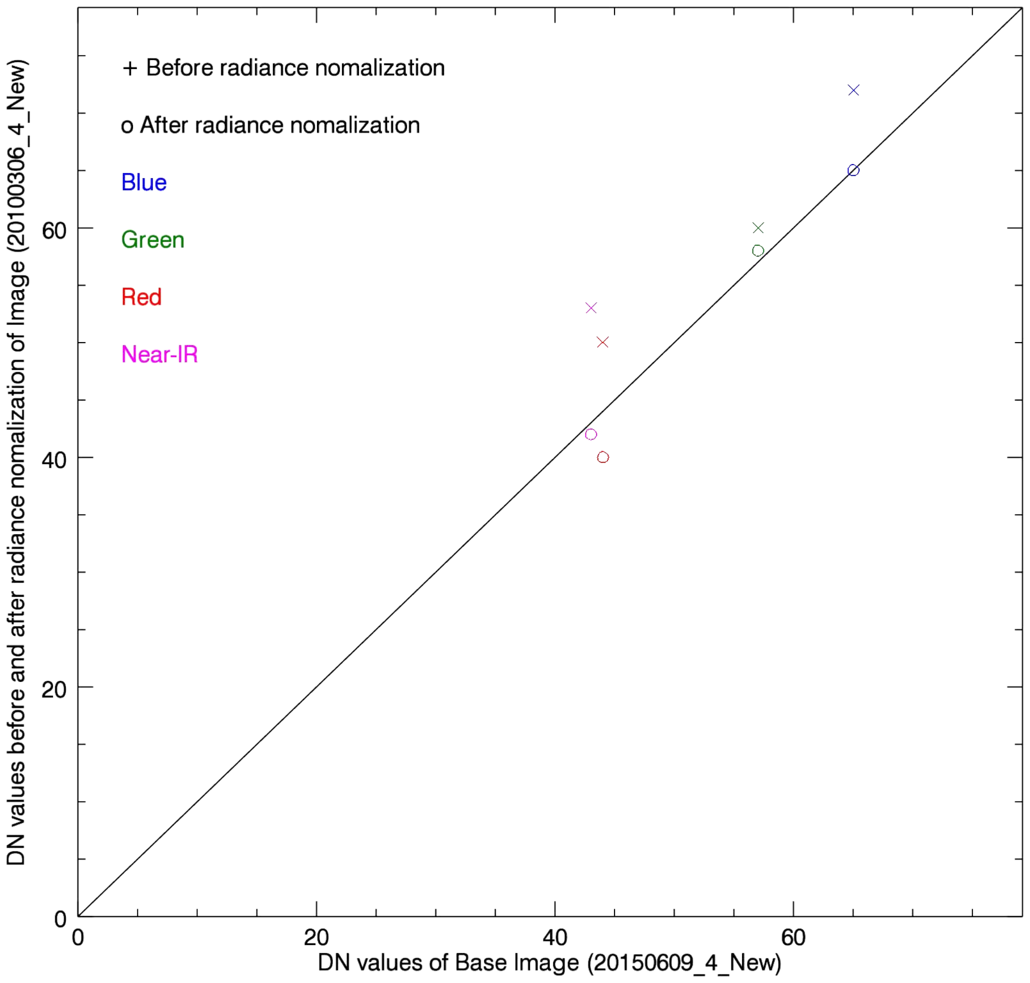

+ Before radiance nomalization
o After radiance nomalization
Blue
Green
Red
Near-IR
DN values before and after radiance nomalization of Image (20100306_4_New)
DN values of Base Image (20150609_4_New)
0
20
40
60
0
20
40
60

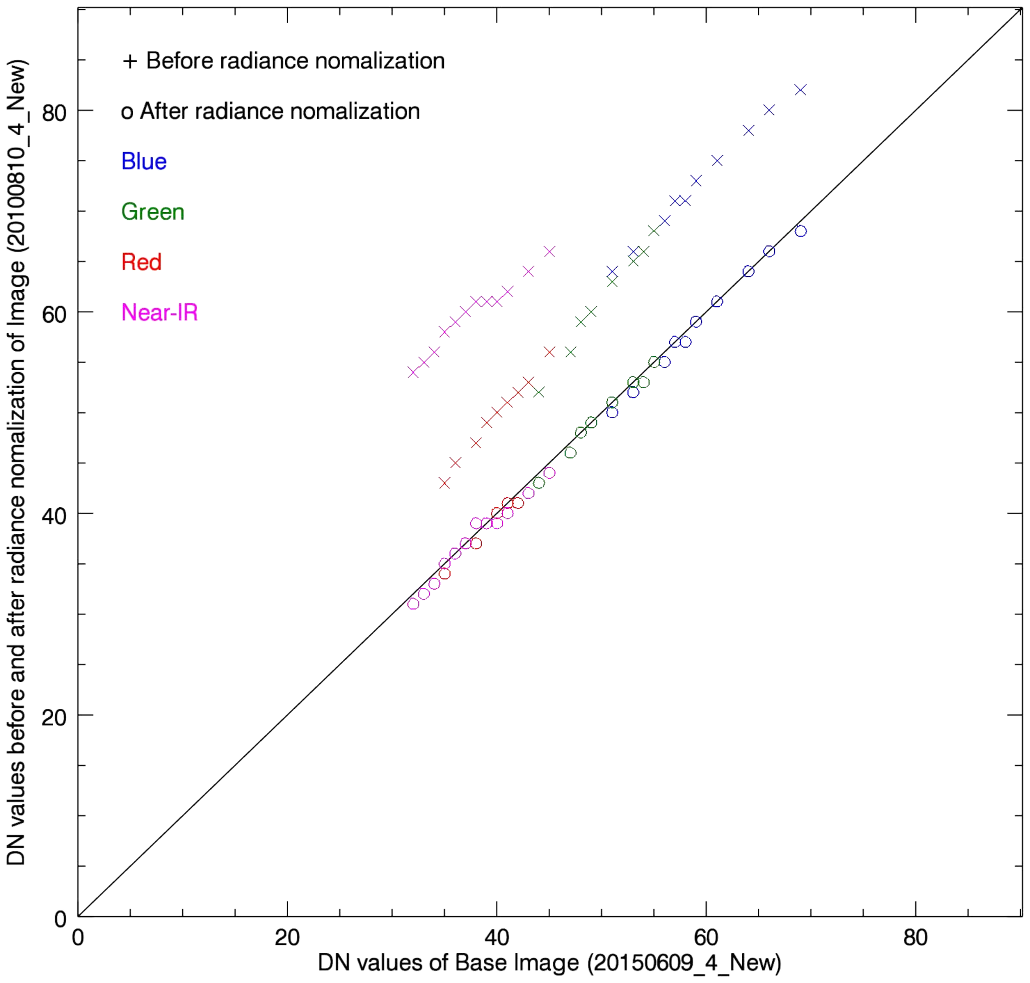
+ Before radiance nomalization
o After radiance nomalization
Blue
Green
Red
Near-IR
DN values before and after radiance nomalization of Image (20100810_4_New)
DN values of Base Image (20150609_4_New)
0
20
40
60
80
0
20
40
60
80

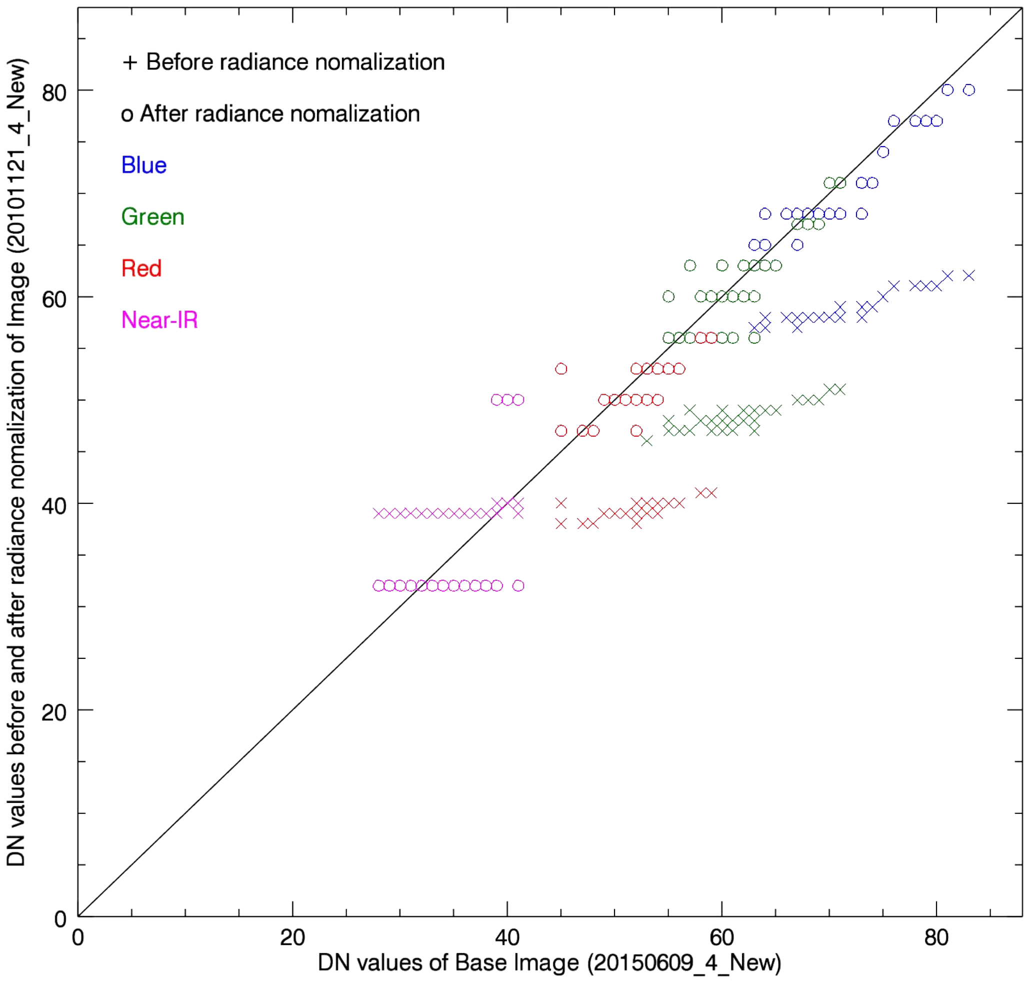
+ Before radiance nomalization
o After radiance nomalization
Blue
Green
Red
Near-IR
DN values before and after radiance nomalization of Image (20101121_4_New)
DN values of Base Image (20150609_4_New)
0
20
40
60
80

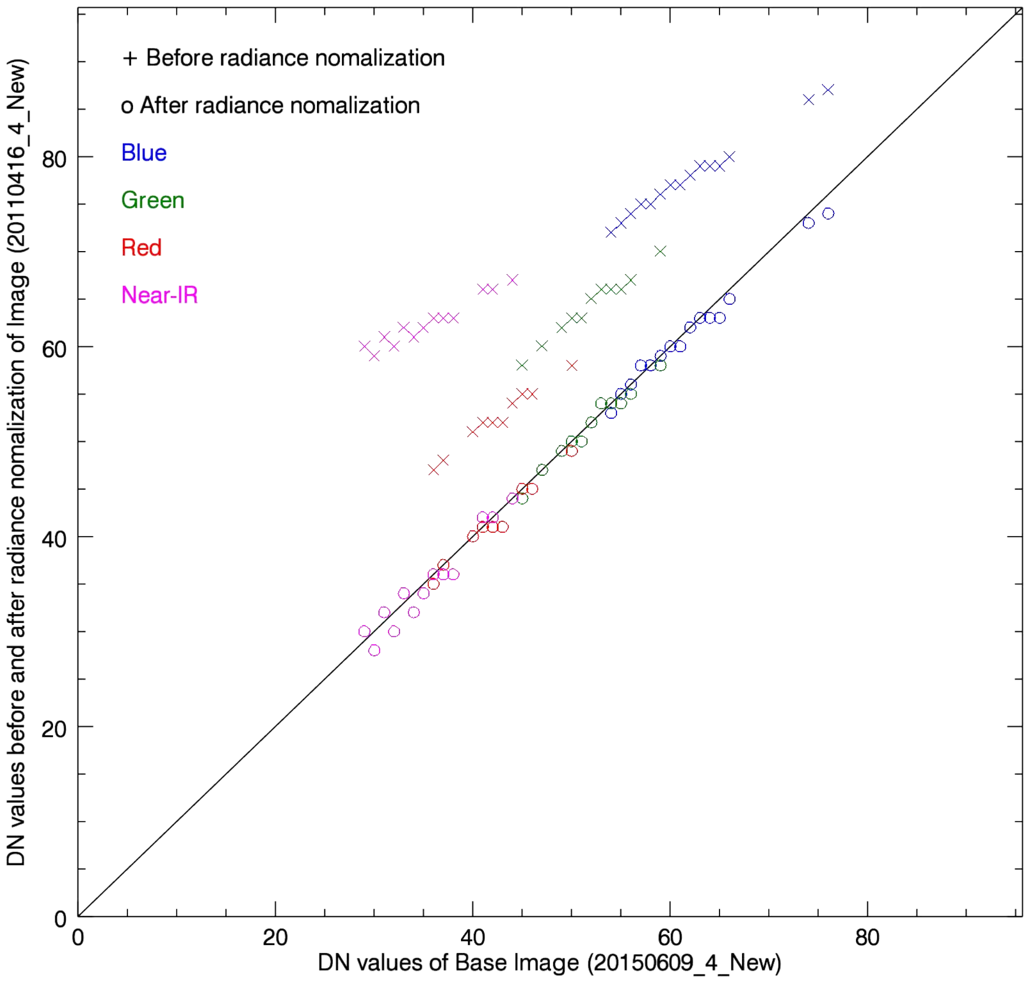

+ Before radiance nomalization
o After radiance nomalization
Blue
Green
Red
Near-IR
DN values before and after radiance nomalization of Image (20110416_4_New)
DN values of Base Image (20150609_4_New)
0
20
40
60
80

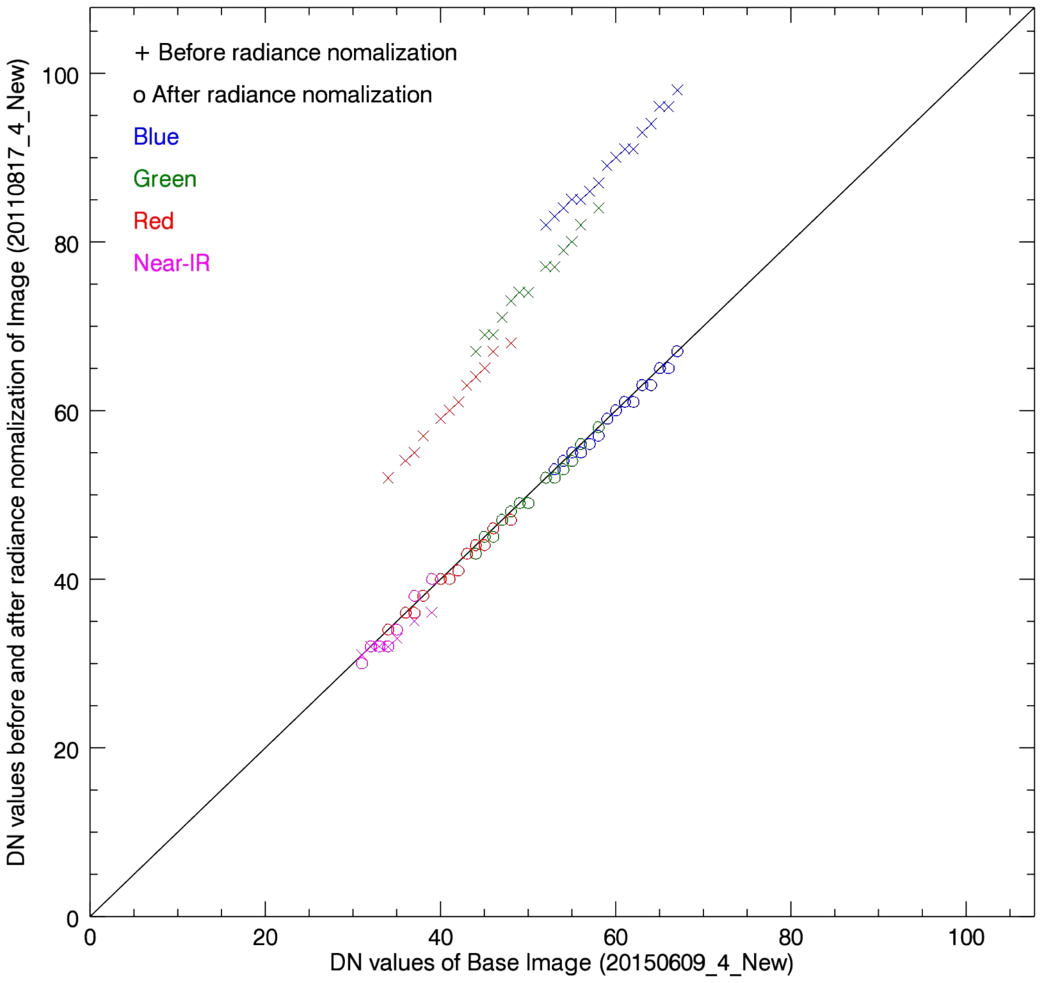

+ Before radiance nomalization
o After radiance nomalization
Blue
Green
Red
Near-IR
DN values before and after radiance nomalization of Image (20110817_4_New)
DN values of Base Image (20150609_4_New)
0
20
40
60
80
100

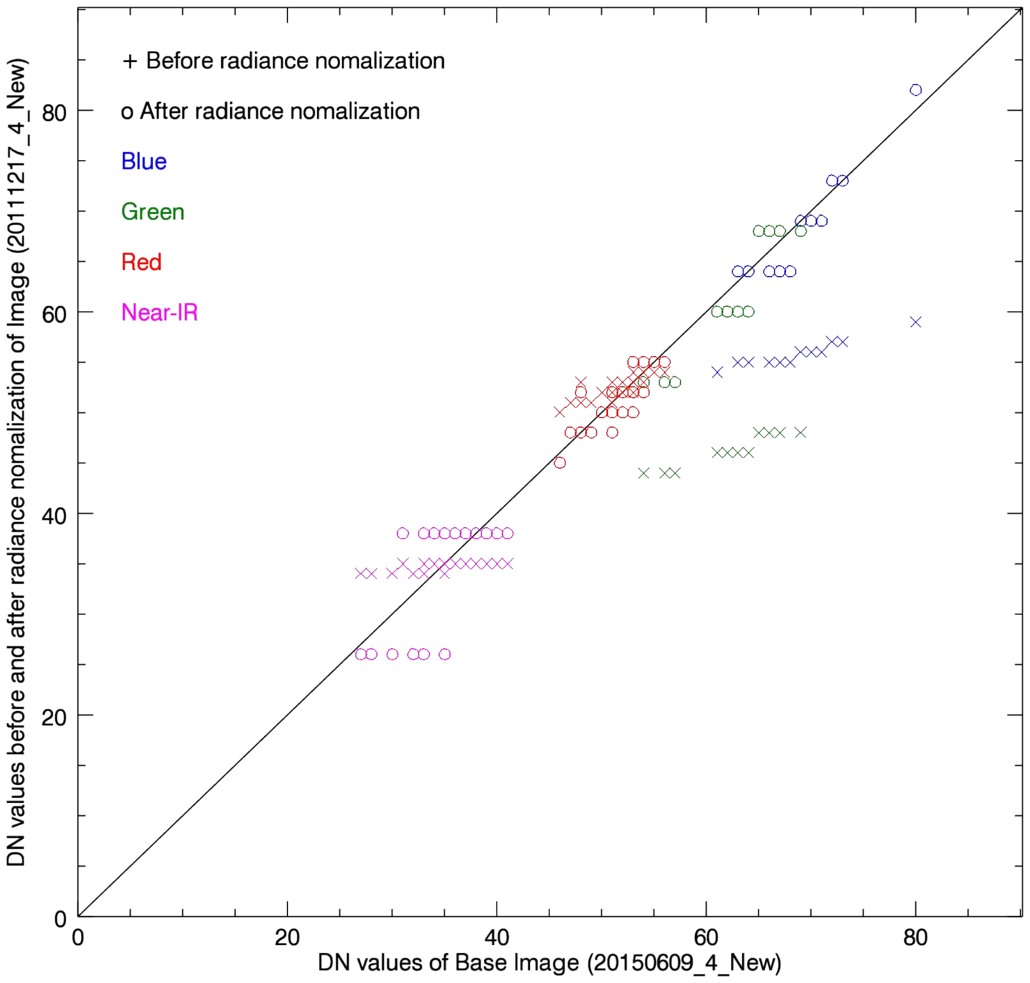

+ Before radiance nomalization
o After radiance nomalization
Blue
Green
Red
Near-IR
DN values before and after radiance nomalization of Image (20111217_4_New)
DN values of Base Image (20150609_4_New)
0
20
40
60
80

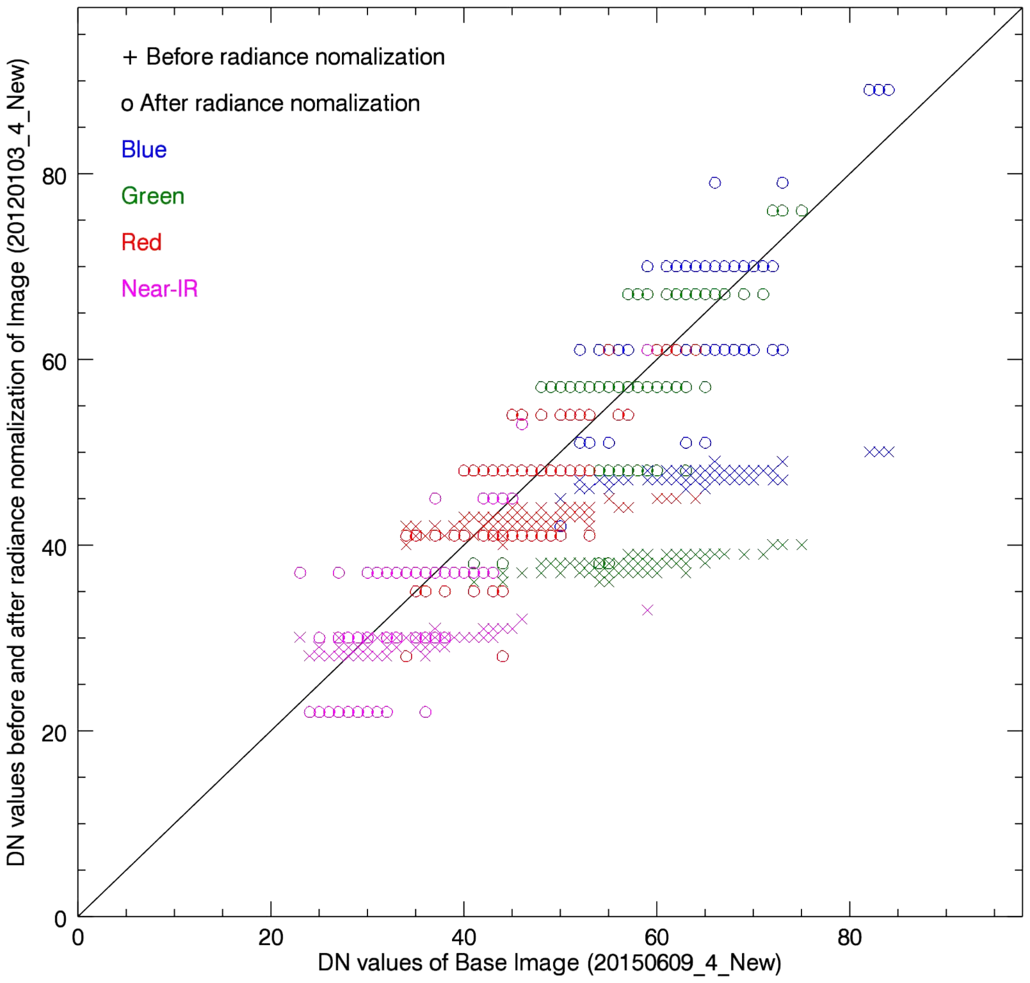
+ Before radiance nomalization
o After radiance nomalization
Blue
Green
Red
Near-IR
DN values before and after radiance nomalization of Image (20120103_4_New)
DN values of Base Image (20150609_4_New)
0
20
40
60
80

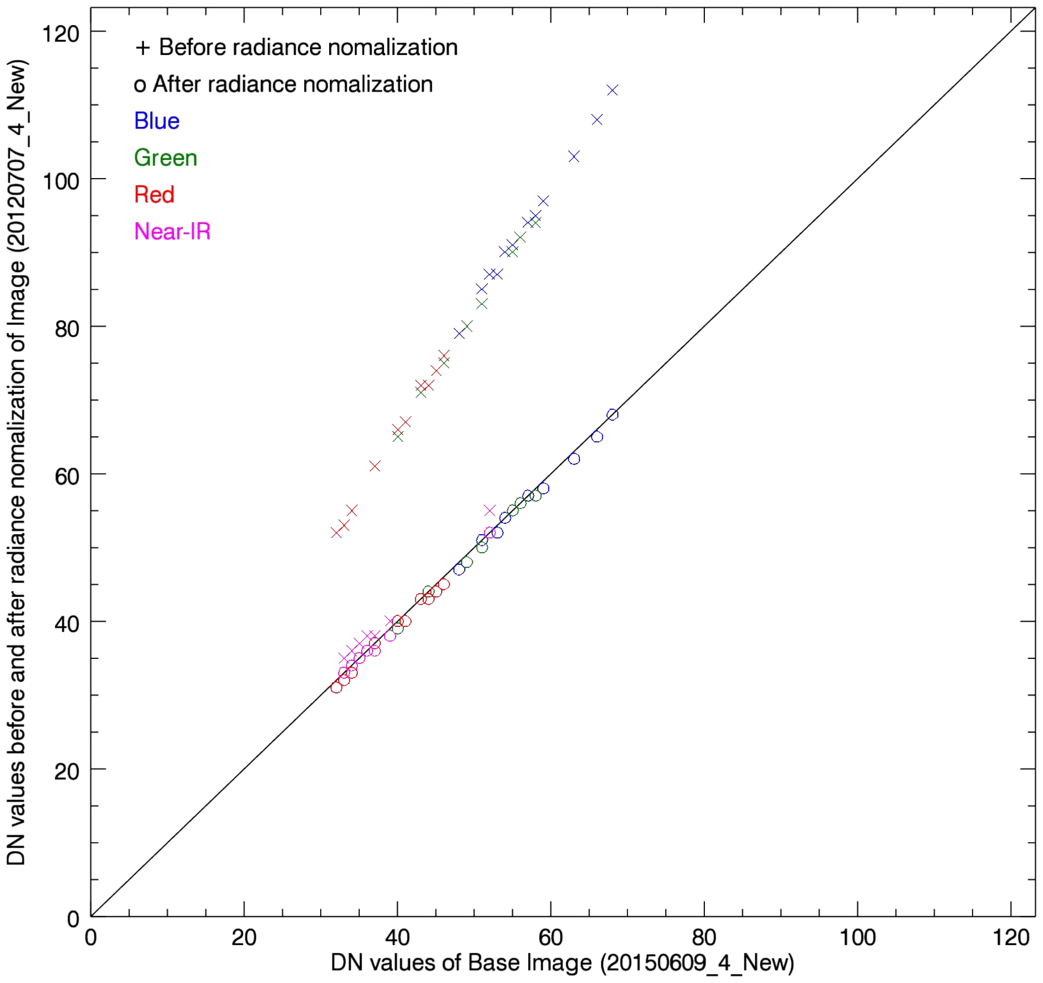

+ Before radiance nomalization
o After radiance nomalization
Blue
Green
Red
Near-IR
DN values before and after radiance nomalization of Image (20120707_4_New)
DN values of Base Image (20150609_4_New)
0
20
40
60
80
100
120

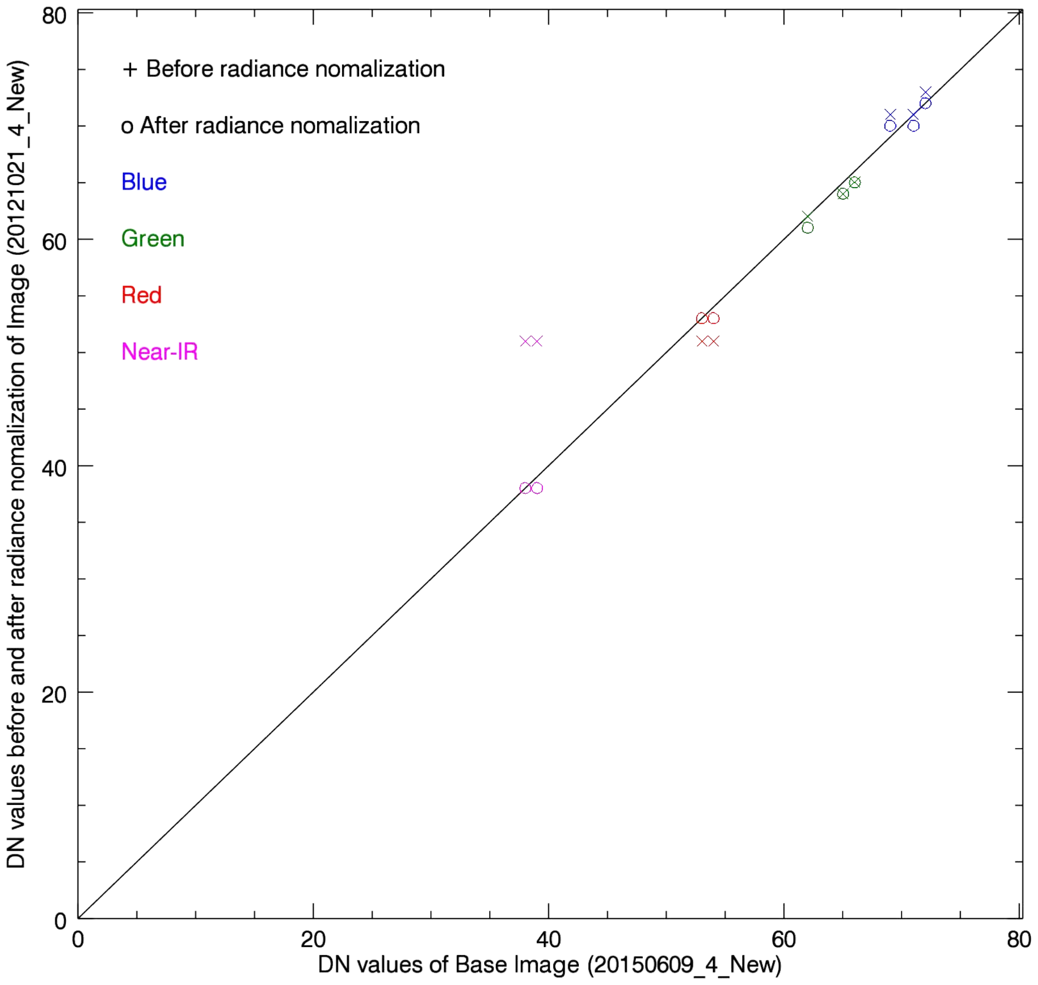

+ Before radiance nomalization
o After radiance nomalization
Blue
Green
Red
Near-IR
DN values before and after radiance nomalization of Image (20121021_4_New)
DN values of Base Image (20150609_4_New)
0
20
40
60
80
0
20
40
60
80

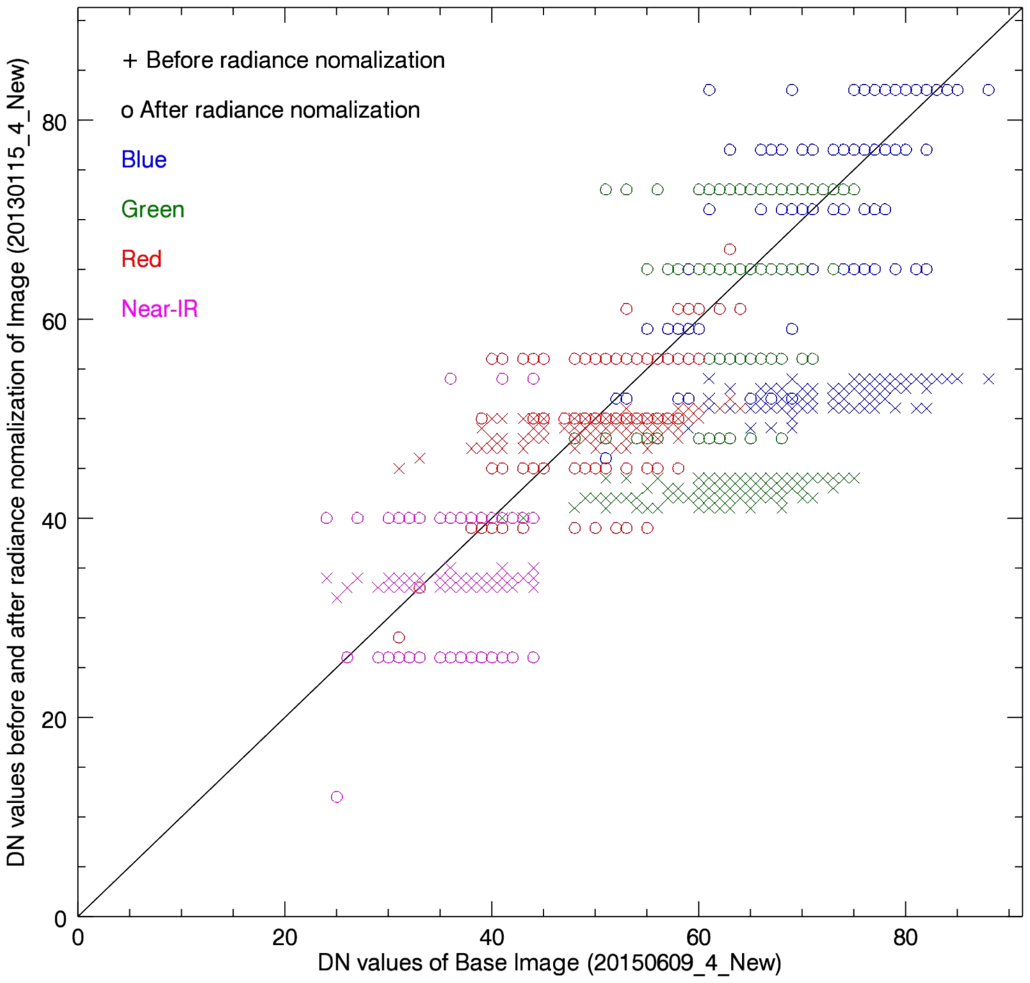
+ Before radiance nomalization
o After radiance nomalization
Blue
Green
Red
Near-IR
DN values before and after radiance nomalization of Image (20130115_4_New)
DN values of Base Image (20150609_4_New)
0
20
40
60
80
0
20
40
60
80

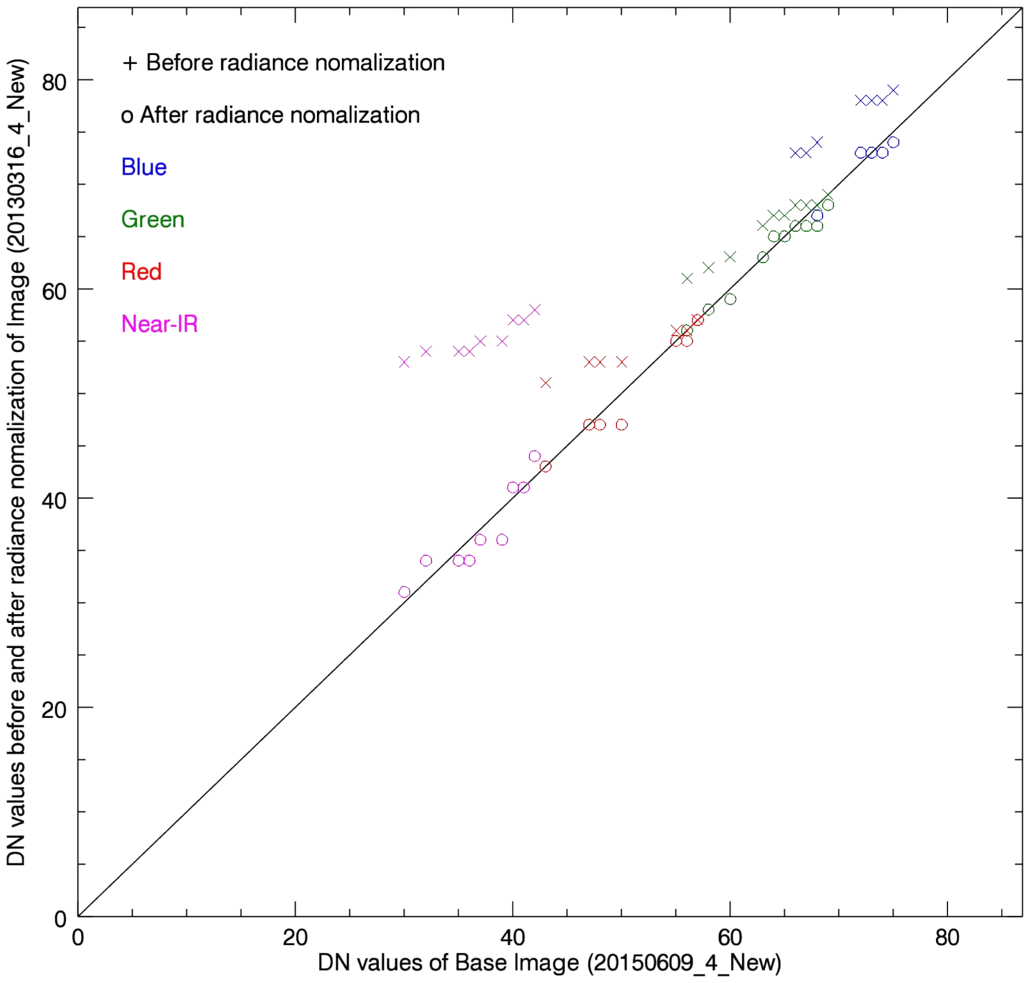
+ Before radiance nomalization
o After radiance nomalization
Blue
Green
Red
Near-IR
DN values before and after radiance nomalization of Image (20130316_4_New)
DN values of Base Image (20150609_4_New)
0
20
40
60
80
0
20
40
60
80

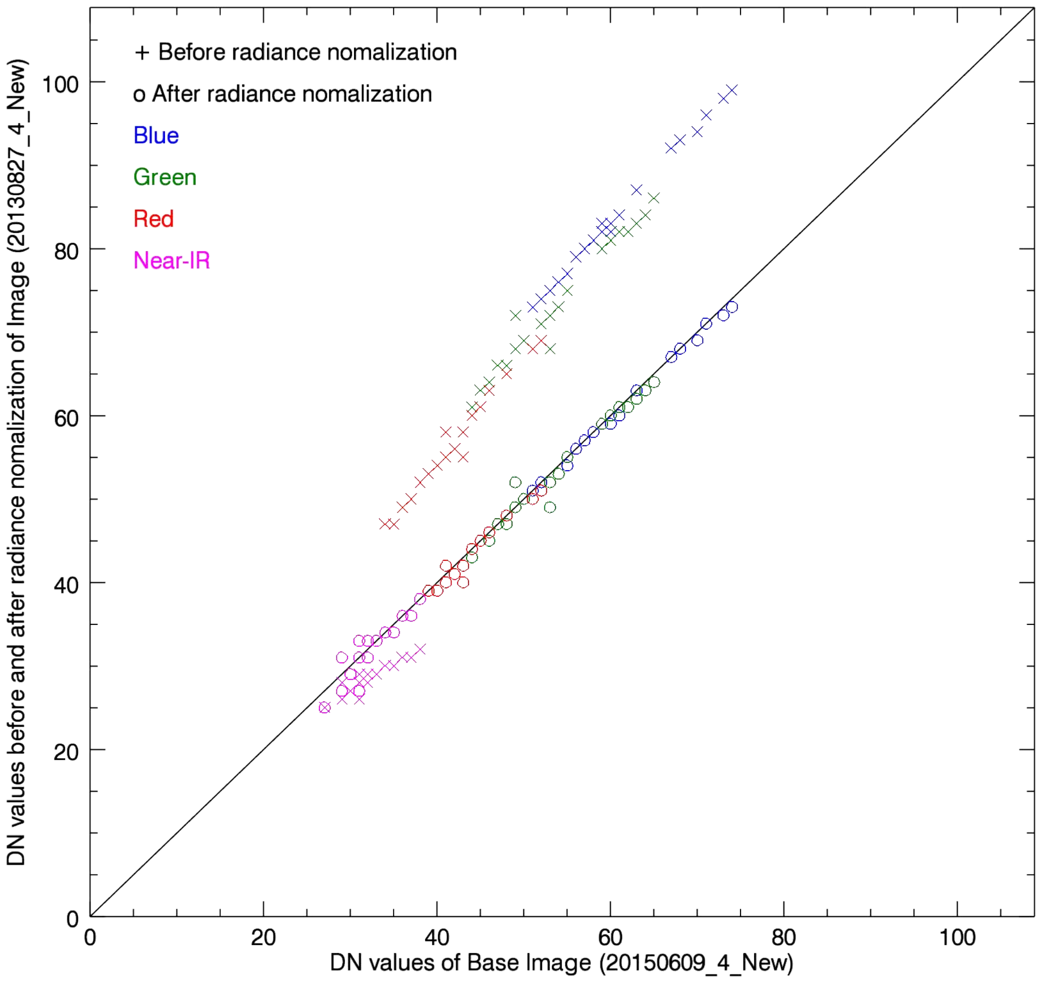

+ Before radiance nomalization
o After radiance nomalization
Blue
Green
Red
Near-IR
DN values before and after radiance nomalization of Image (20130827_4_New)
DN values of Base Image (20150609_4_New)
0
20
40
60
80
100

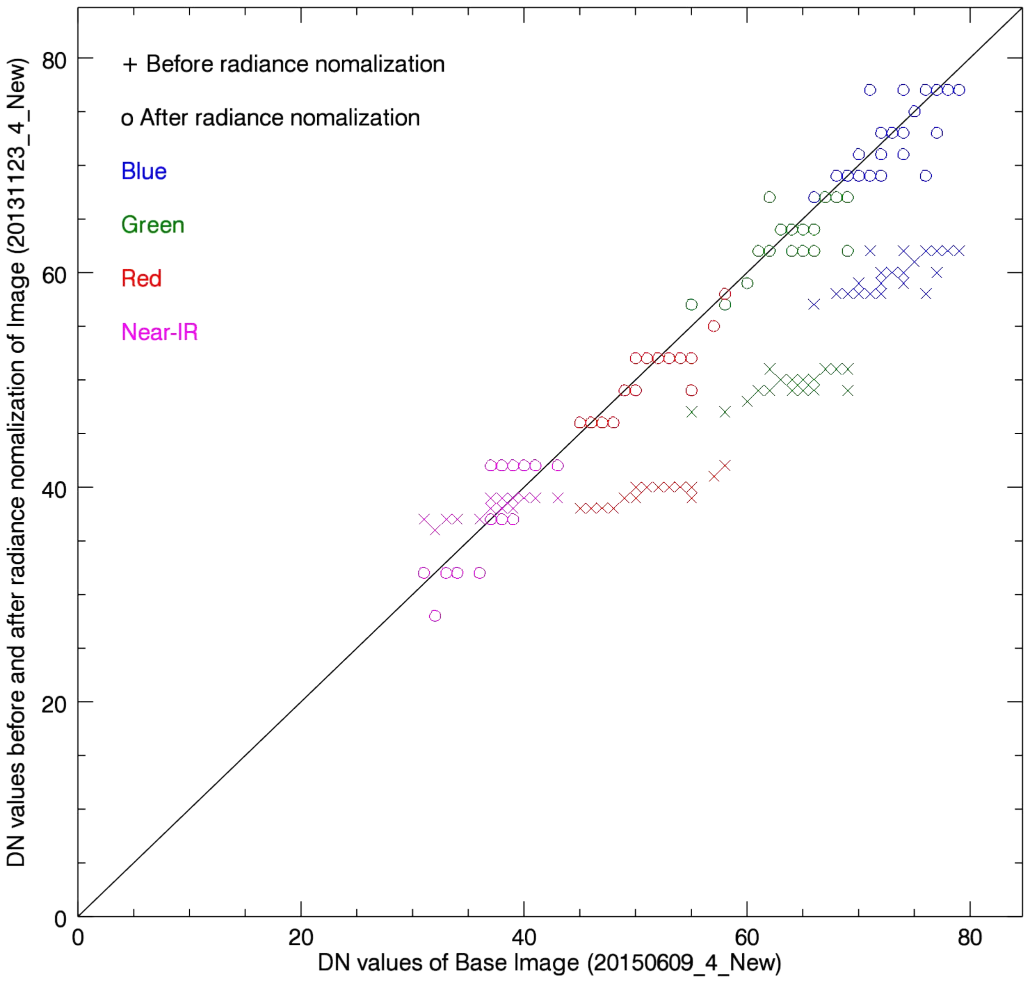

+ Before radiance nomalization
o After radiance nomalization
Blue
Green
Red
Near-IR
DN values before and after radiance nomalization of Image (20131123_4_New)
DN values of Base Image (20150609_4_New)
0
20
40
60
80
0
20
40
60
80

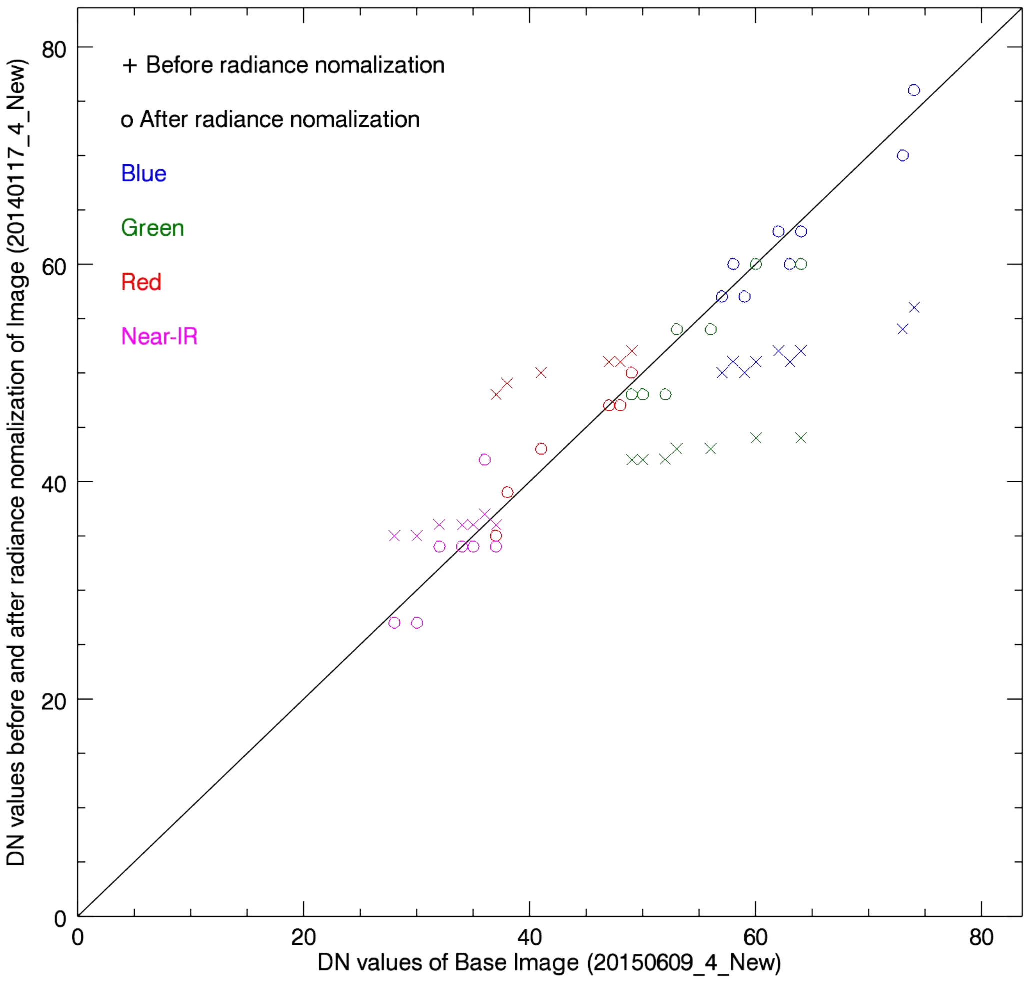

+ Before radiance nomalization
o After radiance nomalization
Blue
Green
Red
Near-IR
DN values before and after radiance nomalization of Image (20140117_4_New)
DN values of Base Image (20150609_4_New)
0
20
40
60
80

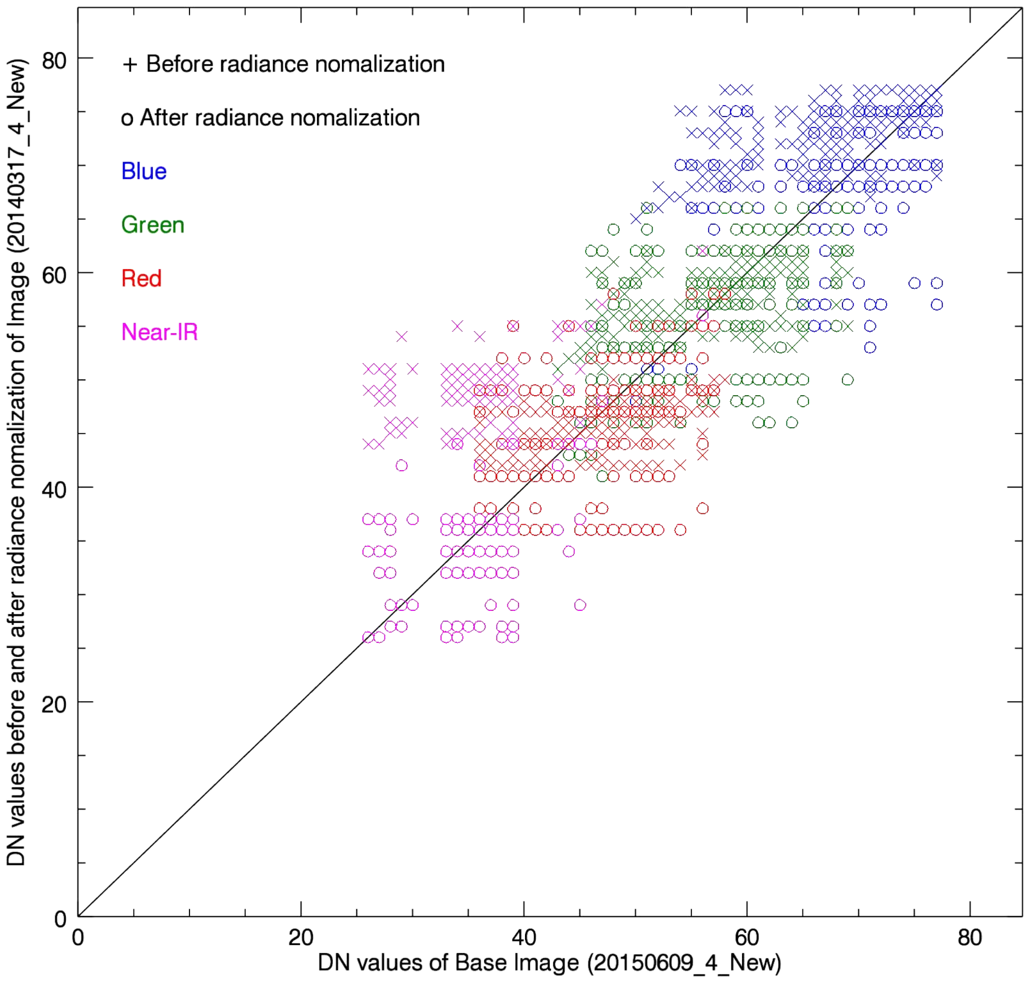

+ Before radiance nomalization
o After radiance nomalization
Blue
Green
Red
Near-IR
0
20
40
60
80
DN values of Base Image (20150609_4_New)
DN values before and after radiance nomalization of Image (20140317_4_New)

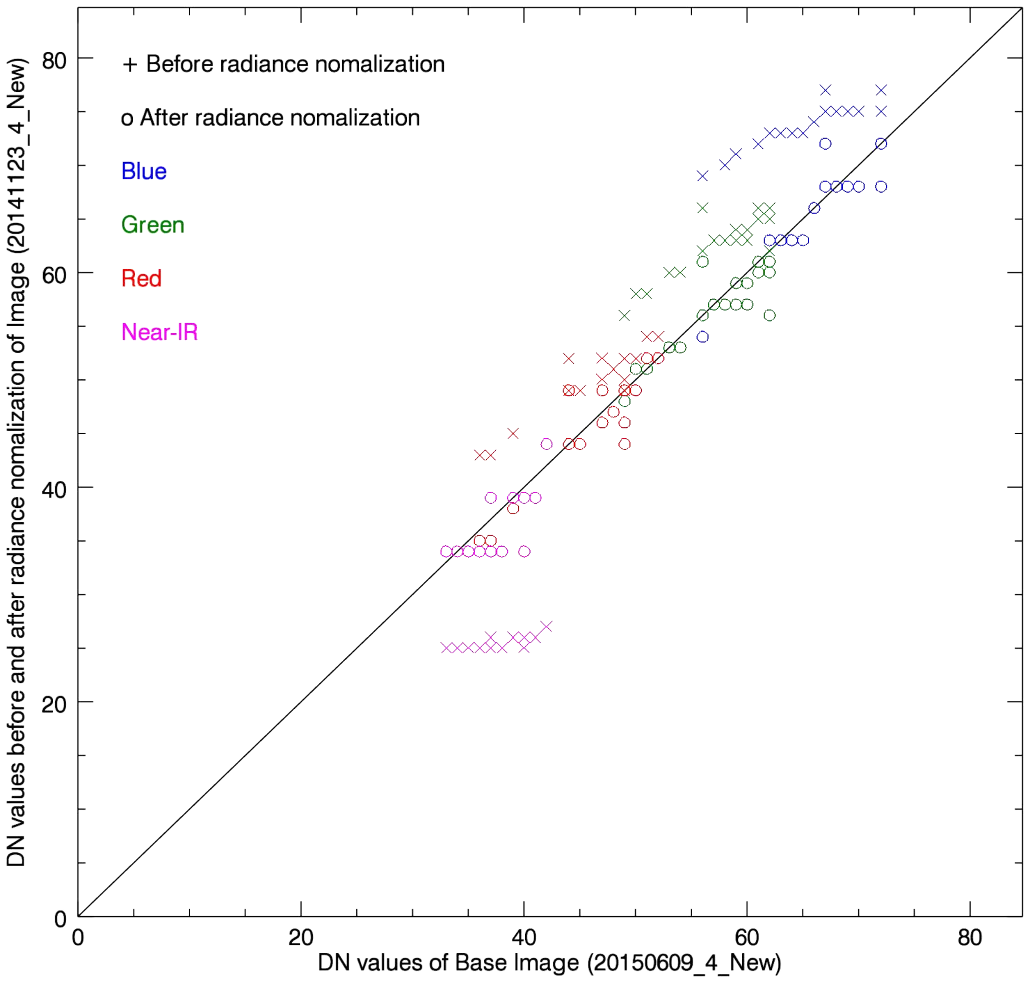

+ Before radiance nomalization
o After radiance nomalization
Blue
Green
Red
Near-IR
DN values before and after radiance nomalization of Image (20141123_4_New)
DN values of Base Image (20150609_4_New)
0
20
40
60
80

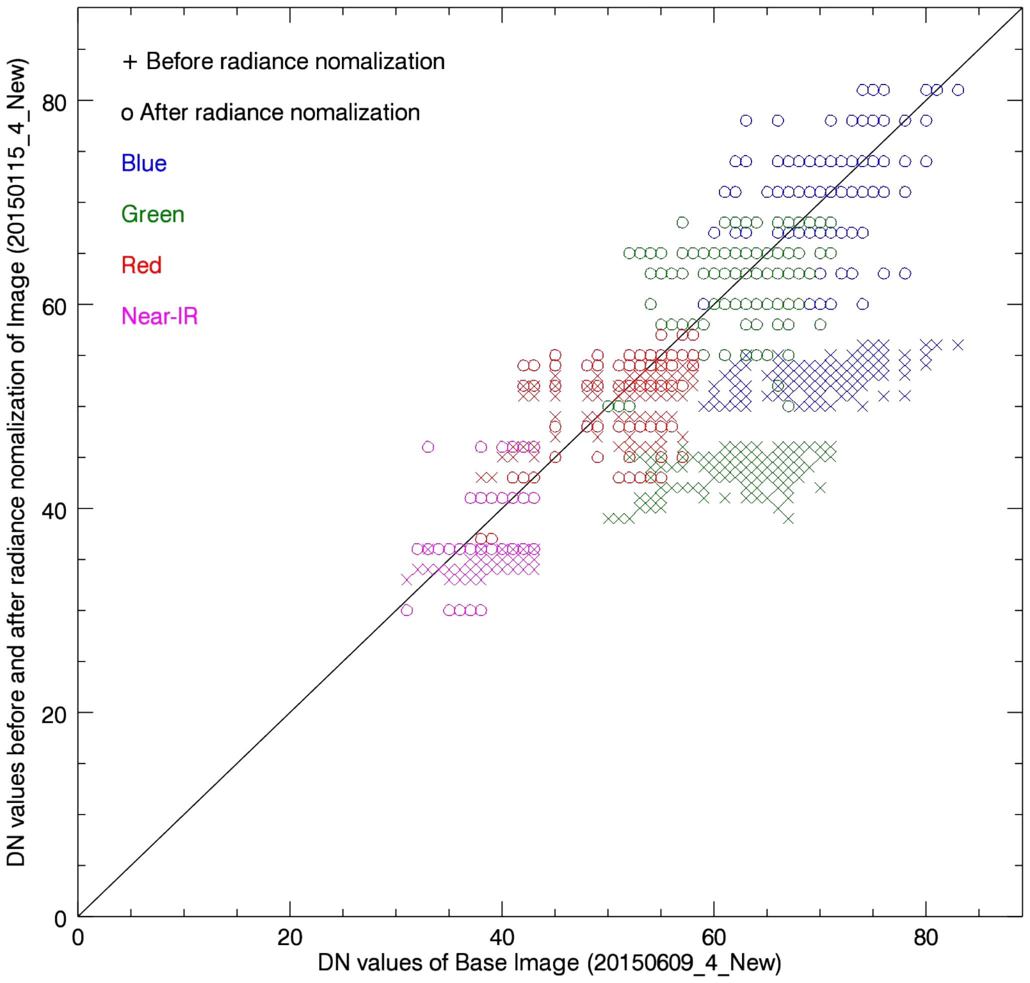
+ Before radiance nomalization
o After radiance nomalization
Blue
Green
Red
Near-IR
DN values before and after radiance nomalization of Image (20150115_4_New)
DN values of Base Image (20150609_4_New)
0
20
40
60
80

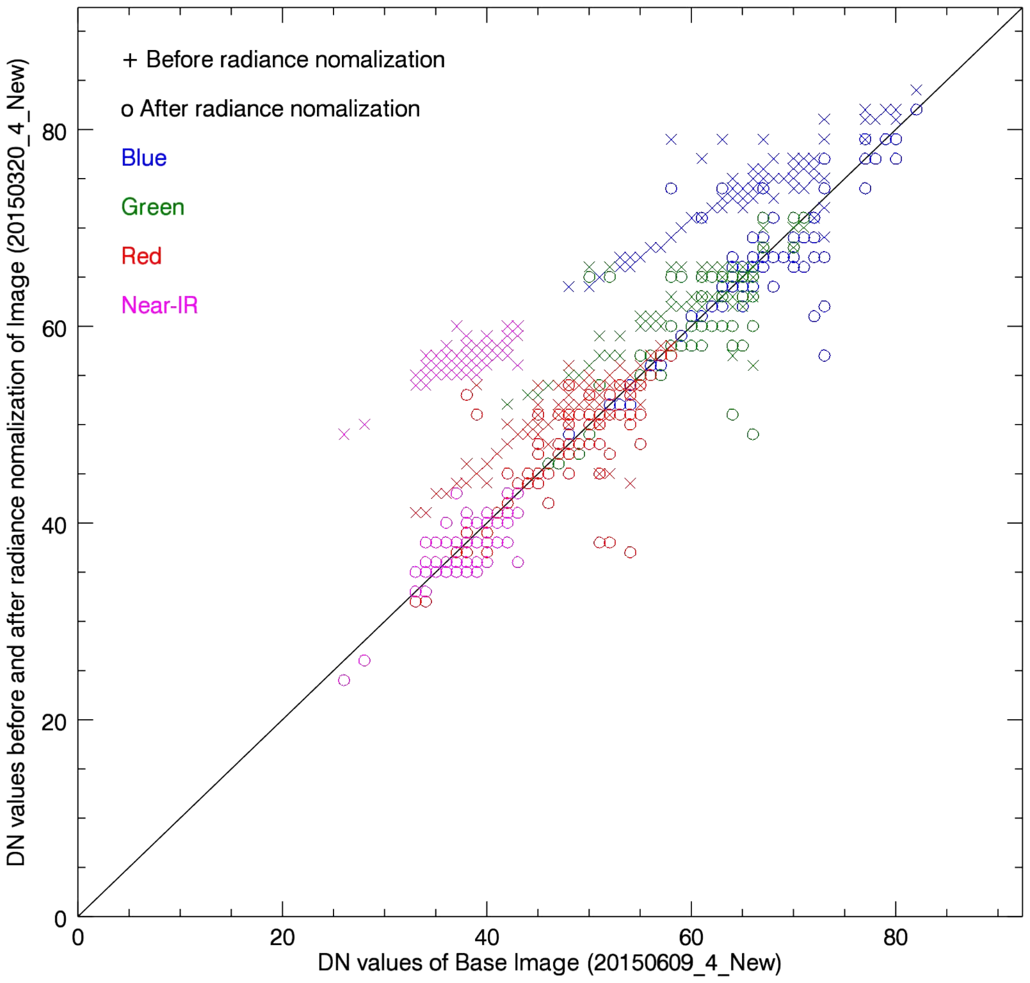

+ Before radiance nomalization
o After radiance nomalization
Blue
Green
Red
Near-IR
DN values before and after radiance nomalization of Image (20150320_4_New)
DN values of Base Image (20150609_4_New)
0
20
40
60
80
0
20
40
60
80

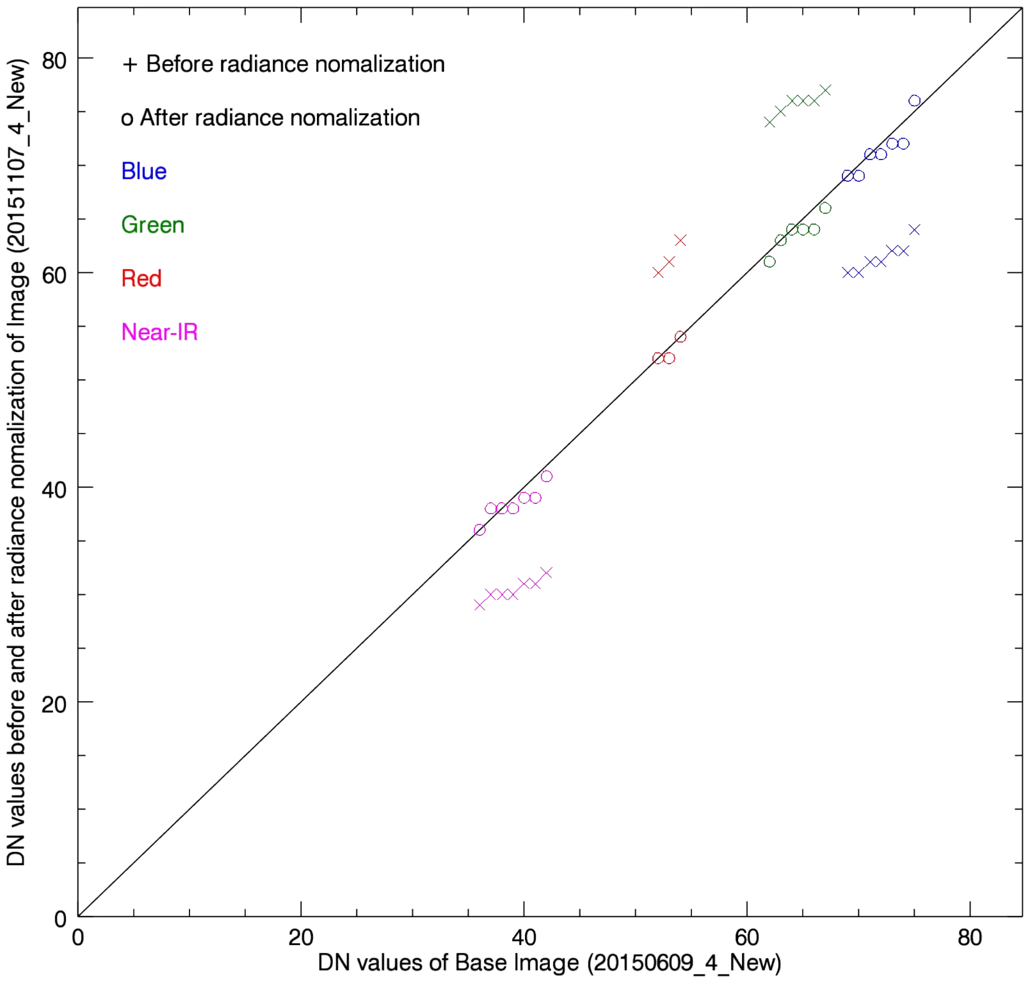

+ Before radiance nomalization
o After radiance nomalization
Blue
Green
Red
Near-IR
0
20
40
60
80
DN values before and after radiance nomalization of Image (20151107_4_New)
DN values of Base Image (20150609_4_New)

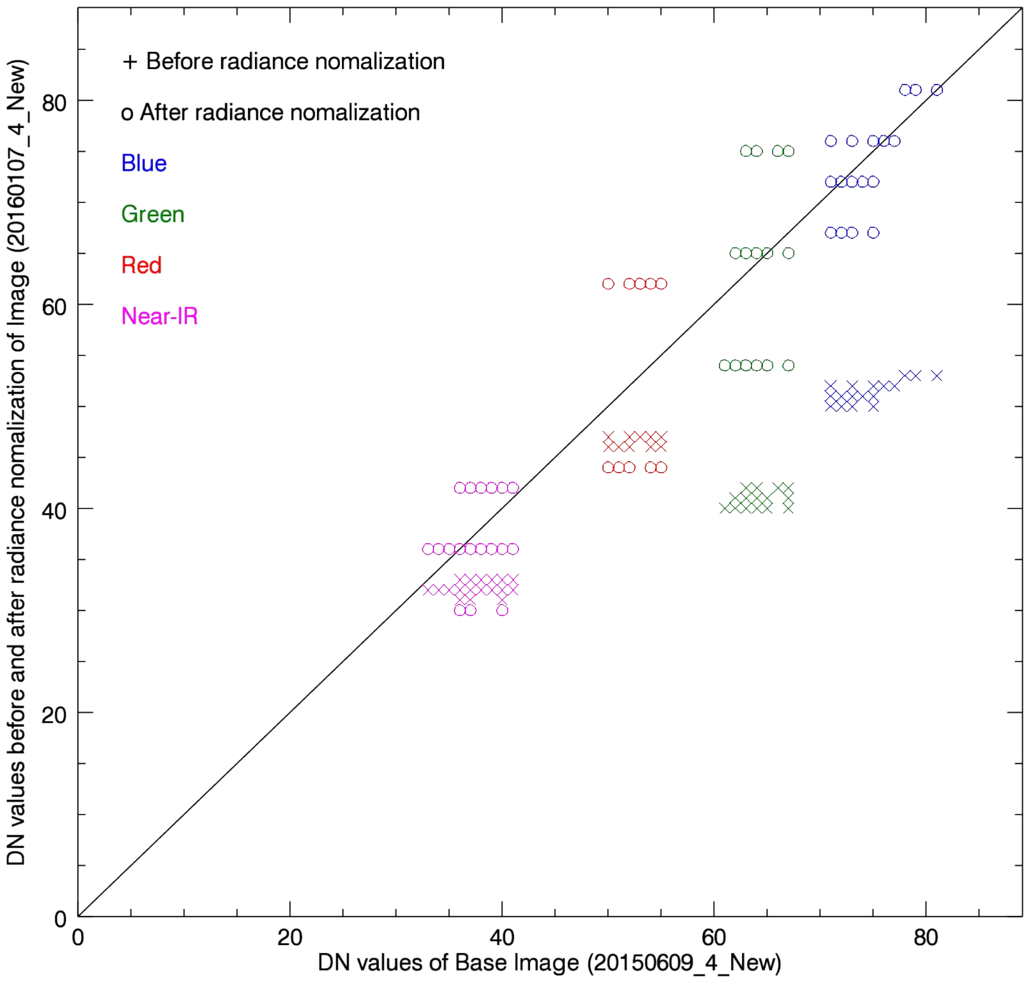

+ Before radiance nomalization
o After radiance nomalization
Blue
Green
Red
Near-IR
0
20
40
60
80
DN values of Base Image (20150609_4_New)
DN values before and after radiance nomalization of Image (20160107_4_New)

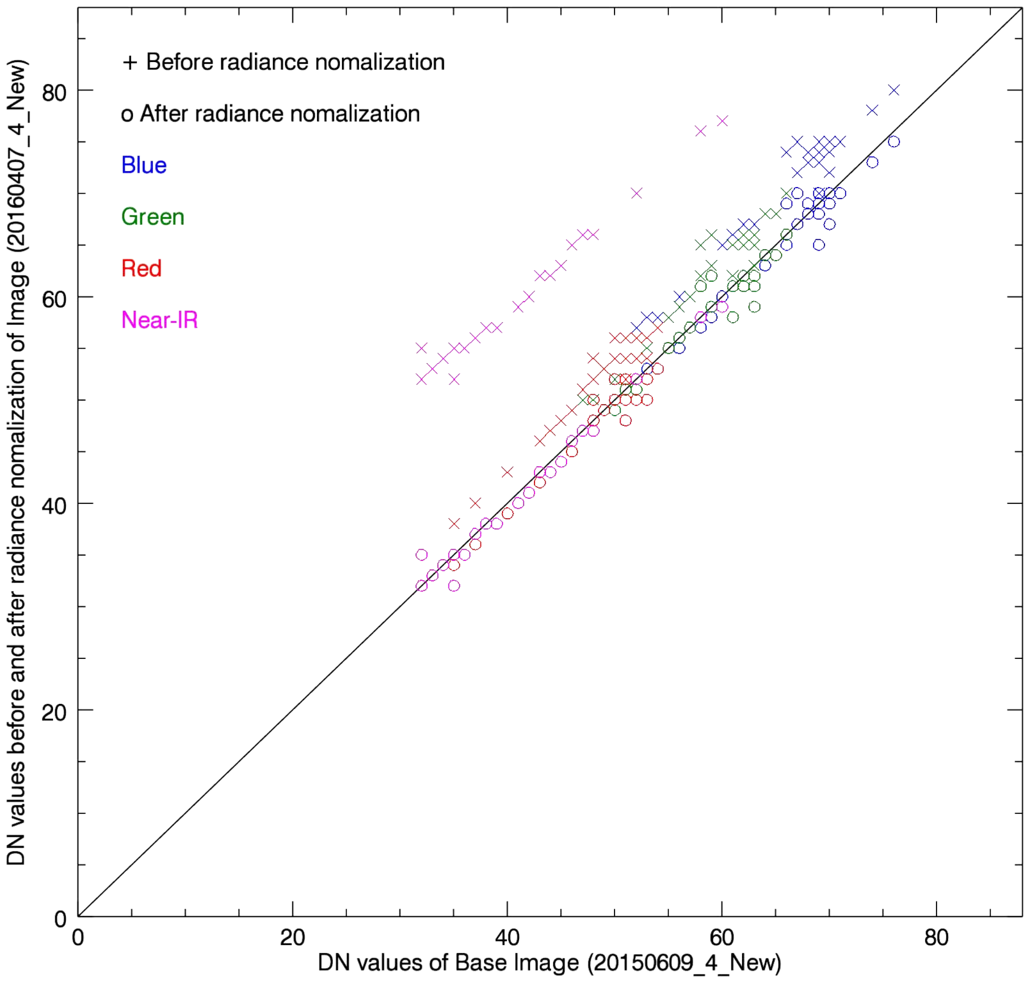
+ Before radiance nomalization
o After radiance nomalization
Blue
Green
Red
Near-IR
DN values before and after radiance nomalization of Image (20160407_4_New)
DN values of Base Image (20150609_4_New)
0
20
40
60
80
